# Circulating miR-146a as a possible candidate biomarker in the indeterminate phase of Chagas disease

**DOI:** 10.1186/s40659-021-00345-3

**Published:** 2021-07-21

**Authors:** Martha Alicia Ballinas-Verdugo, Rogelio Frank Jiménez-Ortega, Eduardo Martínez-Martínez, Nancy Rivas, Erick Abraham Contreras-López, Roxana Carbó, Fausto Sánchez, Rafael Bojalil, Ricardo Márquez-Velasco, Fausto Sánchez-Muñoz, Ricardo Alejandre-Aguilar

**Affiliations:** 1 Departamento de Inmunología, Instituto Nacional de Cardiología Ignacio Chávez, Mexico City, CDMX Mexico; 2 Departamento de Parasitología, Escuela Nacional de Ciencias Biológicas, Instituto Politécnico Nacional, Mexico City, CDMX Mexico; 3 Licenciatura en Nutrición, Plantel Texcoco, Universidad Privada del Estado de México, Texcoco, Estado de México Mexico; 4 Instituto Nacional de Medicina Genómica, Mexico City, CDMX Mexico; 5 Instituto Mexicano del Seguro Social, Hospital General Regional No. 25 Zaragoza, Mexico City, CDMX Mexico; 6 Departamento de Biomedicina Cardiovascular, Instituto Nacional de Cardiología Ignacio Chávez, Mexico City, CDMX Mexico; 7 División de Ciencias Biológicas y de La Salud, Universidad Autónoma Metropolitana Xochimilco, Mexico City, CDMX México; 8 Sección de Postgraduados, Instituto Politécnico Nacional, Escuela Superior de Medicina, Mexico City, CDMX Mexico

**Keywords:** *Trypanosoma cruzi*, Chagas disease, microRNAs, Extracellular vesicles (EVs), Chronic Chagas cardiomyopathy

## Abstract

**Background:**

Chagas disease is considered important and presents intense inflammatory and fibrotic processes induced by the perpetuation of the parasite in the affected tissues and organs. Therefore, it is necessary to inquire about the host defense and attack mechanisms to have a more detailed knowledge about Chagas disease. MicroRNAs are found in blood, tissues and extracellular vesicles. These small regulators of gene expression are involved in physiological and pathological processes in both mammals and parasites. Several microRNAs have deregulated expression in chagasic heart disease, although little is known about their extracellular expression. Our main objective was to evaluate the involvement of miR-21, miR-146a and miR-155 in several samples from mice infected with the TcI Ninoa strain from the acute and indeterminate phases. We also explored a potential functional association of the selected microRNAs using STRING software. This software identified 23 pathways associated with *Trypanosoma cruzi* infection. In addition, eleven genes were identified through bioinformatics analysis, and we found that SMAD family member 5 was downregulated in both phases. This gene serves as a mediator in the TGF-β signaling pathway. Thus, forty female mice of the CD1 strain were distributed into 4 groups and the expression levels of miR-21, miR-146a and miR-155 were measured in samples of heart tissue, total plasma and plasma extracellular vesicles by quantitative real-time polymerase chain reaction.

**Results:**

Overexpression of miR-21, miR-146a and miR-155 was observed in heart and plasma in both phases. Moreover, in extracellular vesicles miR-21 and miR-146a were also overexpressed in the acute phase, whereas in the indeterminate chronic phase we found only miR-146a up-regulated.

**Conclusions:**

The expression of inflammatory microRNAs miR-21, miR-146a and miR-155 were up-regulated in each of the samples from acutely and chronically infected mice. The relevant finding was that miR-146a was up-regulated in each sample in both phases; therefore, this miRNA could be a possible candidate biomarker in Chagas disease.

**Supplementary Information:**

The online version contains supplementary material available at 10.1186/s40659-021-00345-3.

## Background

Several mechanisms are involved in the pathogenesis of Chagas disease. These mechanisms explain the host response to *Trypanosoma cruzi* (*T. cruzi*) infection and adaptive immunity to *T. cruzi* infection [1]. Chagas disease is due to intense inflammatory injury and induced fibrosis in tissues and organs involved in the infectious cycle of the parasite. If this disease is not treated in time, it can damage the heart muscle tissue, leading to chronic chagasic cardiomyopathy (CCC). The CCC is characterized by progressive manifestations, such as fibrosis, myocyte destruction, left ventricular hypertrophy, arrhythmia, intraventricular block (right bundle branch block) that can lead to heart failure [2–4].

One of mechanisms of Chagas pathogenesis is to lead toward parasite products, cellular invasion and intracellular replication. Another is by the implication of the cell-mediated immunity, such as T cells with Th1 and Th17 response and the humoral immunity that is characterized by polyclonal activation of B cells. These mechanisms explain the host response to *T. cruzi* infection and the adaptative immunity to *T. cruzi* infection [2].

These mechanisms are also result of the biochemistry and great genetic diversity of *T. cruzi* strains, which have been studied during many years [4]. Some studies have provided a breakthrough in the knowledge of *T. cruzi* diversity [5–7]. Currently, six *T. cruzi* discrete typing units (DTU) TI-TVI are known by multilocus genotyping [3]. Ruíz-Sánchez et al. determined in 2005 that some Mexican isolates are predominantly TcI DTU [8]. Ninoa is a cardiotrophic and myotropic strain and is classified into TcI [7, 9, 10].

Unicellular and multicellular communication can take place through indirect and direct interactions. The parasite needs to evade the immune response to establish infection and the host has to respond to it through cellular communication. One of the mechanisms of communication between host and parasite is through extracellular vesicles (EVs). EVs play an important role as mediators of cellular communication. In addition to communicating, EVs are involved in the secretion and transfer of soluble factors, such as virulence factors, lipoproteins, complement factors, cytokines, bioactive metabolites and nucleic acids. These bioactive entities are released by many cells of diverse origins. Moreover, these molecules can be carried by surrounding cells to different cellular targets through blood and other body fluids [11–15]. Above all, EVs can be an excellent study model for tracking molecules such as microRNAs (miRNAs) with a potential role as biomarkers for early disease detection.

MiRNAs are small (19–22 nucleotide) non-protein-coding RNAs, which regulate gene expression post-transcriptionally through binding to the 3ʹ untranslated regions (UTR) of messenger RNA (mRNA). These molecules play an important role in multiple biological processes such as: cell proliferation, differentiation, apoptosis, and immunity, and alterations in miRNA expression profiles can lead to or induce the development of multiple diseases [16]. In cardiovascular diseases, Corsten et al. [17] explored the plasma levels of miRNAs that are associated with heart fibrosis and leukocyte infiltration, among them are miR-21 miR-146 and miR-155. While in *T. cruzi* infection, Rodriguez et al. [18] analyzed miR146a-5p and miR-155-5p expression in CCC; Navarro et al. [19] also explored miR-21 expression in colombiana *T. cruzi* strain acute infection and Nonaka et al. [20] recently analyzed miR-21-5p in indeterminate phase and CCC. Three miRNAs are involved in biological mechanisms such as control of TRL- receptor (miR-146a), regulation of TGF- β (miR-21) and susceptibility to *T. cruzi* infection (miR-155) and their target genes are interleukin 1 receptor-associated kinase (*Irak*) 1, TNF receptor-associated factor (*Traf*) 6, *Smad7* and collagen type I alpha 1 (*Col1a1*) [18–22] Currently, miRNAs have been suggested as therapeutic candidates and potential biomarkers for the diagnosis and early detection of cardiovascular diseases and various parasitic diseases, such as Chagas disease. These diagnostic tools can be easily monitored in body fluids such as serum, plasma and blood [23]. Thus, Ji et al. suggested miR-208 [24] and Adachi et al. proposed miR-499 [25] as biomarkers of myocardial injury and acute myocardial infarction, respectively. On the other hand, Linhares et al. [26] proposed miR-208a as a possible biomarker in indeterminate Chagas disease [24–26]. Thus, the aim of this study is to evaluate the involvement of miRNAs miR-21, miR-146a and miR-155 in several samples from mice infected with the Mexican TcI Ninoa strain and their role as possible potential biomarkers for early detection of Chagas disease.

## Results

### Infection and histology

**Fig. 1:**
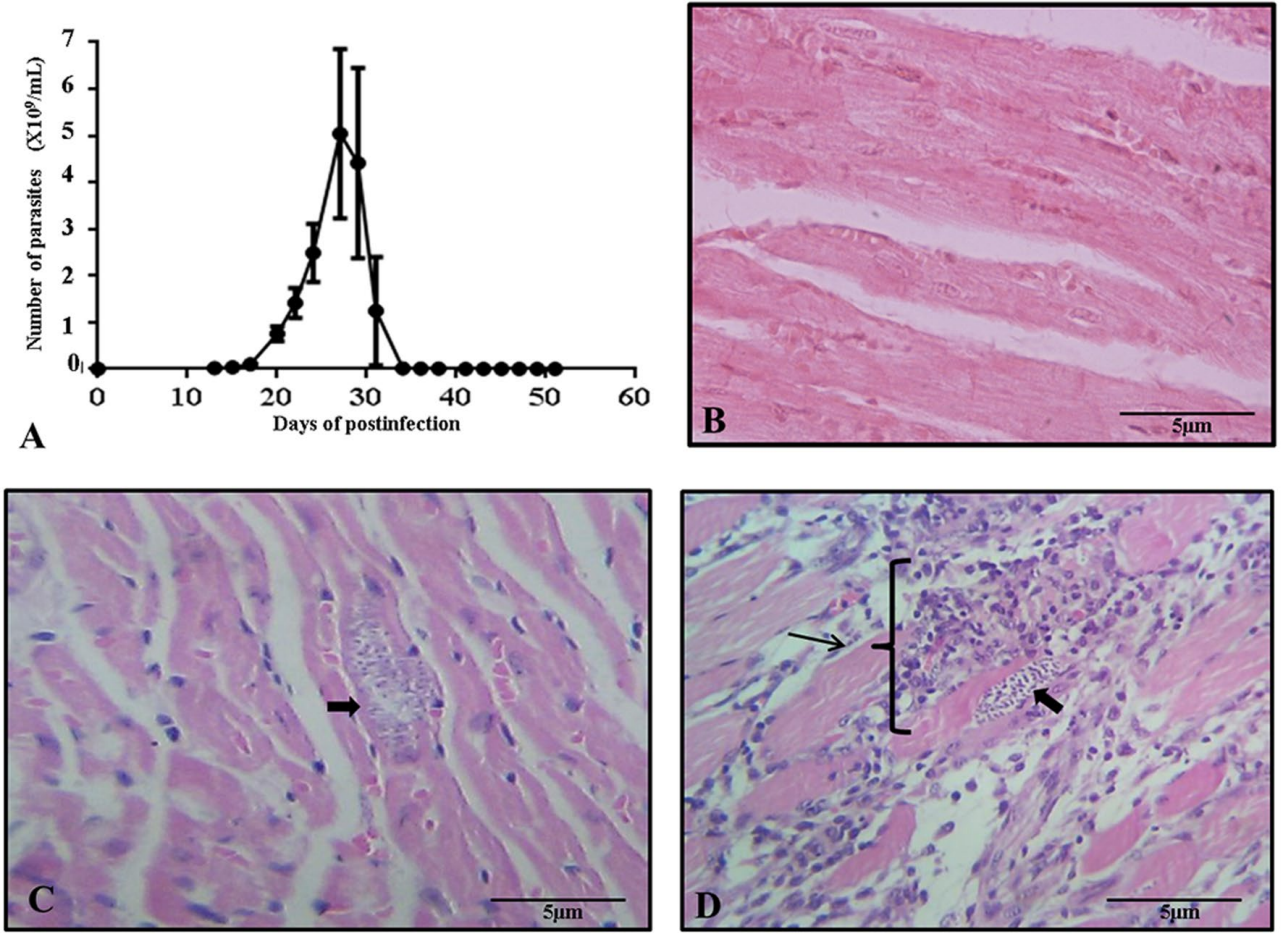
Infection and histology. **A** Parasitemia curve of *T. cruzi* Ninoa strain in mice over 60 days. **B** Histological section of normal cardiac tissue of mouse. **C** Histological section of cardiac tissue is shown during the acute phase; the arrow indicates the presence of amastigote nests with slight presence of mature lymphocytes. **D** Histological section of cardiac tissue is shown during the indeterminate phase; the arrows indicate amastigote nest, the presence of mature lymphocytes, and a severe inflammatory state with a diagnosis of myocarditis. Histological sections were stained by Hematoxylin–Eosin and observed to 40 X

Infected mice showed a maximum parasitemia peak at 27 days post-infection with 5.04 × 10^9^ parasites/ml, with parasites observed in blood unti3 day 47 post-infection (Fig. 1A). There was 83.33% mortality between 27- and 34-days post-infection; only one specimen survived until the end of the curve of parasitemia (Additional file 1: Table S1). Histological sections of cardiac tissue showed differences between control (Fig. 1B), infected cardiac tissue (Fig. 1C) from the acute phase and infected cardiac tissue (Fig. 1D) from the indeterminate phase. During the acute phase, the first nests of amastigotes started to be present in the tissue, slight presence of mature lymphocytes without apparent inflammation. However, in the indeterminate phase, moderate amounts of amastigotes nests were observed, in addition to showing an obvious focus of inflammation with few mature lymphocytes and a severe inflammatory state. As a result of the histopathological examination, the diagnosis of myocarditis was established.

### MiRNAs expression by RT-qPCR

**Fig. 2:**
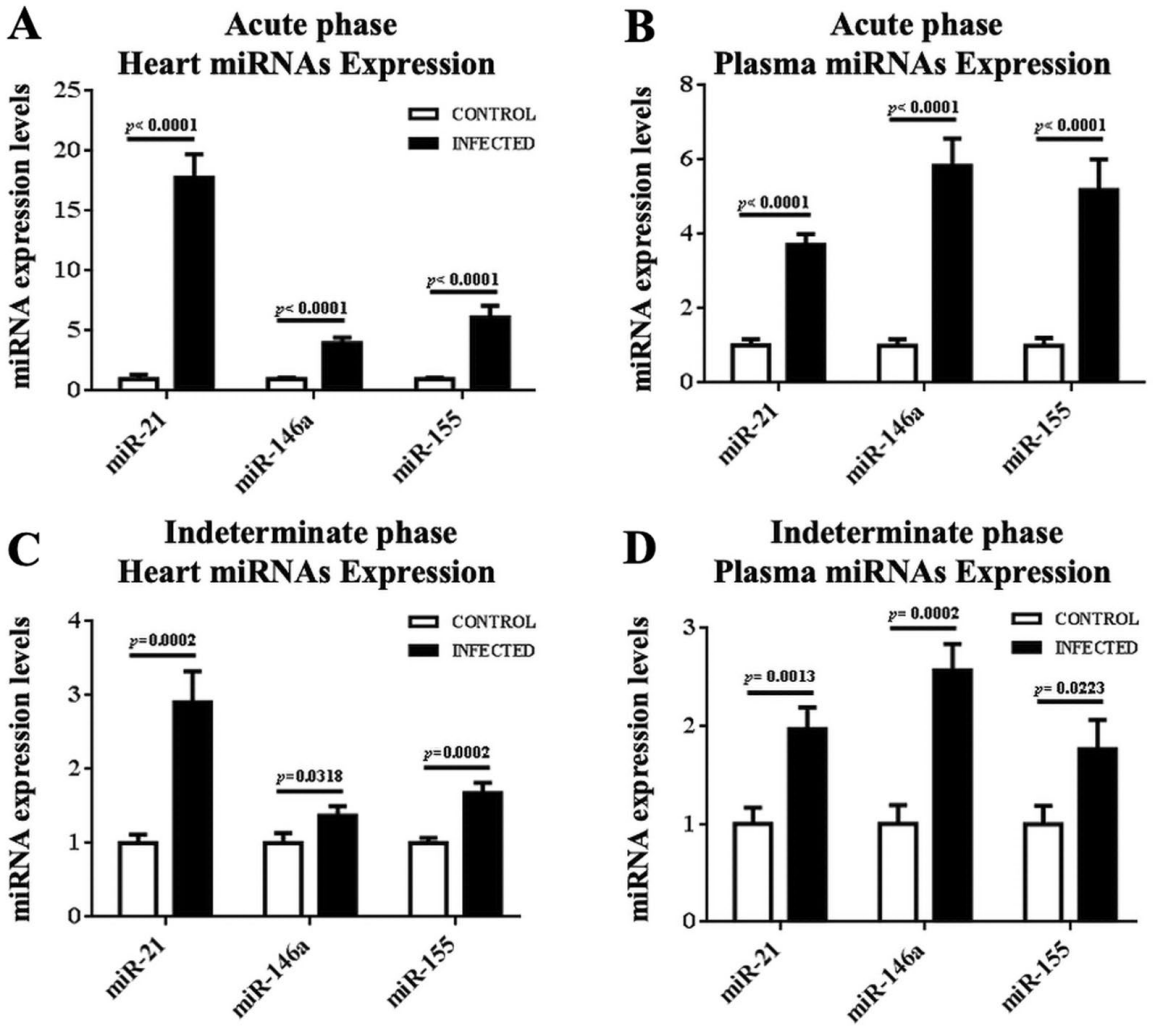
Expression levels of miRNAs from heart and plasma in the acute and indeterminate phase. **A**, **C** The expression levels of miRNAs are shown: miR-21, miR146a, miR-155 with its control of cardiac tissue in both phases. **B**, **D** The expression levels of miRNAs are shown: miR-21, miR146a, miR-155 with its control of plasma in both phases. Cardiac tissue samples were normalized to U6 and plasma samples were normalized to cel-miR-39. The expression was normalized to 1 with housekeeping genes respectively used in both samples

RT-qPCR was performed to prove the expression levels of the three selected miRNAs: miR-21, miR-146a and miR-155. These miRNAs showed a statistically significant high expression level in cardiac tissue (Fig. 2A). The expression of miR-21 (17.75 ± 1.99 relative units [R.U.]), miR-146a (3.99 ± 0.46 R.U.) and miR-155 (6.13 ± 0.96 R.U.) was higher in infected mice compared with their control. Plasma expression of miR-21 (3.72 ± 0.28 R.U.), miR-146a (5.83 ± 0.74 R.U.) and miR-155 (5.18 ± 0.83 R.U.) was also higher in infected mice compared to control mice (Fig. 2B) during the acute phase of the disease.

On the other hand, during the indeterminate phase, the expression levels of miRNAs were also higher. The results show statistically significant levels of miR-21 (2.91 ± 0.42 R.U.), miR-146a (1.37 ± 0.1303 R.U.) and miR-155 (1.68 ± 0.14 R.U.) in cardiac tissue with respect to control (Fig. 2C). Plasma up-regulation was observed in Fig. 2D: miR-21 (1.97 ± 0.22 R.U.), miR-146a (2.57 ± 0.27 R.U.) and miR-155 (1.76 ± 0.30 R.U.). Again, miR-146a expression was more significant in plasma, and miR-21 expression in the heart.

### Distribution and counting of particles and expression of miRNAs EVs

**Fig. 3:**
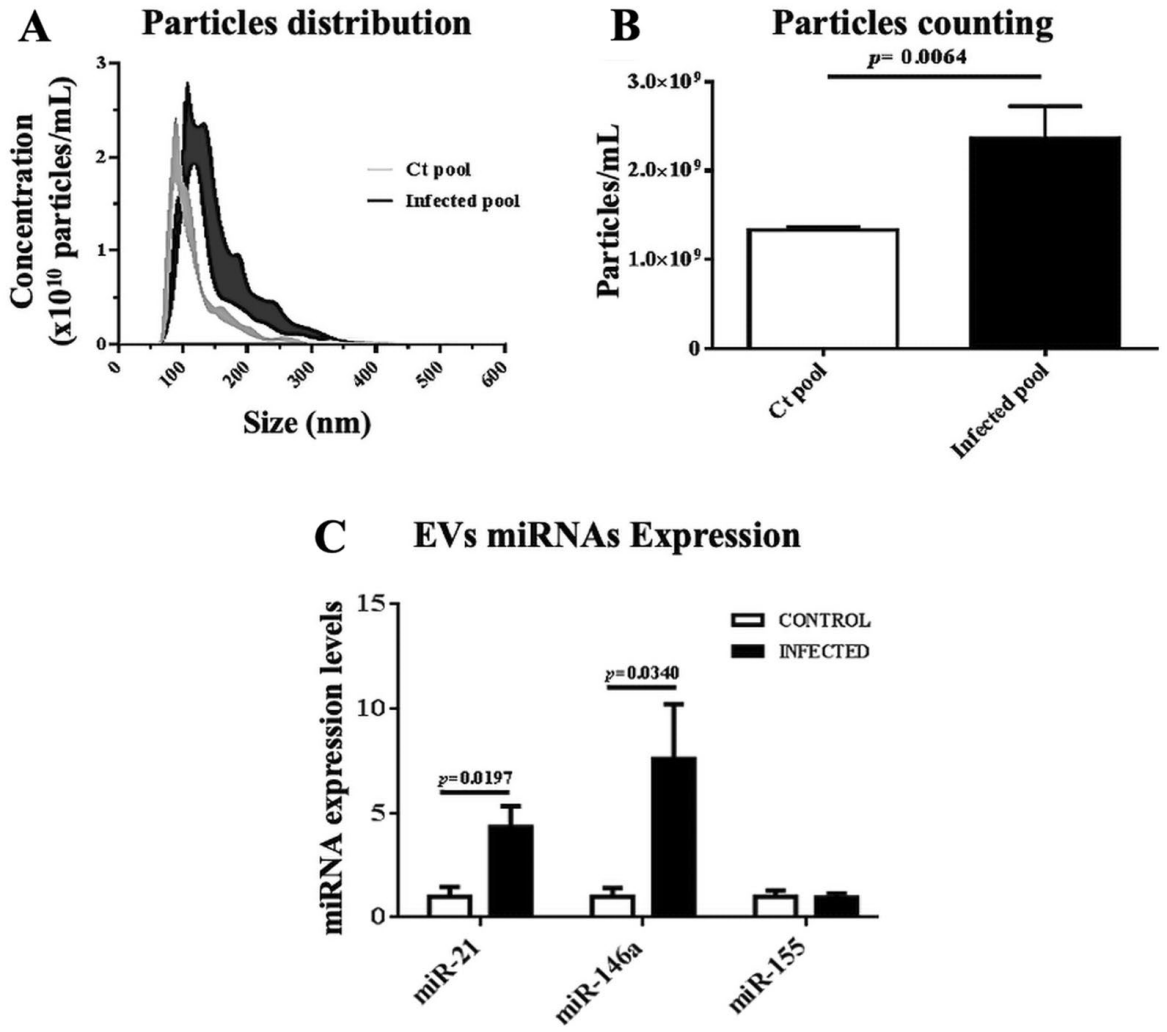
**A** Particles distribution, **B** particles counting and **C** miRNAs expression of EVs in the acute phase. MiRNAs expressions of EVs were normalized to cel-miR-39. The expression was normalized to 1 with housekeeping genes used

**Fig. 4:**
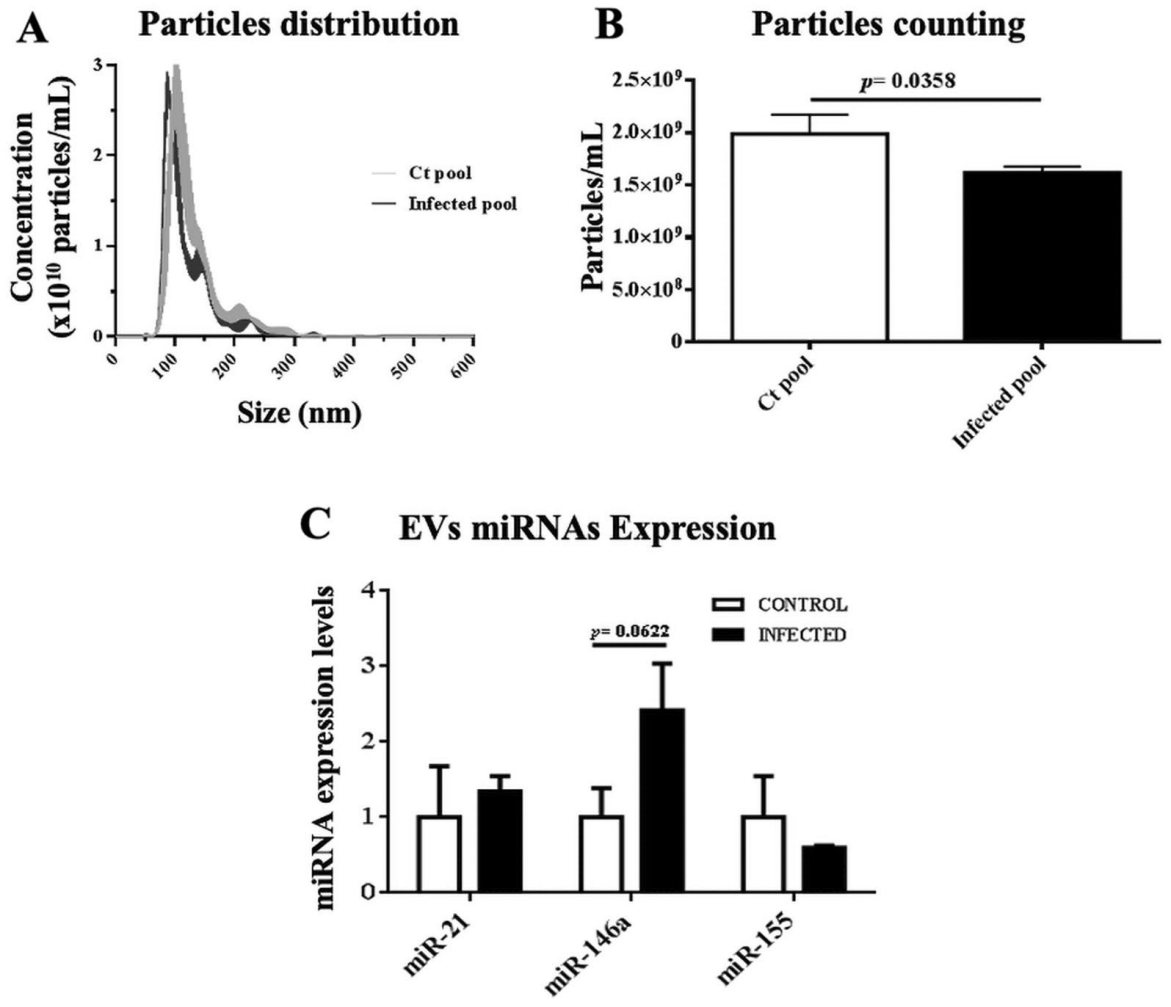
**A** Particles distribution, **B** particles counting and **C** miRNAs expression of EVs in the indeterminate phase. MiRNAs expressions of EVs were normalized to cel-miR-39. The expression was normalized to 1 with housekeeping genes used

To have a first approximation to the evaluation of the total number of EVs present in the plasma, particle counting was performed during the acute phase of the disease, a higher number of particles/mL was observed in infected mice (2361 × 10^9^) with a size of 75 nm to 321 nm compared to the control group (1334 × 10^9^) with a size of 65 nm to 213 nm (Fig. 3A, B). In Fig. 3C, the graph represents the expression of miRNAs of EVs during the acute phase; miR-21 (4.33 ± 0.99 R.U.) and miR-146a (7.6 ± 2.62 R.U.) showed a high expression level in infected mice. Whereas, in the indeterminate phase, infected mice had slightly lower concentration of 1,616 × 10^9^ particles/mL with a size of 69 nm to 179 nm, when compared to their control group with a concentration of 1,987 × 10^9^ particles/mL and a size of 71 nm to 199 nm(Fig. 4A, B). However, we only observed up-regulated miR-146a (2.41 ± 0.62 RU) in the indeterminate phase (Fig. 4C) of infected mice relative to their control.

### Predicted target genes of miRNAs associated with *T. cruzi* infection

We predicted mRNA target genes for miR-21, miR-146a, and miR-155; the criteria for target identification were consistent with prediction from at least three databases, 2020 target genes for miR-21, 1771 target genes for miR-146a and 1239 for miR-155. These lists of potential target genes were linked to the data derived from the differential expression analysis of the microarrays (Additional file 2: Table S2), assuming that a high expression level of a given miRNA corresponds to a low expression level of the target gene. Down-regulation was defined as a minimum of − 0.5-FC.

**Fig. 5:**
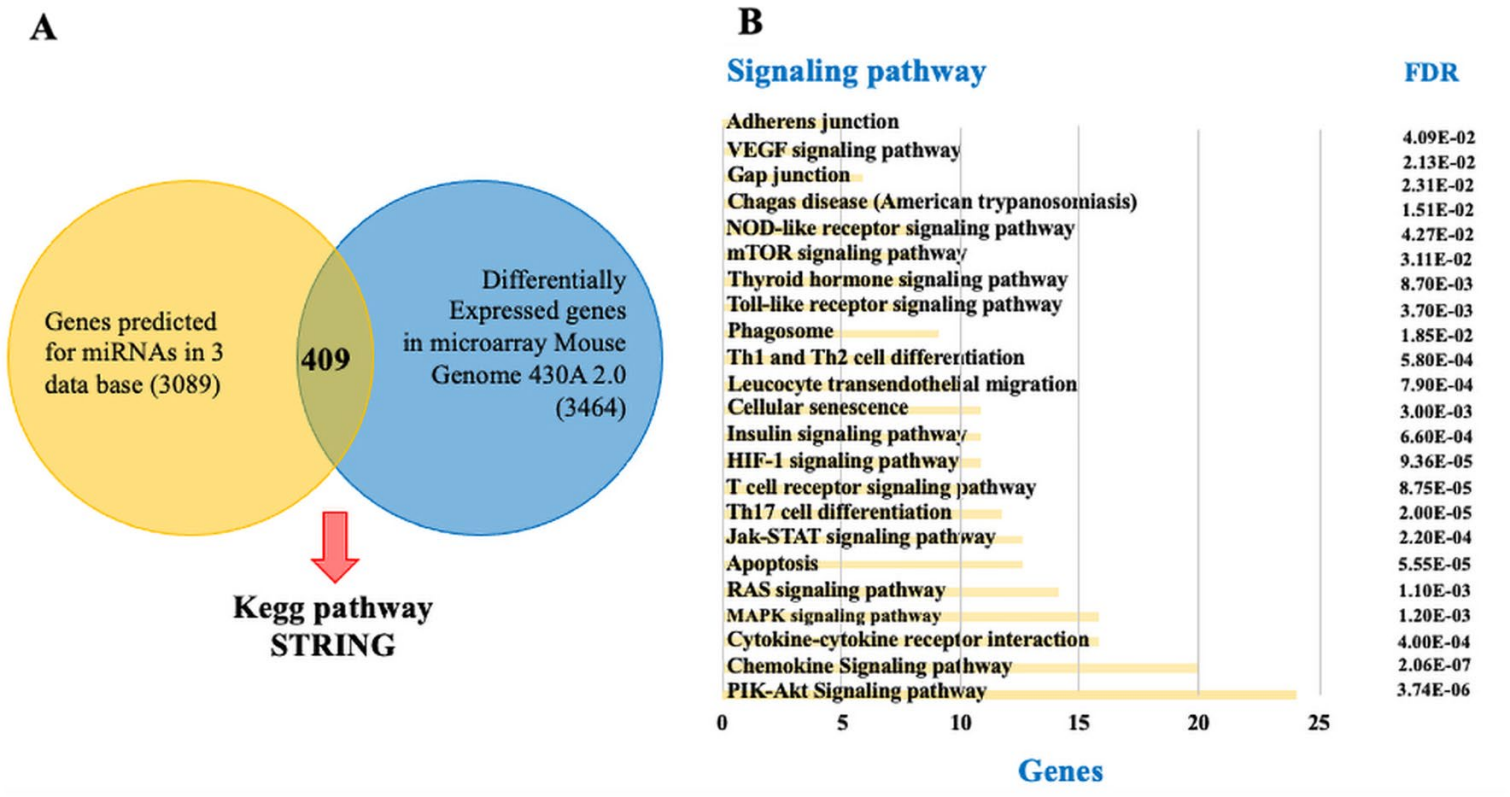
**A** Venn diagram: demonstrating the basis for selecting the potential target genes from miRNAs: mmu-miR-21, mmu-miR-146a and mmu-miR-155 by linking the list obtained from the prediction algorithms with those generated from the data of the analysis of differential expression of microarrays (3464 genes with fold-change ≤ − 0.5 or ≥ 0.5 and *p* < 0.05) and **B** STRING-KEGG: pathway enrichment analysis of the putative target genes from miRNAs of the previous analysis. *FDR* False Discovery Rate

Using this criterion, 409 putative target genes deregulated for mmu-miR-21, mmu-miR146a and mmu-miR-155 were identified. The interaction by combining the lists of target genes generated by the prediction algorithms for the three miRNAs and the microarray expression data set was depicted in Fig. 5A.

### Interaction network of target genes and miRNAs

To explore a possible functional association of the selected miRNAs, the list of 409 target genes was submitted to the STRING online bioinformatics tool to identify possible canonical pathways. The analysis revealed 23 STRING-KEGG pathways in *Mus musculus* that were associated with *T. cruzi* infection and Chagas disease according to an extensive literature review; the pathways predicted as the most markedly enriched by the three miRNAs mentioned above were PI3K-Akt, Chemokines, Cytokine-cytokine receptor interaction, MAPK, Ras, Apoptosis, Jak-STAT, Th17 cell differentiation, among others, shown in Fig. 5B.

**Fig. 6:**
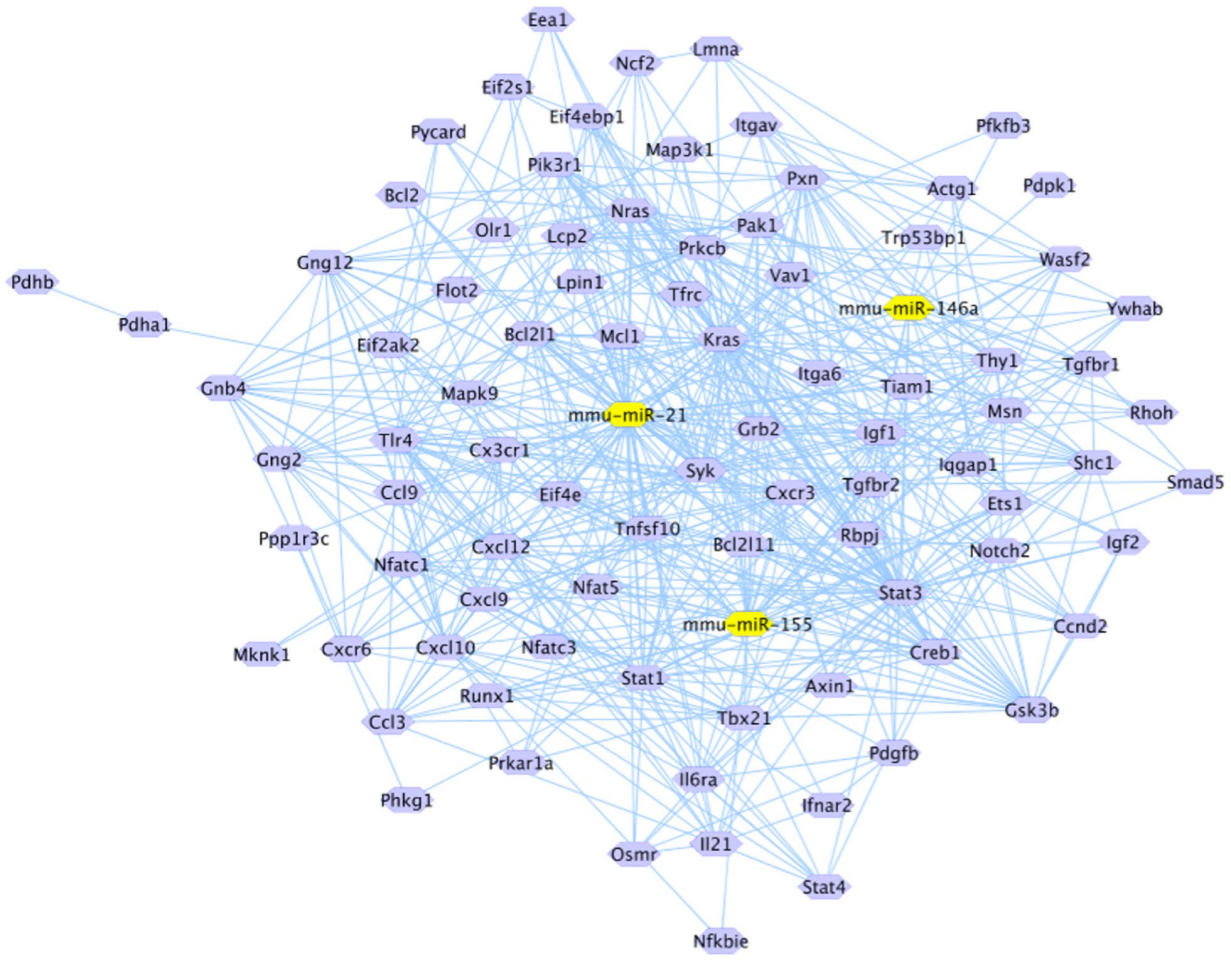
Interaction pathway of mmu-miR-21, mmu-miR-146b and mmu-miR-155 and target genes. These data reveal the interaction of 54 target genes for mmu-miR-21, 14 target genes for mmu-miR-146b and 28 target genes for mmu-miR-155. Red arrows indicate the genes related to virulence by *T. cruzi* and host immune response. CAMP Responsive Element Binding Protein 1(*Creb1*), Mitogen-Activated Protein Kinase Kinase Kinase 1 (*Map3k1*), IQ Motif Containing GTPase Activating Protein 1(*Iqgap1*), BCL2 Apoptosis Regulator (*Bcl2*), SMAD Family Member 5 (*Smad5*), Nuclear factor of activated T cells 1 (*Nfatc1*), Glicogen Synthase Kinase 3 beta (*Gsk3b*), Nuclear factor of activated T cells 5 (*Nfat5*) and Insulin like grown factor 1 receptor (*Igf1r*)

The 86 target genes associated with these pathways were analyzed by Cytoscape v3.7.2 software to construct an interaction network between miRNAs and target genes. The analysis reveals that mmu-miR-21 bound to the 3'UTR region of 54 potential target genes, mmu-miR-146a to 14 genes and mmu-miR-155 to 28 genes (Fig. 6).

### Prediction and verification of target genes

Bioinformatic analysis of the potential target genes displayed in the interaction network revealed that of the 84 genes, 9 potential target genes were strongly associated with virulence mechanisms by *T. cruzi* and the immune response by the host, including: CAMP-sensitive element binding protein 1(*Creb1*), Mitogen-activated protein kinase kinase 1 (*Map3k1*), GTPase activating protein with IQ motif 1(*Iqgap1*), BCL2 apoptosis regulator (*Bcl2*), SMAD family member 5 (*Smad5*), Nuclear factor of activated T cells 1 (*Nfatc1*), Glycogen synthase kinase 3 beta (*Gsk3b*), Nuclear factor of activated T cells 5 (*Nfat5*) and Insulin-like growth factor receptor 1 (*Igf1r*). The specific binding sites of miRNAs to the 3'UTR region of the 9 potential target genes are listed in Table 1.

**Table 1:**
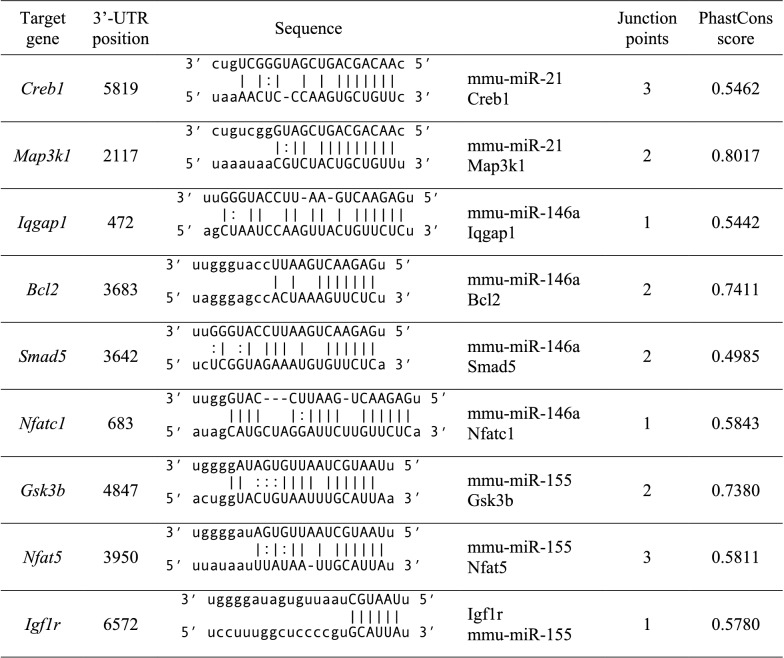
Putative binding sites of miRNAs in the predicted target genes in *Mus musculus*

miR, miRNA, UTR, Untranslated region, CAMP Responsive Element Binding Protein 1(*Creb1*), Mitogen-Activated Protein Kinase Kinase Kinase 1 (*Map3k1*), IQ Motif Containing GTPase Activating Protein 1(*Iqgap1*), BCL2 Apoptosis Regulator (*Bcl2*), SMAD Family Member 5 (*Smad5*), Nuclear factor of activated T cells 1 (*Nfatc1*), Glicogen Synthase Kinase 3 beta (*Gsk3b*), Nuclear factor of activated T cells 5 (*Nfat5*) and Insulin like grown factor 1 receptor (*Igf1r*)

### Target gene expression

**Fig. 7:**
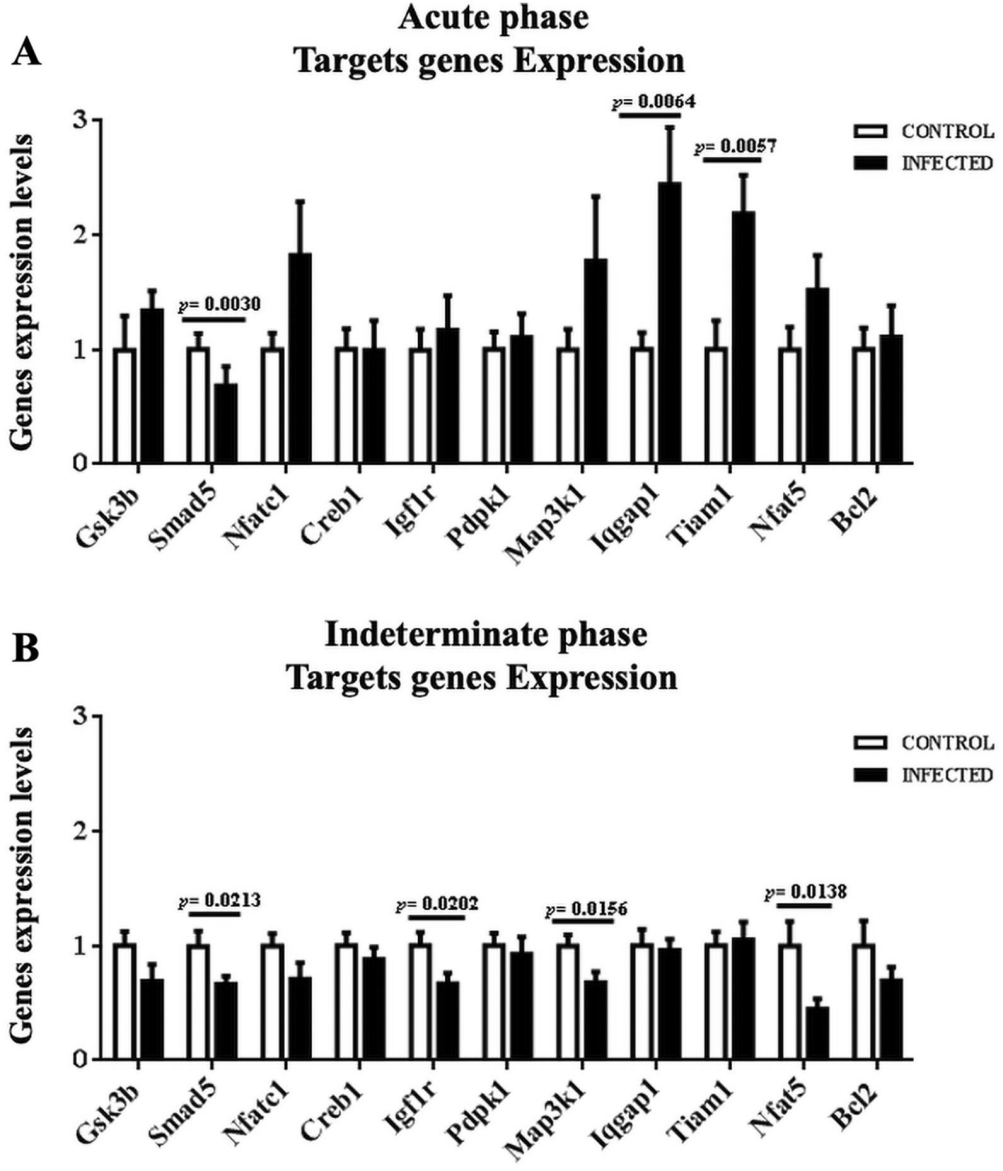
Target genes expressions of *Gsk3b*, *Creb1*, *Igf1r*, *Pdpk1*, *Map3k1*, *Iqgap1*, *Tiam1*, *Bcl2*, *Smad5*, *Nfatc1*, and *Nfat5* genes of heart tissue during the acute and indeterminate phases. Target genes expressions were normalized to Rplp0

Potential target genes of miR-21, miR-146a and miR-155, such as *Gsk3b*,* Creb1*,* Igf1r*,* Pdpk1*,* Map3k1*, *Iqgap1*,* Tiam1*,* Bcl2*,* Smad5*,* Nfatc1 and Nfat5* were proved by RT-qPCR method in mice during the acute (Fig. 7A) and indeterminate phase (Fig. 7B) of the disease. Three genes stand out in the acute phase, Smad5 expression (0.52 ± 0.04 R.U.) was lower than that of its controls (normalized to 1), while Iqgap1 (2.44 ± 0.50 R.U.) and Tiam1 (2.19 ± 0.34 R.U.) were higher than the control. While, Smad5 (0.66 ± 0.07 R.U.), Igf1r (0.67 ± 0.09 R.U.), Map3k1 (0.68 ± 0.09 R.U.) and Nfat5 (0.44 ± 0.09 R.U.) were under-expressed in the indeterminate phase.

## Discussion

Chagas disease is the most severe and potentially fatal cardiomyopathy in humans caused by the *T. cruzi* parasite [27]. The acute phase and the symptomatic chronic phase, also known as CCC, are perceptible by each of the signs and symptoms manifested by the host. Whereas the indeterminate phase manages going unnoticed [28, 29].

In Mexico there is a great variety of *T. cruzi* strains. One of them is the aggressive TcI Ninoa strain, which has tropism for heart tissues with intense focal inflammation. Despite the low inoculation received, histological analysis confirmed the tissue presence of *T. cruzi* Ninoa verified, with foci of inflammation associated with scarce infiltration of mature lymphocytes and intracellular muscle lesions. Also, histological sections showed myolysis spaces filled with amastigotes nests. Previously, histopathological evaluation of the Ninoa strain was tested and similar results were obtained [9, 30].

MicroRNAs play a pivotal role in the regulation of cardiac pathological processes, hypertrophy, atherosclerosis, and inflammatory processes, which are reported as potential tools for monitoring and early detection of multiple diseases [17, 25, 31–35]. Expressions of miRNAs in cardiac tissue from the acute phase, indeterminate asymptomatic phase, and CCC have been explored using murine models and human subjects with Chagas disease. Our selection of miRNAs: miR-21, miR-155 and miR-146a were made based on the literature review of dilated cardiomyopathy, inflammatory processes, fibrosis, myocarditis, hypertrophy, and heart failure, which are alterations that characterize CCC [18–20, 26, 36–38].

Different *T. cruzi* strains have diverse outcomes, TcI Ninoa is predominant in Mexico and there were no previous studies of the role of miRNAs in the pathology associated with this strain. We analyzed the expression profiles of the miRNAs mmu-miR-21, mmu-miR-146a and mmu-miR-155 in cardiac tissue of *Mus musculus* during the acute and indeterminate phase of Chagas disease. Also, the expression of the same miRNAs in blood plasma EVs was analyzed, suggesting a potential use of these miRNAs as biomarkers for the monitoring, diagnosis or early detection of Chagas disease.

Expression profiles of miRNAs showed that the levels of mmu-miR-21, mmu-miR-146a and mmu-miR-155 were significantly elevated during the acute and indeterminate phase of the disease, suggesting an important involvement of these miRNAs in the regulatory processes of Chagas disease. These data are consistent with Navarro et al. [19], who found high levels of miR-21 expression during the acute phase of the disease. On the other hand, the results contrast with the study by Nonaka et al. [20], who evaluated miR-21 expression in CCC, indeterminate phase and control group. They found overexpression in serum and increased expression in the heart of CCC. However, they did not observe overexpression in serum in the indeterminate phase of the disease [19, 20]. Besides, their results in human cells suggest that miR-21 is involved in cardiac fibrosis and hypertrophy in response to *T. cruzi* infection.

Our results showed a high level of miR-21 and miR-146a in EVs, which are responsible for intercellular information transfer and delivery. Halkein et al. suggested that miR-146a secreted in endothelial cell exosomes alters cardiomyocyte metabolism and promotes cellular communication between endothelial cells and cardiomyocytes [39]. We did not separate parasite and host EVs from total EVs. However, there is extensive knowledge about EVs giving and receiving genetic material between parasite and host cells [11, 13, 15], but there is no evidence that miRNAs are present in *T. cruzi* [12, 17, 40]. Therefore, we think that the expression of miRNAs in EVs may originate from the host. Thus, it is conceivable that miR146a could serve as a possible potential and noninvasive tool for early detection of Chagas disease, as overexpression was found in plasma, plasma EVs, and heart tissue of both stages.

Results of data analysis of Mouse Genome 430A 2.0 microarrays normalized by the RMA method (Additional file 3: Figure S1) showed a total of 3465 differentially expressed genes, of which 2839 genes were found to be down-regulated (Additional file 4: figure S2). Combining these genes with the search for target genes for each miRNA allowed the precise identification of genes directly associated with Chagas disease and which were confirmed targets for each miRNA. However, a major obstacle of this approach is the high number of false positives. Therefore, RT-qPCR is generally performed to validate both miRNAs and significant genes identified on platforms such as microarrays. This experimental approach aims to reveal new potential associations between the expression of miRNAs and their respective target genes.

The inflammatory process of cardiac injury in CCC is mainly mediated by CD4+ T cells as they play a central role in controlling specific effectors functions in infection [41]. Studies in humans and mice have shown that the inflammatory reaction observed in CCC is similar to delayed-type hypersensitivity (DTH) composed of mononuclear cells, mainly CD4+ Th1 cells. The lack of association between tissue parasitism and inflammation suggests that the pathogenic response in CCC is not only directed against *T. cruzi* antigens, but also against self T-cell antigens. However, responses to parasite infection may also induce injury and contribute to the development of CCC [42].

During the acute phase of Chagas disease, destruction of intracardiac parasympathetic fibers occurs, leading to the complications seen during the chronic phase of infection. Moreover, the DTH aspect of inflammatory reactions in CCC suggests that Th1 cells may participate in the pathogenesis of this disease; there is an association between the intensity of CCC and the production of high levels of IFN-γ in humans during CCC [43].

MiR-146a plays a role in T cells subpopulations. It is differentially expressed in Th1 and Th2 cells. Mice deficient in miR-146 show an increase in the percentage of INF-γ-producing T cells, whereas it has been observed that miR-146 expression levels in CD4+ T cells are relatively low and it is abundantly expressed in human memory T cells. Moreover, this miRNA is induced upon T cell receptors (TCR) stimulation, which is consistent with its expression being dependent on NF-kB induction. This mouse model suggests that it may be involved in the fate of these cells [44].

Macrophages, monocytes, natural killer (NK) and granulocytes are the first line of defense of the immune system against any pathogen. The inflammatory response induced by macrophages towards an infection, such as *T. cruzi*, involves the positive regulation of several miRNAs such as: miR-155, miR-146a, miR-147 and miR-21 [45]. In our model, we propose that up-regulation of miR-146a could inhibit the function of IL-1 receptor-associated kinase 1 (*Irak1*), whereas miR-21 could regulate the expression of genes regulated by TNF receptor-associated factor 6 (*Traf6*), which are key downstream genes of cytokine and Toll-like receptors (TLRs), indirectly inhibiting the regulation of proinflammatory cytokines characteristic of CCC [46].

On the other hand, transforming growth factor (TGF) is a cytokine involved in various biological and physiological processes; it has been highlighted as a fibrotic regulator in the pathogenesis of Chagas disease [47]. The signaling pathway occurs through three receptors (TGF I, TGF II, TGF III). Binding of active TGF to TGF II activates phosphorylation of proteins of the classical signaling pathway and SMAD proteins [1–7]. TGF plays an important role in the biology of *T. cruzi*; this cytokine is involved in the invasion process of host cells. *T. cruzi* requires functional TGF receptors and its classical signaling pathway to invade the host cell. The amastigote forms capture TGF from the host cell, utilizing it in order to differentiate into its trypomastigote form, allowing it to complete the intracellular cycle of the parasite. The parasite is also able to induce TGF synthesis in cardiomyocytes and cardiac fibroblasts, which influences parasite survival and induces a fibrotic process. [48]. Fan et al. [49] suggest that overexpression of miR-146 could attenuate extracellular matrix (ECM) secretion as well as a fibrotic process, through negative regulation of SMAD family proteins. Also, these results suggest that miR-146a overexpression could be due to a response to parasitic infection by regulating proinflammatory processes through targets such as TGF-βI receptor, TGF-βII and SMAD family member proteins, thus miR-146a could be a potential tool for the detection of *T. cruzi*-induced skeletal muscle fibrosis [49].

The target genes identified for each miRNA were subjected to STRING-KEGG signaling pathway analysis. A total of 23 KEGG pathways associated with inflammatory processes, cytokines, apoptosis, and Chagas disease were identified [27]. Bioinformatic sequence analyses and literature searches in the present study identified several potential target genes for each miRNA, associated with Chagas disease, inflammation, and chemokines: *Gsk3b*,* Creb1*,* Igf1r*,* Pdpk1*,* Map3k1*,* Iqgap1*,* Tiam1*,* Bcl2*,* Smad5*,* Nfatc1 and Nfat5*, which were proved by RT-qPCR.

*Iqgap1 and Tiam1* genes were found to be overexpressed, while *Smad5* was under expressed during the acute phase of the disease. *Iqgap1* has been associated with the immune response as it is involved in cytokine transcription in lymphocytes and regulates immune cell polarization through actin and microtubule activation via MAPK and calcium calmodulin cascades [50]. It is also known that Y (TcII) and Colombian (TcI) EVs can induce and activate *MAPKs* through proteins such as ERK1/2, JNK and p38 [13]. *Tiam1* is a gene encoding a *RAC1-*specific guanine nucleotide exchange *factor *(*GEF*) that exchanges a guanosine diphosphate (*GDP*) for guanosine triphosphate (*GTP*); *GTP* binding induces a conformational change of *RAC1* that allows downstream effectors to transduce a signal [51]. Thus, this gene regulates *RAC1* signaling pathways that affect cell shape, migration, adhesion, growth, survival, and polarity, in addition to influencing actin cytoskeleton formation, endocytosis, and membrane trafficking [52]. This information may explain why it is overexpressed during the acute phase, promoting parasite invasion and transforming immune cells.

*Smad5*,* Igf1r*,* Map3k1 and Nfat5* genes were observed to be under-expressed during the indeterminate phase. The SMAD gene family encodes nucleocytoplasmic mobile proteins responsible for the regulation of embryogenesis processes, including *Smad5*, which serves as a mediator in the TGF signaling pathway. In addition, other biological functions that may be altered during stages of *T. cruzi* infection include cell growth, apoptosis, and cell differentiation [53]. *Igf1r* is a gene associated with growth hormone axis signaling. This activation has effects on carbohydrate and lipid metabolism, in addition to controlling growth and metabolism; the under expression observed in our results can be explained in part by the aforementioned functions of this gene, which perhaps regulate physiological cell turnover and promote the increased fibrosis produced in the dilated cardiomyopathy characteristic of Chagas disease [54]. *Map3k1* belongs to the MAPK family of genes involved in the transmission of information from the cell surface to the cytosol and nucleus. These signal transduction pathways activate MAPK pathways to inhibit the immune response, which are modulated by cytokines such as TNF-α, IL-1, IL-10 and IL-12 [55, 56]. Finally, *Nfat5* belongs to the NFAT gene family, responsible for inducing an independent response to activate transcription factors involved in IFN- production in T cells in response to infection by pathogens such as *T. cruzi* [57].

## Conclusion

Infection by the Ninoa *T. cruzi* strain could have the ability to induce an inflammatory response due to the overexpression of miR-21 and miR-146a in cardiac tissue, plasma, and plasma EVs in mouse blood. These miRNAs maintain a high level of expression that remains constant in the two phases that were studied. MiR-146a could be considered as a possible potential non-invasive biomarker for early detection of Chagas disease. Our results should be taken into consideration with caution and further studies with a panel of DTUs/strains to validate these observations are needed.

## Methods

### Parasites

We used Ninoa (MHOM/MX/1986/Ninoa) [58]. *T. cruzi* (TcI) strain, which was obtained from an acute case (infant) of Chagas disease derived from the State of Oaxaca in southern Mexico. This strain was maintained in triatomine insects by serial passaging in CD1 mice. Metacyclic trypomastigotes were obtained from Triatoma (*Meccus pallidipenis*) urine and feces.

### Experimental infections

Six mice were inoculated intraperitoneally with 1000 metacyclic trypomastigotes to measure parasitemia. An infection curve was established every three days, starting on the 7th day postinoculation, and 6 μL of blood was obtained from the tail of the mice. The blood was screened and the number of motile parasites was counted according to the modified Pizzi–Brener method [59].

In addition, forty pathogen-free female CD1 mice (6–8 weeks), weighing 20-25 g, were divided into 4 groups of 10 mice and maintained on the 12/12 h circadian cycle, at room temperature, with water and food ad libitum. Two groups of ten mice, which were considered as cases, were inoculated intraperitoneally with 1000 metacyclic trypomastigote parasites of Ninoa strain. Two groups of ten mice considered as controls, received urine/feces vector without trypomastigotes by the same route. All animal procedures were performed in accordance with the "Code of Ethics of the World Medical Association" (Declaration of Helsinki): EC Directive 86/609/EEC for animal experimentation.

### Sampling

The first infected group and their controls were sacrificed 21 days after infection (acute phase). The second group and their controls were sacrificed 81 days post-infection (indeterminate phase). In both cases, the mice were euthanized by an overdose of anesthesia with sodium pentobarbital (100–150 mg/kg IP). Blood was obtained by cardiac puncture and the hearts were dissected from the animals.

### Histological evaluation of *T. cruzi*-infected mice

After the infection procedure (21 and 81 days), the apex of the heart was sectioned for miRNA experiments. The remaining of six hearts from infected mice and six hearts from uninfected mice were fixed in buffered formalin solution (10%), embedded in kerosene, cut into 5 μm sections, deparaffinized and stained with hematoxylin–eosin. Finally, these sections were examined at 40X magnification to assess parasite load and inflammation.

### Isolation and enrichment of miRNAs

Total RNA was extracted and subsequently enriched from mouse blood plasma and heart tissue using the commercial Qiagen miRNeasy® Serum / Plasma Kit (ID: 217184) and Qiagen miRNeasy® Kit (ID: 217004) according to the manufacturer’s specifications and stored at − 70 °C until use. Plasma and cardiac tissue samples were homogenized with QIAzol® Lysis Reagent (Qiagen) following the manufacturer’s protocol.

### Identification of miRNAs by RT-qPCR

The selection of miRNAs: miR-21, miR-155 and miR-146a were based on myocarditis, fibrosis, hypertrophy, and cardiomyopathy, which are alterations that characterize Chagas disease. Likewise, this proposal was based on an exhaustive and extensive bibliographic search in the public database PubMed (https://pubmed.ncbi.nlm.nih.gov/) on the function of these miRNAs and their association with *T. cruzi*, Chagas disease and cardiomyopathy.

To obtain cDNA, 1 µL of total RNA was used for each sample type. The plasma volume was used directly, while in the heart samples a dilution of total RNA was adjusted to a concentration of 25 ng/μL. One microliter was then added to a final mix solution volume of 12 μL made by primers specific for: miR-21 (assay ID 000397), miR-146a (assay ID 000468) and miR-155 (assay ID 00257); as well as primers specific for cel-miR-39 (assay ID 000200) and U6 (assay ID 001973) as reference. Reverse transcription reactions were performed with TaqMan® microRNA (Applied Biosystems, Foster City CA, USA) according to the manufacturer’s recommendations (Additional file 5: Table S3).

Relative concentrations of plasma miRNAs were normalized to the Ct values of Cel-miR-39 and values were calculated using the relative expression equation 2^−ΔΔCt^, where ΔΔCt = (Ct miRNA − Ct internal control) experiment −  (Ct miRNA − Ct internal control) control. All Ct values for cel-miR-39 ranged from 18 to 20 cycles for both total plasma and EVs RNA isolates. We used U6 to normalize heart samples.

### Estimation of the number of particles

The description of the particles was performed as described by Brianza-Padilla [60]. Within the plasma EVs, parasite and host EVs were included and referred to as total EVs. One mL of blood plasma from each group was added to 400 µL of PBS and ultracentrifuged (Optima MAX Ultracentrifuge; Beckman Coulter) at 120,000×*g* at 4 °C for 90 min. The pellets were washed with PBS by another centrifugation at 12,000×*g* for 90 min. The final pellet was again resuspended in 50 µL PBS for nanoparticle tracking analysis (NTA). Vesicle size and concentration were determined with the NanoSight NS300 (Malvern Instruments Ltd). Three replicates of 1:200 dilutions were injected into the equipment chamber. The gain of the chamber is at a value of 10 and for vesicle detection the threshold value is at 5.

### Isolation of miRNAs from plasma EVs

For this assay, 200 μL of plasma samples from three or four mice were pooled and processed for RNA isolation using the Qiagen exoRNeasy® serum/plasma midi kit. During the RNA purification step, 3.5 μL of cell-miR-39 Spike-In Control (1.6 × 108 copies/μL) was added according to the supplier’s recommendations. In this assay, 1 μL of RNA isolated from EV plasma was transcribed to cDNA as described above. The relative concentrations of miRNAs from EVs were normalized to the Ct values of Cel-miR-39 and the values were calculated using the relative expression equation 2^−ΔΔCt^ as above.

### Bioinformatics analysis

#### Prediction of potential target genes of miRNAs

To predict the potential target genes of miR-21, miR-146a and miR-155, algorithms based on different computational methods for the identification of target genes were used. This selection is based on the conservation of miRNA binding sites and the 3ʹUTR region of the mRNA (TargetScan); others are based on the accessibility of the target gene site and on thermodynamic properties that allow filtering of binding sites in the seed region (miRanda). In the last decade some databases have used machine learning based on the parameterization of biological data and other predicted features, as reported by Sticht et al. [62]. The databases used were: microRNA.org (http://www.microrna.org/microrna/home.do), miRDB (http://mirdb.org/miRDB/), miRWalk (http://zmf.umm.uniheidelberg.de/apps/zmf/mirwalk2/), TargetScan (http://www.targetscan.org/vert_72/) and PITA v5 0.0 (https://omictools.com/pita-tool). The predicted genes were selected if ≥ 3 databases were present for further analysis [61–63].

### Expression microarray analysis

To select those genes associated with *T. cruzi* infection, we analyzed data in CEL format from the Mouse Genome 430A 2.0 microarray of the Affymetrix platform, these data came from the Gene Expression Omnibus (https://www.ncbi.nlm.nih.gov/geo/) with access number GSE41089. The authors analyzed the expression profiles of mRNAs in the cardiac tissue of wild-type mice infected with *T. cruzi* versus a control group [62].

### Data processing and screening of DEGs

To perform differential expression analysis, the original files were obtained in CEL format (GSE41089) and processed into expression values using the Robust multi-array average (RMA) method in the "R" environment via Affy packages. Subsequently, probe-level data were transformed using R/Bioconductor, followed by background correction and normalization of the data by the quantile method. The cutoff criteria for choosing overexpressed genes were that they had a fold change value < − 0.5 and a false discovery rate (FDR) value < 0.05.

### Selection of potential candidate genes

Candidate genes were selected by a comparative analysis (Venn diagram) between the predicted target genes for each miRNA and the differentially expressed genes from microarray analysis. The set of shared genes was analyzed using the STRING-KEGG Pathway database (https://string-db.org/) of *Mus musculus* to obtain and select those signaling pathways involved in *T. cruzi* infection. This selection was used to develop an interaction network of genes involved in these pathways in Cytoscape v3.7.2 software and select genes that were validated by RT-qPCR.

### RT-qPCR of mRNA genes expression in heart tissue

RT-qPCR assay was performed using primers for each target gene, which were designed with ProbeFinder version 2.5 software for Mouse Universal ProbeLibrary (Integrated DNA Technologies [IDT: www.idtdna.com]). For each target mRNA gene, 1 μg of total RNA from cardiac tissue was reverse transcribed with the Transcripts First Strand cDNA Synthesis Kit (Roche Diagnostics, Mannheim, Germany) and Light Cycler 480 Probe Master Kit (Roche Applied Science, Mannheim, Germany). Cycling conditions were 45 cycles of 95 °C for 10 s, 1 cycle of 60 °C for 30 s and 1 cycle of 72 °C for 1 s. Quantification of relative mRNA levels was calculated from Ct values and normalized to Rplp0 in each sample. The relative expression ratio was calculated by the 2^−ΔΔCt^ method.

### Statistical analysis

Statistical differences between groups were assessed using the unpaired Student's t-test or the Mann–Whitney *U* test (*p* < 0.05) and were performed with Graph Pad Prism software version 6.0. Data were presented as means and standard errors. The data were tested for normality and equality of variance.

## Supplementary Information

**Additional file 1: Table S1.** Parasitemia curve from Ninoa *T. cruzi* strain by Pizzi–Brener method.

**Additional file 2: Table S2.** Matrix expression of the Mouse Genome 430A 2.0 microarray of the Affymetrix.

**Additional file 3: Figure S1.** Intensity histogram and box plot of gene expression in different samples before and after normalization. (A) Gene expression in histogram and box plot of heart tissue samples from control (TC) and *T. cruzi* infected (TI) mice before normalization. (B) Gene expression in histogram and box plot of heart tissue samples from control mice and *T. cruzi*-infected mice after normalization.

**Additional file 4: Figure S2.** Volcano plot of the differentially expressed genes that were evaluated by microarray analysis in cardiac tissue from healthy mice and *T. cruzi*-infected mice. The y-axis indicates the—log10 of the P—values and the x-axis is the CF (measured as the log2 transformed ratio of expression between both experimental groups).

**Additional file 5: Table S3.** Oligonucleotide Design for RT-qPCR.

## Data Availability

Data supporting the results of this study are available in the Gene Expression Omnibus repository (https://www.ncbi.nlm.nih.gov/geo/) under access number GSE41089.

## Abbreviations

*T. cruzi*: *Trypanosoma cruzi*
Th1/Th17: T helper cells
DTUs: Discrete typing units
EVs: Extracellular vesicles
miRNA: MicroRNAs
UTRs: Untranslated regions
mRNA: Messenger RNA
RT-qPCR: Quantitative reverse transcription PCR
PCR: Polymerase chain reaction
CCC: Chronic chagasic cardiomyopathy
cDNA: Complementary DNA
DNA: Deoxyribonucleic acid
RNA: Ribonucleic acid
Ct: Threshold cycle
STRING: Search Tool for the Retrieval of Interacting Genes/Proteins
KEGG: Kyoto Encyclopedia of Genes and Genomes
ERK: Extracellular signal-regulated kinases
JNK: C-Jun N-terminal kinase
RAC1: Ras-related C3 botulinum toxin substrate 1
TGF-β: Transforming growth factor-beta
TNF-α: Tumor necrosis factor-alpha
IL-: Interleukin
IFN-α: Interferon-alpha
