# Supplementary material for: Circulating miR-146a as a possible candidate biomarker in the indeterminate phase of Chagas disease
Source: Biol Res. 2021 Jul 21;54:21. doi: 10.1186/s40659-021-00345-3 (PMC8293491; doi:10.1186/s40659-021-00345-3)
Supplement: Supplementary file 1 — Additional file 1: Table S1. Parasitemia curve from Ninoa T. cruzi strain by Pizzi–Brener method. [file 40659_2021_345_MOESM1_ESM.docx]

Table S1. Parasitemia curve from Ninoa *T. cruzi* strain by Pizzi–Brener method.

Days post-infection Number of parasites/mL

| M1 | | | M2 | M3 | M4 | M5 | M6 |
| --- | --- | --- | --- | --- | --- | --- | --- |
|  | 0 | 0 | 0 | 0 | 0 | 0 | 0 |
|  | 13 | 13300000 | 0 | 26500000 | 31000000 | 0 | 35400000 |
|  | 15 | 66300000 | 17700000 | 48600000 | 53100000 | 8840000 | 35400000 |
|  | 17 | 168000000 | 48600000 | 119000000 | 97300000 | 8840000 | 155000000 |
|  | 20 | 663000000 | 270000000 | 1240000000 | 535000000 | 619000000 | 1210000000 |
|  | 22 | 1860000000 | 929000000 | 1680000000 | 1410000000 | 186000000 | 2430000000 |
|  | 24 | 4310000000 | 1310000000 | 2600000000 | 2110000000 | 478000000 | 4130000000 |
|  | 27 | 8590000000 | 2940000000 | 0 | 7640000000 | 579000000 | 10500000000 |
|  | 29 | 0 | 5240000000 | 0 | 10400000000 | 535000000 | 10300000000 |
|  | 31 | 0 | 7010000000 | 0 | 0 | 447000000 | 0 |
|  | 34 | 0 | 0 | 0 | 0 | 92900000 | 0 |
|  | 36 | 0 | 0 | 0 | 0 | 48600000 | 0 |
|  | 38 | 0 | 0 | 0 | 0 | 39800000 | 0 |
|  | 41 | 0 | 0 | 0 | 0 | 31000000 | 0 |
|  | 43 | 0 | 0 | 0 | 0 | 22100000 | 0 |
|  | 45 | 0 | 0 | 0 | 0 | 8840000 | 0 |
|  | 47 | 0 | 0 | 0 | 0 | 4420000 | 0 |
|  | 49 | 0 | 0 | 0 | 0 | 0 | 0 |
|  | 51 | 0 | 0 | 0 | 0 | 0 | 0 |
| **M= mouse** |  |  |  |  |  |  |  |
