# Supplementary material for: Circulating miR-146a as a possible candidate biomarker in the indeterminate phase of Chagas disease
Source: Biol Res. 2021 Jul 21;54:21. doi: 10.1186/s40659-021-00345-3 (PMC8293491; doi:10.1186/s40659-021-00345-3)
Supplement: Supplementary file 2 — Additional file 2: Table S2. Matrix expression of the Mouse Genome 430A 2.0 microarray of the Affymetrix. [file 40659_2021_345_MOESM2_ESM.docx]

Table S2. Matrix expression of the Mouse Genome 430A 2.0 microarray of the Affymetrix

| **Gene** | **ID** | **logFC** | **AveExpr** | **t** | **P.Value** | **adj.P.Val** | **B** |
| --- | --- | --- | --- | --- | --- | --- | --- |
| Ccl5 | 1418126_at | -5.5475784 | 10.7606998 | -59.525599 | 4.71E-12 | 1.53E-08 | 17.763992 |
| Gbp6 | 1438676_at | -5.5314474 | 10.8138067 | -46.549082 | 3.48E-11 | 4.93E-08 | 16.2450038 |
| Cxcl9 | 1418652_at | -5.4028676 | 10.6252957 | -61.816173 | 3.46E-12 | 1.53E-08 | 17.97539 |
| Gzmb | 1419060_at | -5.1423545 | 10.2900574 | -40.491739 | 1.08E-10 | 9.07E-08 | 15.285733 |
| Gzma | 1417898_a_ | -4.8233631 | 9.68060986 | -55.752638 | 8.02E-12 | 2.02E-08 | 17.3830567 |
| Igtp | 1417141_at | -4.8120293 | 11.6210202 | -41.381513 | 9.05E-11 | 8.21E-08 | 15.4394405 |
| Irgm2 | 1417793_at | -4.7294498 | 10.8449789 | -66.463257 | 1.92E-12 | 1.45E-08 | 18.3637971 |
| NA | 1449009_at | -4.6951226 | 11.9858066 | -59.730273 | 4.58E-12 | 1.53E-08 | 17.7834624 |
| Serpina3g | 1424923_at | -4.6893305 | 9.99157501 | -31.430565 | 8.42E-10 | 3.03E-07 | 13.3983985 |
| Cd274 | 1419714_at | -4.6145063 | 10.1372853 | -79.197035 | 4.60E-13 | 1.04E-08 | 19.2057328 |
| Ms4a4b | 1423467_at | -4.5605592 | 9.46455094 | -35.947538 | 2.84E-10 | 1.57E-07 | 14.4194867 |
| Gbp2 | 1418240_at | -4.5280604 | 11.2021721 | -67.805158 | 1.63E-12 | 1.45E-08 | 18.4668148 |
| Cxcl10 | 1418930_at | -4.492817 | 9.58745381 | -47.980001 | 2.72E-11 | 4.11E-08 | 16.444567 |
| NA | 1438009_at | -4.4321912 | 9.84231845 | -39.294133 | 1.38E-10 | 1.01E-07 | 15.0710724 |
| BC023105 | 1425394_at | -4.4260154 | 9.91816969 | -57.085806 | 6.62E-12 | 1.88E-08 | 17.5225922 |
| Iigp1 | 1419042_at | -4.3688429 | 10.8952474 | -31.71136 | 7.83E-10 | 2.87E-07 | 13.4673056 |
| Iigp1 | 1419043_a_ | -4.3490091 | 11.9715602 | -35.910087 | 2.86E-10 | 1.57E-07 | 14.4117295 |
| Ifit3 | 1449025_at | -4.3066427 | 10.9433523 | -37.259121 | 2.12E-10 | 1.34E-07 | 14.6844312 |
| Irgm1 | 1418825_at | -4.3050002 | 11.0647864 | -63.958739 | 2.62E-12 | 1.49E-08 | 18.1608502 |
| Psmb8 | 1422962_a_ | -4.2947519 | 10.9740182 | -49.684055 | 2.05E-11 | 3.32E-08 | 16.670447 |
| Gbp2 | 1435906_x_a-4.253309 | | 11.6780283 | -52.203693 | 1.37E-11 | 3.11E-08 | 16.9827424 |
| Ifi47 | 1417292_at -4.2513587 | | 10.7587973 | -43.641089 | 5.88E-11 | 6.42E-08 | 15.8091033 |
| NA | 1449556_at -4.1206258 | | 11.8334163 | -40.589996 | 1.06E-10 | 9.07E-08 | 15.3029437 |
| Ccl8 | 1419684_at -4.0816338 | | 10.520463 | -33.700958 | 4.79E-10 | 2.02E-07 | 13.9340629 |
| Ifit2 | 1418293_at -4.0397911 | | 10.2015872 | -33.610705 | 4.89E-10 | 2.02E-07 | 13.913672 |
| Ptprc | 1422124_a_ -3.9787297 | | 10.6125481 | -18.058874 | 7.27E-08 | 6.87E-06 | 8.86674704 |
| Gbp3 | 1418392_a_ -3.976879 | | 10.4677996 | -44.274144 | 5.23E-11 | 6.24E-08 | 15.9076502 |
| Saa3 | 1450826_a_ -3.9612233 | | 10.3967935 | -20.006194 | 3.21E-08 | 3.91E-06 | 9.72890178 |
| Stat1 | 1450033_a_ -3.9462817 | | 11.1015011 | -41.4313 | 8.96E-11 | 8.21E-08 | 15.4479006 |
| Nkg7 | 1450753_at -3.9390368 | | 9.59484592 | -30.945759 | 9.55E-10 | 3.23E-07 | 13.2775652 |
| Ctss | 1448591_at -3.8963166 | | 10.9665526 | -22.300738 | 1.34E-08 | 2.19E-06 | 10.6344797 |
| Gbp7 | 1434380_at -3.8777999 | | 10.1553474 | -50.29522 | 1.85E-11 | 3.32E-08 | 16.7485 |
| Ifit1 | 1450783_at -3.8522408 | | 10.6829233 | -33.027119 | 5.64E-10 | 2.24E-07 | 13.7800716 |
| Psmb9 | 1450696_at -3.841669 | | 10.1903003 | -29.231275 | 1.51E-09 | 4.58E-07 | 12.8303556 |
| Gbp8 | 1418776_at -3.8409564 | | 8.53416744 | -24.282859 | 6.76E-09 | 1.35E-06 | 11.336323 |
| H2-Ab1 | 1451721_a_ -3.8169218 | | 10.1944588 | -17.640971 | 8.76E-08 | 7.83E-06 | 8.66882555 |
| Plac8 | 1451335_at -3.8068029 | | 10.2258348 | -21.348376 | 1.91E-08 | 2.74E-06 | 10.2717381 |
| H2-Aa | 1452431_s_a-3.7971454 | | 10.7129153 | -13.909493 | 5.75E-07 | 2.83E-05 | 6.65204445 |
| Cd52 | 1460218_at -3.7678639 | | 10.6430591 | -16.985706 | 1.18E-07 | 9.43E-06 | 8.34836934 |
| NA | 1418536_at -3.7598724 | | 10.6548275 | -25.227924 | 4.97E-09 | 1.13E-06 | 11.6481218 |
| Mpeg1 | 1427076_at -3.7574828 | | 10.0366899 | -18.139519 | 7.02E-08 | 6.66E-06 | 8.90438475 |
| NA | 1416016_at -3.7273575 | | 10.2105577 | -31.031071 | 9.34E-10 | 3.23E-07 | 13.2990017 |
| Gbp7 | 1425156_at -3.6932561 | | 10.7156303 | -50.552715 | 1.78E-11 | 3.32E-08 | 16.7809337 |
| Itgb2 | 1450678_at -3.6133102 | | 10.4139634 | -19.725446 | 3.59E-08 | 4.20E-06 | 9.6103186 |
| Lgals3 | 1426808_at -3.5727822 | | 10.2673089 | -21.896478 | 1.55E-08 | 2.35E-06 | 10.4826579 |
| NA | 1449875_s_a-3.5721313 | | 10.5918354 | -33.327458 | 5.24E-10 | 2.12E-07 | 13.8492093 |
| Fcgr4 | 1425225_at -3.5313539 | | 9.59424374 | -22.02337 | 1.48E-08 | 2.28E-06 | 10.5306489 |
| Coro1a | 1416246_a_ -3.5193199 | | 10.3002624 | -15.971649 | 1.93E-07 | 1.36E-05 | 7.82627048 |
| Il2rg | 1416295_a_ -3.4834383 | | 9.74095296 | -32.087179 | 7.12E-10 | 2.69E-07 | 13.5583211 |
| Stat1 | 1450034_at -3.4825794 | | 11.2664173 | -45.718417 | 4.03E-11 | 5.08E-08 | 16.1247748 |
| NA | 1431591_s_a-3.4519362 | | 9.34013289 | -26.077723 | 3.81E-09 | 9.19E-07 | 11.9170353 |
| H2-Eb1 | 1417025_at -3.4370075 | | 11.6375815 | -17.05842 | 1.14E-07 | 9.27E-06 | 8.3845603 |
| Parp14 | 1451564_at -3.4367857 | | 10.4852126 | -35.730827 | 2.98E-10 | 1.57E-07 | 14.3744477 |
| H2-Ab1 | 1425477_x_a-3.4268709 | | 10.1751635 | -17.622029 | 8.84E-08 | 7.86E-06 | 8.65973824 |
| Gbp2b | 1420549_at -3.4266225 | | 9.06322483 | -11.484648 | 2.56E-06 | 8.20E-05 | 5.03083283 |
| Ms4a6b | 1418826_at -3.4044993 | | 10.0038511 | -39.645377 | 1.28E-10 | 9.69E-08 | 15.1349804 |
| Lck | 1425396_a_ -3.3723757 | | 9.10743152 | -23.118315 | 1.00E-08 | 1.75E-06 | 10.9322632 |
| Ubd | 1419762_at -3.3621027 | | 9.2467231 | -35.395539 | 3.22E-10 | 1.62E-07 | 14.3040359 |
| Samhd1 | 1418131_at -3.3513859 | | 10.3627679 | -40.021308 | 1.19E-10 | 9.62E-08 | 15.2024995 |
| H2-Ab1 | 1450648_s_a-3.3496236 | | 11.5643422 | -17.173445 | 1.09E-07 | 9.08E-06 | 8.44148378 |

| H2-DMa | 1422527_at -3.3479344 | 9.51932748 | -15.860563 | 2.04E-07 | 1.40E-05 | 7.76701891 |
| --- | --- | --- | --- | --- | --- | --- |
| Coro1a | 1455269_a_ -3.345985 | 9.50544346 | -21.421231 | 1.85E-08 | 2.70E-06 | 10.300119 |
| Laptm5 | 1436905_x_a-3.3293884 | 10.6565228 | -24.656601 | 5.98E-09 | 1.25E-06 | 11.4612887 |
| Lcp1 | 1415983_at -3.3173434 | 10.5790253 | -20.668597 | 2.47E-08 | 3.22E-06 | 10.0016466 |
| Psmb10 | 1448632_at -3.298238 | 11.9987981 | -46.145302 | 3.73E-11 | 4.98E-08 | 16.1869739 |
| Irf7 | 1417244_a_ -3.2825099 | 10.4764601 | -39.80437 | 1.24E-10 | 9.69E-08 | 15.1636466 |
| Ifi30 | 1422476_at -3.2798635 | 10.2495787 | -18.031751 | 7.36E-08 | 6.93E-06 | 8.85404872 |
| Ch25h | 1449227_at -3.2582378 | 9.03106238 | -34.103234 | 4.35E-10 | 1.93E-07 | 14.0240858 |
| Cotl1 | 1436838_x_a-3.2409911 | 10.0489321 | -18.001083 | 7.46E-08 | 6.96E-06 | 8.83966657 |
| NA | 1431008_at -3.2287465 | 11.0730092 | -18.511719 | 5.97E-08 | 6.07E-06 | 9.07582472 |
| Ccr5 | 1424727_at -3.2219836 | 9.44208436 | -29.029877 | 1.60E-09 | 4.72E-07 | 12.7756849 |
| Ptx3 | 1418666_at -3.221757 | 8.8079274 | -15.849107 | 2.05E-07 | 1.40E-05 | 7.7608845 |
| Cd3g | 1419178_at -3.2118836 | 9.01458726 | -16.413209 | 1.56E-07 | 1.15E-05 | 8.05769174 |
| H2-Aa | 1435290_x_a-3.1967778 | 12.2971482 | -13.792538 | 6.14E-07 | 2.94E-05 | 6.58034903 |
| Limd2 | 1456377_x_a-3.1911487 | 10.1883848 | -13.921632 | 5.71E-07 | 2.82E-05 | 6.65945215 |
| Zbp1 | 1429947_a_ -3.1887354 | 9.64910559 | -49.819291 | 2.00E-11 | 3.32E-08 | 16.6878495 |
| NA | 1425854_x_a-3.1877982 | 9.32115657 | -27.761085 | 2.30E-09 | 6.21E-07 | 12.4202136 |
| Il2rg | 1416296_at -3.1357697 | 10.2296829 | -21.138896 | 2.06E-08 | 2.82E-06 | 10.1895316 |
| Cd53 | 1448617_at -3.1329864 | 9.5263422 | -18.276696 | 6.61E-08 | 6.46E-06 | 8.96800081 |
| H2-K1 | 1427746_x_a-3.1301706 | 11.9252984 | -22.068799 | 1.46E-08 | 2.27E-06 | 10.547755 |
| AW112010 | 1434372_at -3.1127675 | 11.6285821 | -20.854737 | 2.30E-08 | 3.07E-06 | 10.0765681 |
| Oasl2 | 1453196_a_ -3.1065697 | 10.0273757 | -43.082914 | 6.52E-11 | 6.73E-08 | 15.7204373 |
| Rac2 | 1417620_at -3.0966616 | 10.1403687 | -18.950689 | 4.95E-08 | 5.22E-06 | 9.27336482 |
| Irf8 | 1416714_at -3.0912708 | 9.20285075 | -19.212792 | 4.43E-08 | 4.81E-06 | 9.38899222 |
| Stat1 | 1420915_at -3.0890061 | 9.04019811 | -35.118164 | 3.43E-10 | 1.65E-07 | 14.245107 |
| Cd74 | 1425519_a_ -3.0614105 | 12.328029 | -15.193305 | 2.87E-07 | 1.73E-05 | 7.401991 |
| Trav9d-3 | 1426168_a_ -3.0587395 | 8.93048291 | -19.646177 | 3.71E-08 | 4.32E-06 | 9.57650499 |
| Hck | 1449455_at -3.0425166 | 9.4008581 | -19.086066 | 4.67E-08 | 5.04E-06 | 9.33329973 |
| NA | 1420394_s_a-3.0408291 | 9.79069254 | -23.184753 | 9.82E-09 | 1.74E-06 | 10.9559356 |
| Aif1 | 1418204_s_a-3.0353847 | 9.32826955 | -14.499644 | 4.15E-07 | 2.27E-05 | 7.00498427 |
| Acod1 | 1427381_at -3.0278943 | 8.65516162 | -26.921762 | 2.94E-09 | 7.59E-07 | 12.1740424 |
| Epsti1 | 1452087_at -3.0015755 | 9.16272424 | -14.228605 | 4.81E-07 | 2.49E-05 | 6.8446887 |
| Ccl2 | 1420380_at -3.0014429 | 9.26056392 | -14.211156 | 4.86E-07 | 2.50E-05 | 6.83426564 |
| NA | 1449580_s_a-2.9972591 | 9.96897006 | -15.679409 | 2.23E-07 | 1.47E-05 | 7.66948159 |
| Rsad2 | 1421009_at -2.9943653 | 10.2602596 | -21.90505 | 1.55E-08 | 2.35E-06 | 10.4859098 |
| Ly86 | 1422903_at -2.9921856 | 10.2526955 | -13.371215 | 7.84E-07 | 3.51E-05 | 6.31704232 |
| NA | 1419004_s_a-2.9855658 | 8.26891466 | -22.164532 | 1.41E-08 | 2.24E-06 | 10.5836742 |
| Timp1 | 1460227_at -2.9838514 | 9.77969487 | -37.065072 | 2.21E-10 | 1.36E-07 | 14.6460404 |
| Ifng | 1425947_at -2.960262 | 8.1031856 | -29.605018 | 1.37E-09 | 4.25E-07 | 12.9305865 |
| Lcp1 | 1448160_at -2.9580141 | 9.58988077 | -17.227498 | 1.06E-07 | 9.03E-06 | 8.46809655 |
| Tgfbi | 1448123_s_a-2.9504401 | 10.199581 | -22.372867 | 1.31E-08 | 2.15E-06 | 10.661243 |
| NA | 1452205_x_a-2.9445992 | 8.44708749 | -18.433073 | 6.17E-08 | 6.25E-06 | 9.03990608 |
| Rsad2 | 1436058_at -2.9379942 | 11.5982179 | -16.525169 | 1.47E-07 | 1.11E-05 | 8.11535378 |
| Tgfbi | 1456250_x_a-2.9288803 | 10.4736113 | -35.637685 | 3.04E-10 | 1.57E-07 | 14.3549767 |
| Id2 | 1435176_a_ -2.9248366 | 9.61043057 | -17.196617 | 1.07E-07 | 9.08E-06 | 8.45290344 |
| Cd8b1 | 1426170_a_ -2.922206 | 8.46606385 | -23.142265 | 9.96E-09 | 1.75E-06 | 10.9408055 |
| NA | 1456014_s_a-2.9164485 | 9.29938033 | -23.055252 | 1.03E-08 | 1.77E-06 | 10.9097217 |
| Ccl7 | 1421228_at -2.9095622 | 9.06358966 | -23.247166 | 9.61E-09 | 1.73E-06 | 10.9781036 |
| Mki67 | 1426817_at -2.9073218 | 8.33183445 | -28.680568 | 1.77E-09 | 5.02E-07 | 12.6797458 |
| Ctsc | 1416382_at -2.8653133 | 11.3239996 | -27.469879 | 2.50E-09 | 6.68E-07 | 12.3358323 |
| NA | 1425226_x_a-2.8519296 | 9.42816662 | -16.533358 | 1.47E-07 | 1.11E-05 | 8.1195554 |
| Ms4a6d | 1419598_at -2.8375824 | 8.38958499 | -22.708851 | 1.16E-08 | 1.96E-06 | 10.784641 |
| Ms4a4c | 1450291_s_a-2.8241673 | 7.73508856 | -17.057409 | 1.15E-07 | 9.27E-06 | 8.38405866 |
| Thy1 | 1423135_at -2.8235154 | 9.81580908 | -15.919499 | 1.98E-07 | 1.38E-05 | 7.79850698 |
| NA | 1451644_a_ -2.8100397 | 8.54646027 | -15.645999 | 2.27E-07 | 1.48E-05 | 7.65136721 |
| Plek | 1448748_at -2.8036515 | 9.11037599 | -22.595684 | 1.21E-08 | 2.03E-06 | 10.7433091 |
| Fcer1g | 1418340_at -2.7923396 | 10.4595614 | -16.911018 | 1.23E-07 | 9.70E-06 | 8.31102804 |
| Rtp4 | 1418580_at -2.7820473 | 10.6880757 | -25.153094 | 5.09E-09 | 1.14E-06 | 11.6239337 |
| Gimap4 | 1424375_s_a-2.7771884 | 10.9277339 | -23.213714 | 9.72E-09 | 1.74E-06 | 10.9662305 |
| Socs3 | 1455899_x_a-2.7769313 | 9.53835139 | -13.233059 | 8.50E-07 | 3.70E-05 | 6.22893998 |
| NA | 1451683_x_a-2.7597505 | 10.9846931 | -24.712774 | 5.87E-09 | 1.25E-06 | 11.4798808 |
| C1qb | 1437726_x_a-2.7569835 | 10.5837723 | -19.026141 | 4.79E-08 | 5.11E-06 | 9.30682564 |
| Cd48 | 1427301_at -2.7564556 | 8.92303346 | -18.198577 | 6.84E-08 | 6.60E-06 | 8.93183509 |
| Gzmk | 1422280_at -2.7475504 | 8.79000244 | -24.817255 | 5.68E-09 | 1.22E-06 | 11.5143315 |
| Lcp2 | 1418641_at -2.7289125 | 8.76363654 | -15.96999 | 1.93E-07 | 1.36E-05 | 7.82538849 |
| Usp18 | 1418191_at -2.7225244 | 9.51412237 | -24.809771 | 5.69E-09 | 1.22E-06 | 11.5118696 |
| Cd3d | 1422828_at -2.7164475 | 9.329683 | -23.08587 | 1.02E-08 | 1.76E-06 | 10.9206744 |
| C1qb | 1417063_at -2.71595 | 11.470921 | -14.574271 | 3.98E-07 | 2.21E-05 | 7.04859717 |
| NA | 1426324_at -2.6986508 | 10.0036624 | -26.584671 | 3.26E-09 | 8.22E-07 | 12.0725668 |
| Bst2 | 1424921_at -2.698371 | 10.3098067 | -38.741038 | 1.55E-10 | 1.06E-07 | 14.9687925 |
| Irf1 | 1448436_a_ -2.6849346 | 11.1576956 | -26.158674 | 3.71E-09 | 9.16E-07 | 11.9421108 |
| Cytip | 1435697_a_ -2.6704429 | 8.88808197 | -19.448563 | 4.02E-08 | 4.56E-06 | 9.49156286 |
| Ms4a6d | 1419599_s_a-2.6678963 | 8.52921549 | -24.898461 | 5.53E-09 | 1.22E-06 | 11.5409913 |
| Fcgr1 | 1417876_at -2.6591427 | 9.00672864 | -28.15076 | 2.05E-09 | 5.68E-07 | 12.5314637 |
| Capg | 1450355_a_ -2.6511273 | 9.70138014 | -17.614176 | 8.87E-08 | 7.86E-06 | 8.65596736 |
| Fcgr2b | 1435477_s_a-2.6504827 | 9.86663988 | -17.806812 | 8.13E-08 | 7.38E-06 | 8.74795381 |
| Uba7 | 1426971_at -2.6457132 | 9.57752254 | -37.74059 | 1.91E-10 | 1.24E-07 | 14.7785107 |
| Ifi44 | 1423555_a_ -2.6440867 | 8.9213596 | -41.812944 | 8.32E-11 | 8.21E-08 | 15.5122665 |
| Slc15a3 | 1420697_at -2.6367747 | 8.58514671 | -22.122711 | 1.43E-08 | 2.24E-06 | 10.5680044 |
| Tyrobp | 1450792_at -2.6363241 | 10.4036869 | -15.557986 | 2.38E-07 | 1.52E-05 | 7.60346048 |
| S100a4 | 1424542_at -2.6286916 | 9.79848539 | -21.162369 | 2.04E-08 | 2.82E-06 | 10.1987879 |
| Tcrb-J | 1426772_x_a-2.626056 | 9.27452763 | -23.262607 | 9.56E-09 | 1.73E-06 | 10.9835777 |
| Tgfbi | 1437463_x_a-2.6202066 | 9.94773221 | -36.83789 | 2.33E-10 | 1.39E-07 | 14.6007435 |
| Lcn2 | 1427747_a_ -2.6146564 | 8.90852452 | -12.820587 | 1.09E-06 | 4.50E-05 | 5.96051048 |
| Tgfbi | 1415871_at -2.6073989 | 10.0020306 | -19.475257 | 3.98E-08 | 4.56E-06 | 9.50309109 |

| Ms4a7 | 1424754_at | -2.5966904 | 9.40791374 | -14.389965 | 4.40E-07 | 2.36E-05 | 6.94047981 |
| --- | --- | --- | --- | --- | --- | --- | --- |
| Cd44 | 1423760_at | -2.5961472 | 9.26858839 | -21.314819 | 1.93E-08 | 2.75E-06 | 10.2586296 |
| Gm12185 | 1425728_at | -2.5954867 | 9.00465744 | -38.761351 | 1.54E-10 | 1.06E-07 | 14.972585 |
| Basp1 | 1428572_at | -2.5926067 | 9.6674538 | -11.849506 | 2.01E-06 | 6.91E-05 | 5.29445252 |
| Slamf9 | 1419315_at | -2.5915024 | 9.7254631 | -14.066351 | 5.26E-07 | 2.65E-05 | 6.74727673 |
| Ifi209 | 1435331_at | -2.5908825 | 8.10230761 | -19.326769 | 4.23E-08 | 4.68E-06 | 9.43874518 |
| Parp9 | 1416897_at | -2.5904792 | 10.3383584 | -34.983034 | 3.54E-10 | 1.67E-07 | 14.2161725 |
| Selplg | 1449127_at | -2.5900856 | 9.41359354 | -13.734765 | 6.35E-07 | 3.02E-05 | 6.54471257 |
| Trim30a | 1451860_a_ | -2.5807643 | 9.27544543 | -35.131903 | 3.42E-10 | 1.65E-07 | 14.2480404 |
| Cytip | 1451206_s_a-2.572454 | | 8.54612413 | -14.99715 | 3.18E-07 | 1.87E-05 | 7.29159726 |
| Batf2 | 1430005_a_ -2.569231 | | 9.9516909 | -21.241287 | 1.98E-08 | 2.78E-06 | 10.2298253 |
| Cfb | 1417314_at -2.5618385 | | 9.14918797 | -19.339937 | 4.21E-08 | 4.68E-06 | 9.44447289 |
| Top2a | 1454694_a_ -2.5596222 | | 8.86833715 | -16.765212 | 1.31E-07 | 1.02E-05 | 8.23763424 |
| Trav9d-3 | 1452405_x_a-2.5588495 | | 9.87353396 | -18.000938 | 7.46E-08 | 6.96E-06 | 8.83959833 |
| C1qc | 1449401_at -2.5545951 | | 10.9972258 | -10.608936 | 4.71E-06 | 0.00012563 | 4.36513381 |
| Gimap7 | 1425084_at -2.5534908 | | 8.65906714 | -11.168487 | 3.17E-06 | 9.62E-05 | 4.79603474 |
| NA | 1452348_s_a-2.5432968 | | 9.95355733 | -24.332838 | 6.65E-09 | 1.35E-06 | 11.3531626 |
| Cd72 | 1426112_a_ -2.5375639 | | 8.84340311 | -14.230925 | 4.80E-07 | 2.49E-05 | 6.8460735 |
| Lst1 | 1425548_a_ -2.5363366 | | 9.85492583 | -12.068211 | 1.74E-06 | 6.28E-05 | 5.44885499 |
| Ptpn6 | 1460188_at -2.5222896 | | 9.09160045 | -9.7072758 | 9.27E-06 | 0.00020216 | 3.62579535 |
| Lgals3bp | 1448380_at -2.5161059 | | 10.7276712 | -22.050991 | 1.47E-08 | 2.27E-06 | 10.5410541 |
| Trav9d-3 | 1426113_x_a-2.5092088 | | 9.49392629 | -15.667808 | 2.25E-07 | 1.47E-05 | 7.66319637 |
| Vcam1 | 1448162_at -2.5071279 | | 10.4505804 | -17.678202 | 8.62E-08 | 7.73E-06 | 8.68665772 |
| Hmgb2 | 1437313_x_a-2.5048271 | | 8.59404266 | -25.495494 | 4.57E-09 | 1.06E-06 | 11.7339286 |
| NA | 1424518_at -2.5023867 | | 8.35996774 | -33.683391 | 4.81E-10 | 2.02E-07 | 13.9300997 |
| NA | 1420464_s_a-2.5014162 | | 8.8174882 | -13.792958 | 6.14E-07 | 2.94E-05 | 6.58060757 |
| Fcgr3 | 1448620_at -2.495731 | | 9.62934507 | -15.222765 | 2.82E-07 | 1.71E-05 | 7.41844677 |
| Rrm2 | 1434437_x_a-2.4890196 | | 8.24941965 | -30.648563 | 1.03E-09 | 3.40E-07 | 13.2023009 |
| Ctsz | 1417868_a_ -2.4879891 | | 10.6995754 | -14.987216 | 3.19E-07 | 1.87E-05 | 7.28596813 |
| Tmsb10 | 1436902_x_a-2.4851577 | | 11.7714565 | -21.133658 | 2.07E-08 | 2.82E-06 | 10.1874648 |
| Slamf8 | 1425294_at -2.476309 | | 8.7522509 | -17.372601 | 9.90E-08 | 8.61E-06 | 8.53910869 |
| NA | 1424775_at -2.4735868 | | 10.0724359 | -30.490022 | 1.08E-09 | 3.44E-07 | 13.1617733 |
| NA | 1417189_at -2.4721088 | | 11.496859 | -43.583546 | 5.94E-11 | 6.42E-08 | 15.8000404 |
| Cxcr6 | 1425832_a_ -2.4719155 | | 8.46844785 | -9.7867666 | 8.71E-06 | 0.00019264 | 3.69336239 |
| Ccr2 | 1421186_at -2.4697188 | | 8.21794681 | -14.091449 | 5.19E-07 | 2.64E-05 | 6.76241667 |
| Samhd1 | 1434438_at -2.4662528 | | 10.0785024 | -13.095354 | 9.23E-07 | 3.93E-05 | 6.14023299 |
| Zbp1 | 1419604_at -2.4639543 | | 9.09659789 | -19.460286 | 4.00E-08 | 4.56E-06 | 9.49662773 |
| Rnf19b | 1432478_a_ -2.4618234 | | 9.04733187 | -20.781658 | 2.36E-08 | 3.14E-06 | 10.0472416 |
| Tmsb10 | 1417219_s_a-2.452632 | | 10.7503092 | -21.757767 | 1.64E-08 | 2.44E-06 | 10.4298402 |
| Tmsb10 | 1437185_s_a-2.4438418 | | 12.7084155 | -19.429341 | 4.05E-08 | 4.57E-06 | 9.48325047 |
| NA | 1418638_at -2.442483 | | 8.58928207 | -16.540402 | 1.46E-07 | 1.11E-05 | 8.12316803 |
| Cxcl16 | 1449195_s_a-2.4358326 | | 8.66127715 | -13.548888 | 7.07E-07 | 3.28E-05 | 6.42905331 |
| Gimap4 | 1424374_at -2.4341428 | | 10.7633367 | -15.276339 | 2.75E-07 | 1.68E-05 | 7.44828967 |
| H2-K1 | 1451931_x_a-2.4338832 | | 12.8272669 | -25.249961 | 4.94E-09 | 1.13E-06 | 11.6552291 |
| Itgax | 1419128_at -2.4333118 | | 9.2806591 | -9.8942615 | 8.02E-06 | 0.00018219 | 3.78397275 |
| Epsti1 | 1454169_a_ -2.4325421 | | 7.90578138 | -10.036626 | 7.19E-06 | 0.0001695 | 3.90265238 |
| Upp1 | 1448562_at -2.4257175 | | 9.54315086 | -26.302264 | 3.55E-09 | 8.86E-07 | 11.9863639 |
| Creb1 | 1452349_x_a-2.425308 | | 11.3472378 | -28.981288 | 1.62E-09 | 4.72E-07 | 12.7624251 |
| Fgl2 | 1421855_at -2.4200356 | | 10.8440226 | -30.607848 | 1.04E-09 | 3.40E-07 | 13.1919183 |
| Pclaf | 1419153_at -2.4200097 | | 9.41419774 | -14.307545 | 4.61E-07 | 2.42E-05 | 6.89168541 |
| C1qa | 1417381_at -2.4181616 | | 11.5373135 | -12.367499 | 1.44E-06 | 5.48E-05 | 5.65592489 |
| AU020206 | 1433935_at -2.408558 | | 9.80049558 | -21.542973 | 1.77E-08 | 2.59E-06 | 10.347306 |
| Rnf19b | 1435226_at -2.4035731 | | 10.1109311 | -19.038992 | 4.77E-08 | 5.10E-06 | 9.31251052 |
| C1s1 | 1424041_s_a-2.4001509 | | 10.303343 | -14.32392 | 4.56E-07 | 2.42E-05 | 6.90140179 |
| Casp4 | 1449591_at -2.3910041 | | 9.53788658 | -21.26854 | 1.96E-08 | 2.77E-06 | 10.2405137 |
| Ms4a6c | 1450234_at -2.3865571 | | 10.5760236 | -19.805224 | 3.48E-08 | 4.11E-06 | 9.64420085 |
| Tap2 | 1453913_a_ -2.3858313 | | 10.070018 | -17.089763 | 1.13E-07 | 9.27E-06 | 8.40011086 |
| Wars | 1415694_at -2.3774796 | | 10.4176128 | -24.195729 | 6.96E-09 | 1.37E-06 | 11.3068698 |
| Naaa | 1452067_at -2.3747327 | | 9.7286443 | -20.713168 | 2.43E-08 | 3.18E-06 | 10.0196538 |
| Socs3 | 1456212_x_a-2.3745227 | | 9.10339523 | -12.792104 | 1.11E-06 | 4.55E-05 | 5.941669 |
| Sema4d | 1420824_at -2.3696007 | | 9.4551449 | -11.856464 | 2.00E-06 | 6.90E-05 | 5.29940572 |
| Plek | 1448749_at -2.3675185 | | 8.63512057 | -14.322303 | 4.57E-07 | 2.42E-05 | 6.90044267 |
| Trim30a | 1417961_a_ -2.3660867 | | 9.94271686 | -30.008059 | 1.22E-09 | 3.86E-07 | 13.0369277 |
| Snca | 1436853_a_ -2.3601267 | | 9.72254968 | -5.1685009 | 0.00080449 | 0.0049043 | -1.2526519 |
| Ltb | 1419135_at -2.3409824 | | 8.83106262 | -8.5184479 | 2.47E-05 | 0.00040025 | 2.55408461 |
| Casp1 | 1449265_at -2.3408608 | | 8.74249702 | -15.85207 | 2.05E-07 | 1.40E-05 | 7.76247164 |
| Tmem140 | 1424354_at -2.3405984 | | 9.77370863 | -28.668868 | 1.77E-09 | 5.02E-07 | 12.6765075 |
| Ido1 | 1420437_at -2.3375213 | | 8.62431895 | -13.889952 | 5.81E-07 | 2.83E-05 | 6.64010705 |
| Wars | 1434813_x_a-2.337117 | | 11.1842776 | -32.661311 | 6.17E-10 | 2.41E-07 | 13.6947511 |
| Laptm5 | 1426025_s_a-2.3282533 | | 10.262292 | -8.6048856 | 2.29E-05 | 0.00037923 | 2.63611545 |
| Slfn1 | 1418612_at -2.3268035 | | 8.27493772 | -9.7325319 | 9.09E-06 | 0.0001988 | 3.64731523 |
| Ifih1 | 1426276_at -2.3267426 | | 9.42590713 | -31.988146 | 7.30E-10 | 2.72E-07 | 13.5344703 |
| Evi2a | 1450241_a_ -2.3200632 | | 8.73794727 | -9.4525177 | 1.13E-05 | 0.00023301 | 3.4059579 |
| Uba7 | 1426970_a_ -2.3183183 | | 10.0910144 | -29.013702 | 1.61E-09 | 4.72E-07 | 12.7712738 |
| Ctsw | 1422632_at -2.3136774 | | 9.30300368 | -13.061474 | 9.41E-07 | 3.98E-05 | 6.11827013 |
| Hmgb2 | 1452534_a_ -2.3123603 | | 9.05304454 | -22.13774 | 1.42E-08 | 2.24E-06 | 10.5736394 |
| Nmi | 1425719_a_ -2.3121668 | | 9.65826057 | -38.318177 | 1.69E-10 | 1.13E-07 | 14.8892085 |
| Ccl12 | 1419282_at -2.3097513 | | 8.75331487 | -17.87583 | 7.88E-08 | 7.27E-06 | 8.78065666 |
| Glipr1 | 1424927_at -2.291983 | | 8.98448009 | -12.413397 | 1.40E-06 | 5.37E-05 | 5.68725951 |
| Tbx21 | 1449361_at -2.2854634 | | 8.1176956 | -18.775994 | 5.33E-08 | 5.55E-06 | 9.1953421 |
| Tapbp | 1421812_at -2.2842412 | | 11.45199 | -22.801446 | 1.12E-08 | 1.92E-06 | 10.8182863 |
| Ifi204 | 1419603_at -2.2818746 | | 7.77894084 | -34.264104 | 4.18E-10 | 1.90E-07 | 14.0596953 |
| Cd8a | 1451673_at -2.2812489 | | 9.12849823 | -15.248763 | 2.79E-07 | 1.69E-05 | 7.43294199 |
| Parp12 | 1426774_at -2.2760395 | | 10.0428329 | -36.718747 | 2.39E-10 | 1.39E-07 | 14.5768353 |
| Fxyd5 | 1418296_at -2.2753856 | | 10.6272951 | -23.334916 | 9.32E-09 | 1.73E-06 | 11.0091566 |

| NA | 1424948_x_a-2.2608385 | 11.5355306 | -22.148401 | 1.42E-08 | 2.24E-06 | 10.5776339 |
| --- | --- | --- | --- | --- | --- | --- |
| Slc4a1 | 1434502_x_a-2.2587557 | 8.43392598 | -4.2149812 | 0.00281227 | 0.01289098 | -2.60302 |
| Cxcr6 | 1422812_at -2.2580612 | 7.20091727 | -11.161155 | 3.19E-06 | 9.65E-05 | 4.79051721 |
| Pld4 | 1433678_at -2.2566943 | 9.30212796 | -9.101317 | 1.51E-05 | 0.00028306 | 3.09437543 |
| Fermt3 | 1433963_a_ -2.252543 | 8.12687973 | -10.102472 | 6.84E-06 | 0.00016365 | 3.95704159 |
| Casp12 | 1449297_at -2.252368 | 9.44889406 | -31.038957 | 9.32E-10 | 3.23E-07 | 13.3009794 |
| NA | 1456437_x_a-2.2515905 | 7.36958626 | -18.396767 | 6.27E-08 | 6.30E-06 | 9.02326982 |
| Rsad2 | 1421008_at -2.2460003 | 9.74525967 | -12.172738 | 1.63E-06 | 6.02E-05 | 5.5217206 |
| Cd68 | 1449164_at -2.2457313 | 9.4033449 | -11.187291 | 3.13E-06 | 9.53E-05 | 4.81016989 |
| Tent5c | 1448021_at -2.2439229 | 10.0000864 | -5.0794796 | 0.00089917 | 0.00534791 | -1.3733004 |
| Lpxn | 1424965_at -2.2366957 | 8.51615855 | -13.108497 | 9.15E-07 | 3.90E-05 | 6.14873859 |
| Cyba | 1454268_a_ -2.2286506 | 10.9171208 | -16.173816 | 1.75E-07 | 1.27E-05 | 7.93303002 |
| Icam1 | 1424067_at -2.2271977 | 9.62330222 | -12.618875 | 1.23E-06 | 4.89E-05 | 5.82620181 |
| H2-D1 | 1451784_x_a-2.2266801 | 12.9927105 | -23.55109 | 8.65E-09 | 1.65E-06 | 11.0850907 |
| Cybb | 1422978_at -2.223209 | 8.41926562 | -9.2492793 | 1.33E-05 | 0.00026028 | 3.22687883 |
| Arg1 | 1419549_at -2.2201159 | 7.88840705 | -15.969442 | 1.93E-07 | 1.36E-05 | 7.82509719 |
| Adgre1 | 1451161_a_ -2.2147983 | 9.36619327 | -14.849268 | 3.44E-07 | 1.96E-05 | 7.20740801 |
| Tnfaip2 | 1438855_x_a-2.2100783 | 10.3348818 | -17.93205 | 7.69E-08 | 7.15E-06 | 8.80719715 |
| Ms4a4d | 1418990_at -2.2073295 | 10.3712954 | -35.620735 | 3.05E-10 | 1.57E-07 | 14.3514261 |
| Id2 | 1422537_a_ -2.1998293 | 9.46716247 | -14.92802 | 3.30E-07 | 1.90E-05 | 7.2523458 |
| Fgl2 | 1421854_at -2.1977142 | 9.88258004 | -20.406838 | 2.74E-08 | 3.49E-06 | 9.89502554 |
| Hmox1 | 1448239_at -2.1923634 | 9.4691636 | -24.381203 | 6.55E-09 | 1.35E-06 | 11.3694207 |
| Ctsz | 1417869_s_a-2.1918701 | 9.58462693 | -13.460671 | 7.44E-07 | 3.40E-05 | 6.3736188 |
| NA | 1427511_at -2.18753 | 8.35629523 | -26.732854 | 3.12E-09 | 7.94E-07 | 12.117363 |
| Ctsz | 1417870_x_a-2.182753 | 10.4106169 | -12.784268 | 1.11E-06 | 4.57E-05 | 5.93647815 |
| NA | 1425545_x_a-2.177436 | 13.0962421 | -21.267323 | 1.96E-08 | 2.77E-06 | 10.2400368 |
| Arhgdib | 1426454_at -2.1753315 | 10.9266535 | -14.879786 | 3.38E-07 | 1.94E-05 | 7.22485047 |
| Il18bp | 1450424_a_ -2.1711021 | 9.54270974 | -24.646446 | 6.00E-09 | 1.25E-06 | 11.4579223 |
| Shisa5 | 1437503_a_ -2.1709455 | 11.6888349 | -20.244114 | 2.92E-08 | 3.62E-06 | 9.82798671 |
| Sh3bgrl3 | 1416528_at -2.1654371 | 11.2256276 | -15.872321 | 2.03E-07 | 1.40E-05 | 7.77331082 |
| C1ra | 1417009_at -2.1598969 | 9.88770526 | -13.239567 | 8.47E-07 | 3.70E-05 | 6.23310987 |
| Ptpn22 | 1417995_at -2.1589439 | 8.15848347 | -12.635435 | 1.22E-06 | 4.86E-05 | 5.83730543 |
| H2-K1 | 1425336_x_a-2.1539668 | 13.3915176 | -19.75337 | 3.55E-08 | 4.17E-06 | 9.62219488 |
| NA | 1416239_at -2.1445741 | 8.62364751 | -15.080246 | 3.04E-07 | 1.82E-05 | 7.33853893 |
| Pla2g7 | 1430700_a_ -2.144503 | 8.80497778 | -7.6996105 | 5.18E-05 | 0.00066002 | 1.74155737 |
| Themis2 | 1427041_at -2.1309778 | 9.98532025 | -20.748555 | 2.39E-08 | 3.16E-06 | 10.0339203 |
| Gatm | 1423569_at -2.1212236 | 8.24752741 | -12.152108 | 1.65E-06 | 6.07E-05 | 5.50738659 |
| Isg20 | 1419569_a_ -2.1088538 | 9.63986933 | -7.7864245 | 4.77E-05 | 0.00062304 | 1.83087125 |
| Ciita | 1421211_a_ -2.1078016 | 8.34304433 | -10.206146 | 6.33E-06 | 0.00015308 | 4.04204234 |
| Rassf5 | 1422637_at -2.1054439 | 7.8059785 | -11.042535 | 3.46E-06 | 0.00010249 | 4.70078772 |
| Ifi35 | 1424617_at -2.0997963 | 9.98926791 | -29.343642 | 1.47E-09 | 4.50E-07 | 12.8606565 |
| Ncf4 | 1418465_at -2.099165 | 8.95939646 | -11.995169 | 1.83E-06 | 6.46E-05 | 5.39758337 |
| Srgn | 1417426_at -2.0952104 | 11.7374235 | -18.292688 | 6.56E-08 | 6.46E-06 | 8.97538419 |
| Gmfg | 1419194_s_a-2.0908105 | 8.62382781 | -14.494946 | 4.16E-07 | 2.27E-05 | 7.00223159 |
| Dnase1l3 | 1421057_at -2.0899685 | 8.53442267 | -7.3321332 | 7.37E-05 | 0.00084578 | 1.35465456 |
| Ms4a4c | 1420671_x_a-2.0891023 | 9.15569294 | -19.078572 | 4.69E-08 | 5.04E-06 | 9.32999377 |
| Cd8a | 1425335_at -2.0890867 | 8.55776588 | -18.821458 | 5.23E-08 | 5.49E-06 | 9.21572169 |
| Fcgr2b | 1451941_a_ -2.080121 | 9.54932748 | -11.878767 | 1.97E-06 | 6.83E-05 | 5.31526506 |
| Ube2c | 1452954_at -2.0786615 | 8.03821763 | -30.948541 | 9.54E-10 | 3.23E-07 | 13.2782653 |
| NA | 1449347_a_ -2.0771286 | 8.29825796 | -15.355797 | 2.64E-07 | 1.63E-05 | 7.49235745 |
| Rassf5 | 1422638_s_a-2.0749616 | 8.70197463 | -12.721034 | 1.16E-06 | 4.68E-05 | 5.89447859 |
| Tspo | 1438948_x_a-2.0749057 | 10.5471118 | -14.157009 | 5.00E-07 | 2.55E-05 | 6.80184078 |
| Rab8b | 1426799_at -2.0748189 | 10.1381432 | -11.967676 | 1.86E-06 | 6.53E-05 | 5.37820814 |
| H2-K1 | 1450534_x_a-2.0729313 | 10.634455 | -19.900659 | 3.35E-08 | 4.02E-06 | 9.68453867 |
| Gpr65 | 1449175_at -2.0702467 | 7.35214828 | -10.820369 | 4.05E-06 | 0.00011317 | 4.53033355 |
| Ifi209 | 1435330_at -2.070071 | 8.71420954 | -9.270208 | 1.31E-05 | 0.00025759 | 3.24547479 |
| Cd2 | 1418770_at -2.0677776 | 7.8708134 | -14.442537 | 4.28E-07 | 2.31E-05 | 6.97145956 |
| Shisa5 | 1423986_a_ -2.0654354 | 11.0836241 | -16.008617 | 1.90E-07 | 1.36E-05 | 7.84589543 |
| NA | 1452178_at -2.0623921 | 9.69018127 | -20.883083 | 2.27E-08 | 3.05E-06 | 10.0879125 |
| C1qb | 1434366_x_a-2.0575325 | 9.86786174 | -16.387582 | 1.57E-07 | 1.16E-05 | 8.0444367 |
| Cxcl11 | 1419697_at -2.0492585 | 7.92579426 | -20.044372 | 3.16E-08 | 3.89E-06 | 9.74488779 |
| Fyb | 1452117_a_ -2.0485627 | 8.98487037 | -15.220813 | 2.83E-07 | 1.71E-05 | 7.41735771 |
| Grn | 1438629_x_a-2.0478045 | 12.4720871 | -23.372477 | 9.20E-09 | 1.73E-06 | 11.022408 |
| Klrc1 | 1425005_at -2.0459569 | 7.52899963 | -34.617839 | 3.85E-10 | 1.78E-07 | 14.1372256 |
| Ccrl2 | 1427736_a_ -2.0384919 | 9.79804119 | -22.276078 | 1.35E-08 | 2.19E-06 | 10.6253071 |
| Serpinb9 | 1422601_at -2.0364077 | 9.93672947 | -27.027698 | 2.85E-09 | 7.44E-07 | 12.2056189 |
| Casp12 | 1418981_at -2.0329425 | 9.25789303 | -25.688566 | 4.30E-09 | 1.03E-06 | 11.7951902 |
| Myo1g | 1427892_at -2.0327579 | 8.49949941 | -12.076596 | 1.73E-06 | 6.27E-05 | 5.45472193 |
| Tpm3 | 1427260_a_ -2.0189383 | 11.5447738 | -33.603014 | 4.90E-10 | 2.02E-07 | 13.9119311 |
| Slc11a1 | 1420361_at -2.0093774 | 9.24148596 | -11.318594 | 2.86E-06 | 8.92E-05 | 4.90826722 |
| NA | 1426906_at -2.0083924 | 10.369217 | -15.825565 | 2.08E-07 | 1.41E-05 | 7.74826403 |
| Efhd2 | 1431339_a_ -2.0015063 | 10.4287506 | -13.244192 | 8.44E-07 | 3.70E-05 | 6.23607247 |
| Hcls1 | 1418842_at -2.0013771 | 8.55009752 | -13.763061 | 6.25E-07 | 2.98E-05 | 6.56218493 |
| Vcam1 | 1415989_at -1.9962913 | 9.68703642 | -13.641503 | 6.70E-07 | 3.15E-05 | 6.48687429 |
| Arpc1b | 1416226_at -1.9932231 | 11.5780053 | -14.166789 | 4.98E-07 | 2.55E-05 | 6.80770646 |
| Tcrb-J | 1426159_x_a-1.9874254 | 9.79746861 | -15.406059 | 2.57E-07 | 1.61E-05 | 7.52011443 |
| Clec7a | 1420699_at -1.9869107 | 9.07341785 | -6.6796096 | 0.00014291 | 0.00139409 | 0.63035248 |
| Lyz2 | 1423547_at -1.9851077 | 12.3004565 | -10.268742 | 6.04E-06 | 0.00014863 | 4.09299372 |
| Mcm6 | 1416251_at -1.9840075 | 9.09395673 | -12.461794 | 1.36E-06 | 5.24E-05 | 5.72018149 |
| NA | 1452231_x_a-1.9839517 | 10.6623462 | -24.299028 | 6.73E-09 | 1.35E-06 | 11.3417752 |
| Irf8 | 1448452_at -1.9789974 | 8.83942097 | -21.712786 | 1.66E-08 | 2.47E-06 | 10.4126315 |
| Cxcl5 | 1419728_at -1.976633 | 7.55297692 | -9.734975 | 9.07E-06 | 0.00019861 | 3.6493943 |
| Trim12a | 1437432_a_ -1.9754082 | 8.26694102 | -23.489733 | 8.84E-09 | 1.67E-06 | 11.0636195 |
| H2-D1 | 1450170_x_a-1.9750366 | 7.39596733 | -32.144375 | 7.02E-10 | 2.69E-07 | 13.5720528 |
| Csf2rb2 | 1449360_at -1.9746826 | 8.32352607 | -10.759255 | 4.23E-06 | 0.00011648 | 4.48288569 |

| Fpr2 | 1422953_at | -1.9739143 | 9.01934812 | -19.216448 | 4.43E-08 | 4.81E-06 | 9.39059284 |
| --- | --- | --- | --- | --- | --- | --- | --- |
| C3ar1 | 1419482_at | -1.9670114 | 9.26842414 | -11.34489 | 2.81E-06 | 8.82E-05 | 4.92778694 |
| NA | 1449289_a_ | -1.9652743 | 13.1918438 | -23.836882 | 7.85E-09 | 1.52E-06 | 11.1842638 |
| Mthfd2 | 1419254_at | -1.9632706 | 8.43960331 | -20.262212 | 2.90E-08 | 3.61E-06 | 9.83547176 |
| Btg1 | 1426083_a_ | -1.9575782 | 10.7356147 | -12.330149 | 1.48E-06 | 5.54E-05 | 5.63034416 |
| Klrd1 | 1460245_at | -1.9541525 | 8.16956628 | -13.90332 | 5.77E-07 | 2.83E-05 | 6.64827561 |
| Msr1 | 1448061_at | -1.9539311 | 8.80418875 | -17.053948 | 1.15E-07 | 9.27E-06 | 8.38233932 |
| Mcm6 1438852_x_a-1.9469281 | | | 7.62702897 | -19.302789 | 4.27E-08 | 4.70E-06 | 9.42830389 |
| Pnp 1416530_a_ -1.9448887 | | | 10.8802958 | -28.387246 | 1.92E-09 | 5.37E-07 | 12.5980691 |
| Dram1 1424524_at -1.9446429 | | | 9.75775444 | -21.559365 | 1.76E-08 | 2.59E-06 | 10.3536368 |
| Cstb 1422506_a_ -1.9419714 | | | 11.4955756 | -26.104277 | 3.78E-09 | 9.19E-07 | 11.9252707 |
| Stmn1 1415849_s_a-1.9408697 | | | 10.2814078 | -11.239731 | 3.02E-06 | 9.28E-05 | 4.8494745 |
| Tapbpl 1451544_at -1.9389883 | | | 9.22352686 | -15.716759 | 2.19E-07 | 1.45E-05 | 7.6896849 |
| Clic1 1416656_at -1.9385798 | | | 10.2705358 | -23.240213 | 9.63E-09 | 1.73E-06 | 10.9756374 |
| Pirb 1424302_at -1.9379218 | | | 8.37918391 | -17.506016 | 9.31E-08 | 8.22E-06 | 8.60385515 |
| Birc5 1424278_a_ -1.9368618 | | | 8.10036034 | -12.56814 | 1.27E-06 | 5.00E-05 | 5.79209691 |
| Alox5ap 1452016_at -1.9328782 | | | 10.2479037 | -9.1488011 | 1.45E-05 | 0.00027519 | 3.13709707 |
| Nckap1l 1428787_at -1.9274118 | | | 8.77989563 | -9.1152419 | 1.49E-05 | 0.0002803 | 3.10692334 |
| AI504432 1456064_at -1.9260474 | | | 7.73257291 | -13.530924 | 7.14E-07 | 3.30E-05 | 6.41779375 |
| Snca 1418493_a_ -1.9229765 | | | 9.92330105 | -4.0386083 | 0.00359698 | 0.01560822 | -2.8664541 |
| Slfn2 1450165_at -1.9215618 | | | 9.93430505 | -13.723215 | 6.39E-07 | 3.03E-05 | 6.53757069 |
| Alas2 1451675_a_ -1.9213784 | | | 11.4055459 | -4.1367454 | 0.0031349 | 0.0140436 | -2.7193766 |
| Cndp2 1448263_a_ -1.9151028 | | | 10.4542915 | -20.333882 | 2.82E-08 | 3.54E-06 | 9.8650417 |
| H2-M3 1421358_at -1.9122741 | | | 9.6546227 | -15.522979 | 2.42E-07 | 1.54E-05 | 7.58432918 |
| Grn 1456567_x_a-1.905389 | | | 11.8381283 | -19.392057 | 4.12E-08 | 4.60E-06 | 9.46710272 |
| Mmp14 1448383_at -1.9042519 | | | 9.58778805 | -18.309919 | 6.51E-08 | 6.45E-06 | 8.98333206 |
| C3 1423954_at -1.8915306 | | | 12.2109243 | -11.808462 | 2.06E-06 | 7.01E-05 | 5.26517862 |
| Grn 1448148_at -1.8906991 | | | 11.2204817 | -11.93938 | 1.90E-06 | 6.63E-05 | 5.35822359 |
| Tapbp 1450378_at -1.8903087 | | | 9.95468487 | -10.846108 | 3.98E-06 | 0.00011196 | 4.55024404 |
| NA 1455504_a_ -1.8890689 | | | 10.3255642 | -5.1269928 | 0.00084721 | 0.00509929 | -1.3087672 |
| Wars 1437832_x_a-1.8870943 | | | 10.892993 | -30.591695 | 1.05E-09 | 3.40E-07 | 13.1877942 |
| Mcub 1418778_at -1.8785198 | | | 8.77640974 | -11.276752 | 2.95E-06 | 9.11E-05 | 4.87712098 |
| Sema4a 1448110_at -1.8717206 | | | 8.70245061 | -11.273474 | 2.95E-06 | 9.11E-05 | 4.87467678 |
| Tspo 1456251_x_a-1.8712887 | | | 9.98266453 | -14.580972 | 3.97E-07 | 2.21E-05 | 7.05250224 |
| B2m 1452428_a_ -1.8705219 | | | 13.6234449 | -16.479021 | 1.51E-07 | 1.12E-05 | 8.09163521 |
| Tmem106a 1425025_at -1.8676889 | | | 9.13227312 | -11.485779 | 2.56E-06 | 8.20E-05 | 5.03166148 |
| Tm6sf1 1424443_at -1.8668908 | | | 8.42092291 | -12.428296 | 1.39E-06 | 5.34E-05 | 5.69740706 |
| Btg1 1437455_a_ -1.8637666 | | | 10.0205865 | -22.153096 | 1.42E-08 | 2.24E-06 | 10.5793924 |
| Pycard 1417346_at -1.8625129 | | | 10.0318982 | -20.530374 | 2.61E-08 | 3.38E-06 | 9.94553083 |
| Prr13 1423686_a_ -1.8587707 | | | 10.0932114 | -12.595534 | 1.25E-06 | 4.94E-05 | 5.81052795 |
| Edem1 1424065_at -1.8514614 | | | 8.64118138 | -14.304749 | 4.61E-07 | 2.42E-05 | 6.89002505 |
| NA 1426505_at -1.8464045 | | | 8.14787554 | -13.817143 | 6.06E-07 | 2.92E-05 | 6.59548169 |
| NA 1453299_a_ -1.8435722 | | | 9.56174166 | -18.174661 | 6.91E-08 | 6.61E-06 | 8.92073009 |
| NA 1418809_at -1.8420211 | | | 7.51167449 | -13.850871 | 5.94E-07 | 2.87E-05 | 6.61618248 |
| Cks2 1417457_at -1.837857 | | | 8.01825441 | -14.69252 | 3.74E-07 | 2.09E-05 | 7.11724971 |
| B4galnt1 1418655_at -1.836998 | | | 8.27743784 | -10.013332 | 7.32E-06 | 0.00017181 | 3.88333596 |
| Dck 1439012_a_ -1.8369895 | | | 7.0927469 | -16.975702 | 1.19E-07 | 9.44E-06 | 8.34337746 |
| Fam49b 1423829_at -1.8270007 | | | 9.83220112 | -12.465397 | 1.36E-06 | 5.24E-05 | 5.72262741 |
| Lgals9 1421217_a_ -1.8240661 | | | 10.1599783 | -24.887419 | 5.55E-09 | 1.22E-06 | 11.5373724 |
| Rrm2 1448226_at -1.8224842 | | | 7.89017558 | -13.227482 | 8.53E-07 | 3.70E-05 | 6.22536453 |
| Tspo 1416695_at -1.8187537 | | | 11.103883 | -15.96373 | 1.94E-07 | 1.36E-05 | 7.82206063 |
| Slc2a3 1437052_s_a-1.8175574 | | | 8.63462471 | -15.48108 | 2.47E-07 | 1.56E-05 | 7.56137403 |
| NA 1451567_a_ -1.8172224 | | | 8.80390516 | -19.81503 | 3.46E-08 | 4.11E-06 | 9.64835554 |
| NA AFFX-18SRN -1.8170197 | | | 9.36223975 | -1.8732234 | 0.09721865 | 0.19088709 | -6.2472548 |
| Mkrn1 1418435_at -1.8154817 | | | 10.0111855 | -4.7421989 | 0.00138486 | 0.00745601 | -1.8405989 |
| Psme1 1417056_at -1.811826 | | | 12.2781478 | -28.106092 | 2.08E-09 | 5.68E-07 | 12.5188067 |
| Litaf 1416303_at -1.8094339 | | | 10.267886 | -13.773134 | 6.21E-07 | 2.97E-05 | 6.56839617 |
| Idnk 1424496_at -1.8009405 | | | 10.4121576 | -23.272646 | 9.52E-09 | 1.73E-06 | 10.9871344 |
| Trim34a 1424857_a_ -1.7969818 | | | 9.12756494 | -24.309636 | 6.70E-09 | 1.35E-06 | 11.3453502 |
| Cks2 1417458_s_a-1.796072 | | | 7.85827823 | -16.227195 | 1.70E-07 | 1.24E-05 | 7.9609898 |
| D17H6S56E- 1417821_at -1.7916958 | | | 8.37895133 | -9.8550647 | 8.26E-06 | 0.00018676 | 3.7510332 |
| Sash3 1427007_at -1.7905593 | | | 9.36808503 | -10.072177 | 7.00E-06 | 0.00016673 | 3.93205653 |
| Nfkbiz 1417483_at -1.7860265 | | | 8.65346265 | -22.388036 | 1.30E-08 | 2.15E-06 | 10.6668591 |
| Itk 1417171_at -1.7791457 | | | 8.09453641 | -14.736964 | 3.65E-07 | 2.05E-05 | 7.14291012 |
| Rab32 1416527_at -1.7762029 | | | 7.9354583 | -13.267595 | 8.33E-07 | 3.68E-05 | 6.25104722 |
| Akna 1427325_s_a-1.775725 | | | 8.56909676 | -13.249623 | 8.42E-07 | 3.69E-05 | 6.23954981 |
| Npc2 1448513_a_ -1.770422 | | | 11.7365458 | -10.487282 | 5.15E-06 | 0.00013285 | 4.26872751 |
| Nusap1 1416309_at -1.7702672 | | | 8.15434084 | -20.389003 | 2.75E-08 | 3.49E-06 | 9.88770646 |
| Serping1 1416625_at -1.7699215 | | | 12.3825138 | -17.286389 | 1.03E-07 | 8.82E-06 | 8.49699217 |
| Snx10 1431055_a_ -1.7643941 | | | 9.61633145 | -12.422031 | 1.39E-06 | 5.36E-05 | 5.6931414 |
| Cd24a 1416034_at -1.7612938 | | | 9.48091411 | -3.8031142 | 0.00503144 | 0.02014175 | -3.2242648 |
| Tpm3 1427567_a_ -1.7594591 | | | 8.91694469 | -14.74725 | 3.63E-07 | 2.04E-05 | 7.14883791 |
| Chil3 1419764_at -1.7585443 | | | 8.12644157 | -9.9265201 | 7.82E-06 | 0.00017893 | 3.81099574 |
| Stk17b 1423452_at -1.7585249 | | | 9.74725531 | -12.465191 | 1.36E-06 | 5.24E-05 | 5.72248739 |
| Cnp 1437341_x_a-1.7582526 | | | 9.57630899 | -15.344435 | 2.65E-07 | 1.64E-05 | 7.48607062 |
| Arrb2 1426239_s_a-1.756606 | | | 9.06144918 | -7.5637229 | 5.89E-05 | 0.0007216 | 1.60017265 |
| Fbn1 1460208_at -1.7557196 | | | 10.2203533 | -18.55597 | 5.85E-08 | 6.01E-06 | 9.09596348 |
| Mx1 1451905_a_ -1.7521154 | | | 7.69117753 | -15.630652 | 2.29E-07 | 1.48E-05 | 7.64303385 |
| Ube2l6 1417172_at -1.7510067 | | | 8.1593068 | -10.165867 | 6.53E-06 | 0.00015687 | 4.00910932 |
| Dck 1449176_a_ -1.7458358 | | | 7.39985895 | -13.930434 | 5.68E-07 | 2.81E-05 | 6.66481917 |
| Aldh1a2 1422789_at -1.7445395 | | | 9.20407953 | -19.277459 | 4.32E-08 | 4.73E-06 | 9.41725952 |
| NA 1418021_at -1.7437914 | | | 10.9217871 | -11.087631 | 3.36E-06 | 0.00010037 | 4.73500408 |
| Spi1 1418747_at -1.7407623 | | | 8.42362483 | -8.3886633 | 2.76E-05 | 0.00043475 | 2.42962282 |
| Emp3 1417104_at -1.7342457 | | | 9.73826678 | -14.437982 | 4.29E-07 | 2.31E-05 | 6.96877968 |
| Fam107b 1416892_s_a-1.7336152 | | | 8.61436101 | -14.434731 | 4.30E-07 | 2.31E-05 | 6.96686675 |

| Arpc5 | 1448129_at | -1.7330112 | 10.6881434 | -15.488588 | 2.46E-07 | 1.56E-05 | 7.56549203 |
| --- | --- | --- | --- | --- | --- | --- | --- |
| Rps6ka1 | 1416896_at | -1.7301643 | 8.74548852 | -9.0983623 | 1.51E-05 | 0.00028318 | 3.09171082 |
| Iqgap1 | 1417379_at | -1.7299521 | 10.2512446 | -14.941206 | 3.27E-07 | 1.89E-05 | 7.25984642 |
| NA | 1439436_x_a-1.7253005 | | 8.133077 | -16.82919 | 1.27E-07 | 9.96E-06 | 8.26991962 |
| Slc9a3r1 | 1438116_x_a-1.7197905 | | 9.66498895 | -9.1937683 | 1.40E-05 | 0.00026772 | 3.17738067 |
| Pstpip1 | 1424560_at -1.7190957 | | 8.10278025 | -9.4272114 | 1.16E-05 | 0.00023564 | 3.38384131 |
| NA | 1456494_a_ -1.7187618 | | 8.99717333 | -19.98262 | 3.24E-08 | 3.93E-06 | 9.71901424 |
| Ankrd44 | 1434856_at -1.7180935 | | 8.82322123 | -10.78528 | 4.15E-06 | 0.00011462 | 4.50312134 |
| Cd5 | 1418353_at -1.7129817 | | 8.07951453 | -14.855583 | 3.42E-07 | 1.96E-05 | 7.21101995 |
| Stk17b | 1450997_at -1.6989766 | | 9.36381606 | -12.606255 | 1.24E-06 | 4.92E-05 | 5.81773069 |
| Ddx60 | 1451777_at -1.6987761 | | 8.0516233 | -17.820553 | 8.08E-08 | 7.38E-06 | 8.7544754 |
| Sirpa | 1416985_at -1.6962838 | | 9.46977613 | -7.0804684 | 9.46E-05 | 0.00102263 | 1.08113126 |
| Vim | 1456292_a_ -1.6958233 | | 11.325348 | -11.71361 | 2.20E-06 | 7.31E-05 | 5.19716043 |
| Cd24a | 1448182_a_ -1.6957496 | | 9.49210825 | -3.8267697 | 0.00486291 | 0.01964384 | -3.1880238 |
| Sat1 | 1420502_at -1.6951058 | | 9.85172251 | -11.195863 | 3.12E-06 | 9.51E-05 | 4.81660638 |
| Fcgr2b | 1455332_x_a-1.6933379 | | 9.34245264 | -10.530061 | 4.99E-06 | 0.00013041 | 4.30274285 |
| Il1rn | 1451798_at -1.6916721 | | 7.97725627 | -15.799604 | 2.10E-07 | 1.42E-05 | 7.73432449 |
| Smc2 | 1448635_at -1.6908033 | | 8.34003681 | -15.50293 | 2.44E-07 | 1.55E-05 | 7.57335266 |
| Socs3 | 1416576_at -1.6892071 | | 9.16017268 | -8.5871347 | 2.32E-05 | 0.00038367 | 2.61932522 |
| Slc4a1 | 1416464_at -1.686968 | | 8.55339417 | -4.7606674 | 0.00135192 | 0.00733316 | -1.8145937 |
| Prelid1 | 1439253_x_a-1.6861001 | | 10.8233144 | -17.811037 | 8.12E-08 | 7.38E-06 | 8.74995987 |
| Mt2 | 1428942_at -1.6829872 | | 11.7874366 | -6.0254449 | 0.00029136 | 0.00235433 | -0.1476437 |
| Adcy7 | 1456307_s_a-1.6828063 | | 9.55984958 | -12.181633 | 1.62E-06 | 6.00E-05 | 5.52789404 |
| Actb | AFFX-b-Actin -1.6826546 | | 12.8496261 | -15.733952 | 2.17E-07 | 1.44E-05 | 7.69896902 |
| Sla | 1420819_at -1.6822213 | | 8.71480817 | -12.461342 | 1.36E-06 | 5.24E-05 | 5.71987431 |
| Prelid1 | 1448202_x_a-1.6811056 | | 10.6677603 | -21.797249 | 1.61E-08 | 2.42E-06 | 10.444912 |
| Adam8 | 1416871_at -1.6808966 | | 8.33936449 | -12.344589 | 1.46E-06 | 5.52E-05 | 5.64024236 |
| Ptpn1 | 1417068_a_ -1.6779239 | | 9.69704872 | -13.637374 | 6.71E-07 | 3.15E-05 | 6.48430491 |
| Ccr2 | 1421187_at -1.6777576 | | 8.07249538 | -9.8055084 | 8.59E-06 | 0.00019107 | 3.709223 |
| Unc93b1 | 1423768_at -1.6766288 | | 9.90007743 | -7.7316907 | 5.02E-05 | 0.00064618 | 1.77465253 |
| Gimap3 | 1449220_at -1.6761407 | | 9.33969762 | -7.5477821 | 5.98E-05 | 0.00072972 | 1.58345876 |
| Mkrn1 | 1434853_x_a-1.6699444 | | 9.21000033 | -8.466055 | 2.58E-05 | 0.0004151 | 2.50402875 |
| Ccr5 | 1422259_a_ -1.6686577 | | 8.52990463 | -10.953639 | 3.69E-06 | 0.00010683 | 4.63296264 |
| Ddit3 | 1417516_at -1.6679731 | | 10.2223674 | -16.576948 | 1.44E-07 | 1.10E-05 | 8.14188483 |
| Cxcl16 | 1418718_at -1.6675338 | | 10.0713693 | -10.25262 | 6.12E-06 | 0.0001496 | 4.07989749 |
| Gna13 | 1422555_s_a-1.667356 | | 9.88135254 | -15.649432 | 2.27E-07 | 1.48E-05 | 7.65323055 |
| Iqgap1 | 1417380_at -1.6663258 | | 10.3493819 | -10.399055 | 5.49E-06 | 0.0001384 | 4.19818026 |
| Xdh | 1451006_at -1.6648515 | | 11.1716607 | -15.609408 | 2.32E-07 | 1.50E-05 | 7.63148398 |
| Spp1 | 1449254_at -1.6634782 | | 7.7104316 | -18.15681 | 6.96E-08 | 6.64E-06 | 8.91243149 |
| Col3a1 | 1427884_at -1.6623018 | | 8.79247168 | -11.383108 | 2.74E-06 | 8.66E-05 | 4.95608161 |
| Apobec3 | 1417470_at -1.6610696 | | 9.00248532 | -6.5406804 | 0.00016555 | 0.00154961 | 0.46962541 |
| Rgs1 | 1417601_at -1.6609653 | | 8.15905076 | -19.935291 | 3.30E-08 | 3.98E-06 | 9.69912495 |
| Actb | 1436722_a_ -1.659473 | | 13.2479065 | -14.969026 | 3.22E-07 | 1.88E-05 | 7.27565071 |
| Tpm3 | 1436958_x_a-1.6594672 | | 10.2836414 | -20.465327 | 2.67E-08 | 3.43E-06 | 9.91897944 |
| Scpep1 | 1455908_a_ -1.6545705 | | 9.98212491 | -11.686922 | 2.24E-06 | 7.41E-05 | 5.17792989 |
| Trim21 | 1448940_at -1.6520534 | | 9.86913264 | -25.596572 | 4.42E-09 | 1.04E-06 | 11.7660683 |
| Vwa5a | 1426221_at -1.6502248 | | 10.6484799 | -27.039902 | 2.84E-09 | 7.44E-07 | 12.2092469 |
| Rap1b | 1435519_at -1.6493504 | | 11.2479152 | -14.943898 | 3.27E-07 | 1.89E-05 | 7.26137735 |
| Erap1 | 1416942_at -1.6475206 | | 9.0822327 | -18.521091 | 5.94E-08 | 6.07E-06 | 9.08009416 |
| Tmem71 | 1436212_at -1.6473509 | | 9.56920525 | -20.3276 | 2.82E-08 | 3.54E-06 | 9.86245454 |
| Lyz1 | 1436996_x_a-1.6447001 | | 12.6586014 | -10.965164 | 3.66E-06 | 0.00010619 | 4.64178408 |
| Itgb7 | 1418741_at -1.6421499 | | 9.34569007 | -8.8435698 | 1.87E-05 | 0.00032794 | 2.85913579 |
| Mcm7 | 1438320_s_a-1.6405894 | | 9.57998408 | -15.013392 | 3.15E-07 | 1.86E-05 | 7.30079311 |
| Cfl1 | 1455138_x_a-1.6369688 | | 12.3228489 | -25.565861 | 4.47E-09 | 1.04E-06 | 11.756319 |
| Litaf | 1416304_at -1.6349646 | | 10.0751875 | -16.419483 | 1.55E-07 | 1.15E-05 | 8.06093379 |
| Samhd1 | 1420272_at -1.6346412 | | 9.01456151 | -11.842054 | 2.02E-06 | 6.93E-05 | 5.28914473 |
| Arl6ip1 | 1423819_s_a-1.6294842 | | 9.99252532 | -13.4164 | 7.63E-07 | 3.45E-05 | 6.34566551 |
| Itgam | 1422046_at -1.6275376 | | 7.91660899 | -14.83731 | 3.46E-07 | 1.97E-05 | 7.20056364 |
| Bst1 | 1449453_at -1.6270879 | | 8.21176713 | -15.750554 | 2.16E-07 | 1.44E-05 | 7.70792393 |
| Was | 1419631_at -1.6269643 | | 8.58556916 | -8.9454588 | 1.71E-05 | 0.00030811 | 2.95280942 |
| Tlr1 | 1449049_at -1.624674 | | 7.95846708 | -12.343364 | 1.46E-06 | 5.52E-05 | 5.63940315 |
| NA | 1448775_at -1.6237504 | | 10.0018927 | -16.033 | 1.87E-07 | 1.35E-05 | 7.85881389 |
| Arl6ip1 | 1423818_a_ -1.620498 | | 10.751361 | -9.0539056 | 1.57E-05 | 0.0002903 | 3.05153006 |
| Lasp1 | 1438634_x_a-1.6167568 | | 10.0662416 | -19.420791 | 4.07E-08 | 4.57E-06 | 9.47955057 |
| D17H6S56E- | 1417822_at -1.6152535 | | 8.95495852 | -9.2370897 | 1.35E-05 | 0.00026242 | 3.21603142 |
| P2ry6 | 1425214_at -1.6151682 | | 8.91843215 | -8.0345288 | 3.80E-05 | 0.00053477 | 2.08187312 |
| NA | 1418364_a_ -1.6146744 | | 11.3441422 | -12.131138 | 1.67E-06 | 6.12E-05 | 5.49279257 |
| Gpsm3 | 1418396_at -1.6141727 | | 8.84659293 | -6.4832206 | 0.00017604 | 0.00162571 | 0.40245477 |
| Dock2 | 1422808_s_a-1.6105565 | | 8.87846693 | -8.0738096 | 3.66E-05 | 0.00052077 | 2.12104673 |
| Arf3 | 1437331_a_ -1.6093903 | | 9.58297475 | -14.981909 | 3.20E-07 | 1.87E-05 | 7.28295941 |
| Gpx1 | 1460671_at -1.6075627 | | 12.2776825 | -10.619908 | 4.67E-06 | 0.00012522 | 4.3737795 |
| Arrb2 | 1451987_at -1.6068429 | | 9.11528746 | -6.7627182 | 0.00013102 | 0.00129929 | 0.72537782 |
| Pla2g16 | 1451611_at -1.6066292 | | 11.6090296 | -21.135685 | 2.06E-08 | 2.82E-06 | 10.1882647 |
| Cnn3 | 1436836_x_a-1.6002044 | | 10.5306715 | -23.578698 | 8.57E-09 | 1.65E-06 | 11.0947311 |
| Rbl1 | 1424156_at -1.5980505 | | 8.31199909 | -14.069784 | 5.25E-07 | 2.65E-05 | 6.7493492 |
| Klrg1 | 1420788_at -1.5965047 | | 8.79831287 | -16.398671 | 1.57E-07 | 1.16E-05 | 8.05017466 |
| Cks1b | 1416698_a_ -1.5957029 | | 9.83430263 | -14.554824 | 4.02E-07 | 2.23E-05 | 7.03725393 |
| Npc2 | 1416901_at -1.5939731 | | 11.0244625 | -7.8382513 | 4.55E-05 | 0.00060406 | 1.88381952 |
| Cst7 | 1419202_at -1.5935698 | | 8.01180463 | -12.617297 | 1.23E-06 | 4.89E-05 | 5.82514292 |
| Glrx | 1416592_at -1.5928895 | | 10.3287457 | -16.318777 | 1.63E-07 | 1.20E-05 | 8.00874173 |
| Slc25a37 | 1417750_a_ -1.590856 | | 10.0342152 | -3.9305583 | 0.00419165 | 0.01749284 | -3.0297925 |
| Cd38 | 1433741_at -1.5908519 | | 9.69687318 | -15.822455 | 2.08E-07 | 1.41E-05 | 7.74659526 |
| Pfkfb3 | 1416432_at -1.5901938 | | 9.01940331 | -6.4526792 | 0.00018192 | 0.00166371 | 0.36658438 |
| Cndp2 | 1460177_at -1.5890465 | | 10.5233358 | -11.603051 | 2.36E-06 | 7.70E-05 | 5.11722717 |
| Limd2 | 1429104_at -1.5854961 | | 9.19691212 | -10.980776 | 3.62E-06 | 0.00010522 | 4.65372011 |

| Evl | 1434920_a_ -1.5816465 | 8.95033463 | -6.6862603 | 0.00014192 | 0.00138559 | 0.63798754 |
| --- | --- | --- | --- | --- | --- | --- |
| Vim | 1450641_at -1.5813611 | 12.9501291 | -14.547286 | 4.04E-07 | 2.23E-05 | 7.03285274 |
| Arhgap9 | 1419810_x_a-1.5780426 | 7.89773353 | -7.9281144 | 4.18E-05 | 0.00057293 | 1.97497742 |
| Procr | 1420664_s_a-1.5769873 | 8.13610093 | -11.702212 | 2.21E-06 | 7.34E-05 | 5.18895262 |
| Col3a1 | 1427883_a_ -1.5753906 | 12.1740256 | -12.26947 | 1.53E-06 | 5.73E-05 | 5.58862789 |
| Cstb | 1422507_at -1.5705782 | 9.93834478 | -19.818505 | 3.46E-08 | 4.11E-06 | 9.64982688 |
| Cd84 | 1422875_at -1.5701762 | 8.16406489 | -6.8749952 | 0.00011665 | 0.00119384 | 0.85243963 |
| Skap2 | 1418895_at -1.5701303 | 9.55637876 | -14.099434 | 5.17E-07 | 2.63E-05 | 6.76722812 |
| Acsl5 | 1428082_at -1.5688058 | 10.357122 | -9.8723017 | 8.15E-06 | 0.00018466 | 3.76553271 |
| Tlr3 | 1422782_s_a-1.5663252 | 8.65268965 | -21.353206 | 1.90E-08 | 2.74E-06 | 10.273623 |
| NA | 1439426_x_a-1.5659141 | 11.7179816 | -10.432417 | 5.36E-06 | 0.00013657 | 4.22491958 |
| Hdc | 1454713_s_a-1.5606579 | 7.76734979 | -13.202605 | 8.65E-07 | 3.75E-05 | 6.20939918 |
| Rap1b | 1435518_at -1.5593066 | 10.6279324 | -17.92308 | 7.72E-08 | 7.15E-06 | 8.80296851 |
| Marcksl1 | 1437226_x_a-1.5577324 | 8.62267062 | -9.9687038 | 7.57E-06 | 0.00017545 | 3.84621654 |
| Wars | 1425106_a_ -1.5550869 | 8.72565711 | -11.630309 | 2.32E-06 | 7.62E-05 | 5.13700047 |
| Myd88 | 1419272_at -1.5526104 | 9.81536027 | -19.6044 | 3.77E-08 | 4.34E-06 | 9.55862497 |
| Klra2 | 1421304_at -1.551493 | 7.31241073 | -7.2755948 | 7.79E-05 | 0.00088414 | 1.29382409 |
| Anxa4 | 1421223_a_ -1.5482503 | 9.88790961 | -13.305003 | 8.15E-07 | 3.63E-05 | 6.27492965 |
| Taok3 | 1435964_a_ -1.5454443 | 8.06399679 | -15.361869 | 2.63E-07 | 1.63E-05 | 7.49571551 |
| Nckap1l | 1428786_at -1.5449321 | 8.94733309 | -9.5899854 | 1.02E-05 | 0.00021575 | 3.52521212 |
| Pltp | 1417963_at -1.5447449 | 9.9340177 | -7.4554474 | 6.54E-05 | 0.00077872 | 1.48610936 |
| Cd44 | 1452483_a_ -1.5433306 | 7.77075808 | -10.236302 | 6.19E-06 | 0.00015091 | 4.06662302 |
| Hif1a | 1427418_a_ -1.5428072 | 10.6919234 | -23.988659 | 7.46E-09 | 1.46E-06 | 11.2363786 |
| Slfn8 | 1451655_at -1.5382973 | 8.19160107 | -14.890755 | 3.36E-07 | 1.94E-05 | 7.23111127 |
| Sh3bp2 | 1448328_at -1.5382091 | 8.04700484 | -8.6163164 | 2.27E-05 | 0.00037662 | 2.64691248 |
| Glrx | 1416593_at -1.5317038 | 9.9599271 | -15.324246 | 2.68E-07 | 1.64E-05 | 7.47488693 |
| Cep55 | 1452242_at -1.5311442 | 7.71728744 | -11.021049 | 3.52E-06 | 0.00010336 | 4.6844403 |
| Bpgm | 1415864_at -1.5307933 | 9.2220905 | -3.2146556 | 0.01202881 | 0.03972254 | -4.1438618 |
| Samhd1 | 1420273_x_a-1.5280441 | 8.9220168 | -13.162639 | 8.86E-07 | 3.82E-05 | 6.18368887 |
| Msr1 | 1422062_at -1.5260304 | 9.21339574 | -14.044427 | 5.33E-07 | 2.67E-05 | 6.73402923 |
| Mkrn1 | 1451425_a_ -1.5241195 | 8.80434086 | -5.1966279 | 0.00077688 | 0.00478081 | -1.2147649 |
| Ppa1 | 1416939_at -1.5236877 | 11.2827295 | -12.23586 | 1.57E-06 | 5.84E-05 | 5.5654376 |
| Ccnb2 | 1450920_at -1.5230849 | 8.32732464 | -11.898112 | 1.95E-06 | 6.77E-05 | 5.32899813 |
| Adcy7 | 1450065_at -1.5230554 | 8.25449126 | -7.7176876 | 5.09E-05 | 0.0006518 | 1.76021956 |
| Ucp2 | 1448188_at -1.5193284 | 10.9379162 | -9.0192833 | 1.61E-05 | 0.00029534 | 3.02012189 |
| Cdc20 | 1439377_x_a-1.5186766 | 8.21126966 | -15.334676 | 2.66E-07 | 1.64E-05 | 7.48066652 |
| Bpgm | 1415865_s_a-1.5186537 | 10.2876424 | -3.679429 | 0.00602016 | 0.0231545 | -3.4147777 |
| NA | 1451318_a_ -1.5169906 | 10.0881732 | -11.304913 | 2.89E-06 | 8.97E-05 | 4.89809465 |
| Cdkn1a | 1421679_a_ -1.5166608 | 9.84133481 | -8.8123669 | 1.92E-05 | 0.00033372 | 2.83026778 |
| C3ar1 | 1419483_at -1.516336 | 8.69963428 | -9.4842459 | 1.10E-05 | 0.00022887 | 3.43361488 |
| Glipr2 | 1428492_at -1.516256 | 8.26705006 | -12.095813 | 1.71E-06 | 6.22E-05 | 5.46815423 |
| Anxa2 | 1419091_a_ -1.5157491 | 11.7825651 | -15.032005 | 3.12E-07 | 1.85E-05 | 7.31131889 |
| NA | 1425763_x_a-1.5152701 | 9.26816941 | -4.9549374 | 0.00105262 | 0.00603731 | -1.5439735 |
| Tubb5 | 1455719_at -1.5132626 | 10.678279 | -11.702398 | 2.21E-06 | 7.34E-05 | 5.18908676 |
| Gna13 | 1450656_at -1.5105231 | 9.19374874 | -13.617441 | 6.79E-07 | 3.18E-05 | 6.47188918 |
| Hexb | 1437874_s_a-1.5103808 | 9.70255164 | -10.120948 | 6.75E-06 | 0.00016208 | 3.97224586 |
| Nrros | 1451174_at -1.5072463 | 9.12068608 | -7.3417862 | 7.30E-05 | 0.00084195 | 1.36500496 |
| Casp8 | 1424552_at -1.5071389 | 9.29240302 | -12.846458 | 1.07E-06 | 4.46E-05 | 5.97758976 |
| Prelid1 | 1431420_s_a-1.5050621 | 10.6259471 | -12.779713 | 1.12E-06 | 4.57E-05 | 5.93345936 |
| Tlr3 | 1422781_at -1.502955 | 7.74930387 | -18.361694 | 6.37E-08 | 6.36E-06 | 9.00716515 |
| Cmpk2 | 1450484_a_ -1.5018347 | 8.53319221 | -19.606543 | 3.77E-08 | 4.34E-06 | 9.55954285 |
| NA | 1425598_a_ -1.5011001 | 9.09877384 | -8.2280747 | 3.19E-05 | 0.00047814 | 2.27342803 |
| Psat1 | 1451064_a_ -1.5005757 | 8.31854839 | -20.470626 | 2.67E-08 | 3.43E-06 | 9.92114586 |
| Lair1 | 1430447_a_ -1.4981537 | 8.05854665 | -8.3522327 | 2.85E-05 | 0.00044218 | 2.39440347 |
| Capza1 | 1439455_x_a-1.4979895 | 9.78350579 | -18.247971 | 6.69E-08 | 6.49E-06 | 8.95472133 |
| Pltp | 1456424_s_a-1.4964557 | 10.9052068 | -8.8620412 | 1.84E-05 | 0.00032368 | 2.87618482 |
| Ccnd1 | 1417420_at -1.49388 | 10.3491374 | -14.941046 | 3.27E-07 | 1.89E-05 | 7.25975588 |
| Tagln2 | 1426529_a_ -1.4928334 | 10.7469782 | -14.372279 | 4.44E-07 | 2.38E-05 | 6.93003269 |
| Helz2 | 1435454_a_ -1.4925006 | 9.09279532 | -18.283328 | 6.59E-08 | 6.46E-06 | 8.97106353 |
| Fam111a | 1422628_at -1.492382 | 8.78419987 | -10.707823 | 4.39E-06 | 0.00011923 | 4.44276497 |
| Myh7 | 1448553_at -1.489487 | 10.3213182 | -4.1079879 | 0.00326331 | 0.0144703 | -2.7623471 |
| Cxcr3 | 1449925_at -1.4883213 | 8.65285687 | -8.4090643 | 2.71E-05 | 0.00042937 | 2.44929112 |
| Runx2 | 1424704_at -1.4876597 | 7.72452007 | -8.670443 | 2.16E-05 | 0.00036403 | 2.69787772 |
| Lat | 1460651_at -1.4868601 | 8.91190902 | -12.17493 | 1.63E-06 | 6.02E-05 | 5.52324244 |
| Arhgap45 | 1428242_at -1.482716 | 8.31235005 | -9.3533985 | 1.23E-05 | 0.00024625 | 3.31903879 |
| Soat1 | 1417695_a_ -1.4815884 | 8.60813413 | -11.978882 | 1.85E-06 | 6.51E-05 | 5.38611078 |
| Serpina3n | 1419100_at -1.4801573 | 9.54978869 | -9.9056819 | 7.95E-06 | 0.00018108 | 3.79354847 |
| Ly6a | 1417185_at -1.4781994 | 13.446524 | -17.333977 | 1.01E-07 | 8.73E-06 | 8.52026696 |
| Myh9 | 1420172_at -1.4749444 | 10.5842018 | -10.086121 | 6.93E-06 | 0.00016516 | 3.94356432 |
| S100a10 | 1416762_at -1.4726113 | 11.1864857 | -13.506982 | 7.24E-07 | 3.33E-05 | 6.40276398 |
| Slamf7 | 1453472_a_ -1.4719275 | 7.7497945 | -11.874293 | 1.98E-06 | 6.84E-05 | 5.31208607 |
| Mthfd2 | 1419253_at -1.4711691 | 9.4543151 | -12.28644 | 1.52E-06 | 5.68E-05 | 5.60031437 |
| Mpp1 | 1450919_at -1.4674133 | 10.1870466 | -9.624174 | 9.89E-06 | 0.00021247 | 3.55464078 |
| Cotl1 | 1416002_x_a-1.4666104 | 8.65361767 | -7.4773737 | 6.40E-05 | 0.00076886 | 1.50930969 |
| H2afz | 1438092_x_a-1.4638339 | 9.56071101 | -17.757992 | 8.31E-08 | 7.51E-06 | 8.72474129 |
| Trim59 | 1416118_at -1.4632256 | 7.70597959 | -12.799461 | 1.10E-06 | 4.55E-05 | 5.94653936 |
| Tm6sf1 | 1451353_at -1.4624616 | 9.36280868 | -9.0485209 | 1.57E-05 | 0.00029095 | 3.04665192 |
| Lasp1 | 1438633_x_a-1.4617716 | 9.69571972 | -20.019373 | 3.19E-08 | 3.91E-06 | 9.73442394 |
| Pfkp | 1416069_at -1.4611638 | 10.4922319 | -13.251159 | 8.41E-07 | 3.69E-05 | 6.24053303 |
| Tnc | 1416342_at -1.4601844 | 8.31073783 | -9.1276884 | 1.47E-05 | 0.00027833 | 3.1181252 |
| Trafd1 | 1428346_at -1.4597113 | 10.4091916 | -16.016058 | 1.89E-07 | 1.36E-05 | 7.84983985 |
| Arf6 | 1418822_a_ -1.4589728 | 10.397116 | -20.900815 | 2.26E-08 | 3.05E-06 | 10.0950004 |
| Bcl11b | 1450339_a_ -1.4573176 | 8.25170844 | -8.2938399 | 3.00E-05 | 0.00045839 | 2.33769047 |
| Myh9 | 1420171_s_a-1.4569358 | 11.3272924 | -12.709806 | 1.17E-06 | 4.70E-05 | 5.88700059 |
| Mob1a | 1423782_at -1.4525294 | 9.34196329 | -14.185735 | 4.93E-07 | 2.53E-05 | 6.81905771 |

| Il6 | 1450297_at | -1.4490552 | 7.21948761 | -11.172188 | 3.17E-06 | 9.62E-05 | 4.79881879 |
| --- | --- | --- | --- | --- | --- | --- | --- |
| Txn1 | 1416119_at | -1.4472735 | 11.8116583 | -24.873682 | 5.57E-09 | 1.22E-06 | 11.5328672 |
| Lrrfip1 | 1433842_at | -1.4453764 | 9.55851658 | -13.156128 | 8.90E-07 | 3.82E-05 | 6.17949332 |
| Fermt3 | 1433964_s_a-1.4434073 | | 8.69326979 | -11.410567 | 2.69E-06 | 8.53E-05 | 4.97635663 |
| Sema4a | 1438934_x_a-1.4427651 | | 8.02387536 | -11.018575 | 3.52E-06 | 0.00010341 | 4.68255588 |
| Actr3 | 1452051_at -1.4418944 | | 11.3903789 | -12.1823 | 1.62E-06 | 6.00E-05 | 5.52835705 |
| Cap1 | 1417461_at -1.4400178 | | 9.34520094 | -10.495339 | 5.12E-06 | 0.00013236 | 4.27514383 |
| Ccna2 | 1417910_at -1.4390809 | | 8.75189139 | -14.047285 | 5.32E-07 | 2.67E-05 | 6.73575707 |
| Gypa | 1423016_a_ -1.4370601 | | 7.69400619 | -4.1349233 | 0.00314287 | 0.01407098 | -2.7220961 |
| Cd82 | 1416401_at -1.4348995 | | 9.74942973 | -8.0124642 | 3.87E-05 | 0.00054194 | 2.05980163 |
| Vim | 1438118_x_a-1.4348308 | | 12.1949777 | -15.694955 | 2.22E-07 | 1.46E-05 | 7.67789647 |
| Trim30d | 1438716_at -1.4333223 | | 7.92638424 | -17.216493 | 1.06E-07 | 9.04E-06 | 8.46268569 |
| Mvp | 1448618_at -1.4313575 | | 8.61500333 | -9.5486381 | 1.05E-05 | 0.0002209 | 3.48949951 |
| Slc9a3r1 | 1438115_a_ -1.4283396 | | 9.77644555 | -9.368762 | 1.21E-05 | 0.0002441 | 3.33256295 |
| Cfp | 1452279_at -1.4260223 | | 8.91796815 | -7.1937315 | 8.45E-05 | 0.00093951 | 1.20511443 |
| Ifitm3 | 1423754_at -1.4257194 | | 12.8091708 | -17.158319 | 1.09E-07 | 9.08E-06 | 8.43402133 |
| Iqgap1 | 1434998_at -1.4242854 | | 10.872937 | -10.576762 | 4.82E-06 | 0.00012753 | 4.33973429 |
| Gdap10 | 1420342_at -1.4204218 | | 9.61649361 | -17.035244 | 1.16E-07 | 9.28E-06 | 8.37304298 |
| Tnfaip2 | 1416273_at -1.4168442 | | 10.1211056 | -10.694202 | 4.43E-06 | 0.00011984 | 4.43211059 |
| Psat1 | 1454607_s_a-1.4158988 | | 7.48588179 | -15.981816 | 1.92E-07 | 1.36E-05 | 7.83167226 |
| Ccl3 | 1419561_at -1.4145008 | | 8.01436649 | -22.389457 | 1.30E-08 | 2.15E-06 | 10.6673851 |
| Dhx58 | 1451426_at -1.4095753 | | 8.07795715 | -15.660653 | 2.26E-07 | 1.48E-05 | 7.65931745 |
| Klf6 | 1433508_at -1.4082576 | | 10.0180182 | -16.707952 | 1.35E-07 | 1.04E-05 | 8.20863028 |
| Cyp4f18 | 1419219_at -1.4075797 | | 8.23076503 | -6.6391289 | 0.00014913 | 0.00143504 | 0.58376432 |
| Csf1r | 1419872_at -1.4068984 | | 11.5210193 | -8.0851135 | 3.62E-05 | 0.00051688 | 2.13229157 |
| Cd24a | 1437502_x_a-1.4058932 | | 8.29520598 | -3.3009171 | 0.01055725 | 0.03578487 | -4.0072236 |
| Wipf1 | 1436953_at -1.4034691 | | 9.27833367 | -10.982084 | 3.61E-06 | 0.00010522 | 4.65471957 |
| S100a6 | 1421375_a_ -1.403291 | | 10.8574646 | -15.323511 | 2.68E-07 | 1.64E-05 | 7.47447934 |
| Tmem173 | 1427911_at -1.3949628 | | 8.99790083 | -10.21435 | 6.29E-06 | 0.00015258 | 4.04873586 |
| Ciita | 1421210_at -1.3928401 | | 8.35345135 | -8.3597951 | 2.83E-05 | 0.00044044 | 2.40172474 |
| Dbnl | 1460334_at -1.3924507 | | 10.0281651 | -13.298855 | 8.18E-07 | 3.63E-05 | 6.27100891 |
| Akna | 1452393_at -1.3922685 | | 7.99563962 | -7.5779903 | 5.81E-05 | 0.00071509 | 1.61510897 |
| Pabpc1 | 1418883_a_ -1.3909898 | | 12.8807716 | -11.750555 | 2.14E-06 | 7.20E-05 | 5.22371474 |
| Ccna2 | 1417911_at -1.3898424 | | 8.42879401 | -12.032067 | 1.78E-06 | 6.36E-05 | 5.42352026 |
| Oasl1 | 1424339_at -1.389218 | | 8.87905615 | -16.497776 | 1.49E-07 | 1.12E-05 | 8.1012831 |
| Rab7b | 1424987_at -1.3873728 | | 8.9419163 | -8.4351685 | 2.65E-05 | 0.00042254 | 2.47440086 |
| Ccnd1 | 1448698_at -1.3871827 | | 10.7935928 | -17.158019 | 1.09E-07 | 9.08E-06 | 8.43387307 |
| Lasp1 | 1439264_x_a-1.3858178 | | 9.56686931 | -14.305524 | 4.61E-07 | 2.42E-05 | 6.89048545 |
| Ly75 | 1449328_at -1.3828713 | | 6.63795114 | -10.556598 | 4.89E-06 | 0.00012878 | 4.3237804 |
| Rnf149 | 1429321_at -1.3828333 | | 9.17668945 | -10.330909 | 5.77E-06 | 0.00014345 | 4.14332148 |
| Fbn1 | 1425896_a_ -1.3800312 | | 9.46568434 | -12.474175 | 1.35E-06 | 5.24E-05 | 5.7285839 |
| Smc4 | 1427275_at -1.3794938 | | 8.34824867 | -12.906226 | 1.03E-06 | 4.34E-05 | 6.0169207 |
| Capza1 | 1452038_at -1.3790839 | | 10.9006062 | -11.076951 | 3.38E-06 | 0.00010099 | 4.72691231 |
| Igsf6 | 1421408_at -1.3783068 | | 7.92062915 | -7.8929821 | 4.32E-05 | 0.00058407 | 1.93943651 |
| Cd47 | 1449507_a_ -1.3778362 | | 11.6534438 | -21.098964 | 2.09E-08 | 2.84E-06 | 10.1737591 |
| S100a10 | 1456642_x_a-1.3773357 | | 10.2222326 | -17.075193 | 1.14E-07 | 9.27E-06 | 8.39288618 |
| Slc2a1 | 1434773_a_ -1.3757914 | | 9.34371127 | -18.710193 | 5.48E-08 | 5.68E-06 | 9.16575254 |
| Ppp1r18 | 1431299_a_ -1.37413 | | 10.2188241 | -9.8049317 | 8.59E-06 | 0.00019107 | 3.70873531 |
| Rhog | 1422572_at -1.3738491 | | 10.0848341 | -10.0199 | 7.29E-06 | 0.00017131 | 3.88878607 |
| Emp1 | 1416529_at -1.373668 | | 10.6852731 | -15.595971 | 2.33E-07 | 1.50E-05 | 7.62416993 |
| Inpp5b | 1451330_a_ -1.3713508 | | 9.15561029 | -13.662169 | 6.62E-07 | 3.12E-05 | 6.49972392 |
| NA | 1422660_at -1.3699786 | | 11.2543423 | -14.998126 | 3.18E-07 | 1.87E-05 | 7.29215002 |
| Actr3 | 1434968_a_ -1.3688939 | | 11.7737729 | -16.496872 | 1.49E-07 | 1.12E-05 | 8.10081846 |
| Mcm3 | 1420028_s_a-1.3665175 | | 8.04374564 | -11.682926 | 2.24E-06 | 7.41E-05 | 5.17504716 |
| Rcn1 | 1417090_at -1.3658794 | | 9.67827545 | -12.029212 | 1.79E-06 | 6.36E-05 | 5.42151587 |
| Msn | 1450379_at -1.3644132 | | 10.0868924 | -8.9029657 | 1.78E-05 | 0.00031624 | 2.91385204 |
| C2 | 1416051_at -1.3638174 | | 9.0884207 | -9.4507286 | 1.13E-05 | 0.00023301 | 3.40439595 |
| Uhrf1 | 1415810_at -1.3625103 | | 7.84379648 | -9.1220364 | 1.48E-05 | 0.00027919 | 3.11304 |
| Mcm5 | 1415945_at -1.3624451 | | 7.82398343 | -9.7188071 | 9.18E-06 | 0.00020074 | 3.63562678 |
| H2afz | 1438091_a_ -1.3606997 | | 10.2039309 | -12.981514 | 9.88E-07 | 4.15E-05 | 6.06621662 |
| Arl4c | 1436512_at -1.3598359 | | 9.34310433 | -11.063809 | 3.41E-06 | 0.00010153 | 4.71694558 |
| Cfl1 | 1448346_at -1.3594674 | | 11.4492129 | -9.9068562 | 7.94E-06 | 0.00018108 | 3.79453249 |
| Bcl3 | 1418133_at -1.3588357 | | 8.74699452 | -10.730456 | 4.32E-06 | 0.00011799 | 4.46044246 |
| Tifa | 1426501_a_ -1.3580097 | | 8.97127609 | -12.720394 | 1.16E-06 | 4.68E-05 | 5.8940528 |
| Fscn1 | 1416514_a_ -1.357072 | | 9.37722904 | -8.0798053 | 3.64E-05 | 0.0005184 | 2.12701265 |
| Asns | 1433966_x_a-1.3568639 | | 7.7409288 | -8.7362801 | 2.04E-05 | 0.00034929 | 2.75951485 |
| Eif1a | 1424343_a_ -1.356409 | | 8.97886815 | -15.990427 | 1.91E-07 | 1.36E-05 | 7.83624502 |
| Cmtm7 | 1460253_at -1.3558187 | | 9.50627347 | -8.204049 | 3.26E-05 | 0.00048343 | 2.24984767 |
| Socs1 | 1450446_a_ -1.355608 | | 8.3042411 | -11.055325 | 3.43E-06 | 0.00010198 | 4.71050542 |
| Lasp1 | 1455470_x_a-1.3554617 | | 9.74817042 | -12.082645 | 1.73E-06 | 6.26E-05 | 5.45895232 |
| Ctsh | 1418365_at -1.3548672 | | 10.5152886 | -6.713018 | 0.00013799 | 0.00135192 | 0.66865155 |
| Pilra | 1427327_at -1.3545261 | | 7.92463092 | -9.213525 | 1.37E-05 | 0.00026503 | 3.19502657 |
| Otulinl | 1435375_at -1.3524129 | | 8.55825164 | -8.5337214 | 2.43E-05 | 0.00039667 | 2.56862914 |
| Kcnn4 | 1435945_a_ -1.351551 | | 9.20028386 | -14.802236 | 3.52E-07 | 2.00E-05 | 7.18045647 |
| Cd40 | 1460415_a_ -1.3500605 | | 8.30813728 | -10.205684 | 6.33E-06 | 0.00015308 | 4.04166556 |
| Lgmn | 1448883_at -1.349372 | | 10.0640775 | -9.9416543 | 7.73E-06 | 0.00017785 | 3.82364698 |
| Rbm43 | 1451321_a_ -1.3486387 | | 9.10138277 | -13.429937 | 7.57E-07 | 3.43E-05 | 6.35422234 |
| Marcks | 1456700_x_a-1.3484175 | | 9.74594333 | -9.0771724 | 1.54E-05 | 0.00028685 | 3.07257982 |
| Cnn3 | 1455570_x_a-1.3475335 | | 9.99277969 | -18.396287 | 6.27E-08 | 6.30E-06 | 9.02304949 |
| Cotl1 | 1436236_x_a-1.347522 | | 9.27290274 | -4.7994072 | 0.00128555 | 0.0070423 | -1.7602019 |
| Cd40 | 1449473_s_a-1.3461074 | | 7.81261602 | -16.281022 | 1.66E-07 | 1.21E-05 | 7.98908949 |
| Cd300c2 | 1428018_a_ -1.3439143 | | 9.27958729 | -9.999011 | 7.40E-06 | 0.00017297 | 3.87144016 |
| Ccl4 | 1421578_at -1.3432944 | | 9.428448 | -12.134307 | 1.67E-06 | 6.12E-05 | 5.49499924 |
| NA | 1460351_at -1.3425747 | | 11.8144591 | -12.845913 | 1.07E-06 | 4.46E-05 | 5.97723062 |
| Ogfr | 1422511_a_ -1.3414709 | | 10.8669516 | -18.657519 | 5.60E-08 | 5.78E-06 | 9.14198569 |

| Parp3 | 1451969_s_a-1.3399858 | 10.9758091 | -14.35067 | 4.50E-07 | 2.40E-05 | 6.91725084 |
| --- | --- | --- | --- | --- | --- | --- |
| Tagln2 | 1439407_x_a-1.3399674 | 10.737459 | -15.898673 | 2.00E-07 | 1.39E-05 | 7.78739421 |
| Cdca5 | 1416802_a_ -1.3391489 | 7.76681494 | -10.744613 | 4.27E-06 | 0.00011728 | 4.47148174 |
| Mtpn | 1437457_a_ -1.3360924 | 9.34704836 | -7.658974 | 5.38E-05 | 0.00067825 | 1.69948092 |
| Naaa | 1452068_at -1.3345787 | 8.32801 | -9.1544346 | 1.44E-05 | 0.00027496 | 3.14215303 |
| Tlr2 | 1419132_at -1.3339863 | 8.92656252 | -14.527646 | 4.08E-07 | 2.24E-05 | 7.02137528 |
| Esyt1 | 1451099_at -1.3330489 | 9.43471606 | -8.3972085 | 2.74E-05 | 0.00043237 | 2.43786588 |
| Csf1r | 1419873_s_a-1.3328191 | 10.3315034 | -5.9001299 | 0.00033597 | 0.00260977 | -0.3029887 |
| Lsp1 | 1417756_a_ -1.3294873 | 10.8794447 | -7.5361257 | 6.05E-05 | 0.0007345 | 1.57121976 |
| NA | 1416923_a_ -1.3254787 | 10.223798 | -4.4675635 | 0.00199273 | 0.00990039 | -2.2329641 |
| Marcks | 1415971_at -1.3252486 | 10.0913714 | -8.6066521 | 2.28E-05 | 0.00037911 | 2.63778477 |
| Ccl6 | 1417266_at -1.3247904 | 10.3287435 | -7.0305536 | 9.95E-05 | 0.00105881 | 1.02602673 |
| Cdt1 | 1424143_a_ -1.3223537 | 8.63737834 | -14.660003 | 3.80E-07 | 2.12E-05 | 7.09842625 |
| Slc2a3 | 1455898_x_a-1.3205001 | 8.27134605 | -13.178504 | 8.78E-07 | 3.79E-05 | 6.19390422 |
| Actr3 | 1426392_a_ -1.3204241 | 10.4933062 | -15.10838 | 3.00E-07 | 1.80E-05 | 7.35437314 |
| Slc39a6 | 1424674_at -1.3184196 | 8.57705312 | -12.848426 | 1.07E-06 | 4.46E-05 | 5.97888773 |
| Cd86 | 1449858_at -1.3180359 | 7.89868526 | -6.9076325 | 0.0001128 | 0.00116187 | 0.88909429 |
| Racgap1 | 1421546_a_ -1.3179604 | 7.53343441 | -11.045608 | 3.46E-06 | 0.0001024 | 4.70312351 |
| Casp7 | 1448659_at -1.3173743 | 9.24747899 | -17.132299 | 1.11E-07 | 9.16E-06 | 8.4211674 |
| Tpm4 | 1433883_at -1.3168953 | 11.5359639 | -17.31805 | 1.01E-07 | 8.76E-06 | 8.51248452 |
| NA | 1448500_a_ -1.3164787 | 8.84920534 | -10.546433 | 4.93E-06 | 0.00012916 | 4.31572778 |
| Psma5 | 1434356_a_ -1.3150338 | 11.4378813 | -16.099483 | 1.81E-07 | 1.31E-05 | 7.89393584 |
| Clec4a2 | 1422013_at -1.3139217 | 8.27255402 | -7.9492024 | 4.10E-05 | 0.00056464 | 1.99625098 |
| Plat | 1415806_at -1.3122797 | 9.56967364 | -14.278003 | 4.68E-07 | 2.44E-05 | 6.87412748 |
| Bid | 1417045_at -1.3118967 | 7.96175519 | -13.080234 | 9.31E-07 | 3.94E-05 | 6.13043781 |
| Fasl | 1449235_at -1.3107595 | 7.7024004 | -13.429606 | 7.57E-07 | 3.43E-05 | 6.35401326 |
| Inpp5d | 1418110_a_ -1.3066644 | 9.01247271 | -8.4094879 | 2.71E-05 | 0.00042937 | 2.4496991 |
| Gimap6 | 1427891_at -1.3058231 | 9.39290731 | -11.722317 | 2.18E-06 | 7.28E-05 | 5.20342562 |
| Smc4 | 1452197_at -1.3039617 | 9.13545132 | -7.8483242 | 4.50E-05 | 0.00060068 | 1.89407837 |
| Cd37 | 1419206_at -1.3034889 | 8.75667977 | -5.7445022 | 0.00040212 | 0.00294803 | -0.4988367 |
| Trim25 | 1419879_s_a-1.3031212 | 9.89722874 | -17.414875 | 9.71E-08 | 8.47E-06 | 8.55968035 |
| Dusp2 | 1450698_at -1.3017438 | 9.11130147 | -18.188612 | 6.87E-08 | 6.60E-06 | 8.92721005 |
| Hnrnpf | 1437099_x_a-1.3003343 | 10.7804756 | -17.441078 | 9.59E-08 | 8.40E-06 | 8.57240532 |
| Ptk2b | 1434653_at -1.2998612 | 8.6875233 | -6.8082803 | 0.00012496 | 0.00125571 | 0.77712065 |
| Nup210 | 1417585_at -1.2982415 | 8.71468392 | -11.544903 | 2.46E-06 | 7.97E-05 | 5.07490147 |
| Vcam1 | 1451314_a_ -1.2935435 | 9.53463461 | -11.683716 | 2.24E-06 | 7.41E-05 | 5.17561693 |
| Csf2ra | 1420703_at -1.2928286 | 9.1608145 | -7.0723518 | 9.54E-05 | 0.00102808 | 1.07219032 |
| Ptpre | 1418540_a_ -1.2911375 | 8.62739745 | -9.4078 | 1.17E-05 | 0.0002389 | 3.36684192 |
| Cnn3 | 1456380_x_a-1.2896503 | 7.37625007 | -13.810914 | 6.08E-07 | 2.92E-05 | 6.59165323 |
| Frmd4b | 1438169_a_ -1.2892216 | 9.44971381 | -8.6869837 | 2.13E-05 | 0.00036024 | 2.71339972 |
| Rgs19 | 1417786_a_ -1.2882975 | 8.87762505 | -8.8169402 | 1.91E-05 | 0.00033284 | 2.83450416 |
| Ccnd1 | 1417419_at -1.2879424 | 9.24511638 | -9.4684653 | 1.12E-05 | 0.00023108 | 3.41986918 |
| Arf6 | 1434312_at -1.2871327 | 8.22757458 | -12.061299 | 1.75E-06 | 6.29E-05 | 5.44401548 |
| Prkcb | 1460419_a_ -1.2859688 | 8.6111537 | -8.6966924 | 2.11E-05 | 0.00035779 | 2.72249913 |
| NA | 1452426_x_a-1.2848517 | 8.26560578 | -10.989645 | 3.59E-06 | 0.00010511 | 4.6604941 |
| Gnb1 | 1417432_a_ -1.2844062 | 9.70675209 | -12.199096 | 1.60E-06 | 5.96E-05 | 5.5400018 |
| Cnn3 | 1436759_x_a-1.2841723 | 10.1636256 | -15.962728 | 1.94E-07 | 1.36E-05 | 7.82152799 |
| Rai14 | 1417400_at -1.2835786 | 7.9330374 | -15.813761 | 2.09E-07 | 1.41E-05 | 7.74192905 |
| Ptger4 | 1424208_at -1.2830507 | 8.81051351 | -6.9184679 | 0.00011156 | 0.00115267 | 0.90123572 |
| Erdr1 | 1452406_x_a-1.2817395 | 11.7764914 | -5.8628796 | 0.00035064 | 0.0026833 | -0.3495691 |
| Tnfrsf1b | 1418099_at -1.2812269 | 8.61596926 | -8.2534834 | 3.11E-05 | 0.00047002 | 2.2983052 |
| Rgs19 | 1434940_x_a-1.2793531 | 7.46254674 | -13.082493 | 9.30E-07 | 3.94E-05 | 6.13190195 |
| Stat2 | 1450403_at -1.2757522 | 8.47576014 | -11.495774 | 2.54E-06 | 8.17E-05 | 5.03898591 |
| Hnrnpf | 1456664_x_a-1.2757496 | 11.1353798 | -15.360277 | 2.63E-07 | 1.63E-05 | 7.49483567 |
| NA | 1449277_at -1.2749468 | 8.98097458 | -9.970488 | 7.56E-06 | 0.00017545 | 3.84770333 |
| Bpgm | 1448119_at -1.2737157 | 9.63760788 | -2.6087628 | 0.03069516 | 0.08174568 | -5.110407 |
| Actr2 | 1452587_at -1.2722471 | 11.7194963 | -12.491428 | 1.33E-06 | 5.21E-05 | 5.7402795 |
| Irf5 | 1460231_at -1.2692665 | 8.41220288 | -6.2582092 | 0.00022479 | 0.00193495 | 0.13542471 |
| Ptgs2 | 1417262_at -1.268833 | 7.66546332 | -11.088687 | 3.36E-06 | 0.00010037 | 4.73580341 |
| Il10ra | 1448731_at -1.2677569 | 7.90294096 | -8.9444049 | 1.72E-05 | 0.00030811 | 2.95184502 |
| Rhoc | 1435394_s_a-1.2664846 | 11.0960027 | -15.56067 | 2.37E-07 | 1.52E-05 | 7.60492563 |
| Cmip | 1453015_at -1.2640825 | 10.9129966 | -8.5337655 | 2.43E-05 | 0.00039667 | 2.56867117 |
| M6pr | 1416385_a_ -1.2631061 | 11.1819704 | -9.8864241 | 8.07E-06 | 0.00018304 | 3.77739565 |
| Nipsnap3b | 1448967_at -1.261952 | 8.64518462 | -13.411288 | 7.65E-07 | 3.45E-05 | 6.34243193 |
| Rcan1 | 1416600_a_ -1.2580457 | 10.7856856 | -11.577221 | 2.41E-06 | 7.81E-05 | 5.09845012 |
| Atp6ap2 | 1439456_x_a-1.2571382 | 10.5592121 | -11.134498 | 3.25E-06 | 9.77E-05 | 4.77042934 |
| Gm2a | 1448241_at -1.2570614 | 10.1985032 | -5.0756376 | 0.00090353 | 0.00536675 | -1.3785326 |
| Cadm1 | 1417376_a_ -1.2542406 | 7.9290466 | -6.3832039 | 0.0001961 | 0.00174871 | 0.28455134 |
| Ptpn6 | 1456694_x_a-1.2532333 | 9.96884041 | -10.297896 | 5.92E-06 | 0.00014595 | 4.11662928 |
| Ptpn18 | 1419125_at -1.251042 | 9.01318421 | -8.0238057 | 3.83E-05 | 0.00053876 | 2.07115269 |
| Mfsd1 | 1424129_at -1.2492584 | 9.15000223 | -14.543579 | 4.05E-07 | 2.23E-05 | 7.0306874 |
| Tmem51 | 1424383_at -1.2488247 | 8.88572372 | -9.4343482 | 1.15E-05 | 0.00023451 | 3.39008369 |
| Plek | 1417523_at -1.2459523 | 8.71689347 | -14.265962 | 4.71E-07 | 2.45E-05 | 6.86696075 |
| Dbf4 | 1418334_at -1.2429286 | 7.40730482 | -11.32367 | 2.85E-06 | 8.90E-05 | 4.91203811 |
| Pclaf | 1419152_at -1.2422797 | 7.46073969 | -10.885342 | 3.87E-06 | 0.0001096 | 4.58051111 |
| Abi1 | 1423177_a_ -1.2400811 | 9.66823552 | -9.4145168 | 1.17E-05 | 0.00023784 | 3.37272747 |
| F2r | 1437308_s_a-1.2399681 | 11.2144651 | -9.8500782 | 8.30E-06 | 0.0001871 | 3.74683451 |
| Crlf2 | 1418097_a_ -1.2384063 | 9.54873074 | -10.001603 | 7.39E-06 | 0.00017283 | 3.87359393 |
| Rap1b | 1455349_at -1.23839 | 8.794441 | -16.61659 | 1.41E-07 | 1.08E-05 | 8.16213974 |
| Jak2 | 1421066_at -1.2377292 | 9.44315885 | -15.602351 | 2.32E-07 | 1.50E-05 | 7.62764334 |
| Tgm2 | 1437277_x_a-1.236862 | 12.6529209 | -11.732691 | 2.17E-06 | 7.24E-05 | 5.21088453 |
| Rhoc | 1448605_at -1.2364508 | 9.12986873 | -15.315237 | 2.69E-07 | 1.65E-05 | 7.4698918 |
| Ccl9 | 1448898_at -1.2362575 | 8.77002037 | -7.0902674 | 9.37E-05 | 0.00101648 | 1.09191551 |
| Cmtm3 | 1448316_at -1.2342595 | 8.76038891 | -9.0194536 | 1.61E-05 | 0.00029534 | 3.02027665 |
| Msn | 1421814_at -1.2340075 | 11.894817 | -13.603301 | 6.85E-07 | 3.20E-05 | 6.4630708 |

| Ptprj | 1455030_at | -1.2323888 | 8.71770782 | -5.6801899 | 0.00043353 | 0.00310823 | -0.5807275 |
| --- | --- | --- | --- | --- | --- | --- | --- |
| Gna13 | 1422556_at | -1.2300136 | 8.83034628 | -10.912882 | 3.79E-06 | 0.00010855 | 4.60169773 |
| Apobec1 | 1451755_a_ | -1.2295124 | 8.82761712 | -8.3650672 | 2.82E-05 | 0.00044026 | 2.40682555 |
| Apaf1 | 1452870_at | -1.2294274 | 8.59756133 | -17.029074 | 1.16E-07 | 9.28E-06 | 8.36997364 |
| Manf | 1428112_at | -1.227956 | 9.94502925 | -13.887055 | 5.82E-07 | 2.83E-05 | 6.6383358 |
| Pmaip1 | 1418203_at | -1.2276189 | 7.33583629 | -10.318033 | 5.83E-06 | 0.00014451 | 4.13292039 |
| Cd300lf | 1427994_at | -1.2269471 | 8.23579258 | -9.9224042 | 7.85E-06 | 0.00017931 | 3.80755217 |
| Cflar | 1424996_at | -1.2261952 | 9.23422268 | -12.032276 | 1.78E-06 | 6.36E-05 | 5.42366703 |
| Ect2 | 1419513_a_ | -1.2260507 | 7.26140761 | -13.557401 | 7.03E-07 | 3.27E-05 | 6.43438434 |
| Pomp | 1416979_at | -1.2244578 | 11.8286377 | -18.258382 | 6.66E-08 | 6.49E-06 | 8.95953704 |
| Tgm2 | 1455900_x_a-1.2237143 | | 11.5588964 | -10.986714 | 3.60E-06 | 0.00010519 | 4.6582565 |
| Pcna | 1417947_at -1.2232354 | | 10.8944415 | -15.981788 | 1.92E-07 | 1.36E-05 | 7.83165749 |
| Zap70 | 1422701_at -1.2230655 | | 8.19838514 | -8.375505 | 2.80E-05 | 0.00043853 | 2.4169164 |
| Man2b1 | 1416340_a_ -1.2229213 | | 10.7870078 | -6.0220452 | 0.00029248 | 0.00236002 | -0.1518306 |
| Cp | 1455393_at -1.2195613 | | 10.8478176 | -8.0734074 | 3.66E-05 | 0.00052077 | 2.12064644 |
| NA | 1435792_at -1.2194588 | | 7.65946568 | -9.2879348 | 1.29E-05 | 0.00025488 | 3.26119759 |
| Cd83 | 1416111_at -1.2189426 | | 8.27563443 | -6.3855811 | 0.0001956 | 0.00174658 | 0.28736818 |
| Pla2g4a | 1448558_a_ -1.2178498 | | 8.48844467 | -13.575263 | 6.96E-07 | 3.24E-05 | 6.44555868 |
| Pfn1 | 1449018_at -1.2177807 | | 11.9073692 | -11.83461 | 2.03E-06 | 6.94E-05 | 5.28383914 |
| Rrm2 | 1416120_at -1.2175344 | | 7.79736944 | -17.026929 | 1.16E-07 | 9.28E-06 | 8.36890637 |
| Fam117a | 1433639_at -1.2171727 | | 8.98459692 | -5.0904681 | 0.00088685 | 0.00528711 | -1.3583472 |
| Gpr31b | 1452408_at -1.2160601 | | 7.33291784 | -9.4429595 | 1.14E-05 | 0.00023354 | 3.39761049 |
| Myof | 1427318_s_a-1.2158239 | | 8.72211413 | -7.9461808 | 4.11E-05 | 0.00056587 | 1.99320554 |
| Tpd52 | 1419493_a_ -1.2154702 | | 10.2850328 | -9.0977743 | 1.51E-05 | 0.00028318 | 3.09118046 |
| Gns | 1433488_x_a-1.2141975 | | 11.1296288 | -18.775743 | 5.33E-08 | 5.55E-06 | 9.19522954 |
| Cd300ld | 1424832_at -1.2110084 | | 8.98704156 | -7.3403927 | 7.31E-05 | 0.00084225 | 1.36351145 |
| Gm9706 | 1453939_x_a-1.210433 | | 8.06284831 | -9.2890164 | 1.29E-05 | 0.00025488 | 3.26215609 |
| Gusb | 1448124_at -1.2102528 | | 9.27485894 | -6.7505815 | 0.00013268 | 0.00131233 | 0.71155287 |
| Nfkbia | 1449731_s_a-1.2096411 | | 10.6582393 | -9.3334679 | 1.25E-05 | 0.00024826 | 3.30146575 |
| NA | AFFX-18SRN -1.209636 | | 9.11681903 | -1.8176588 | 0.10592429 | 0.20282043 | -6.3282947 |
| Arhgap9 | 1436097_x_a-1.2092868 | | 8.75663455 | -7.3195182 | 7.46E-05 | 0.00085488 | 1.34111259 |
| Anxa4 | 1424176_a_ -1.2090189 | | 8.7887984 | -12.622839 | 1.23E-06 | 4.89E-05 | 5.82886104 |
| Myh9 | 1417472_at -1.2087937 | | 10.4915868 | -5.9663993 | 0.00031151 | 0.00247399 | -0.220579 |
| Klf6 | 1427742_a_ -1.2083847 | | 9.84315069 | -14.435503 | 4.29E-07 | 2.31E-05 | 6.96732076 |
| Birc3 | 1421392_a_ -1.2072344 | | 8.90674419 | -8.9555178 | 1.70E-05 | 0.00030672 | 2.96200861 |
| Rab43 | 1425333_at -1.2061399 | | 9.20032381 | -7.405715 | 6.86E-05 | 0.00080454 | 1.43329397 |
| Sdc4 | 1417654_at -1.2059917 | | 9.66456754 | -10.745429 | 4.27E-06 | 0.00011728 | 4.47211773 |
| Pdia3 | 1423423_at -1.2052753 | | 11.3389928 | -10.611056 | 4.70E-06 | 0.00012558 | 4.36680437 |
| Ccr1 | 1419609_at -1.2052379 | | 8.20830404 | -11.735106 | 2.17E-06 | 7.24E-05 | 5.21262002 |
| Sdc3 | 1450027_at -1.2042653 | | 10.5645617 | -6.8779773 | 0.00011629 | 0.00119126 | 0.85579398 |
| Ifi27l2a | 1426278_at -1.2027784 | | 11.5838229 | -8.7025589 | 2.10E-05 | 0.00035653 | 2.72799332 |
| NA | 1422302_s_a-1.2023705 | | 12.8979144 | -11.63853 | 2.31E-06 | 7.59E-05 | 5.14295505 |
| Cnn3 | 1426724_at -1.2005731 | | 10.1112529 | -13.524961 | 7.16E-07 | 3.30E-05 | 6.41405275 |
| Ncf1 | 1451767_at -1.1986567 | | 8.45132453 | -7.7158976 | 5.10E-05 | 0.00065216 | 1.75837315 |
| Blvrb | 1451386_at -1.1980263 | | 9.50722783 | -12.658193 | 1.20E-06 | 4.82E-05 | 5.85254192 |
| Osmr | 1418674_at -1.1964428 | | 9.7790002 | -7.8190168 | 4.63E-05 | 0.00061176 | 1.86420095 |
| Tmtc4 | 1428113_at -1.194571 | | 9.50585803 | -17.460366 | 9.51E-08 | 8.36E-06 | 8.5817594 |
| Leprotl1 | 1423552_at -1.1931717 | | 9.32769774 | -12.507984 | 1.32E-06 | 5.16E-05 | 5.75148874 |
| Grina | 1436297_a_ -1.1928125 | | 10.2492744 | -11.950551 | 1.88E-06 | 6.59E-05 | 5.36611854 |
| Icos | 1421931_at -1.1926531 | | 7.97442565 | -9.4532179 | 1.13E-05 | 0.00023301 | 3.40656912 |
| Gclm | 1418627_at -1.1919588 | | 10.1440616 | -6.8198873 | 0.00012347 | 0.00124589 | 0.79026254 |
| Arl6ip1 | 1451131_at -1.1905446 | | 9.72625654 | -9.9888359 | 7.46E-06 | 0.00017414 | 3.86297933 |
| F2r | 1450852_s_a-1.1903581 | | 10.5868841 | -10.261858 | 6.08E-06 | 0.00014903 | 4.08740337 |
| Ccl9 | 1417936_at -1.1902827 | | 10.2314824 | -5.8606999 | 0.00035152 | 0.00268822 | -0.3523005 |
| NA | 1422188_s_a-1.1881167 | | 7.31547124 | -5.8173911 | 0.00036951 | 0.00278917 | -0.4067038 |
| NA | 1435872_at -1.18514 | | 9.45658085 | -14.297975 | 4.63E-07 | 2.42E-05 | 6.88600111 |
| Ripk1 | 1449485_at -1.1838126 | | 9.09964905 | -17.052535 | 1.15E-07 | 9.27E-06 | 8.38163731 |
| Atp6ap2 | 1437688_x_a-1.1835392 | | 11.0200959 | -10.483541 | 5.16E-06 | 0.00013297 | 4.26574725 |
| Marcks | 1456028_x_a-1.1813288 | | 10.2495612 | -8.1145362 | 3.53E-05 | 0.00050772 | 2.16150163 |
| Trim25 | 1425974_a_ -1.1797299 | | 10.223481 | -12.769755 | 1.12E-06 | 4.59E-05 | 5.9268563 |
| Pim1 | 1435458_at -1.1795849 | | 9.65655884 | -14.155185 | 5.01E-07 | 2.55E-05 | 6.80074632 |
| Rmdn3 | 1428196_a_ -1.1784886 | | 8.58912875 | -15.775413 | 2.13E-07 | 1.43E-05 | 7.72131448 |
| Cap1 | 1417462_at -1.1779109 | | 8.40629603 | -6.7406675 | 0.00013406 | 0.00132026 | 0.70024657 |
| Pik3cd | 1453281_at -1.176373 | | 8.12206594 | -9.2292121 | 1.36E-05 | 0.00026343 | 3.20901463 |
| Cdk1 | 1448314_at -1.1757369 | | 8.68915911 | -6.8906056 | 0.00011479 | 0.00117866 | 0.86998715 |
| Cd47 | 1419554_at -1.1750967 | | 10.3838612 | -10.202862 | 6.35E-06 | 0.00015324 | 4.03936219 |
| Phc2 | 1437239_x_a-1.1741915 | | 10.2650103 | -13.689437 | 6.52E-07 | 3.09E-05 | 6.5166496 |
| Slc2a1 | 1426599_a_ -1.1731087 | | 8.94174582 | -12.265916 | 1.54E-06 | 5.74E-05 | 5.58617892 |
| Peli1 | 1417372_a_ -1.1719039 | | 8.5673929 | -11.735414 | 2.17E-06 | 7.24E-05 | 5.21284164 |
| NA | 1421811_at -1.1708371 | | 8.40326951 | -7.8617599 | 4.45E-05 | 0.00059589 | 1.90774609 |
| Tor4a | 1424829_at -1.1685512 | | 9.23333606 | -7.0294915 | 9.96E-05 | 0.00105945 | 1.02485117 |
| Wipf1 | 1436954_at -1.166772 | | 8.77032017 | -7.5910185 | 5.74E-05 | 0.00070855 | 1.62872919 |
| Twf2 | 1439440_x_a-1.1665402 | | 10.7002082 | -14.474476 | 4.20E-07 | 2.29E-05 | 6.9902253 |
| Pla1a | 1417785_at -1.1655202 | | 9.55364279 | -10.569781 | 4.85E-06 | 0.00012788 | 4.33421374 |
| Cx3cl1 | 1415803_at -1.1652628 | | 8.7403861 | -11.743823 | 2.15E-06 | 7.22E-05 | 5.21888183 |
| Cdkn1a | 1424638_at -1.1625394 | | 10.3368171 | -7.7842864 | 4.78E-05 | 0.00062392 | 1.82868092 |
| NA | 1416869_x_a-1.1624556 | | 8.86578545 | -12.903201 | 1.04E-06 | 4.34E-05 | 6.01493462 |
| Cd180 | 1421547_at -1.1616933 | | 9.00381243 | -5.288524 | 0.00069364 | 0.00440612 | -1.0917588 |
| Frmd4b | 1452123_s_a-1.1612872 | | 8.84468636 | -6.9129854 | 0.00011219 | 0.00115813 | 0.89509404 |
| Marcks | 1415973_at -1.1598168 | | 9.77217239 | -6.4172126 | 0.00018901 | 0.0017032 | 0.32478299 |
| Eif4e2 | 1435803_a_ -1.1587625 | | 8.60667877 | -17.168507 | 1.09E-07 | 9.08E-06 | 8.43904836 |
| Irf9 | 1421322_a_ -1.1582784 | | 8.92203407 | -10.883313 | 3.87E-06 | 0.0001096 | 4.57894859 |
| Ripk3 | 1448449_at -1.1564482 | | 8.08485128 | -10.113266 | 6.79E-06 | 0.00016267 | 3.96592732 |
| Mmp14 | 1416572_at -1.1562284 | | 8.50731929 | -5.2786404 | 0.0007021 | 0.00444073 | -1.1049314 |
| Gm2a | 1416188_at -1.1561955 | | 9.46777141 | -4.2248166 | 0.00277431 | 0.0127608 | -2.5884493 |

| Scpep1 | 1426555_at | -1.1559144 | 10.2876461 | -6.217487 | 0.00023511 | 0.00200177 | 0.08640919 |
| --- | --- | --- | --- | --- | --- | --- | --- |
| Tmsb4x | 1415906_at | -1.1549044 | 13.7528955 | -15.519177 | 2.42E-07 | 1.54E-05 | 7.58224915 |
| NA | 1421653_a_ | -1.1539208 | 9.44195527 | -3.6397772 | 0.00637941 | 0.02414491 | -3.476204 |
| Clec4n | 1419627_s_a-1.1538763 | | 8.33985921 | -16.887437 | 1.24E-07 | 9.77E-06 | 8.29920285 |
| Hif1a | 1416035_at -1.1535085 | | 9.32479485 | -17.838983 | 8.02E-08 | 7.36E-06 | 8.76321389 |
| NA | 1450639_at -1.1513832 | | 9.19769961 | -6.9394697 | 0.00010919 | 0.00113128 | 0.9247297 |
| Sec61a1 | 1416189_a_ -1.1506713 | | 10.5068352 | -18.980872 | 4.88E-08 | 5.18E-06 | 9.28676747 |
| NA | 1455725_a_ -1.1479677 | | 11.5943563 | -17.057708 | 1.15E-07 | 9.27E-06 | 8.38420696 |
| NA | 1429381_x_a-1.1468517 | | 8.95641205 | -4.1350244 | 0.00314243 | 0.01407098 | -2.7219451 |
| Pip4k2a | 1449404_at -1.14577 | | 9.28333844 | -8.8613747 | 1.84E-05 | 0.00032368 | 2.87557015 |
| Itga6 | 1422445_at -1.1454746 | | 10.3428743 | -12.793053 | 1.11E-06 | 4.55E-05 | 5.94229707 |
| Arpc3 | 1448279_at -1.145075 | | 11.1559432 | -10.737168 | 4.30E-06 | 0.00011776 | 4.46567758 |
| Pip4k2a | 1419279_at -1.1445924 | | 9.01313911 | -7.5346601 | 6.06E-05 | 0.00073501 | 1.5696799 |
| Nt5c | 1417252_at -1.1435723 | | 8.92855715 | -9.9606033 | 7.62E-06 | 0.00017629 | 3.83946333 |
| H2-T24 | 1422160_at -1.1428656 | | 7.4944286 | -12.128727 | 1.68E-06 | 6.12E-05 | 5.49111307 |
| Actg1 | 1415779_s_a-1.1428331 | | 12.7466826 | -12.822663 | 1.09E-06 | 4.50E-05 | 5.9618826 |
| Tubb2a | 1427347_s_a-1.1425872 | | 10.5906643 | -17.691764 | 8.56E-08 | 7.71E-06 | 8.69314329 |
| NA | 1421550_a_ -1.1420462 | | 9.02462135 | -15.36047 | 2.63E-07 | 1.63E-05 | 7.49494217 |
| Ptbp3 | 1455819_at -1.1417351 | | 9.94209305 | -12.114038 | 1.69E-06 | 6.17E-05 | 5.48087406 |
| Ncapg2 | 1417926_at -1.1400581 | | 7.79876404 | -6.3316062 | 0.00020742 | 0.00182421 | 0.22323296 |
| Sfrp2 | 1448201_at -1.1393207 | | 8.33025745 | -3.4210907 | 0.00881537 | 0.03108809 | -3.8177604 |
| Vasp | 1451097_at -1.139185 | | 9.91278705 | -5.9861496 | 0.0003046 | 0.00243276 | -0.1961313 |
| Lrmp | 1448409_at -1.1388054 | | 7.59511377 | -11.603067 | 2.36E-06 | 7.70E-05 | 5.11723864 |
| Zeb2 | 1422748_at -1.1377555 | | 8.6959409 | -7.9002488 | 4.29E-05 | 0.0005812 | 1.94679798 |
| Cklf | 1425860_x_a-1.1372535 | | 8.07519332 | -13.147551 | 8.94E-07 | 3.84E-05 | 6.17396274 |
| Arpc4 | 1423589_at -1.1372219 | | 10.5221848 | -7.1348083 | 8.96E-05 | 0.00098431 | 1.14079632 |
| Rnf114 | 1455975_x_a-1.1355583 | | 10.0973954 | -16.514159 | 1.48E-07 | 1.12E-05 | 8.10970126 |
| Trpv2 | 1416935_at -1.1338968 | | 8.38374245 | -9.2974718 | 1.28E-05 | 0.0002538 | 3.2696458 |
| Ncf2 | 1448561_at -1.1331798 | | 8.32459324 | -5.5268944 | 0.00051979 | 0.00354816 | -0.7782085 |
| Cd247 | 1426396_at -1.131833 | | 8.15327932 | -7.179115 | 8.57E-05 | 0.00094796 | 1.18919634 |
| NA | 1456032_x_a-1.1307401 | | 8.84379296 | -13.661362 | 6.62E-07 | 3.12E-05 | 6.49922237 |
| Stat3 | 1426587_a_ -1.1293653 | | 11.1553837 | -8.0443753 | 3.76E-05 | 0.00053179 | 2.09170707 |
| Ppt1 | 1422468_at -1.1286294 | | 9.54633094 | -7.5120896 | 6.19E-05 | 0.00074832 | 1.54593653 |
| BC028528 | 1427996_at -1.1281887 | | 8.88344221 | -5.2501758 | 0.00072713 | 0.00454504 | -1.1429446 |
| Tmem56 | 1434553_at -1.1277023 | | 7.29151108 | -15.782734 | 2.12E-07 | 1.42E-05 | 7.72525354 |
| Il4ra | 1421034_a_ -1.1270987 | | 8.63348055 | -5.0570182 | 0.00092494 | 0.00547675 | -1.403919 |
| Cd6 | 1451910_a_ -1.1266463 | | 8.28349508 | -9.3493927 | 1.23E-05 | 0.00024661 | 3.31550939 |
| Entpd1 | 1423326_at -1.1247532 | | 8.35495523 | -6.9547378 | 0.0001075 | 0.00111773 | 0.94177712 |
| Stk26 | 1419033_at -1.1245129 | | 8.48808642 | -6.9554552 | 0.00010742 | 0.00111773 | 0.94257745 |
| Tnfrsf1a | 1417291_at -1.1235326 | | 10.6694641 | -12.739344 | 1.14E-06 | 4.66E-05 | 5.90666074 |
| Rpl13a | 1433928_a_ -1.1213768 | | 9.48175245 | -11.513881 | 2.51E-06 | 8.08E-05 | 5.05223969 |
| Myh9 | 1420170_at -1.1212397 | | 9.66869515 | -6.7172767 | 0.00013738 | 0.00134707 | 0.67352396 |
| Psma7 | 1423567_a_ -1.1211098 | | 11.1172753 | -15.033834 | 3.12E-07 | 1.85E-05 | 7.31235275 |
| Itga6 | 1422444_at -1.1207376 | | 8.91799334 | -15.47027 | 2.49E-07 | 1.56E-05 | 7.55544109 |
| Arpc4 | 1423588_at -1.1202675 | | 9.88841467 | -7.4439176 | 6.61E-05 | 0.00078417 | 1.47388875 |
| Adam10 | 1428103_at -1.119608 | | 9.93027032 | -10.718914 | 4.35E-06 | 0.00011856 | 4.45143139 |
| Fndc1 | 1453321_at -1.1195646 | | 9.03506259 | -7.1788336 | 8.58E-05 | 0.00094796 | 1.18888968 |
| Atf3 | 1449363_at -1.119545 | | 9.49037439 | -9.7929787 | 8.67E-06 | 0.00019199 | 3.69862248 |
| Tgm2 | 1433428_x_a-1.1191242 | | 12.5756508 | -9.3446515 | 1.24E-05 | 0.00024712 | 3.31133038 |
| Laptm5 | 1417721_s_a-1.1176569 | | 8.74604622 | -5.1334348 | 0.00084042 | 0.00507023 | -1.3000422 |
| Egr2 | 1427683_at -1.1173123 | | 7.31405806 | -8.8698569 | 1.83E-05 | 0.00032237 | 2.88338967 |
| Cklf | 1424495_a_ -1.1167736 | | 7.74918786 | -10.71755 | 4.36E-06 | 0.00011856 | 4.45036612 |
| NA | 1434881_s_a-1.1165929 | | 9.38578758 | -5.7581186 | 0.0003958 | 0.00292055 | -0.4815706 |
| Ikbke | 1417813_at -1.1157486 | | 8.17695828 | -10.25229 | 6.12E-06 | 0.0001496 | 4.07962941 |
| NA | 1448205_at -1.1148025 | | 7.99192626 | -13.078821 | 9.32E-07 | 3.94E-05 | 6.12952214 |
| Asah1 | 1416735_at -1.1137362 | | 10.6322343 | -9.5287489 | 1.07E-05 | 0.00022317 | 3.472273 |
| Ptpre | 1418539_a_ -1.1125842 | | 8.31655125 | -9.4859004 | 1.10E-05 | 0.00022887 | 3.43505488 |
| Pml | 1448757_at -1.1119536 | | 9.06405168 | -10.578357 | 4.82E-06 | 0.00012753 | 4.3409951 |
| Ankfy1 | 1417685_at -1.1110008 | | 9.1630548 | -15.829649 | 2.07E-07 | 1.41E-05 | 7.75045509 |
| Cp | 1417495_x_a-1.1088049 | | 10.400805 | -12.192284 | 1.61E-06 | 5.98E-05 | 5.53528119 |
| Marcksl1 | 1415922_s_a-1.1075637 | | 9.69457283 | -8.2953282 | 3.00E-05 | 0.00045831 | 2.33913992 |
| Snx5 | 1448791_at -1.1075078 | | 9.84975604 | -5.7382541 | 0.00040506 | 0.00296164 | -0.5067678 |
| Gnb4 | 1419470_at -1.1069302 | | 9.06022817 | -15.874524 | 2.03E-07 | 1.40E-05 | 7.77448889 |
| Parp3 | 1426210_x_a-1.1058394 | | 9.44324468 | -9.8344415 | 8.40E-06 | 0.00018825 | 3.73365607 |
| Cnn2 | 1450981_at -1.1047661 | | 11.0979139 | -8.1138821 | 3.53E-05 | 0.00050772 | 2.16085315 |
| Elf1 | 1417540_at -1.103334 | | 8.5316307 | -11.883386 | 1.97E-06 | 6.82E-05 | 5.31854601 |
| Cd47 | 1428187_at -1.1027682 | | 10.3137613 | -7.9662891 | 4.04E-05 | 0.00055787 | 2.01345517 |
| Cotl1 | 1425801_x_a-1.1026514 | | 9.78154679 | -8.5577809 | 2.38E-05 | 0.00039044 | 2.59149696 |
| Tmem176a | 1425603_at -1.1020771 | | 8.99435787 | -10.348646 | 5.70E-06 | 0.0001419 | 4.15763175 |
| S100a8 | 1419394_s_a-1.1002454 | | 8.27016532 | -3.2136336 | 0.01204748 | 0.03976684 | -4.1454835 |
| Tmem229b | 1454632_at -1.0990836 | | 8.47046158 | -7.2937873 | 7.65E-05 | 0.00087105 | 1.31343637 |
| Cp | 1417494_a_ -1.0984045 | | 11.2551353 | -9.3357745 | 1.24E-05 | 0.00024806 | 3.30350109 |
| Asns | 1451095_at -1.0983628 | | 8.82479849 | -6.788874 | 0.0001275 | 0.00127222 | 0.75511216 |
| Gas5 | 1419291_x_a-1.097696 | | 10.1358068 | -8.9365666 | 1.73E-05 | 0.0003094 | 2.94467001 |
| Klrk1 | 1450495_a_ -1.0969771 | | 8.24922109 | -11.518278 | 2.50E-06 | 8.08E-05 | 5.05545521 |
| Bgn | 1448323_a_ -1.0968538 | | 12.0473914 | -8.8752279 | 1.82E-05 | 0.00032167 | 2.88833788 |
| Fignl1 | 1422430_at -1.0963112 | | 8.00786258 | -13.297621 | 8.18E-07 | 3.63E-05 | 6.27022215 |
| Elovl1 | 1456530_x_a-1.095301 | | 9.16542984 | -9.5828353 | 1.02E-05 | 0.00021643 | 3.51904602 |
| Psma7 | 1423568_at -1.0949995 | | 10.3976848 | -15.065241 | 3.07E-07 | 1.83E-05 | 7.33008202 |
| M6pr | 1416386_a_ -1.0947029 | | 9.88990474 | -8.2168733 | 3.22E-05 | 0.00048105 | 2.26244112 |
| Slc20a1 | 1448568_a_ -1.094453 | | 9.39773592 | -14.731864 | 3.66E-07 | 2.05E-05 | 7.13996966 |
| H2afy | 1424572_a_ -1.0942142 | | 10.2666781 | -8.551306 | 2.40E-05 | 0.00039235 | 2.58534798 |
| Marcks | 1415972_at -1.0940979 | | 9.78947067 | -5.9373863 | 0.00032197 | 0.00253048 | -0.2565863 |
| Ptprcap | 1448511_at -1.0939963 | | 9.52485666 | -9.2480351 | 1.34E-05 | 0.00026032 | 3.22577225 |
| Ypel5 | 1433593_at -1.0930822 | | 8.07551966 | -6.6467583 | 0.00014794 | 0.00142596 | 0.59256006 |

| Tcirg1 | 1420635_a_ | -1.0916774 | 8.65805924 | -8.5127747 | 2.48E-05 | 0.0004015 | 2.54867666 |
| --- | --- | --- | --- | --- | --- | --- | --- |
| Il2rb | 1448759_at | -1.0913929 | 8.35876794 | -5.7774203 | 0.00038702 | 0.00287818 | -0.4571386 |
| Ctla2b | 1452352_at | -1.0913659 | 8.26915423 | -11.904139 | 1.94E-06 | 6.76E-05 | 5.33327229 |
| Cyfip1 | 1416329_at | -1.0911941 | 10.9428375 | -10.244507 | 6.15E-06 | 0.00015031 | 4.07329972 |
| NA | 1455869_at | -1.090244 | 8.85017478 | -4.0885283 | 0.00335341 | 0.01477739 | -2.7914849 |
| Evl | 1450106_a_ | -1.0900363 | 8.37201333 | -5.5599856 | 0.00049969 | 0.00344515 | -0.7353053 |
| NA | 1425470_at | -1.0899847 | 7.1147937 | -5.8125661 | 0.00037158 | 0.00279825 | -0.4127805 |
| NA | 1436971_x_a-1.0898382 | | 11.0473996 | -13.372843 | 7.83E-07 | 3.51E-05 | 6.31807544 |
| Bub1 | 1424046_at -1.0893029 | | 7.56005551 | -9.0261843 | 1.60E-05 | 0.00029436 | 3.02639038 |
| H2afx | 1416746_at -1.0892029 | | 9.4117777 | -7.329832 | 7.39E-05 | 0.00084713 | 1.35218557 |
| Stt3a | 1455824_x_a-1.0889517 | | 10.2350755 | -16.867296 | 1.25E-07 | 9.83E-06 | 8.28908887 |
| Ahsp | 1449077_at -1.0887165 | | 8.02391254 | -3.3246818 | 0.01018601 | 0.03485982 | -3.9696694 |
| NA | 1417714_x_a-1.0883887 | | 12.8942464 | -3.2973187 | 0.01061469 | 0.03595808 | -4.0129134 |
| Kars | 1416068_at -1.0867397 | | 10.3802896 | -11.783721 | 2.10E-06 | 7.09E-05 | 5.24748627 |
| Angptl4 | 1417130_s_a-1.0859355 | | 9.49572837 | -5.5045748 | 0.00053385 | 0.00362884 | -0.8072318 |
| Icam2 | 1448862_at -1.0839173 | | 9.89652695 | -9.5807978 | 1.02E-05 | 0.00021657 | 3.51728817 |
| Tmem176b | 1418004_a_ -1.0835346 | | 10.9165959 | -10.221189 | 6.26E-06 | 0.00015229 | 4.0543121 |
| Fyn | 1448765_at -1.0827506 | | 8.81318818 | -12.58334 | 1.26E-06 | 4.96E-05 | 5.80232863 |
| Emb | 1415857_at -1.0818505 | | 8.9698691 | -14.745203 | 3.63E-07 | 2.04E-05 | 7.14765884 |
| Ckap4 | 1455019_x_a-1.0816032 | | 10.6363843 | -12.032769 | 1.78E-06 | 6.36E-05 | 5.42401304 |
| Ccndbp1 | 1420745_a_ -1.0807105 | | 9.94424993 | -4.480529 | 0.0019583 | 0.00975281 | -2.2142038 |
| Snx5 | 1417646_a_ -1.0797756 | | 9.63198181 | -8.0178591 | 3.85E-05 | 0.00054035 | 2.06520263 |
| Phgdh | 1454714_x_a-1.077556 | | 8.72580778 | -7.9034744 | 4.28E-05 | 0.0005812 | 1.95006386 |
| Eif4e3 | 1417978_at -1.077337 | | 10.4351491 | -13.953537 | 5.61E-07 | 2.78E-05 | 6.67889117 |
| Gltp | 1419027_s_a-1.0770542 | | 10.1423836 | -6.4224388 | 0.00018795 | 0.0017002 | 0.33095257 |
| Col1a2 | 1423110_at -1.0769049 | | 10.2852132 | -7.0156457 | 0.00010105 | 0.00107189 | 1.00951314 |
| Slc16a3 | 1449005_at -1.0768095 | | 8.03574141 | -5.2409401 | 0.00073545 | 0.00458319 | -1.155303 |
| Tubb2b | 1452679_at -1.0760574 | | 7.59024483 | -11.273199 | 2.95E-06 | 9.11E-05 | 4.87447141 |
| Anxa5 | 1425567_a_ -1.0753122 | | 11.1504091 | -9.5066375 | 1.09E-05 | 0.00022649 | 3.45308524 |
| Gm4354 | 1424607_a_ -1.0746529 | | 11.2439349 | -11.414689 | 2.68E-06 | 8.52E-05 | 4.9793964 |
| Spata13 | 1454656_at -1.0746367 | | 8.66460041 | -11.91143 | 1.93E-06 | 6.74E-05 | 5.33843977 |
| Sec61b | 1417083_at -1.0735112 | | 10.5630532 | -14.770521 | 3.58E-07 | 2.03E-05 | 7.16223351 |
| Dnmt1 | 1435122_x_a-1.072573 | | 8.57287936 | -13.6631 | 6.62E-07 | 3.12E-05 | 6.50030264 |
| Cp | 1448734_at -1.0706364 | | 9.72030122 | -13.281288 | 8.26E-07 | 3.66E-05 | 6.2597969 |
| Anpep | 1421424_a_ -1.070608 | | 8.84246464 | -4.3660617 | 0.00228601 | 0.01100332 | -2.3806313 |
| Hexb | 1460180_at -1.0705182 | | 9.22318862 | -5.8273611 | 0.00036528 | 0.00276615 | -0.3941575 |
| Ostf1 | 1452246_at -1.0701643 | | 8.80036567 | -6.6235927 | 0.0001516 | 0.00145568 | 0.56583104 |
| Gna13 | 1460317_s_a-1.0698777 | | 8.75152317 | -8.2140515 | 3.23E-05 | 0.00048106 | 2.25967148 |
| Elovl1 | 1455994_x_a-1.0675865 | | 9.30404739 | -11.399909 | 2.71E-06 | 8.58E-05 | 4.96849223 |
| NA | 1450672_a_ -1.0668896 | | 9.73317512 | -9.8629825 | 8.21E-06 | 0.00018581 | 3.75769628 |
| Gem | 1426063_a_ -1.066799 | | 7.88005913 | -12.052927 | 1.76E-06 | 6.31E-05 | 5.43815041 |
| Csrp1 | 1425811_a_ -1.0654354 | | 9.66102228 | -7.0501712 | 9.76E-05 | 0.00104354 | 1.04771829 |
| Adam19 | 1418402_at -1.0625903 | | 9.20215756 | -7.4678606 | 6.46E-05 | 0.00077318 | 1.49925028 |
| NA | 1416076_at -1.0625403 | | 7.44205289 | -8.9011922 | 1.78E-05 | 0.00031646 | 2.9122227 |
| Fam129a | 1422567_at -1.0625026 | | 7.97708713 | -8.3806627 | 2.78E-05 | 0.00043716 | 2.42189899 |
| Gsap | 1427515_at -1.0621333 | | 7.50740094 | -8.138434 | 3.45E-05 | 0.00050103 | 2.18516429 |
| Gng2 | 1418451_at -1.0619653 | | 8.80410792 | -12.015665 | 1.80E-06 | 6.40E-05 | 5.41200013 |
| Mmp3 | 1418945_at -1.0607015 | | 8.316902 | -11.260299 | 2.98E-06 | 9.18E-05 | 4.86484448 |
| Fam107b | 1448509_at -1.0605388 | | 8.28229991 | -8.5972746 | 2.30E-05 | 0.0003809 | 2.6289199 |
| Nfkbie | 1431843_a_ -1.0601929 | | 7.80895815 | -7.2451371 | 8.03E-05 | 0.0009026 | 1.26090688 |
| Tbk1 | 1460315_s_a-1.0596241 | | 9.21150838 | -16.810936 | 1.29E-07 | 9.99E-06 | 8.26072112 |
| Gm4354 | 1424609_a_ -1.0588444 | | 11.2793444 | -10.729405 | 4.32E-06 | 0.00011799 | 4.45962181 |
| Gimap1 | 1449988_at -1.0575283 | | 8.26114128 | -8.0412696 | 3.77E-05 | 0.00053281 | 2.0886064 |
| Cdc42 | 1460708_s_a-1.0574284 | | 11.6604345 | -11.312856 | 2.88E-06 | 8.94E-05 | 4.90400227 |
| Ptpro | 1417676_a_ -1.0571329 | | 8.53011879 | -4.8435534 | 0.00121421 | 0.00674758 | -1.6984784 |
| Adar | 1425405_a_ -1.0570332 | | 8.618225 | -9.6957484 | 9.35E-06 | 0.00020327 | 3.61595691 |
| Prkcd | 1422847_a_ -1.0559256 | | 9.11564068 | -5.0321148 | 0.00095445 | 0.0056105 | -1.4379501 |
| AI413582 | 1452599_s_a-1.0559032 | | 8.18208787 | -11.376622 | 2.75E-06 | 8.68E-05 | 4.95128611 |
| NA | 1416811_s_a-1.0555478 | | 9.78206448 | -9.5038071 | 1.09E-05 | 0.00022658 | 3.45062623 |
| NA | 1451184_at -1.0541322 | | 10.5005845 | -10.187863 | 6.42E-06 | 0.00015464 | 4.02710818 |
| Havcr2 | 1451584_at -1.052696 | | 7.5299357 | -8.9152312 | 1.76E-05 | 0.00031332 | 2.92511299 |
| Rnf115 | 1437009_a_ -1.0526678 | | 9.67506903 | -14.334548 | 4.54E-07 | 2.41E-05 | 6.90770202 |
| Sh2d1a | 1449393_at -1.0519325 | | 7.01393238 | -9.2204006 | 1.37E-05 | 0.00026488 | 3.20115994 |
| Csf1r | 1423593_a_ -1.0512174 | | 9.8590389 | -5.2473122 | 0.0007297 | 0.00455483 | -1.1467751 |
| Bst1 | 1449454_at -1.0494891 | | 7.2663777 | -10.812714 | 4.07E-06 | 0.00011351 | 4.52440369 |
| Fen1 | 1421731_a_ -1.0493045 | | 8.32708022 | -11.365189 | 2.77E-06 | 8.72E-05 | 4.94282596 |
| Clic4 | 1423393_at -1.0492841 | | 12.704454 | -11.155628 | 3.20E-06 | 9.66E-05 | 4.78635561 |
| Atp6ap2 | 1423662_at -1.0488539 | | 10.4765984 | -6.4285456 | 0.00018671 | 0.00169188 | 0.3381573 |
| Nuf2 | 1430811_a_ -1.0488036 | | 7.14458602 | -9.1086854 | 1.50E-05 | 0.00028158 | 3.10101715 |
| Col1a2 | 1450857_a_ -1.0478238 | | 12.1781775 | -8.114469 | 3.53E-05 | 0.00050772 | 2.16143505 |
| Tlr4 | 1418162_at -1.0476043 | | 8.27400159 | -10.940776 | 3.72E-06 | 0.00010725 | 4.62310628 |
| Cadm1 | 1417378_at -1.0463295 | | 8.74657499 | -4.3569551 | 0.00231452 | 0.01110519 | -2.3939487 |
| Abracl | 1426964_at -1.0451535 | | 10.7778747 | -11.514722 | 2.51E-06 | 8.08E-05 | 5.05285476 |
| Sdcbp | 1423332_at -1.0450707 | | 11.9805662 | -13.310871 | 8.12E-07 | 3.63E-05 | 6.2786704 |
| Bak1 | 1418991_at -1.0448009 | | 9.20805428 | -4.463136 | 0.00200464 | 0.00993778 | -2.2393758 |
| Caprin1 | 1448347_a_ -1.043085 | | 10.8895548 | -13.864667 | 5.90E-07 | 2.86E-05 | 6.62463556 |
| Gnai3 | 1428645_at -1.0429259 | | 9.66179116 | -13.940562 | 5.65E-07 | 2.80E-05 | 6.67099122 |
| Rab31 | 1416165_at -1.0418201 | | 9.3507778 | -7.956677 | 4.08E-05 | 0.00056179 | 2.0037805 |
| Diaph1 | 1454979_at -1.0412706 | | 10.0678182 | -10.268326 | 6.05E-06 | 0.00014863 | 4.09265554 |
| Psma5 | 1424681_a_ -1.0409645 | | 11.1818018 | -12.464207 | 1.36E-06 | 5.24E-05 | 5.72181941 |
| Samsn1 | 1421457_a_ -1.0404841 | | 7.62472903 | -9.6694009 | 9.54E-06 | 0.00020703 | 3.59343167 |
| Sh3bgrl | 1428107_at -1.0402738 | | 9.83975547 | -6.4791741 | 0.00017681 | 0.00163146 | 0.39770888 |
| Tgfb1 | 1420653_at -1.0391048 | | 9.80909825 | -5.1055262 | 0.00087027 | 0.00520814 | -1.3378839 |
| Vav1 | 1422932_a_ -1.0377722 | | 7.35744761 | -8.3242594 | 2.92E-05 | 0.00044959 | 2.36727527 |
| Dcaf12 | 1438669_at -1.0375308 | | 10.0197699 | -4.1084745 | 0.00326109 | 0.01446631 | -2.7616191 |

| Anxa1 | 1448213_at | -1.0374313 | 10.6128882 | -10.636531 | 4.62E-06 | 0.00012403 | 4.38686256 |
| --- | --- | --- | --- | --- | --- | --- | --- |
| Cd86 | 1420404_at | -1.0373575 | 8.14396902 | -5.9289883 | 0.00032507 | 0.00255106 | -0.2670297 |
| Mtpn | 1420474_at | -1.0367288 | 10.8777892 | -11.008449 | 3.55E-06 | 0.00010401 | 4.67484006 |
| Tnfsf13b | 1460255_at | -1.0365847 | 8.06870746 | -5.9527988 | 0.00031637 | 0.00249769 | -0.2374443 |
| Mcm4 | 1416214_at | -1.0365162 | 8.83189633 | -7.3319818 | 7.37E-05 | 0.00084578 | 1.35449214 |
| Rassf2 | 1428392_at | -1.0364417 | 7.86655755 | -6.4339412 | 0.00018563 | 0.00168542 | 0.34451919 |
| Smpdl3b | 1417300_at | -1.0353206 | 8.31584836 | -10.007626 | 7.35E-06 | 0.00017238 | 3.87859776 |
| Lig1 | 1416641_at | -1.0341278 | 8.43023231 | -9.9841942 | 7.49E-06 | 0.00017457 | 3.85911712 |
| Smarcd2 | 1448400_a_ | -1.0313121 | 10.0677715 | -8.4064261 | 2.72E-05 | 0.00043007 | 2.44674981 |
| Kcnab2 | 1416956_at | -1.0295118 | 8.37843226 | -9.9513665 | 7.68E-06 | 0.00017705 | 3.83175688 |
| Nupr1 | 1419665_a_ | -1.0289779 | 8.40914724 | -7.8652438 | 4.43E-05 | 0.00059432 | 1.91128716 |
| Fstl1 | 1416221_at | -1.0280585 | 10.7275864 | -9.9775047 | 7.52E-06 | 0.00017492 | 3.85354813 |
| Soat1 | 1417696_at | -1.0276192 | 8.67250723 | -9.8421203 | 8.35E-06 | 0.00018774 | 3.74012998 |
| Nfkbia | 1448306_at | -1.0275441 | 10.2573831 | -8.7577321 | 2.01E-05 | 0.00034557 | 2.77951492 |
| Cdc42 | 1415724_a_ | -1.0271346 | 11.116045 | -8.3274578 | 2.92E-05 | 0.0004489 | 2.3703808 |
| Stat4 | 1448713_at | -1.0261735 | 7.80306428 | -9.0935665 | 1.52E-05 | 0.00028346 | 3.08738436 |
| Serp1 | 1415828_a_ | -1.0261723 | 9.97412751 | -11.142203 | 3.23E-06 | 9.73E-05 | 4.77624029 |
| Lfng | 1420643_at | -1.0256408 | 8.55286665 | -4.914454 | 0.00110847 | 0.00627839 | -1.5999261 |
| Kif22 | 1437716_x_a-1.0246103 | | 8.35468967 | -14.394771 | 4.39E-07 | 2.36E-05 | 6.94331699 |
| Nras | 1454060_a_ -1.0238965 | | 8.96063641 | -10.511994 | 5.05E-06 | 0.00013167 | 4.28839237 |
| Cib1 | 1448564_at -1.0236536 | | 9.54121342 | -8.9244509 | 1.74E-05 | 0.00031182 | 2.93356897 |
| Gdf15 | 1418949_at -1.023388 | | 8.4315869 | -14.069452 | 5.26E-07 | 2.65E-05 | 6.74914844 |
| NA | 1421571_a_ -1.0233163 | | 12.6881675 | -13.026031 | 9.62E-07 | 4.05E-05 | 6.09523474 |
| Map4k1 | 1439323_a_ -1.0221736 | | 7.75273004 | -6.9344471 | 0.00010975 | 0.00113554 | 0.91911581 |
| Cd3e | 1422105_at -1.0220715 | | 9.91636254 | -6.805493 | 0.00012532 | 0.00125655 | 0.77396235 |
| Arpc2 | 1437148_at -1.0215178 | | 12.6708202 | -10.43653 | 5.34E-06 | 0.00013631 | 4.22821123 |
| Hnrnpu | 1423051_at -1.0211797 | | 11.6897678 | -10.915052 | 3.79E-06 | 0.00010852 | 4.60336502 |
| Il12rb1 | 1418166_at -1.0202004 | | 8.46490374 | -8.3722352 | 2.80E-05 | 0.00043886 | 2.4137564 |
| Csk | 1423518_at -1.0200389 | | 9.04040398 | -5.3259387 | 0.00066258 | 0.00426618 | -1.0420183 |
| Gba | 1437044_a_ -1.019719 | | 8.82719433 | -11.334059 | 2.83E-06 | 8.86E-05 | 4.9197515 |
| Anxa7 | 1416138_at -1.0185909 | | 10.7354879 | -10.984792 | 3.61E-06 | 0.0001052 | 4.65678813 |
| Relb | 1417856_at -1.0181549 | | 8.19690494 | -8.2734437 | 3.06E-05 | 0.00046376 | 2.31780456 |
| Sh3bgrl | 1436997_x_a-1.0180405 | | 10.9160216 | -9.4031862 | 1.18E-05 | 0.00023927 | 3.36279694 |
| Arrdc4 | 1426818_at -1.0175862 | | 8.74097303 | -9.1511814 | 1.45E-05 | 0.00027496 | 3.13923363 |
| Noc4l | 1438095_x_a-1.017528 | | 7.96152706 | -13.186355 | 8.74E-07 | 3.78E-05 | 6.19895474 |
| Rtn4 | 1452649_at -1.0172456 | | 11.4849884 | -10.55444 | 4.90E-06 | 0.00012878 | 4.32207135 |
| NA | 1416415_a_ -1.0168714 | | 11.4070045 | -11.750937 | 2.14E-06 | 7.20E-05 | 5.22398856 |
| Car2 | 1448752_at -1.0158792 | | 9.16941544 | -4.2661296 | 0.00262078 | 0.01221838 | -2.5273868 |
| Ywhaq | 1460590_s_a-1.0157546 | | 11.0937793 | -10.841859 | 3.99E-06 | 0.00011197 | 4.54696049 |
| Mis18a | 1453314_x_a-1.0144208 | | 8.44644151 | -10.425779 | 5.38E-06 | 0.00013693 | 4.21960582 |
| Gba | 1450099_a_ -1.014051 | | 9.37798719 | -10.039316 | 7.18E-06 | 0.0001695 | 3.90488011 |
| Kif11 | 1435306_a_ -1.0140282 | | 7.40239376 | -7.3159022 | 7.49E-05 | 0.00085706 | 1.33722764 |
| Gas5 | 1449410_a_ -1.013884 | | 10.3332201 | -7.8828294 | 4.36E-05 | 0.00058784 | 1.92914244 |
| Anln | 1433543_at -1.0134507 | | 7.3886803 | -7.3002053 | 7.61E-05 | 0.00086688 | 1.32034646 |
| Crem | 1449037_at -1.0129963 | | 8.12908442 | -11.996534 | 1.83E-06 | 6.46E-05 | 5.39854432 |
| Oas3 | 1425374_at -1.0126975 | | 8.2112212 | -6.0303738 | 0.00028974 | 0.00234963 | -0.1415763 |
| Csf2rb | 1421326_at -1.0117941 | | 7.85872556 | -5.838035 | 0.00036081 | 0.00273898 | -0.3807401 |
| Ptpn12 | 1422045_a_ -1.0114152 | | 9.85708259 | -16.621479 | 1.41E-07 | 1.08E-05 | 8.16463424 |
| Clec4e | 1420330_at -1.0108472 | | 7.62948183 | -10.468776 | 5.22E-06 | 0.00013344 | 4.25397426 |
| Acsl4 | 1433531_at -1.0108263 | | 8.55579749 | -11.414598 | 2.68E-06 | 8.52E-05 | 4.97932947 |
| Flna | 1426677_at -1.0098749 | | 9.80563983 | -7.2552625 | 7.95E-05 | 0.00089667 | 1.27186141 |
| Il17ra | 1420905_at -1.0098363 | | 8.06615262 | -6.675765 | 0.00014349 | 0.00139613 | 0.62593643 |
| Plaur | 1452521_a_ -1.0093117 | | 9.14096666 | -11.275246 | 2.95E-06 | 9.11E-05 | 4.875998 |
| Axl | 1423586_at -1.0087508 | | 10.520169 | -6.3198776 | 0.0002101 | 0.00184059 | 0.20924763 |
| sep-06 | 1424181_at -1.0082662 | | 8.15853692 | -8.0673319 | 3.68E-05 | 0.00052271 | 2.11459726 |
| Id2 | 1453596_at -1.0079748 | | 7.47767101 | -13.386831 | 7.76E-07 | 3.50E-05 | 6.3269456 |
| Mfsd1 | 1451247_at -1.0077443 | | 9.05860261 | -8.4252709 | 2.68E-05 | 0.00042524 | 2.46488783 |
| Sptlc2 | 1460243_at -1.0058239 | | 8.78855924 | -11.77353 | 2.11E-06 | 7.13E-05 | 5.24018872 |
| Mdfic | 1427040_at -1.0052677 | | 9.69164838 | -6.5707521 | 0.00016033 | 0.00151579 | 0.50461626 |
| Rrbp1 | 1426123_a_ -1.0051345 | | 8.07411404 | -13.30379 | 8.15E-07 | 3.63E-05 | 6.27415626 |
| Mkrn1 | 1418434_at -1.0044062 | | 9.58554584 | -6.6134157 | 0.00015324 | 0.00146832 | 0.55406781 |
| Slc13a3 | 1438377_x_a-1.0032674 | | 7.03693566 | -11.519028 | 2.50E-06 | 8.08E-05 | 5.05600371 |
| Snx20 | 1426299_at -1.0013917 | | 8.84613291 | -8.3545993 | 2.85E-05 | 0.00044186 | 2.39669521 |
| Dctn6 | 1416499_a_ -1.0006669 | | 10.4530685 | -8.417034 | 2.69E-05 | 0.00042697 | 2.45696395 |
| Sirt7 | 1424238_at -1.0000256 | | 9.02042294 | -7.006134 | 0.00010203 | 0.00107882 | 0.99896352 |
| Smad5 | 1448525_a_ -0.9998177 | | 9.42871025 | -3.4969648 | 0.0078741 | 0.02849039 | -3.6987509 |
| Tspan32 | 1418398_a_ -0.9997747 | | 6.98992356 | -6.2793951 | 0.00021962 | 0.00190707 | 0.16084105 |
| Aldh18a1 | 1437325_x_a-0.9992739 | | 8.83449467 | -9.5351098 | 1.06E-05 | 0.00022237 | 3.47778573 |
| Ets1 | 1426725_s_a-0.9988737 | | 10.2129802 | -9.2180167 | 1.37E-05 | 0.00026494 | 3.19903389 |
| Ppp4c | 1460288_a_ -0.9984903 | | 9.88281594 | -6.9080951 | 0.00011275 | 0.00116187 | 0.88961287 |
| Ly9 | 1449156_at -0.9980086 | | 7.60912591 | -6.9420474 | 0.0001089 | 0.00112882 | 0.92760972 |
| Stt3a | 1450841_at -0.9973708 | | 9.00002166 | -7.957236 | 4.07E-05 | 0.00056179 | 2.00434348 |
| Adam17 | 1421858_at -0.9972036 | | 9.66451658 | -7.5711383 | 5.85E-05 | 0.00071784 | 1.60793847 |
| Mical1 | 1456439_x_a-0.9968696 | | 7.62795565 | -7.6522659 | 5.41E-05 | 0.00067993 | 1.69251853 |
| Cbfb | 1460716_a_ -0.9962361 | | 10.6187287 | -12.336688 | 1.47E-06 | 5.53E-05 | 5.63482753 |
| Abi1 | 1450890_a_ -0.9954396 | | 9.35686541 | -8.6250764 | 2.25E-05 | 0.00037488 | 2.65517882 |
| Plekho2 | 1427103_at -0.9948372 | | 9.10508888 | -5.5836941 | 0.00048581 | 0.00337149 | -0.7046599 |
| Txnrd1 | 1421529_a_ -0.9947973 | | 10.0538573 | -13.236001 | 8.49E-07 | 3.70E-05 | 6.23082506 |
| Ppbp | 1418480_at -0.9945284 | | 7.93342207 | -6.1692233 | 0.00024803 | 0.00208205 | 0.02803919 |
| Myo1f | 1421618_at -0.9941585 | | 8.62038745 | -10.909557 | 3.80E-06 | 0.00010867 | 4.59914212 |
| Gm20559 | 1436172_at -0.9939146 | | 8.28788629 | -10.508466 | 5.07E-06 | 0.00013186 | 4.28558754 |
| Adgre5 | 1418394_a_ -0.9938432 | | 9.87176292 | -7.4083832 | 6.84E-05 | 0.0008037 | 1.43613443 |
| Gja4 | 1416255_at -0.9937332 | | 8.84043523 | -12.170105 | 1.63E-06 | 6.02E-05 | 5.51989245 |
| Rcan1 | 1416601_a_ -0.9937258 | | 9.22309693 | -8.3638499 | 2.82E-05 | 0.00044026 | 2.40564802 |
| Crip1 | 1416326_at -0.9934365 | | 12.4129758 | -7.3908582 | 6.96E-05 | 0.00081247 | 1.41746384 |

| Cybc1 | 1423678_at | -0.9930033 | 9.11401298 | -9.7403401 | 9.03E-06 | 0.00019797 | 3.65395847 |
| --- | --- | --- | --- | --- | --- | --- | --- |
| Bgn | 1416405_at | -0.99281 | 12.4640355 | -6.5609716 | 0.00016201 | 0.00152429 | 0.49324816 |
| Ctla2a | 1448471_a_ | -0.9918937 | 9.43294519 | -7.7324971 | 5.02E-05 | 0.00064618 | 1.77548303 |
| Tbk1 | 1422469_at | -0.9904013 | 8.22214732 | -8.9149638 | 1.76E-05 | 0.00031332 | 2.92486765 |
| Ctps | 1416563_at | -0.9902841 | 10.7167419 | -11.770837 | 2.12E-06 | 7.13E-05 | 5.23825896 |
| Slc41a3 | 1451707_s_a-0.990244 | | 9.48616155 | -7.6866952 | 5.24E-05 | 0.0006655 | 1.72820324 |
| Mafb | 1451716_at -0.9892949 | | 8.65141356 | -5.1161938 | 0.00085873 | 0.00515463 | -1.3234066 |
| Slc3a2 | 1425364_a_ -0.9891618 | | 10.5531594 | -14.354358 | 4.49E-07 | 2.40E-05 | 6.91943397 |
| NA | 1416922_a_ -0.9889143 | | 10.9260998 | -5.3557103 | 0.00063896 | 0.00415533 | -1.0025791 |
| Galnt7 | 1452232_at -0.9886323 | | 8.36065372 | -8.9172125 | 1.75E-05 | 0.00031322 | 2.92693074 |
| Il16 | 1417391_a_ -0.9878322 | | 8.32131958 | -6.5918259 | 0.00015678 | 0.00149346 | 0.52907093 |
| Mtpn | 1420475_at -0.9876109 | | 9.76011289 | -8.799525 | 1.94E-05 | 0.00033659 | 2.81836201 |
| Il21 | 1450334_at -0.9872178 | | 7.50269032 | -11.816626 | 2.05E-06 | 7.01E-05 | 5.27100901 |
| sep-09 | 1417038_at -0.9871797 | | 9.83573128 | -7.8949143 | 4.31E-05 | 0.00058338 | 1.94139441 |
| Rnasel | 1426604_at -0.9867903 | | 7.85264851 | -14.014016 | 5.42E-07 | 2.70E-05 | 6.71561946 |
| Hells | 1417541_at -0.9867443 | | 7.27457643 | -9.6158928 | 9.95E-06 | 0.00021265 | 3.54752092 |
| Lag3 | 1449911_at -0.9865136 | | 8.08426822 | -6.9996826 | 0.0001027 | 0.00108129 | 0.99180216 |
| Lrrk1 | 1451985_at -0.9856716 | | 8.45515701 | -8.1278634 | 3.49E-05 | 0.00050361 | 2.17470463 |
| Cdc42se1 | 1428132_at -0.9851549 | | 8.92460439 | -11.129299 | 3.26E-06 | 9.79E-05 | 4.76650654 |
| Ugdh | 1416308_at -0.9831502 | | 9.50055206 | -9.2302191 | 1.35E-05 | 0.00026343 | 3.20991187 |
| Sdcbp | 1450941_at -0.983144 | | 9.27027364 | -13.483665 | 7.34E-07 | 3.36E-05 | 6.38810187 |
| Adam9 | 1416094_at -0.9823011 | | 10.6193525 | -13.543066 | 7.09E-07 | 3.28E-05 | 6.42540555 |
| Tubb5 | 1416256_a_ -0.98211 | | 11.8979706 | -10.580201 | 4.81E-06 | 0.00012753 | 4.34245269 |
| Rtn4 | 1421116_a_ -0.9814561 | | 10.2695967 | -7.4999771 | 6.26E-05 | 0.00075629 | 1.53317205 |
| Tnfsf10 | 1420412_at -0.9799754 | | 7.90355608 | -11.213992 | 3.08E-06 | 9.42E-05 | 4.830204 |
| Ptbp3 | 1424083_at -0.9786329 | | 7.2628216 | -8.1643402 | 3.37E-05 | 0.00049436 | 2.21075267 |
| Atp1b3 | 1423126_at -0.9765395 | | 10.8746019 | -9.5009302 | 1.09E-05 | 0.00022689 | 3.44812624 |
| Anp32b | 1417082_at -0.9757041 | | 10.371407 | -11.034003 | 3.49E-06 | 0.00010297 | 4.69429986 |
| Ugcg | 1435133_at -0.975093 | | 7.90502105 | -8.6108797 | 2.28E-05 | 0.00037811 | 2.64177873 |
| Uck2 | 1448604_at -0.9748643 | | 9.7493666 | -8.8559841 | 1.85E-05 | 0.0003249 | 2.87059738 |
| Wsb1 | 1425241_a_ -0.9747468 | | 9.08585268 | -11.422713 | 2.67E-06 | 8.49E-05 | 4.98531106 |
| Sirpa | 1448534_at -0.9730916 | | 9.37185824 | -6.035292 | 0.00028814 | 0.00233831 | -0.1355251 |
| Lbr | 1415829_at -0.9729029 | | 10.0139217 | -9.955009 | 7.65E-06 | 0.00017686 | 3.83479665 |
| Abcg1 | 1423570_at -0.9708851 | | 8.95374264 | -6.4236399 | 0.0001877 | 0.00169951 | 0.33237004 |
| Mcm7 | 1439269_x_a-0.969221 | | 7.66023015 | -10.398244 | 5.49E-06 | 0.0001384 | 4.19752912 |
| Zdhhc20 | 1451437_at -0.9686243 | | 7.66257988 | -11.622415 | 2.33E-06 | 7.62E-05 | 5.13127814 |
| Phc2 | 1416048_at -0.968617 | | 9.64303089 | -9.0711627 | 1.54E-05 | 0.0002878 | 3.06714716 |
| Rnf145 | 1452769_at -0.968606 | | 9.08660051 | -5.5320563 | 0.0005166 | 0.00352955 | -0.7715061 |
| Tes | 1424246_a_ -0.9681982 | | 8.30973305 | -9.084139 | 1.53E-05 | 0.00028544 | 3.0788737 |
| Hist1h1c | 1436994_a_ -0.9676465 | | 11.000929 | -8.7431312 | 2.03E-05 | 0.00034803 | 2.76590675 |
| Trim21 | 1418077_at -0.9664585 | | 8.99256549 | -11.144467 | 3.23E-06 | 9.73E-05 | 4.77794647 |
| Stap1 | 1421098_at -0.96599 | | 7.3714297 | -6.262187 | 0.00022381 | 0.00193164 | 0.14020124 |
| Ywhaz | 1436981_a_ -0.965218 | | 8.03356536 | -7.5676036 | 5.87E-05 | 0.00071949 | 1.6042374 |
| Atp6v1a | 1450634_at -0.9651641 | | 9.22511244 | -8.1519063 | 3.41E-05 | 0.0004972 | 2.1984794 |
| Zyx | 1417240_at -0.9650016 | | 10.7136745 | -4.992279 | 0.00100382 | 0.00582075 | -1.4925695 |
| Ifnar2 | 1451462_a_ -0.9645954 | | 10.1011511 | -8.5863426 | 2.33E-05 | 0.00038367 | 2.61857534 |
| Snx2 | 1460224_at -0.9632194 | | 9.8929076 | -8.8631964 | 1.84E-05 | 0.00032368 | 2.87725002 |
| Rgs10 | 1416882_at -0.9631252 | | 8.88648518 | -3.682146 | 0.00599634 | 0.02309967 | -3.4105747 |
| Sema7a | 1459903_at -0.9626141 | | 9.57365949 | -9.0999554 | 1.51E-05 | 0.00028314 | 3.09314756 |
| Casp3 | 1449839_at -0.9619998 | | 8.79370239 | -7.7145299 | 5.11E-05 | 0.00065263 | 1.75696212 |
| Rrbp1 | 1449221_a_ -0.9618132 | | 8.9408889 | -11.749669 | 2.15E-06 | 7.20E-05 | 5.22307846 |
| Cd7 | 1419711_at -0.9613833 | | 8.75997033 | -5.4401888 | 0.00057679 | 0.00384356 | -0.8913433 |
| Pilrb1 | 1422041_at -0.9610511 | | 8.98816036 | -14.846793 | 3.44E-07 | 1.96E-05 | 7.20599174 |
| Cdt1 | 1424144_at -0.9605554 | | 7.92112265 | -7.9019085 | 4.29E-05 | 0.0005812 | 1.94847852 |
| Plgrkt | 1460361_at -0.960465 | | 9.54830881 | -11.425891 | 2.66E-06 | 8.49E-05 | 4.98765224 |
| Hist1h1c | 1416101_a_ -0.9604266 | | 12.3346834 | -10.672911 | 4.50E-06 | 0.0001212 | 4.41543187 |
| NA | 1449968_s_a-0.9602779 | | 9.32453796 | -12.005986 | 1.82E-06 | 6.43E-05 | 5.40519489 |
| Kcnn4 | 1421038_a_ -0.9594724 | | 7.34882418 | -8.3612389 | 2.83E-05 | 0.00044044 | 2.40312188 |
| Glipr2 | 1452803_at -0.9581042 | | 8.35959553 | -11.992961 | 1.83E-06 | 6.46E-05 | 5.39602865 |
| Atf4 | 1438992_x_a-0.9578581 | | 8.71312785 | -8.1338905 | 3.47E-05 | 0.00050209 | 2.18066979 |
| Ogfr | 1422512_a_ -0.9577742 | | 8.82930706 | -6.4769966 | 0.00017722 | 0.00163394 | 0.39515413 |
| Fes | 1452410_a_ -0.956334 | | 8.37550474 | -5.7855651 | 0.00038338 | 0.00286054 | -0.446844 |
| Ppib | 1437649_x_a-0.9559805 | | 10.8235546 | -15.033405 | 3.12E-07 | 1.85E-05 | 7.31211005 |
| Coro1b | 1448351_at -0.9557819 | | 10.0814829 | -6.3108534 | 0.00021218 | 0.0018524 | 0.19847514 |
| Prf1 | 1451862_a_ -0.95467 | | 8.06498455 | -13.442154 | 7.52E-07 | 3.43E-05 | 6.36193743 |
| Cd244a | 1449991_at -0.954203 | | 6.79851705 | -7.683003 | 5.26E-05 | 0.00066745 | 1.72438235 |
| Spred1 | 1423161_s_a-0.9541352 | | 8.63648437 | -13.998048 | 5.47E-07 | 2.72E-05 | 6.70593772 |
| Pag1 | 1436570_at -0.9541064 | | 8.38953309 | -8.5627269 | 2.37E-05 | 0.00038989 | 2.59619146 |
| Cx3cr1 | 1450020_at -0.9538475 | | 8.1224241 | -6.2767175 | 0.00022027 | 0.00191122 | 0.157632 |
| NA | 1426260_a_ -0.9535063 | | 9.35445668 | -3.6978443 | 0.00586069 | 0.02268495 | -3.3863063 |
| Bcl10 | 1418970_a_ -0.9525622 | | 9.94398351 | -9.2354174 | 1.35E-05 | 0.00026256 | 3.21454228 |
| Cxcl1 | 1419209_at -0.9516629 | | 8.41560429 | -6.9129651 | 0.00011219 | 0.00115813 | 0.89507133 |
| Sgpl1 | 1415892_at -0.9515953 | | 8.38446697 | -5.796364 | 0.00037861 | 0.00283334 | -0.4332087 |
| Chfr | 1434529_x_a-0.9514772 | | 8.15401951 | -9.7558044 | 8.92E-06 | 0.00019637 | 3.66710185 |
| Gns | 1433546_at -0.9503109 | | 11.5539737 | -7.2866413 | 7.71E-05 | 0.00087632 | 1.30573706 |
| H13 | 1417287_at -0.9501545 | | 9.53421254 | -7.9674793 | 4.04E-05 | 0.00055787 | 2.01465247 |
| Arhgap17 | 1426623_a_ -0.9482933 | | 9.13893786 | -7.4677637 | 6.46E-05 | 0.00077318 | 1.49914767 |
| Traf1 | 1423602_at -0.9481844 | | 7.46497679 | -7.8513277 | 4.49E-05 | 0.00059957 | 1.8971354 |
| Tinagl1 | 1417109_at -0.9479476 | | 10.6551249 | -8.6639342 | 2.17E-05 | 0.00036525 | 2.69176305 |
| Tdrd7 | 1426716_at -0.9475616 | | 8.78961696 | -15.732474 | 2.18E-07 | 1.44E-05 | 7.69817127 |
| NA | 1424269_a_ -0.9474349 | | 12.0081085 | -8.0377467 | 3.78E-05 | 0.00053375 | 2.08508801 |
| Topbp1 | 1452241_at -0.9472873 | | 8.71132404 | -9.7496868 | 8.97E-06 | 0.00019696 | 3.66190453 |
| Aprt | 1451703_s_a-0.9464608 | | 10.2740845 | -10.522816 | 5.01E-06 | 0.00013079 | 4.29699045 |
| Mcm3 | 1449705_x_a-0.9462094 | | 7.9631273 | -6.2259703 | 0.00023292 | 0.00198656 | 0.09663769 |
| Clic4 | 1438606_a_ -0.9448165 | | 12.4705463 | -9.4690768 | 1.12E-05 | 0.00023108 | 3.42040222 |

| Baiap3 | 1427509_at | -0.9441507 | 8.07550244 | -6.7663085 | 0.00013053 | 0.00129501 | 0.72946421 |
| --- | --- | --- | --- | --- | --- | --- | --- |
| Asf1b | 1423714_at | -0.9433703 | 8.83616057 | -12.590611 | 1.25E-06 | 4.95E-05 | 5.80721831 |
| Col5a2 | 1422437_at | -0.9430243 | 10.182586 | -9.186976 | 1.40E-05 | 0.00026875 | 3.17130661 |
| Gpr35 | 1449976_a_ | -0.9425252 | 7.87638815 | -9.6162264 | 9.95E-06 | 0.00021265 | 3.54780781 |
| Bin3 | 1417691_at | -0.9418467 | 9.50890073 | -9.567121 | 1.03E-05 | 0.00021831 | 3.50548018 |
| Ly6i | 1425890_at | -0.9418175 | 8.84960595 | -9.8481574 | 8.31E-06 | 0.00018719 | 3.74521669 |
| Lipa | 1450872_s_a-0.9411263 | | 9.9114512 | -4.190464 | 0.00290935 | 0.01324234 | -2.6393967 |
| Prkcb | 1423478_at -0.9401943 | | 7.62592521 | -10.50359 | 5.09E-06 | 0.00013218 | 4.2817094 |
| Cdca4 | 1423682_a_ -0.9380332 | | 8.27583034 | -10.76548 | 4.21E-06 | 0.0001161 | 4.48772993 |
| Hbb-b2 | 1427866_x_a-0.9379558 | | 9.67193133 | -2.4169598 | 0.0414899 | 0.10237124 | -5.4145231 |
| Rnf114 | 1427874_at -0.937921 | | 10.3293646 | -11.809465 | 2.06E-06 | 7.01E-05 | 5.2658949 |
| Lap3 | 1450860_at -0.9373896 | | 10.618733 | -9.4744743 | 1.11E-05 | 0.00023044 | 3.42510567 |
| Kif2c | 1437611_x_a-0.9373284 | | 7.12143773 | -9.7057431 | 9.28E-06 | 0.00020221 | 3.62448781 |
| Tmem176a | 1423909_at -0.9365845 | | 9.54489203 | -6.9670933 | 0.00010616 | 0.00110999 | 0.95555269 |
| Arf3 | 1423973_a_ -0.9362033 | | 9.83501336 | -8.242437 | 3.15E-05 | 0.0004736 | 2.28749744 |
| Actb | AFFX-b-Actin -0.935877 | | 13.6308621 | -12.06721 | 1.74E-06 | 6.28E-05 | 5.44815434 |
| S1pr5 | 1449365_at -0.9357461 | | 7.46602107 | -15.846197 | 2.06E-07 | 1.40E-05 | 7.75932592 |
| Plbd1 | 1448786_at -0.9356797 | | 10.7687436 | -6.0556508 | 0.00028161 | 0.00229515 | -0.1105104 |
| Dgkz | 1452169_a_ -0.9352863 | | 10.1267371 | -9.7494406 | 8.97E-06 | 0.00019696 | 3.66169532 |
| Acp5 | 1431609_a_ -0.9344229 | | 8.57276824 | -4.1704372 | 0.00299132 | 0.01354746 | -2.6691697 |
| Nfkbia | 1420088_at -0.9342274 | | 12.3993877 | -14.291215 | 4.65E-07 | 2.42E-05 | 6.88198443 |
| Coro1c | 1449660_s_a-0.9339839 | | 8.35048448 | -5.8462055 | 0.00035743 | 0.00272128 | -0.3704799 |
| Egr2 | 1427682_a_ -0.9337974 | | 7.07377976 | -9.227324 | 1.36E-05 | 0.00026361 | 3.20733213 |
| Ckap4 | 1426755_at -0.9316309 | | 9.60623626 | -12.686306 | 1.18E-06 | 4.76E-05 | 5.87132782 |
| Rhox4b | 1439200_x_a-0.9315098 | | 9.71213361 | -4.5405875 | 0.00180699 | 0.00917007 | -2.1276069 |
| Cadm1 | 1417377_at -0.9312637 | | 8.02009469 | -7.3559218 | 7.20E-05 | 0.00083425 | 1.38014335 |
| Pxn | 1456135_s_a-0.9310678 | | 10.5107176 | -8.8229876 | 1.90E-05 | 0.00033201 | 2.8401033 |
| C5ar1 | 1422190_at -0.9306018 | | 8.62915268 | -5.6731545 | 0.00043712 | 0.00312496 | -0.5897202 |
| Clta | 1421062_s_a-0.9298921 | | 11.7166063 | -10.690012 | 4.44E-06 | 0.00011994 | 4.42883034 |
| Katna1 | 1450949_at -0.9297866 | | 9.21656724 | -11.340743 | 2.82E-06 | 8.83E-05 | 4.92471092 |
| Slfn4 | 1427102_at -0.929638 | | 8.12373636 | -6.7799957 | 0.00012868 | 0.00127905 | 0.74502836 |
| Tgm2 | 1417500_a_ -0.9291224 | | 11.7742528 | -5.7478915 | 0.00040054 | 0.00294303 | -0.4945365 |
| Tpd52 | 1419494_a_ -0.9288475 | | 9.57960156 | -9.9154737 | 7.89E-06 | 0.00018009 | 3.80175088 |
| G3bp2 | 1421323_a_ -0.9285886 | | 10.3544212 | -11.836645 | 2.03E-06 | 6.94E-05 | 5.28528972 |
| Ncapg | 1429171_a_ -0.925959 | | 7.14002778 | -7.9249616 | 4.20E-05 | 0.00057395 | 1.97179311 |
| Mospd2 | 1424124_at -0.9251343 | | 8.59179085 | -8.2146735 | 3.22E-05 | 0.00048106 | 2.26028212 |
| NA | 1417365_a_ -0.9245194 | | 11.4131952 | -6.3082872 | 0.00021278 | 0.00185617 | 0.19540985 |
| Ywhaq | 1460621_x_a-0.9238926 | | 10.9451585 | -9.8206757 | 8.49E-06 | 0.00018913 | 3.72203917 |
| Klf6 | 1418280_at -0.9226954 | | 8.47813495 | -7.1498384 | 8.83E-05 | 0.00097293 | 1.15724003 |
| Stxbp3 | 1416653_at -0.9223494 | | 8.31712486 | -8.4636816 | 2.59E-05 | 0.00041567 | 2.50175524 |
| Brd2 | 1437210_a_ -0.9221095 | | 9.74184305 | -8.9879076 | 1.65E-05 | 0.00030047 | 2.99157086 |
| Nedd9 | 1437132_x_a-0.9211032 | | 8.9074117 | -12.54041 | 1.29E-06 | 5.07E-05 | 5.77340113 |
| Crlf3 | 1460338_a_ -0.9208537 | | 8.41244781 | -5.7385054 | 0.00040494 | 0.00296164 | -0.5064488 |
| Dok2 | 1416333_at -0.9207382 | | 9.01392909 | -5.979225 | 0.00030701 | 0.00244592 | -0.2046969 |
| Marco | 1449498_at -0.9203603 | | 8.11267975 | -7.3351215 | 7.35E-05 | 0.00084532 | 1.35785985 |
| Twf2 | 1431292_a_ -0.9200451 | | 10.0952637 | -8.6203422 | 2.26E-05 | 0.00037586 | 2.65071227 |
| Ifitm1 | 1424254_at -0.9200034 | | 9.60371224 | -6.3278451 | 0.00020828 | 0.0018296 | 0.21875009 |
| Jpt1 | 1438988_x_a-0.9199286 | | 8.76326258 | -12.396211 | 1.42E-06 | 5.40E-05 | 5.67553903 |
| Gna13 | 1430295_at -0.9193219 | | 8.45151113 | -13.267997 | 8.33E-07 | 3.68E-05 | 6.25130375 |
| Clic4 | 1423392_at -0.9180843 | | 11.657903 | -12.395496 | 1.42E-06 | 5.40E-05 | 5.67505146 |
| Tbxas1 | 1416827_at -0.9179614 | | 8.51359113 | -7.4153779 | 6.80E-05 | 0.00079991 | 1.44357696 |
| Stk24 | 1426247_at -0.9173486 | | 8.61682633 | -7.8371498 | 4.55E-05 | 0.00060433 | 1.882697 |
| Col1a1 | 1423669_at -0.917101 | | 10.4536707 | -3.9545659 | 0.00405096 | 0.01704679 | -2.9933759 |
| Il18r1 | 1421628_at -0.916977 | | 8.17650857 | -8.4185317 | 2.69E-05 | 0.00042686 | 2.45840515 |
| Snx6 | 1425148_a_ -0.9167022 | | 9.48926481 | -8.0958378 | 3.59E-05 | 0.00051338 | 2.14294822 |
| Tnip1 | 1427689_a_ -0.9141017 | | 10.0932341 | -9.2714331 | 1.31E-05 | 0.00025759 | 3.24656218 |
| Ptpn2 | 1417140_a_ -0.9132127 | | 8.96541915 | -12.724957 | 1.15E-06 | 4.68E-05 | 5.89708996 |
| Ankrd1 | 1420991_at -0.9128596 | | 13.9395032 | -10.623239 | 4.66E-06 | 0.00012507 | 4.37640247 |
| NA | 1425407_s_a-0.9125366 | | 8.13617951 | -4.2608172 | 0.00264 | 0.01228247 | -2.5352259 |
| Plekha2 | 1417288_at -0.9111516 | | 10.4908423 | -8.8633313 | 1.84E-05 | 0.00032368 | 2.8773744 |
| Hdac1 | 1448246_at -0.9106097 | | 9.41683739 | -11.625563 | 2.33E-06 | 7.62E-05 | 5.13356074 |
| Apobr | 1420382_at -0.9105684 | | 8.10049329 | -7.6561839 | 5.39E-05 | 0.00067913 | 1.6965857 |
| Emilin1 | 1416414_at -0.910074 | | 9.23170604 | -14.29823 | 4.63E-07 | 2.42E-05 | 6.88615309 |
| Batf | 1419410_at -0.90973 | | 8.56915304 | -14.084774 | 5.21E-07 | 2.64E-05 | 6.75839272 |
| Tor3a | 1421998_at -0.9092342 | | 8.35786923 | -12.645261 | 1.21E-06 | 4.85E-05 | 5.84388721 |
| Cmtm6 | 1423792_a_ -0.9088966 | | 9.19884795 | -9.517052 | 1.08E-05 | 0.00022504 | 3.46212752 |
| Foxn2 | 1454831_at -0.9082061 | | 8.24379159 | -11.191115 | 3.13E-06 | 9.53E-05 | 4.81304214 |
| Mob1a | 1424483_at -0.9070312 | | 9.57534644 | -6.1050136 | 0.00026644 | 0.00220074 | -0.0500847 |
| Elk3 | 1448797_at -0.9062942 | | 9.41215479 | -6.2796791 | 0.00021955 | 0.00190707 | 0.16118142 |
| March5 | 1428843_at -0.9061335 | | 11.5741008 | -10.916693 | 3.78E-06 | 0.00010852 | 4.60462567 |
| Ece2 | 1423358_at -0.9060511 | | 9.02826152 | -12.315295 | 1.49E-06 | 5.59E-05 | 5.62014977 |
| Cdc20 | 1416664_at -0.9058219 | | 8.38864811 | -6.7440821 | 0.00013359 | 0.00131785 | 0.704142 |
| P2rx4 | 1425525_a_ -0.9056105 | | 9.6519509 | -7.073199 | 9.53E-05 | 0.0010277 | 1.07312396 |
| Sh3bgrl | 1421871_at -0.9048441 | | 9.35411445 | -7.7166953 | 5.10E-05 | 0.00065204 | 1.75919611 |
| Ak2 | 1448450_at -0.9042687 | | 9.43573786 | -6.0017078 | 0.00029928 | 0.00240295 | -0.1769092 |
| Dctn6 | 1448368_at -0.9034103 | | 10.1977864 | -8.7422974 | 2.03E-05 | 0.00034803 | 2.765129 |
| Prc1 | 1423774_a_ -0.9030962 | | 7.58222484 | -6.1556606 | 0.0002518 | 0.00210589 | 0.0115821 |
| Resf1 | 1437110_at -0.902982 | | 9.61092918 | -5.008554 | 0.00098332 | 0.00572826 | -1.4702274 |
| Rab29 | 1451362_at -0.9018009 | | 9.26262716 | -11.88957 | 1.96E-06 | 6.80E-05 | 5.32293645 |
| Lpin2 | 1452836_at -0.9017506 | | 9.31219131 | -7.9621492 | 4.05E-05 | 0.00055966 | 2.00928945 |
| Cpne2 | 1424831_at -0.90167 | | 8.01236519 | -7.6324956 | 5.52E-05 | 0.0006887 | 1.67197104 |
| Mitd1 | 1426133_a_ -0.9014698 | | 7.75695246 | -10.944713 | 3.71E-06 | 0.00010723 | 4.62612395 |
| Tmod3 | 1423088_at -0.9010454 | | 9.56883877 | -5.736455 | 0.00040591 | 0.00296337 | -0.5090526 |
| Rmdn3 | 1452704_at -0.9007591 | | 8.88551658 | -9.3033718 | 1.28E-05 | 0.00025281 | 3.27486855 |
| Clec4n | 1425951_a_ -0.8999895 | | 7.26811671 | -5.751062 | 0.00039906 | 0.00293698 | -0.4905155 |

| Fstl1 | 1448259_at | -0.8987821 | 11.2913252 | -6.8151984 | 0.00012407 | 0.00124896 | 0.78495548 |
| --- | --- | --- | --- | --- | --- | --- | --- |
| Ptbp1 | 1424874_a_ | -0.8985758 | 10.5552144 | -5.7776192 | 0.00038693 | 0.00287818 | -0.4568871 |
| Vmp1 | 1423722_at | -0.8980904 | 10.6060349 | -8.701146 | 2.11E-05 | 0.0003567 | 2.72667038 |
| Daxx | 1419026_at | -0.897131 | 9.46661297 | -14.435202 | 4.29E-07 | 2.31E-05 | 6.96714371 |
| Cdca3 | 1452040_a_ | -0.8965035 | 7.85625496 | -11.661461 | 2.27E-06 | 7.50E-05 | 5.15954513 |
| Gng12 | 1421947_at | -0.8960765 | 9.29969002 | -10.798398 | 4.11E-06 | 0.00011425 | 4.51330387 |
| Fgd2 | 1419515_at | -0.8960762 | 8.22268762 | -7.2582959 | 7.93E-05 | 0.00089533 | 1.27514097 |
| Tcrb-J | 1427656_at | -0.8937121 | 7.97164725 | -4.6800392 | 0.0015023 | 0.00793464 | -1.9284782 |
| Lrrc59 | 1416234_at | -0.8922314 | 10.3746095 | -11.594277 | 2.38E-06 | 7.73E-05 | 5.11085347 |
| D16Ertd472e | 1429897_a_ | -0.8921361 | 7.88075788 | -8.6598042 | 2.18E-05 | 0.00036605 | 2.68788118 |
| Cks1b | 1448441_at | -0.8919679 | 8.24160801 | -9.0365325 | 1.59E-05 | 0.00029331 | 3.03578248 |
| Tmem167 | 1425780_a_ | -0.8917867 | 9.83042456 | -11.793165 | 2.09E-06 | 7.07E-05 | 5.25424387 |
| Birc2 | 1418854_at | -0.8916549 | 9.5028406 | -10.552119 | 4.91E-06 | 0.00012878 | 4.3202328 |
| Pdlim1 | 1416554_at | -0.8912458 | 11.2377452 | -8.8259859 | 1.89E-05 | 0.00033168 | 2.84287814 |
| Grb2 | 1449111_a_ | -0.8910506 | 10.1121165 | -8.999834 | 1.64E-05 | 0.0002983 | 3.00243342 |
| Sh3bp5 | 1421923_at | -0.8902238 | 10.0851131 | -9.8399326 | 8.36E-06 | 0.00018782 | 3.73828598 |
| Ebp | 1416667_at | -0.8899186 | 9.5190805 | -8.9995209 | 1.64E-05 | 0.0002983 | 3.00214844 |
| Ngdn | 1438318_x_a-0.8891571 | | 10.5766625 | -11.187755 | 3.13E-06 | 9.53E-05 | 4.81051835 |
| Cdc6 | 1417019_a_ -0.8889667 | | 7.0542944 | -13.46164 | 7.43E-07 | 3.40E-05 | 6.37422972 |
| Sectm1a | 1425002_at -0.8875756 | | 7.32676323 | -10.028305 | 7.24E-06 | 0.0001704 | 3.89575635 |
| Rab8a | 1418692_at -0.886939 | | 10.085602 | -8.9985488 | 1.64E-05 | 0.0002983 | 3.00126347 |
| Slbp | 1460168_at -0.8867322 | | 9.20027219 | -6.0271114 | 0.00029081 | 0.00235186 | -0.1455919 |
| Ncaph | 1436707_x_a-0.8862029 | | 8.19275552 | -10.885188 | 3.87E-06 | 0.0001096 | 4.58039269 |
| Cttnbp2nl | 1449300_at -0.8855372 | | 8.80968257 | -10.262539 | 6.07E-06 | 0.00014903 | 4.08795689 |
| Gnai2 | 1419449_a_ -0.8854783 | | 11.1471484 | -6.2705377 | 0.00022177 | 0.00191764 | 0.15022203 |
| NA | 1428262_s_a-0.8852783 | | 10.7536425 | -8.5276091 | 2.45E-05 | 0.00039766 | 2.5628111 |
| Tubb6 | 1416431_at -0.8849993 | | 9.98761405 | -8.7097481 | 2.09E-05 | 0.00035488 | 2.73472209 |
| NA | 1423103_at -0.8844888 | | 8.66562582 | -7.6608624 | 5.37E-05 | 0.00067791 | 1.70144017 |
| Ywhah | 1416004_at -0.8839571 | | 10.9480474 | -8.2242454 | 3.20E-05 | 0.00047914 | 2.2696734 |
| Stx6 | 1460004_x_a-0.8835418 | | 8.79835892 | -6.2778218 | 0.00022 | 0.00190964 | 0.15895557 |
| Mcm7 | 1416030_a_ -0.8830485 | | 8.71347304 | -6.471488 | 0.00017827 | 0.00163898 | 0.38868879 |
| Clta | 1434540_a_ -0.8825393 | | 10.6740976 | -6.4118025 | 0.00019012 | 0.0017101 | 0.31839265 |
| NA | 1427820_at -0.8823114 | | 8.51501467 | -6.2744897 | 0.00022081 | 0.00191269 | 0.15496132 |
| Ptpn2 | 1438562_a_ -0.8821685 | | 9.87704505 | -10.891807 | 3.85E-06 | 0.0001096 | 4.58548902 |
| Rac1 | 1423734_at -0.8819947 | | 11.2682542 | -8.5776773 | 2.34E-05 | 0.000386 | 2.61036791 |
| Nono | 1415820_x_a-0.8819774 | | 10.1921258 | -6.4017879 | 0.00019219 | 0.00172637 | 0.30655388 |
| Sri | 1450878_at -0.8811258 | | 9.97115372 | -7.9248829 | 4.20E-05 | 0.00057395 | 1.9717136 |
| Aida | 1423870_at -0.879402 | | 10.3686971 | -9.1629042 | 1.43E-05 | 0.0002734 | 3.14974939 |
| Faim | 1418029_at -0.8791667 | | 8.08061071 | -7.6474477 | 5.44E-05 | 0.00068191 | 1.68751466 |
| Ptma | 1423455_at -0.8782369 | | 12.5708638 | -9.5902542 | 1.02E-05 | 0.00021575 | 3.52544385 |
| Cntd1 | 1439405_x_a-0.8781528 | | 8.7991856 | -10.392663 | 5.52E-06 | 0.00013874 | 4.1930484 |
| Vrk2 | 1453343_s_a-0.8778224 | | 8.0875543 | -6.4535872 | 0.00018174 | 0.00166275 | 0.36765253 |
| Irf2 | 1418265_s_a-0.8770169 | | 7.99671833 | -5.8802209 | 0.00034372 | 0.00265185 | -0.3278612 |
| Pde4b | 1422474_at -0.8766141 | | 9.76393049 | -9.6386973 | 9.78E-06 | 0.00021046 | 3.56711461 |
| Sppl2a | 1452225_at -0.8763742 | | 10.2209777 | -7.7468818 | 4.95E-05 | 0.00064029 | 1.79028688 |
| Coro2a | 1452367_at -0.8759967 | | 7.32051391 | -7.45301 | 6.55E-05 | 0.00078015 | 1.48352721 |
| Rnpep | 1451243_at -0.8756725 | | 9.31450763 | -9.2868649 | 1.29E-05 | 0.00025488 | 3.26024938 |
| Ezh2 | 1416544_at -0.8756095 | | 7.70066938 | -10.576919 | 4.82E-06 | 0.00012753 | 4.33985813 |
| Gla | 1418248_at -0.8750835 | | 7.31219608 | -8.5082835 | 2.49E-05 | 0.00040236 | 2.54439343 |
| Gpm6b | 1423091_a_ -0.8750294 | | 8.60347969 | -7.7290196 | 5.04E-05 | 0.00064744 | 1.771901 |
| Yy1 | 1422569_at -0.8745613 | | 9.61722245 | -8.8914559 | 1.79E-05 | 0.00031806 | 2.90327304 |
| App | 1420621_a_ -0.8745337 | | 10.6556783 | -5.4276101 | 0.00058561 | 0.00388637 | -0.9078431 |
| Trim10 | 1419311_at -0.8745309 | | 8.1285323 | -3.7972452 | 0.00507421 | 0.02028434 | -3.2332662 |
| Lgals1 | 1455439_a_ -0.8737674 | | 12.9336734 | -13.88928 | 5.82E-07 | 2.83E-05 | 6.63969607 |
| Cflar | 1425686_at -0.8736898 | | 8.55414129 | -7.6693708 | 5.33E-05 | 0.0006746 | 1.71026269 |
| Sp100 | 1451821_a_ -0.8732735 | | 8.68837896 | -9.3372084 | 1.24E-05 | 0.00024806 | 3.30476617 |
| St6galnac4 | 1418075_at -0.8731696 | | 8.89004376 | -12.402932 | 1.41E-06 | 5.39E-05 | 5.68012436 |
| Caprin1 | 1416461_at -0.8728074 | | 11.0767717 | -10.749007 | 4.26E-06 | 0.00011719 | 4.47490531 |
| Phgdh | 1437621_x_a-0.8723464 | | 7.86626388 | -7.8328958 | 4.57E-05 | 0.00060637 | 1.87836087 |
| NA | 1431213_a_ -0.8722919 | | 8.95240126 | -3.1941643 | 0.01240906 | 0.04070574 | -4.1763887 |
| Acp2 | 1424655_at -0.8713523 | | 9.07141148 | -5.7380357 | 0.00040516 | 0.00296164 | -0.5070452 |
| Haspin | 1450886_at -0.8702191 | | 6.99058083 | -11.380097 | 2.75E-06 | 8.67E-05 | 4.95385533 |
| Smc4 | 1427276_at -0.8690758 | | 7.76890167 | -9.8233045 | 8.47E-06 | 0.00018894 | 3.72425865 |
| Tacc3 | 1417450_a_ -0.8685956 | | 7.30319927 | -6.9548478 | 0.00010749 | 0.00111773 | 0.94189982 |
| Nras | 1422688_a_ -0.8681343 | | 8.47232264 | -10.215946 | 6.29E-06 | 0.00015258 | 4.05003788 |
| Pdcd10 | 1448527_at -0.8675306 | | 9.95552394 | -10.057586 | 7.08E-06 | 0.00016788 | 3.91999897 |
| Usp1 | 1451080_at -0.8675232 | | 8.86771962 | -10.222223 | 6.26E-06 | 0.00015229 | 4.05515515 |
| NA | 1453473_a_ -0.8664813 | | 10.0550156 | -9.0537747 | 1.57E-05 | 0.0002903 | 3.05141147 |
| Tpx2 | 1428104_at -0.8661737 | | 7.42731558 | -9.0549473 | 1.56E-05 | 0.0002903 | 3.05247352 |
| Smap1 | 1423956_at -0.8661408 | | 9.09635626 | -10.689721 | 4.45E-06 | 0.00011994 | 4.42860264 |
| Ywhaz | 1448218_s_a-0.8652635 | | 10.9121556 | -6.196051 | 0.00024076 | 0.00203304 | 0.06052169 |
| Marcksl1 | 1435627_x_a-0.8643771 | | 8.69797351 | -9.6182433 | 9.93E-06 | 0.00021265 | 3.54954234 |
| Msr1 | 1425434_a_ -0.8638893 | | 8.03107111 | -10.868914 | 3.91E-06 | 0.00011044 | 4.56784968 |
| Cdc25b | 1421963_a_ -0.8636882 | | 8.25382234 | -9.6075414 | 1.00E-05 | 0.00021385 | 3.54033527 |
| Spg21 | 1451036_at -0.8634913 | | 8.77278276 | -5.8263037 | 0.00036573 | 0.00276734 | -0.3954875 |
| Snrnp40 | 1452713_a_ -0.8634757 | | 8.54514153 | -11.090229 | 3.35E-06 | 0.00010037 | 4.7369712 |
| Jak2 | 1421065_at -0.8633844 | | 8.54482831 | -9.4025204 | 1.18E-05 | 0.00023927 | 3.36221312 |
| Prkar1a | 1425550_a_ -0.8627158 | | 11.5014689 | -10.523486 | 5.01E-06 | 0.00013079 | 4.29752259 |
| Prcp | 1452190_at -0.8624238 | | 8.68755641 | -5.8427423 | 0.00035886 | 0.00272829 | -0.3748278 |
| Lmo2 | 1454086_a_ -0.8618452 | | 9.96772679 | -11.62598 | 2.33E-06 | 7.62E-05 | 5.13386287 |
| Tes | 1460378_a_ -0.8617585 | | 8.65106517 | -8.3302237 | 2.91E-05 | 0.00044874 | 2.37306554 |
| Myc | 1424942_a_ -0.8617583 | | 8.04123906 | -10.239026 | 6.18E-06 | 0.00015076 | 4.06884031 |
| Trip13 | 1429295_s_a-0.8612267 | | 7.09642444 | -12.387824 | 1.42E-06 | 5.42E-05 | 5.66981414 |
| Lasp1 | 1460173_at -0.8603212 | | 9.67359695 | -4.7469012 | 0.0013764 | 0.00742344 | -1.833973 |
| Lamp2 | 1416344_at -0.8594043 | | 9.98540124 | -5.7250731 | 0.00041134 | 0.0029924 | -0.523517 |

| Rbms1 | 1434005_at | -0.8593504 | 10.6064993 | -7.97487 | 4.01E-05 | 0.00055586 | 2.02208403 |
| --- | --- | --- | --- | --- | --- | --- | --- |
| Junb | 1415899_at | -0.8590243 | 9.03185945 | -4.5817813 | 0.00171055 | 0.0087811 | -2.0685012 |
| Nampt | 1417190_at | -0.8585438 | 10.8422966 | -9.9410664 | 7.74E-06 | 0.00017785 | 3.82315585 |
| Rgs16 | 1426037_a_ | -0.8585206 | 6.97552358 | -7.508799 | 6.21E-05 | 0.00075029 | 1.54247036 |
| Sap30 | 1417719_at | -0.8579473 | 9.3597102 | -14.18497 | 4.93E-07 | 2.53E-05 | 6.81859965 |
| Gars | 1423784_at | -0.8577499 | 11.0005487 | -11.860944 | 1.99E-06 | 6.89E-05 | 5.30259399 |
| Pik3cd | 1422992_s_a-0.857294 | | 7.35611502 | -5.5568663 | 0.00050155 | 0.00345376 | -0.7393431 |
| Cdc42 | 1449574_a_ -0.8567103 | | 11.6818523 | -9.4359784 | 1.15E-05 | 0.00023442 | 3.39150907 |
| Lrrc8c | 1423614_at -0.8560306 | | 8.55703125 | -6.8901724 | 0.00011484 | 0.00117866 | 0.86950058 |
| Mtmr6 | 1456540_s_a-0.855423 | | 9.04035425 | -9.1982107 | 1.39E-05 | 0.00026743 | 3.18135127 |
| Ets1 | 1452163_at -0.8550237 | | 10.528117 | -5.5889973 | 0.00048277 | 0.00335394 | -0.6978156 |
| Cxcr4 | 1448710_at -0.8546208 | | 7.59885903 | -4.4600623 | 0.00201295 | 0.0099681 | -2.2438285 |
| Ptpn12 | 1455105_at -0.8544613 | | 9.87691953 | -13.850444 | 5.94E-07 | 2.87E-05 | 6.61592075 |
| Ptbp3 | 1424084_at -0.854295 | | 6.92487703 | -10.790468 | 4.14E-06 | 0.00011443 | 4.50714957 |
| Rbbp8 | 1427061_at -0.8542018 | | 8.55740912 | -8.831851 | 1.89E-05 | 0.00033055 | 2.84830386 |
| Notch2 | 1455556_at -0.853379 | | 9.4171189 | -5.6797201 | 0.00043377 | 0.00310823 | -0.5813279 |
| Nubp1 | 1418905_at -0.8533126 | | 8.83454181 | -12.754776 | 1.13E-06 | 4.63E-05 | 5.91691459 |
| Bmp2k | 1437419_at -0.8528549 | | 7.19546597 | -9.4536856 | 1.13E-05 | 0.00023301 | 3.40697737 |
| Inpp1 | 1418045_at -0.852252 | | 8.73881332 | -11.446732 | 2.63E-06 | 8.39E-05 | 5.00299202 |
| Cflar | 1449317_at -0.8522407 | | 8.68571638 | -9.3041117 | 1.28E-05 | 0.00025281 | 3.27552327 |
| Tlr4 | 1418163_at -0.8520356 | | 8.09575277 | -8.60368 | 2.29E-05 | 0.00037935 | 2.63497597 |
| Eif4e3 | 1417977_at -0.8514377 | | 11.2933802 | -8.3136066 | 2.95E-05 | 0.00045315 | 2.35692473 |
| Gypa | 1425643_at -0.8512107 | | 8.12985328 | -3.0415657 | 0.01566794 | 0.04876673 | -4.4192888 |
| Edem1 | 1451218_at -0.8509958 | | 9.21987056 | -10.993778 | 3.58E-06 | 0.00010494 | 4.66364961 |
| Ly96 | 1449874_at -0.8509531 | | 7.98296268 | -10.597951 | 4.75E-06 | 0.00012618 | 4.3564693 |
| Lrwd1 | 1437194_x_a-0.8509242 | | 8.4751349 | -8.1556073 | 3.40E-05 | 0.00049618 | 2.20213416 |
| Cyp4v3 | 1417071_s_a-0.8507768 | | 8.58725046 | -5.9081896 | 0.00033288 | 0.00259291 | -0.2929347 |
| Parvg | 1416875_at -0.850163 | | 8.35355747 | -4.4850822 | 0.00194636 | 0.00970827 | -2.2076211 |
| Ak2 | 1448451_at -0.8499659 | | 10.4318948 | -8.9617413 | 1.69E-05 | 0.00030562 | 2.96769574 |
| Eif1a | 1424344_s_a-0.8499201 | | 8.42210291 | -12.866656 | 1.06E-06 | 4.42E-05 | 5.99090117 |
| Atp7a | 1418774_a_ -0.8494593 | | 7.87026187 | -8.9997309 | 1.64E-05 | 0.0002983 | 3.00233963 |
| Tln1 | 1448402_at -0.8492558 | | 10.7262755 | -5.0225424 | 0.00096607 | 0.00566034 | -1.4510544 |
| Acot9 | 1418073_at -0.8480909 | | 10.090802 | -9.3355463 | 1.24E-05 | 0.00024806 | 3.30329979 |
| Aspm | 1422814_at -0.8479876 | | 7.86334841 | -7.8069024 | 4.68E-05 | 0.00061655 | 1.85182522 |
| Rasa3 | 1415850_at -0.8479528 | | 9.05471199 | -8.9793311 | 1.67E-05 | 0.0003019 | 2.98375183 |
| Shcbp1 | 1416299_at -0.8477873 | | 7.79973188 | -8.0056835 | 3.90E-05 | 0.00054432 | 2.05300907 |
| Prc1 | 1423775_s_a-0.8477163 | | 8.36509004 | -6.4142732 | 0.00018961 | 0.00170794 | 0.32131151 |
| Ywhaz | 1448219_a_ -0.8476504 | | 11.7238281 | -9.0123032 | 1.62E-05 | 0.0002961 | 3.01377749 |
| Ada | 1417976_at -0.8472077 | | 7.3703873 | -8.1639777 | 3.37E-05 | 0.00049436 | 2.21039501 |
| Ctsa | 1448128_at -0.8470285 | | 10.826993 | -7.3009127 | 7.60E-05 | 0.00086671 | 1.32110774 |
| Mcm4 | 1436708_x_a-0.8469842 | | 8.24194981 | -7.2596881 | 7.92E-05 | 0.00089454 | 1.27664583 |
| Dnmt1 | 1422946_a_ -0.8461843 | | 9.42468402 | -6.1911778 | 0.00024206 | 0.00204176 | 0.05462825 |
| Nras | 1422687_at -0.8457176 | | 9.56761838 | -9.2692191 | 1.31E-05 | 0.00025759 | 3.24459686 |
| Pitpnm1 | 1437724_x_a-0.8456187 | | 9.42408655 | -6.6805254 | 0.00014277 | 0.00139335 | 0.63140421 |
| Itgav | 1452784_at -0.8453736 | | 10.2719386 | -7.3553248 | 7.21E-05 | 0.00083427 | 1.37950436 |
| Nts | 1422860_at -0.845191 | | 8.24770739 | -6.3536758 | 0.0002025 | 0.00179015 | 0.24950158 |
| Pfkp | 1430634_a_ -0.8448648 | | 8.66064342 | -8.2922735 | 3.01E-05 | 0.0004585 | 2.33616468 |
| Actn1 | 1428585_at -0.8447816 | | 10.0958534 | -10.798887 | 4.11E-06 | 0.00011425 | 4.51368358 |
| Rap2b | 1417914_at -0.8441252 | | 9.35597067 | -7.921084 | 4.21E-05 | 0.00057562 | 1.96787527 |
| NA | 1439255_s_a-0.8438112 | | 8.08046003 | -6.5558605 | 0.00016289 | 0.00153106 | 0.48730264 |
| Psmg4 | 1435431_at -0.8435133 | | 9.56675296 | -7.7998457 | 4.71E-05 | 0.00061815 | 1.84460937 |
| Ogfrl1 | 1424413_at -0.8433671 | | 8.29319528 | -7.0192309 | 0.00010068 | 0.0010685 | 1.01348686 |
| Sla | 1420818_at -0.8428606 | | 8.28419681 | -6.3238547 | 0.00020919 | 0.00183475 | 0.21399192 |
| Slc2a1 | 1426600_at -0.8425746 | | 8.9866144 | -6.8354693 | 0.0001215 | 0.0012329 | 0.80787996 |
| Rnh1 | 1451201_s_a-0.8425082 | | 10.3807401 | -10.615973 | 4.69E-06 | 0.00012543 | 4.37067971 |
| Atp6v1a | 1422508_at -0.8411894 | | 9.28538504 | -5.626981 | 0.00046155 | 0.00325428 | -0.6489072 |
| Mcm3 | 1426652_at -0.8403326 | | 7.72661296 | -8.1714188 | 3.35E-05 | 0.00049361 | 2.21773304 |
| Cd38 | 1450136_at -0.8403183 | | 7.76018873 | -6.4742832 | 0.00017774 | 0.0016352 | 0.39196995 |
| Soat1 | 1417697_at -0.8400912 | | 8.24124696 | -6.3175154 | 0.00021064 | 0.00184259 | 0.20642886 |
| Tram1 | 1423732_at -0.8389932 | | 10.5852853 | -7.556857 | 5.93E-05 | 0.00072497 | 1.59297703 |
| Prkd2 | 1434333_a_ -0.8389161 | | 7.87005099 | -9.0344327 | 1.59E-05 | 0.00029331 | 3.03387748 |
| Pqlc3 | 1424906_at -0.8387764 | | 8.19914385 | -7.7539837 | 4.92E-05 | 0.00063749 | 1.79758771 |
| Sppl2a | 1425193_at -0.835935 | | 9.96692452 | -10.879521 | 3.88E-06 | 0.00010975 | 4.57602719 |
| Gmip | 1428784_at -0.8359328 | | 8.26615591 | -7.0500425 | 9.76E-05 | 0.00104354 | 1.04757607 |
| Lamtor5 | 1436152_a_ -0.8358687 | | 9.99526675 | -9.014256 | 1.62E-05 | 0.00029585 | 3.01555283 |
| Txndc17 | 1423035_s_a-0.8356434 | | 10.4177667 | -9.2561395 | 1.33E-05 | 0.00025906 | 3.23297836 |
| Rad51 | 1418281_at -0.8347152 | | 7.40390207 | -8.3500628 | 2.86E-05 | 0.00044246 | 2.39230181 |
| Morc3 | 1452224_at -0.8346135 | | 8.11909386 | -7.5634111 | 5.89E-05 | 0.0007216 | 1.59984596 |
| Mcl1 | 1416881_at -0.8342857 | | 10.5006427 | -9.6190681 | 9.93E-06 | 0.00021265 | 3.55025157 |
| Hexa | 1449024_a_ -0.8342668 | | 10.7631778 | -3.735838 | 0.00554573 | 0.02173542 | -3.3276812 |
| Rrm1 | 1415878_at -0.8339876 | | 8.80862019 | -9.2134246 | 1.37E-05 | 0.00026503 | 3.194937 |
| Sdc4 | 1448793_a_ -0.8316615 | | 8.55653777 | -7.5251316 | 6.11E-05 | 0.00074032 | 1.55966295 |
| Rasa4 | 1417333_at -0.8311829 | | 7.83616991 | -4.9479421 | 0.00106205 | 0.00608073 | -1.5536252 |
| Cxcl12 | 1448823_at -0.8310828 | | 9.58408788 | -8.4376105 | 2.65E-05 | 0.00042254 | 2.47674656 |
| Serp1 | 1415827_a_ -0.8305904 | | 10.5483394 | -7.3783021 | 7.05E-05 | 0.00081952 | 1.40406626 |
| Prkcq | 1426044_a_ -0.8305476 | | 9.05042511 | -6.7958623 | 0.00012658 | 0.00126526 | 0.76304271 |
| Fyn | 1417558_at -0.8292947 | | 10.0967197 | -6.1291941 | 0.00025933 | 0.00215383 | -0.0206013 |
| Sppl2a | 1450459_at -0.828572 | | 9.74286482 | -8.5123948 | 2.48E-05 | 0.0004015 | 2.54831445 |
| Nhp2 | 1416605_at -0.82857 | | 8.93226435 | -8.7293209 | 2.06E-05 | 0.00035066 | 2.75301795 |
| NA | 1428116_a_ -0.827676 | | 11.2079498 | -9.5955886 | 1.01E-05 | 0.00021551 | 3.53004149 |
| Peli1 | 1417371_at -0.8270492 | | 9.66102366 | -7.5396073 | 6.03E-05 | 0.00073349 | 1.57487689 |
| Slain1 | 1424824_at -0.8268264 | | 6.93189613 | -6.8089849 | 0.00012487 | 0.00125571 | 0.77791887 |
| Dek | 1452659_at -0.8262268 | | 10.566019 | -10.699837 | 4.41E-06 | 0.00011964 | 4.4365195 |
| Vps26c | 1415745_a_ -0.8261744 | | 9.80015627 | -11.158435 | 3.20E-06 | 9.66E-05 | 4.78846972 |
| NA | 1425451_s_a-0.8258218 | | 7.87789594 | -8.9315258 | 1.73E-05 | 0.00031047 | 2.9400529 |

| Usp25 | 1448939_at | -0.8254844 | 9.54152596 | -9.9280539 | 7.81E-06 | 0.0001789 | 3.81227868 |
| --- | --- | --- | --- | --- | --- | --- | --- |
| Nab1 | 1417624_at | -0.825444 | 10.4267309 | -9.5953495 | 1.01E-05 | 0.00021551 | 3.52983544 |
| Tgfbr1 | 1420895_at | -0.8252842 | 9.83563682 | -4.2524317 | 0.00267065 | 0.01238442 | -2.5476076 |
| Adam17 | 1421859_at | -0.8246309 | 8.98961405 | -6.735713 | 0.00013476 | 0.00132653 | 0.69459188 |
| Ppt1 | 1422467_at | -0.8240892 | 9.75081316 | -5.917663 | 0.0003293 | 0.00257471 | -0.2811283 |
| Slc9a3r1 | 1450982_at | -0.823202 | 9.46646963 | -3.3984452 | 0.00911882 | 0.03196635 | -3.8533756 |
| Nmt1 | 1415683_at | -0.8231441 | 11.3146211 | -11.28454 | 2.93E-06 | 9.09E-05 | 4.88292641 |
| Cd160 | 1420066_s_a-0.8224122 | | 7.92952553 | -9.1950863 | 1.39E-05 | 0.00026766 | 3.17855889 |
| Tuba1a | 1418884_x_a-0.8218035 | | 11.9478283 | -12.363224 | 1.45E-06 | 5.48E-05 | 5.65300027 |
| Fcgr2b | 1435476_a_ -0.8212862 | | 10.6185234 | -8.2185323 | 3.21E-05 | 0.00048097 | 2.26406914 |
| Plp2 | 1453572_a_ -0.8209572 | | 10.7423222 | -7.9992407 | 3.92E-05 | 0.00054659 | 2.04655081 |
| Atp13a2 | 1428340_s_a-0.8206555 | | 9.11034108 | -4.4919024 | 0.00192863 | 0.00963464 | -2.1977664 |
| Trp53inp1 | 1416926_at -0.8205424 | | 9.0855787 | -6.475183 | 0.00017757 | 0.0016352 | 0.39302599 |
| Tbrg1 | 1452648_at -0.8202031 | | 10.3451588 | -9.9775797 | 7.52E-06 | 0.00017492 | 3.85361059 |
| Ddx17 | 1437773_x_a-0.8195759 | | 10.8794661 | -7.7809226 | 4.80E-05 | 0.00062481 | 1.82523407 |
| Prkd2 | 1434334_at -0.819389 | | 8.74173854 | -7.4272547 | 6.72E-05 | 0.00079528 | 1.45620196 |
| Ctps2 | 1448111_at -0.8193715 | | 8.70269529 | -7.248384 | 8.01E-05 | 0.00090189 | 1.26442084 |
| Adap2 | 1425638_at -0.8184837 | | 8.05436124 | -6.3489643 | 0.00020354 | 0.00179768 | 0.24389885 |
| Mcam | 1416357_a_ -0.818114 | | 8.91271771 | -8.7238626 | 2.07E-05 | 0.00035195 | 2.74791915 |
| Psma2 | 1448206_at -0.8177986 | | 11.7496866 | -12.337667 | 1.47E-06 | 5.53E-05 | 5.63549901 |
| Washc4 | 1437719_x_a-0.8173514 | | 7.27600955 | -9.263452 | 1.32E-05 | 0.00025798 | 3.23947578 |
| Stat3 | 1460700_at -0.8161317 | | 11.4018169 | -6.7280794 | 0.00013584 | 0.00133425 | 0.68587353 |
| Ccl6 | 1420249_s_a-0.8157271 | | 8.88819794 | -5.4429209 | 0.00057489 | 0.00383768 | -0.8877624 |
| Pabpn1 | 1422849_a_ -0.8149988 | | 10.4526075 | -13.027586 | 9.61E-07 | 4.05E-05 | 6.09624655 |
| Washc4 | 1439450_x_a-0.8141882 | | 7.68927793 | -8.0241284 | 3.83E-05 | 0.00053876 | 2.07147548 |
| Cd28 | 1417597_at -0.8129916 | | 7.94666902 | -8.3670876 | 2.82E-05 | 0.00044002 | 2.40877957 |
| NA | 1454611_a_ -0.8110448 | | 11.8318459 | -9.1523522 | 1.44E-05 | 0.00027496 | 3.14028442 |
| Dok1 | 1417790_at -0.8109141 | | 8.29649424 | -7.0732986 | 9.53E-05 | 0.0010277 | 1.07323362 |
| Snx6 | 1429497_s_a-0.8108981 | | 9.58187871 | -12.038817 | 1.78E-06 | 6.36E-05 | 5.42825733 |
| Ddx39 | 1451065_a_ -0.8105223 | | 8.57327999 | -6.690942 | 0.00014122 | 0.00138057 | 0.64335903 |
| Flnb | 1426750_at -0.8105063 | | 9.00949055 | -6.8060111 | 0.00012526 | 0.00125655 | 0.77454954 |
| Plin2 | 1448318_at -0.8104886 | | 11.2324252 | -5.3969541 | 0.00060774 | 0.00400044 | -0.9481474 |
| Fli1 | 1433512_at -0.8100953 | | 9.1892847 | -6.7780467 | 0.00012894 | 0.00128098 | 0.7428135 |
| Creld2 | 1452754_at -0.8097127 | | 9.38202706 | -6.2635339 | 0.00022348 | 0.00193098 | 0.14181812 |
| Ddx39 | 1455814_x_a-0.8091836 | | 9.08292224 | -10.117535 | 6.77E-06 | 0.00016232 | 3.96943939 |
| Sorl1 | 1426258_at -0.8090645 | | 9.15168856 | -8.7637177 | 2.00E-05 | 0.00034434 | 2.78508809 |
| Plk1 | 1448191_at -0.8090131 | | 7.8290069 | -7.9770673 | 4.00E-05 | 0.00055532 | 2.02429245 |
| Icos | 1421930_at -0.8089322 | | 8.52226597 | -8.2159685 | 3.22E-05 | 0.00048106 | 2.26155316 |
| Srd5a3 | 1423574_s_a-0.8085538 | | 8.00357866 | -7.1317189 | 8.99E-05 | 0.00098593 | 1.13741321 |
| Tgfbr2 | 1426397_at -0.8072533 | | 10.543071 | -5.1153459 | 0.00085964 | 0.0051563 | -1.3245567 |
| Aoah | 1450764_at -0.8072012 | | 7.75687418 | -5.2757144 | 0.00070463 | 0.0044498 | -1.1088337 |
| Rnf114 | 1422959_s_a-0.8068889 | | 9.41617439 | -6.2906453 | 0.00021693 | 0.0018873 | 0.17431437 |
| Grk6 | 1451672_at -0.8058861 | | 9.06335581 | -5.7496199 | 0.00039973 | 0.00293811 | -0.4923443 |
| Tcf19 | 1423809_at -0.8058357 | | 8.25278937 | -7.5938227 | 5.72E-05 | 0.00070782 | 1.63165842 |
| Myl12b | 1428608_at -0.8054695 | | 11.0538042 | -6.585498 | 0.00015784 | 0.00149848 | 0.52173365 |
| Spc25 | 1424118_a_ -0.8048315 | | 7.97100133 | -12.678633 | 1.19E-06 | 4.77E-05 | 5.86620429 |
| Map3k8 | 1419208_at -0.8045128 | | 7.83953419 | -7.0655701 | 9.61E-05 | 0.00103317 | 1.06471405 |
| Ralb | 1435517_x_a-0.8043632 | | 8.7536818 | -10.417863 | 5.41E-06 | 0.00013727 | 4.21326467 |
| Xpo7 | 1439411_a_ -0.8036184 | | 9.0303244 | -7.1620156 | 8.72E-05 | 0.00096211 | 1.17054356 |
| Lmnb1 | 1423520_at -0.8035865 | | 8.05411938 | -8.816413 | 1.91E-05 | 0.00033284 | 2.83401597 |
| Ccm2 | 1434648_a_ -0.8032032 | | 10.305029 | -11.629825 | 2.32E-06 | 7.62E-05 | 5.13664963 |
| Fbxo32 | 1448747_at -0.8028764 | | 9.46917038 | -5.9629718 | 0.00031273 | 0.0024793 | -0.2248269 |
| Vcan | 1427256_at -0.8027347 | | 8.45202109 | -9.615949 | 9.95E-06 | 0.00021265 | 3.54756925 |
| Nucks1 | 1444952_a_ -0.8021706 | | 8.52955383 | -6.6632812 | 0.00014539 | 0.00140793 | 0.61158458 |
| Gnb4 | 1419469_at -0.8021273 | | 8.41806743 | -10.268784 | 6.04E-06 | 0.00014863 | 4.09302752 |
| Rrbp1 | 1452767_at -0.8013101 | | 11.3429248 | -6.8907916 | 0.00011477 | 0.00117866 | 0.87019603 |
| Commd7 | 1426765_at -0.8010636 | | 8.89668856 | -7.0137398 | 0.00010124 | 0.00107346 | 1.0074001 |
| Hpcal1 | 1448812_at -0.8009751 | | 9.90937456 | -8.2296819 | 3.18E-05 | 0.00047808 | 2.27500341 |
| Racgap1 | 1451358_a_ -0.8009562 | | 7.75648025 | -9.3508426 | 1.23E-05 | 0.00024654 | 3.31678694 |
| Loxl3 | 1418269_at -0.8006264 | | 8.90300851 | -7.4970306 | 6.28E-05 | 0.00075749 | 1.53006459 |
| Cnp | 1418980_a_ -0.8005768 | | 8.66744288 | -5.6325911 | 0.0004585 | 0.00324095 | -0.6417004 |
| Klrb1b | 1420421_s_a-0.7993859 | | 7.14072099 | -9.0953287 | 1.51E-05 | 0.00028329 | 3.08897428 |
| Pkib | 1421137_a_ -0.7990193 | | 7.05314852 | -6.5649032 | 0.00016133 | 0.00152018 | 0.49781941 |
| Ccnd2 | 1448229_s_a-0.7988461 | | 9.7228515 | -5.6768508 | 0.00043523 | 0.00311573 | -0.5849947 |
| Eif3e | 1439268_x_a-0.7987601 | | 11.2158056 | -9.6585099 | 9.63E-06 | 0.00020801 | 3.58410498 |
| Sub1 | 1422693_a_ -0.7984878 | | 10.9912696 | -8.9248995 | 1.74E-05 | 0.00031182 | 2.93398025 |
| Csrp1 | 1425810_a_ -0.7984552 | | 8.81856127 | -5.4384484 | 0.000578 | 0.00384938 | -0.8936249 |
| Nudt13 | 1422126_a_ -0.7964863 | | 8.36639716 | -11.025284 | 3.51E-06 | 0.00010332 | 4.68766485 |
| Srsf9 | 1417727_at -0.7961054 | | 9.19475511 | -7.1132594 | 9.16E-05 | 0.00099998 | 1.11717611 |
| Enc1 | 1420965_a_ -0.795872 | | 10.0326977 | -11.17007 | 3.17E-06 | 9.62E-05 | 4.79722524 |
| Prkd2 | 1436589_x_a-0.7951522 | | 7.8458944 | -11.070761 | 3.40E-06 | 0.00010129 | 4.72221893 |
| Itgal | 1425367_at -0.7950732 | | 8.23842606 | -6.3336519 | 0.00020696 | 0.00182226 | 0.22567043 |
| G3bp2 | 1415697_at -0.7947171 | | 9.7846826 | -9.9307387 | 7.80E-06 | 0.00017872 | 3.81452388 |
| Ppp2r5c | 1425542_a_ -0.7946397 | | 9.58045928 | -8.9466498 | 1.71E-05 | 0.00030811 | 2.95389904 |
| Fes | 1427368_x_a-0.7940473 | | 8.95805951 | -5.8763525 | 0.00034525 | 0.00265853 | -0.3327001 |
| Gda | 1422868_s_a-0.7940125 | | 7.50322844 | -5.6600705 | 0.0004439 | 0.00316334 | -0.6064621 |
| Fam220a | 1418389_at -0.7937891 | | 8.9156141 | -2.7627376 | 0.02412676 | 0.06817226 | -4.8647407 |
| Enc1 | 1450061_at -0.7928362 | | 9.30510835 | -10.941278 | 3.72E-06 | 0.00010725 | 4.62349174 |
| Tk1 | 1416258_at -0.7925508 | | 8.58409083 | -6.5457502 | 0.00016465 | 0.0015438 | 0.47553238 |
| Tuba1b | 1423846_x_a-0.792454 | | 13.3082743 | -10.46084 | 5.25E-06 | 0.00013406 | 4.24764021 |
| Creb3 | 1424741_s_a-0.7917097 | | 10.3222809 | -10.016097 | 7.31E-06 | 0.00017163 | 3.88563077 |
| Cdc42se1 | 1428131_a_ -0.791287 | | 9.26451055 | -8.015752 | 3.86E-05 | 0.00054078 | 2.06309353 |
| Scarb2 | 1460235_at -0.7912533 | | 10.9803285 | -7.8689248 | 4.42E-05 | 0.00059265 | 1.91502716 |
| Crybg1 | 1426942_at -0.7910673 | | 7.68792372 | -7.0075828 | 0.00010188 | 0.00107882 | 1.00057108 |
| Prim1 | 1418369_at -0.7905489 | | 8.14423543 | -8.9449487 | 1.71E-05 | 0.00030811 | 2.95234268 |

| Mrpl27 | 1415690_at -0.7903713 | 11.3842073 | -10.105308 | 6.83E-06 | 0.00016348 | 3.95937696 |
| --- | --- | --- | --- | --- | --- | --- |
| Eif4ebp1 | 1434976_x_a-0.7897492 | 11.5708604 | -6.9627634 | 0.00010663 | 0.00111387 | 0.95072718 |
| Znfx1 | 1427091_at -0.7896909 | 9.1784362 | -9.0162685 | 1.62E-05 | 0.00029584 | 3.01738218 |
| Ctsb | 1448732_at -0.7896065 | 12.799064 | -6.379308 | 0.00019693 | 0.00175299 | 0.27993314 |
| Lpin2 | 1452837_at -0.7892046 | 7.56507975 | -6.8158325 | 0.00012399 | 0.00124896 | 0.7856734 |
| Dusp6 | 1415834_at -0.7890241 | 9.78852545 | -6.0810969 | 0.00027367 | 0.00224498 | -0.0793215 |
| Sfpq | 1423795_at -0.7883672 | 10.0771591 | -7.0712959 | 9.55E-05 | 0.00102869 | 1.07102658 |
| Dynll1 | 1448682_at -0.788207 | 10.9263107 | -7.9178198 | 4.22E-05 | 0.00057662 | 1.96457604 |
| Pglyrp2 | 1420515_a_ -0.7876361 | 7.54072129 | -8.8919611 | 1.79E-05 | 0.00031806 | 2.90373762 |
| Rab7b | 1435830_a_ -0.7872308 | 7.90457851 | -6.0275939 | 0.00029065 | 0.00235186 | -0.1449979 |
| Fam102a | 1426894_s_a-0.7863759 | 8.96810915 | -4.8745215 | 0.00116673 | 0.00654305 | -1.6553448 |
| Rrad | 1422562_at -0.785805 | 11.0199555 | -4.8575784 | 0.00119245 | 0.00664855 | -1.678927 |
| Stard3nl | 1430274_a_ -0.785717 | 9.33742721 | -7.0029661 | 0.00010236 | 0.00107991 | 0.99544767 |
| Il1rn | 1423017_a_ -0.7849593 | 8.63117228 | -12.824531 | 1.09E-06 | 4.50E-05 | 5.96311636 |
| Jpt1 | 1448180_a_ -0.7845896 | 10.2005646 | -8.0452145 | 3.76E-05 | 0.00053179 | 2.09254471 |
| Jak1 | 1433803_at -0.7841232 | 9.57424952 | -5.9959105 | 0.00030125 | 0.00241365 | -0.184068 |
| Nans | 1417773_at -0.7836199 | 9.14617145 | -10.470018 | 5.21E-06 | 0.00013344 | 4.25496496 |
| Abhd16a | 1460709_a_ -0.7835187 | 10.5751722 | -10.49638 | 5.11E-06 | 0.00013236 | 4.27597242 |
| Leprot | 1436900_x_a-0.7831857 | 9.41381091 | -8.0893219 | 3.61E-05 | 0.00051556 | 2.13647475 |
| Bin1 | 1425532_a_ -0.7831674 | 8.14493405 | -5.3335407 | 0.00065646 | 0.0042412 | -1.031936 |
| Vps4b | 1417007_a_ -0.7823806 | 10.3215969 | -9.6539748 | 9.66E-06 | 0.00020855 | 3.58021856 |
| Elovl1 | 1425676_a_ -0.7823717 | 9.43672564 | -9.6472118 | 9.71E-06 | 0.00020946 | 3.57441993 |
| NA | 1426092_a_ -0.7819397 | 6.69262732 | -12.080174 | 1.73E-06 | 6.26E-05 | 5.45722461 |
| Zfp36l2 | 1437626_at -0.7818691 | 10.5310794 | -4.9781914 | 0.00102193 | 0.00590468 | -1.5119392 |
| Arid5b | 1420973_at -0.7818571 | 8.16406608 | -6.4517098 | 0.00018211 | 0.00166478 | 0.36544393 |
| Txndc17 | 1439184_s_a-0.7811072 | 10.6837438 | -10.668902 | 4.51E-06 | 0.00012132 | 4.41228761 |
| Morc3 | 1420091_s_a-0.7808504 | 9.00995297 | -7.9326665 | 4.17E-05 | 0.00057122 | 1.97957337 |
| Mtpn | 1420473_at -0.7807576 | 10.7100877 | -8.2054598 | 3.25E-05 | 0.00048313 | 2.25123386 |
| Rbpj | 1448957_at -0.7806084 | 9.48732337 | -6.3833333 | 0.00019607 | 0.00174871 | 0.28470462 |
| Rbm7 | 1451237_s_a-0.7802729 | 9.85677556 | -8.0088007 | 3.89E-05 | 0.00054317 | 2.05613228 |
| Hcst | 1419119_at -0.780021 | 8.44349625 | -10.059638 | 7.07E-06 | 0.00016779 | 3.9216962 |
| NA | 1417451_a_ -0.7798434 | 13.0691539 | -9.7648081 | 8.86E-06 | 0.00019557 | 3.67474584 |
| Ywhaq | 1420830_x_a-0.7797624 | 11.0600301 | -9.1210774 | 1.48E-05 | 0.00027919 | 3.11217689 |
| Lrrk1 | 1451986_s_a-0.779556 | 8.20176466 | -7.9089366 | 4.26E-05 | 0.00057999 | 1.95559208 |
| Cdipt | 1436715_s_a-0.7795479 | 10.0375615 | -10.388611 | 5.53E-06 | 0.000139 | 4.18979356 |
| Rfc5 | 1452917_at -0.7790197 | 8.03464422 | -9.2161612 | 1.37E-05 | 0.00026503 | 3.19737866 |
| Cemip2 | 1424711_at -0.7789985 | 8.30619935 | -8.2382226 | 3.16E-05 | 0.00047507 | 2.28337106 |
| Eif3e | 1434523_x_a-0.7789904 | 11.2805978 | -11.522952 | 2.49E-06 | 8.07E-05 | 5.05887162 |
| Ccnd3 | 1415907_at -0.7787443 | 10.244297 | -6.1481347 | 0.00025392 | 0.00212048 | 0.00243986 |
| Ywhaz | 1439005_x_a-0.7786242 | 8.69177922 | -8.2847458 | 3.03E-05 | 0.00046103 | 2.32882877 |
| Taok3 | 1448019_at -0.778053 | 8.04910021 | -7.3787473 | 7.04E-05 | 0.00081952 | 1.40454165 |
| Cd302 | 1448919_at -0.7779596 | 9.15860621 | -6.3631977 | 0.00020041 | 0.00177496 | 0.26081606 |
| Nedd9 | 1422818_at -0.7778609 | 9.04550392 | -5.025906 | 0.00096197 | 0.00564153 | -1.4464483 |
| Rab4b | 1451643_a_ -0.7777806 | 8.57370611 | -7.8744577 | 4.40E-05 | 0.00059136 | 1.92064621 |
| Pgd | 1436771_x_a-0.7777024 | 10.0878502 | -4.4926846 | 0.0019266 | 0.00962665 | -2.1966365 |
| Lgals8 | 1422662_at -0.7773287 | 9.38163506 | -11.04572 | 3.46E-06 | 0.0001024 | 4.70320833 |
| Runx1 | 1422864_at -0.7768073 | 7.50593585 | -10.606407 | 4.72E-06 | 0.00012571 | 4.36313937 |
| Nudt21 | 1417681_at -0.776322 | 10.4440468 | -8.2583419 | 3.10E-05 | 0.00046879 | 2.30305504 |
| Ormdl2 | 1424235_at -0.7762958 | 8.75007049 | -6.0886851 | 0.00027135 | 0.00222939 | -0.0700373 |
| Naip2 | 1460273_a_ -0.7759845 | 7.67681022 | -5.7991651 | 0.00037739 | 0.0028279 | -0.4296745 |
| Fndc3a | 1426903_at -0.7759632 | 9.12760812 | -9.617021 | 9.94E-06 | 0.00021265 | 3.54849118 |
| Smagp | 1425108_a_ -0.7754078 | 8.39553711 | -5.4771556 | 0.00055169 | 0.00371341 | -0.8429808 |
| Camk1d | 1452050_at -0.7752122 | 8.36095131 | -4.0132769 | 0.00372778 | 0.01601652 | -2.9046171 |
| Vsir | 1448407_at -0.7744102 | 8.99633548 | -5.9186791 | 0.00032892 | 0.00257349 | -0.2798626 |
| Metrnl | 1424356_a_ -0.7743987 | 9.49651636 | -5.5725613 | 0.00049228 | 0.00340437 | -0.7190404 |
| Arhgap9 | 1424249_a_ -0.7739635 | 9.38685015 | -5.2714544 | 0.00070833 | 0.00446569 | -1.1145171 |
| App | 1427442_a_ -0.773896 | 12.1302448 | -7.6536251 | 5.41E-05 | 0.00067943 | 1.69392965 |
| NA | 1455571_x_a-0.7736036 | 11.839652 | -8.1485431 | 3.42E-05 | 0.00049839 | 2.19515716 |
| Rps20 | 1456436_x_a-0.7735774 | 13.0219585 | -11.448607 | 2.62E-06 | 8.39E-05 | 5.00437047 |
| Ccnd2 | 1455956_x_a-0.7731915 | 10.2374487 | -8.1431054 | 3.44E-05 | 0.00049956 | 2.18978323 |
| Tm4sf1 | 1450958_at -0.7726616 | 11.9858182 | -7.6963202 | 5.19E-05 | 0.00066133 | 1.73815701 |
| NA | 1425436_x_a-0.7724446 | 8.63102796 | -6.390715 | 0.00019451 | 0.00174031 | 0.29344934 |
| Plxnb2 | 1416683_at -0.7724117 | 10.8343622 | -7.5361148 | 6.05E-05 | 0.0007345 | 1.57120829 |
| Nupl1 | 1437843_s_a-0.7724065 | 8.63487976 | -10.359101 | 5.65E-06 | 0.00014112 | 4.16605574 |
| Sec61a1 | 1434986_a_ -0.7721083 | 9.19691096 | -9.5460868 | 1.05E-05 | 0.00022115 | 3.48729153 |
| Rbms1 | 1418703_at -0.7720528 | 10.1242133 | -7.9717037 | 4.02E-05 | 0.00055646 | 2.0189009 |
| Eif6 | 1427578_a_ -0.7720098 | 10.0894616 | -13.121637 | 9.08E-07 | 3.89E-05 | 6.15723337 |
| Mical1 | 1416759_at -0.7714907 | 7.78763264 | -5.3500055 | 0.00064341 | 0.00417831 | -1.0101268 |
| Nupr1 | 1419666_x_a-0.7707746 | 8.64206961 | -5.0273965 | 0.00096016 | 0.00563529 | -1.4444077 |
| Map2k1 | 1416351_at -0.7705629 | 10.2930456 | -9.4927697 | 1.10E-05 | 0.00022812 | 3.4410313 |
| E2f8 | 1436186_at -0.7704956 | 7.68110805 | -7.6488599 | 5.43E-05 | 0.00068137 | 1.68898154 |
| Col4a1 | 1452035_at -0.7698484 | 12.0604771 | -7.0067934 | 0.00010196 | 0.00107882 | 0.99969524 |
| Arhgap4 | 1419296_at -0.7695495 | 7.89928644 | -5.8844691 | 0.00034205 | 0.00264203 | -0.3225494 |
| Plk4 | 1419838_s_a-0.7694967 | 7.68711756 | -10.482598 | 5.16E-06 | 0.00013297 | 4.26499578 |
| Mad2l1 | 1422460_at -0.7687483 | 8.42742739 | -7.744776 | 4.96E-05 | 0.00064046 | 1.78812105 |
| Ywhaq | 1437608_x_a-0.7687094 | 8.33160395 | -9.0267181 | 1.60E-05 | 0.00029436 | 3.0268751 |
| Tmpo | 1452036_a_ -0.7686538 | 9.6372697 | -5.4359526 | 0.00057974 | 0.00385759 | -0.8968976 |
| Cip2a | 1449699_s_a-0.768581 | 6.75483157 | -6.2366747 | 0.00023019 | 0.00197209 | 0.10953112 |
| Far1 | 1426371_at -0.7683789 | 8.41939561 | -6.3238456 | 0.00020919 | 0.00183475 | 0.21398115 |
| Hnrnpf | 1450963_at -0.7683698 | 9.77307014 | -6.2068604 | 0.00023789 | 0.00201739 | 0.0735832 |
| Agfg1 | 1452237_at -0.768349 | 8.29414686 | -7.9739876 | 4.01E-05 | 0.00055597 | 2.02119706 |
| Ccr5 | 1422260_x_a-0.7679697 | 7.24533776 | -5.316562 | 0.00067022 | 0.00429828 | -1.0544656 |
| Mcm2 | 1448777_at -0.767819 | 8.04891916 | -5.6610466 | 0.00044339 | 0.0031607 | -0.6052123 |
| Zwint | 1427539_a_ -0.767708 | 7.55084715 | -9.3892886 | 1.19E-05 | 0.00024139 | 3.35060247 |
| Psmc2 | 1435859_x_a-0.7675141 | 11.6140308 | -11.25281 | 3.00E-06 | 9.21E-05 | 4.85925101 |

| NA | 1427404_x_a-0.7659099 | 11.952739 | -3.591999 | 0.00684281 | 0.02544882 | -3.5504345 |
| --- | --- | --- | --- | --- | --- | --- |
| Nfatc3 | 1419976_s_a-0.7656174 | 8.52447813 | -6.5212481 | 0.00016902 | 0.001573 | 0.44695484 |
| Rab5c | 1424684_at -0.7652943 | 9.38688533 | -6.333999 | 0.00020688 | 0.00182226 | 0.22608402 |
| Gng12 | 1455089_at -0.7649985 | 9.15840385 | -6.9833037 | 0.00010442 | 0.00109536 | 0.97359917 |
| Cbl | 1455886_at -0.7649058 | 8.14926403 | -7.4452852 | 6.60E-05 | 0.00078395 | 1.47533905 |
| Gna13 | 1453470_a_ -0.7648081 | 8.25971279 | -8.7180845 | 2.08E-05 | 0.00035342 | 2.74251878 |
| Vsir | 1416619_at -0.7645261 | 9.00138797 | -4.0251763 | 0.00366571 | 0.0158206 | -2.8866801 |
| Eif4e2 | 1421985_a_ -0.7632927 | 9.60186197 | -8.6232617 | 2.25E-05 | 0.00037519 | 2.65346694 |
| Ggct | 1435695_a_ -0.7631327 | 9.44718988 | -12.150757 | 1.65E-06 | 6.07E-05 | 5.50644692 |
| Ywhaq | 1420829_a_ -0.7631324 | 10.0729112 | -8.4345113 | 2.65E-05 | 0.00042254 | 2.47376951 |
| Nhp2 | 1416606_s_a-0.7630017 | 9.52342654 | -8.1933702 | 3.29E-05 | 0.00048681 | 2.23934899 |
| Tmem168 | 1423821_at -0.7629437 | 7.6176624 | -5.8557406 | 0.00035353 | 0.00269996 | -0.3585175 |
| Xbp1 | 1420011_s_a-0.7626061 | 10.4073819 | -8.4181238 | 2.69E-05 | 0.00042686 | 2.45801265 |
| Ctsb | 1417490_at -0.7625651 | 13.227901 | -8.5133674 | 2.48E-05 | 0.0004015 | 2.54924184 |
| Txndc17 | 1423034_at -0.7623938 | 9.21623112 | -7.8574173 | 4.47E-05 | 0.00059724 | 1.90333052 |
| Tor1aip2 | 1435526_at -0.7619841 | 8.61203265 | -13.253 | 8.40E-07 | 3.69E-05 | 6.24171135 |
| Abi1 | 1438506_s_a-0.7615954 | 7.07251688 | -10.366668 | 5.62E-06 | 0.00014064 | 4.17214814 |
| Mcl1 | 1456381_x_a-0.7609614 | 10.3945738 | -10.818192 | 4.06E-06 | 0.00011321 | 4.52864786 |
| Rpl27 | 1448217_a_ -0.7605928 | 10.6951364 | -12.729603 | 1.15E-06 | 4.68E-05 | 5.9001822 |
| Lipa | 1423141_at -0.7604874 | 9.15523666 | -5.9977866 | 0.00030061 | 0.00241023 | -0.1817508 |
| Hdc | 1451796_s_a-0.7601788 | 8.52400406 | -10.717757 | 4.36E-06 | 0.00011856 | 4.45052823 |
| Rad50 | 1422630_at -0.760162 | 8.79361242 | -8.5422609 | 2.42E-05 | 0.00039487 | 2.57675179 |
| Adam10 | 1450105_at -0.75989 | 8.85997909 | -5.238904 | 0.0007373 | 0.00458735 | -1.1580291 |
| Rps9 | 1434624_x_a-0.7594624 | 11.3426055 | -8.1205843 | 3.51E-05 | 0.00050594 | 2.16749551 |
| G6pdx | 1448354_at -0.7592613 | 9.28578357 | -5.1863384 | 0.00078685 | 0.00481882 | -1.2286118 |
| NA | 1456590_x_a-0.7591197 | 11.3419278 | -9.8509681 | 8.29E-06 | 0.0001871 | 3.74758397 |
| Rnps1 | 1437027_x_a-0.7589875 | 10.3086332 | -10.06575 | 7.04E-06 | 0.0001672 | 3.92674788 |
| Sars | 1426257_a_ -0.7589493 | 10.5880223 | -8.1915781 | 3.29E-05 | 0.00048727 | 2.237586 |
| Ddx54 | 1438853_x_a-0.7576857 | 9.16342465 | -10.706298 | 4.39E-06 | 0.00011923 | 4.44157269 |
| Pak1 | 1420979_at -0.7575706 | 7.48621331 | -6.3110372 | 0.00021214 | 0.0018524 | 0.19869471 |
| Tor1aip2 | 1448958_at -0.7570014 | 8.41656775 | -7.8100034 | 4.67E-05 | 0.00061513 | 1.85499455 |
| Atp13a1 | 1417475_at -0.7564857 | 9.54621717 | -8.8855646 | 1.80E-05 | 0.00031939 | 2.89785369 |
| Cdr2 | 1417430_at -0.7560735 | 8.77636797 | -3.4480782 | 0.00846762 | 0.0301003 | -3.7753725 |
| Slc7a5 | 1418326_at -0.7557979 | 8.84962547 | -7.7959426 | 4.73E-05 | 0.00062001 | 1.84061596 |
| Kif11 | 1452314_at -0.7557691 | 7.54830674 | -7.5264983 | 6.11E-05 | 0.00074 | 1.56110028 |
| Btg2 | 1416250_at -0.7556433 | 10.4983247 | -7.9853962 | 3.97E-05 | 0.00055221 | 2.03265901 |
| Taok3 | 1455733_at -0.7553858 | 7.79770129 | -5.3228431 | 0.00066509 | 0.00427628 | -1.0461263 |
| Snhg1 | 1439399_a_ -0.7547112 | 8.51632354 | -9.829961 | 8.43E-06 | 0.00018834 | 3.7298765 |
| Tep1 | 1418196_at -0.7544373 | 9.18974706 | -6.5876147 | 0.00015749 | 0.001497 | 0.52418856 |
| Apod | 1416371_at -0.7543798 | 9.26334911 | -9.7597119 | 8.90E-06 | 0.00019596 | 3.67041994 |
| Pdcd6ip | 1449674_s_a-0.7539189 | 9.2463489 | -5.957974 | 0.00031451 | 0.00248786 | -0.2310239 |
| Pgd | 1437380_x_a-0.7536444 | 9.98317493 | -4.8229254 | 0.00124699 | 0.00687724 | -1.7272854 |
| Stxbp1 | 1420505_a_ -0.7535006 | 9.11093878 | -4.9626647 | 0.00104232 | 0.00599497 | -1.53332 |
| Zup1 | 1436899_at -0.7528134 | 8.14686 | -8.7325671 | 2.05E-05 | 0.00035013 | 2.75604904 |
| Lat2 | 1426169_a_ -0.7523232 | 8.09514803 | -7.2990253 | 7.62E-05 | 0.00086701 | 1.31907626 |
| Cdk9 | 1417269_at -0.7522406 | 9.80350212 | -6.5443187 | 0.00016491 | 0.00154474 | 0.47386479 |
| Clec4d | 1420804_s_a-0.7518775 | 7.91706401 | -12.635903 | 1.22E-06 | 4.86E-05 | 5.83761924 |
| Tgm2 | 1426004_a_ -0.7518222 | 9.92313852 | -4.9212109 | 0.00109893 | 0.00623992 | -1.5905711 |
| Cept1 | 1416471_at -0.7511213 | 10.4745682 | -6.2126226 | 0.00023638 | 0.00201031 | 0.08053983 |
| NA | 1436822_x_a-0.7509216 | 12.0936582 | -9.6456956 | 9.72E-06 | 0.00020951 | 3.5731195 |
| Srd5a3 | 1456691_s_a-0.7508589 | 7.81762034 | -7.4134135 | 6.81E-05 | 0.00080061 | 1.4414873 |
| Psma3 | 1448442_a_ -0.7507814 | 11.6518563 | -9.1279616 | 1.47E-05 | 0.00027833 | 3.11837095 |
| Ikbkb | 1426207_at -0.7506025 | 9.00392921 | -4.2520999 | 0.00267187 | 0.01238755 | -2.5480978 |
| Ggh | 1419595_a_ -0.7504028 | 8.98633064 | -5.888471 | 0.00034049 | 0.00263584 | -0.3175479 |
| Far1 | 1426369_at -0.750051 | 8.44979986 | -5.4082886 | 0.00059945 | 0.00395623 | -0.9332304 |
| Cmas | 1426662_at -0.7490436 | 10.2325382 | -5.6198057 | 0.00046548 | 0.0032719 | -0.658131 |
| Triobp | 1427447_a_ -0.7486204 | 10.1788988 | -7.5147359 | 6.17E-05 | 0.00074681 | 1.54872321 |
| Snx5 | 1417647_at -0.7483496 | 9.7556861 | -6.4432709 | 0.00018377 | 0.00167458 | 0.35551096 |
| Zwilch | 1416757_at -0.7479239 | 6.97114227 | -6.488961 | 0.00017496 | 0.00161902 | 0.40918372 |
| Aurkb | 1424128_x_a-0.7476473 | 7.44144655 | -5.3949139 | 0.00060924 | 0.00400665 | -0.9508344 |
| Rpn1 | 1439257_x_a-0.7475716 | 11.2202415 | -8.5475984 | 2.40E-05 | 0.00039333 | 2.58182532 |
| Pdpn | 1419309_at -0.7470461 | 9.47487744 | -9.0233769 | 1.61E-05 | 0.00029481 | 3.02384084 |
| Prcp | 1452191_at -0.7460148 | 7.95201466 | -5.5928328 | 0.00048058 | 0.00334467 | -0.6928679 |
| Slc25a45 | 1426883_at -0.7457502 | 9.12132537 | -6.4741247 | 0.00017777 | 0.0016352 | 0.39178391 |
| Rpl18 | 1437005_a_ -0.7456956 | 12.7868063 | -8.8247988 | 1.90E-05 | 0.00033176 | 2.84177966 |
| Aars | 1423685_at -0.7454018 | 9.84334283 | -5.8519117 | 0.00035509 | 0.00270732 | -0.3633196 |
| Csnk2a2 | 1453099_at -0.7449315 | 8.82272968 | -9.1888949 | 1.40E-05 | 0.00026856 | 3.173023 |
| Tmem179b | 1449842_at -0.7444867 | 8.80534638 | -6.8492653 | 0.00011978 | 0.0012182 | 0.8234539 |
| Timeless | 1417586_at -0.7444844 | 9.25363981 | -6.0815995 | 0.00027352 | 0.00224452 | -0.0787063 |
| Xbp1 | 1437223_s_a-0.7443591 | 11.4737688 | -9.4481589 | 1.14E-05 | 0.00023301 | 3.40215213 |
| Ptpn1 | 1438670_at -0.7434867 | 9.57060357 | -4.7959601 | 0.00129131 | 0.00706362 | -1.7650331 |
| Sirpa | 1416986_a_ -0.7433155 | 8.33614666 | -5.8661646 | 0.00034932 | 0.0026759 | -0.3454538 |
| Ubxn4 | 1426486_at -0.7431461 | 10.1918058 | -6.6126616 | 0.00015336 | 0.00146852 | 0.55319562 |
| Mtdh | 1434882_at -0.743049 | 8.65037603 | -7.9143313 | 4.24E-05 | 0.00057782 | 1.96104894 |
| Bach1 | 1440831_at -0.7426584 | 9.7497162 | -6.1369179 | 0.00025711 | 0.00213771 | -0.0111997 |
| Pkp3 | 1418831_at -0.7420624 | 8.07651869 | -7.3124924 | 7.52E-05 | 0.00085906 | 1.33356283 |
| Faap20 | 1456310_a_ -0.7414845 | 8.68529418 | -8.2341045 | 3.17E-05 | 0.00047651 | 2.27933726 |
| Nbeal2 | 1455694_at -0.7414548 | 8.25485307 | -5.1089389 | 0.00086656 | 0.00518929 | -1.3332506 |
| Nudt5 | 1448651_at -0.7414481 | 8.49593751 | -8.7708702 | 1.99E-05 | 0.00034276 | 2.79174359 |
| Il13ra1 | 1454783_at -0.7410004 | 9.15527623 | -10.951276 | 3.69E-06 | 0.00010687 | 4.63115257 |
| Tmod3 | 1423089_at -0.7407368 | 8.0699683 | -6.2023289 | 0.00023909 | 0.00202211 | 0.06810942 |
| S100a9 | 1448756_at -0.7406415 | 8.4521103 | -2.2954741 | 0.05023038 | 0.1179842 | -5.6052412 |
| Myo5a | 1419754_at -0.7399893 | 7.05520445 | -8.0188359 | 3.85E-05 | 0.0005402 | 2.06618023 |
| Lxn | 1416503_at -0.7398721 | 8.62705504 | -6.9544141 | 0.00010754 | 0.00111773 | 0.94141601 |
| Tapbpl | 1424961_at -0.7388589 | 7.59567071 | -9.2931342 | 1.29E-05 | 0.00025427 | 3.26580435 |

| Tra2a | 1454725_at | -0.7385929 | 8.83226759 | -9.338627 | 1.24E-05 | 0.00024806 | 3.30601763 |
| --- | --- | --- | --- | --- | --- | --- | --- |
| Nop58 | 1450986_at | -0.7385774 | 8.29462775 | -8.437505 | 2.65E-05 | 0.00042254 | 2.47664524 |
| Ints5 | 1434011_a_ | -0.7385769 | 9.88594788 | -10.562233 | 4.87E-06 | 0.00012843 | 4.3282419 |
| Chac1 | 1451382_at | -0.7383947 | 8.40386729 | -7.0057627 | 0.00010207 | 0.00107882 | 0.99855153 |
| Ifitm2 | 1417460_at | -0.7382327 | 11.7935069 | -9.0346252 | 1.59E-05 | 0.00029331 | 3.03405211 |
| Enpp4 | 1452639_at | -0.7381685 | 8.83456922 | -10.552184 | 4.91E-06 | 0.00012878 | 4.32028448 |
| Stmn1 | 1448113_at | -0.7381189 | 7.33527944 | -7.5763539 | 5.82E-05 | 0.00071543 | 1.61339704 |
| Il27ra | 1449508_at | -0.7377732 | 8.43123771 | -7.180616 | 8.56E-05 | 0.00094767 | 1.19083213 |
| NA | 1419943_s_a-0.7377549 | | 7.78591027 | -8.3848643 | 2.77E-05 | 0.00043588 | 2.42595597 |
| Napsa | 1423590_at -0.7372061 | | 8.13171792 | -5.7367316 | 0.00040578 | 0.00296337 | -0.5087013 |
| Spred1 | 1423160_at -0.7366673 | | 9.29384666 | -10.402021 | 5.48E-06 | 0.0001384 | 4.20056065 |
| Trim27 | 1438376_s_a-0.7365344 | | 9.6216049 | -9.6295233 | 9.85E-06 | 0.00021178 | 3.55923711 |
| Ppp1r15b | 1426798_a_ -0.7364729 | | 9.41669502 | -5.4422751 | 0.00057534 | 0.00383954 | -0.8886088 |
| Pak1 | 1450070_s_a-0.7364472 | | 8.07629193 | -5.6186843 | 0.0004661 | 0.00327421 | -0.6595733 |
| Mov10l1 | 1419340_at -0.7363869 | | 10.163831 | -7.6239394 | 5.56E-05 | 0.00069195 | 1.66306571 |
| Prkch | 1422079_at -0.7358629 | | 8.17286495 | -4.8544033 | 0.00119734 | 0.00666854 | -1.6833508 |
| Dnajc3 | 1449372_at -0.7356427 | | 9.47537407 | -7.1160547 | 9.13E-05 | 0.00099766 | 1.12024307 |
| NA | 1416026_a_ -0.7355686 | | 12.3714474 | -10.569863 | 4.85E-06 | 0.00012788 | 4.33427894 |
| Brip1os | 1433954_at -0.735365 | | 9.21781667 | -9.9509342 | 7.68E-06 | 0.00017705 | 3.831396 |
| Rnf115 | 1423615_at -0.7351857 | | 9.68635317 | -10.805792 | 4.09E-06 | 0.00011393 | 4.51903827 |
| Tax1bp1 | 1420174_s_a-0.7348645 | | 7.90237137 | -10.409704 | 5.45E-06 | 0.00013794 | 4.20672345 |
| Il13ra1 | 1427164_at -0.7345597 | | 8.45273516 | -8.9753048 | 1.67E-05 | 0.00030267 | 2.98007894 |
| Tpm3 | 1449996_a_ -0.7342745 | | 7.87520224 | -8.5275527 | 2.45E-05 | 0.00039766 | 2.56275748 |
| Tmem123 | 1417222_a_ -0.7340775 | | 9.16292076 | -8.8385073 | 1.87E-05 | 0.00032896 | 2.85445788 |
| Lamp2 | 1416343_a_ -0.7340671 | | 9.85932475 | -5.2482932 | 0.00072881 | 0.00455354 | -1.1454628 |
| Ncf1 | 1425609_at -0.7339244 | | 7.48230998 | -6.4927837 | 0.00017424 | 0.00161503 | 0.41366242 |
| Ywhaq | 1420828_s_a-0.7337203 | | 9.66027212 | -6.4407156 | 0.00018428 | 0.00167718 | 0.35250157 |
| Rela | 1419536_a_ -0.7336825 | | 10.6885212 | -6.3177413 | 0.00021059 | 0.00184259 | 0.20669842 |
| Dync1h1 | 1416648_at -0.733535 | | 11.5075858 | -6.6540012 | 0.00014681 | 0.00141767 | 0.60090362 |
| Spcs2 | 1423215_at -0.7322688 | | 10.5785273 | -6.4852149 | 0.00017566 | 0.00162355 | 0.40479294 |
| Ccdc12 | 1419803_s_a-0.7317814 | | 11.255552 | -6.3224511 | 0.00020951 | 0.00183613 | 0.21231784 |
| Xbp1 | 1420886_a_ -0.7317034 | | 11.8297807 | -8.8184571 | 1.91E-05 | 0.00033277 | 2.83590901 |
| Eif3c | 1456374_x_a-0.7316507 | | 10.972223 | -8.1904146 | 3.30E-05 | 0.00048746 | 2.2364412 |
| Ywhaz | 1416102_at -0.7315822 | | 10.6907971 | -8.098015 | 3.58E-05 | 0.00051312 | 2.1451103 |
| Arid5a | 1451340_at -0.7315195 | | 8.71611678 | -10.792969 | 4.13E-06 | 0.00011443 | 4.50909075 |
| Selenos | 1435735_x_a-0.7304568 | | 9.05493248 | -9.1268708 | 1.47E-05 | 0.00027833 | 3.11738974 |
| Cd27 | 1452389_at -0.7295354 | | 7.15205689 | -6.0796278 | 0.00027412 | 0.00224625 | -0.0811199 |
| Tmem45a | 1422587_at -0.7291176 | | 9.0324677 | -9.3262529 | 1.25E-05 | 0.00024924 | 3.29509623 |
| Nsa2 | 1423132_a_ -0.7290231 | | 10.0832729 | -7.4966634 | 6.28E-05 | 0.00075749 | 1.52967718 |
| NA | 1460257_a_ -0.7289242 | | 7.69294035 | -5.0764683 | 0.00090258 | 0.00536256 | -1.3774011 |
| Rarres2 | 1428538_s_a-0.7288412 | | 10.18878 | -6.7597658 | 0.00013142 | 0.00130158 | 0.72201633 |
| Atp7a | 1436921_at -0.7286686 | | 8.59013684 | -7.1904604 | 8.48E-05 | 0.00094165 | 1.20155417 |
| Cdc42se2 | 1435220_s_a-0.7285254 | | 8.9454186 | -6.4489776 | 0.00018264 | 0.00166901 | 0.36222906 |
| Etv3 | 1418637_at -0.7284028 | | 8.86518681 | -7.355866 | 7.20E-05 | 0.00083425 | 1.3800836 |
| Fam189b | 1454893_at -0.7283183 | | 8.15623533 | -5.6886755 | 0.00042924 | 0.00308696 | -0.5698903 |
| Ell2 | 1450744_at -0.7282869 | | 8.87213433 | -8.111727 | 3.54E-05 | 0.00050839 | 2.15871643 |
| Cdkn2d | 1416253_at -0.7275018 | | 9.48852711 | -7.4233606 | 6.74E-05 | 0.00079722 | 1.45206421 |
| Cmip | 1427980_at -0.7271977 | | 9.21143533 | -5.3344502 | 0.00065573 | 0.00423891 | -1.0307302 |
| Fam91a1 | 1433724_at -0.7264053 | | 7.85687154 | -6.9951998 | 0.00010316 | 0.00108471 | 0.98682328 |
| Hprt | 1448736_a_ -0.7262435 | | 10.7879119 | -8.9818383 | 1.66E-05 | 0.00030156 | 2.98603819 |
| Npm1 | 1415839_a_ -0.7257014 | | 11.3520194 | -8.3109621 | 2.96E-05 | 0.00045338 | 2.35435367 |
| Nars | 1428666_at -0.7253647 | | 10.5303045 | -12.355263 | 1.45E-06 | 5.50E-05 | 5.6475522 |
| Fam120a | 1433572_a_ -0.7251668 | | 11.1069461 | -10.367921 | 5.62E-06 | 0.00014064 | 4.1731569 |
| Rbmxl1 | 1416177_at -0.7250546 | | 9.18461792 | -9.9682316 | 7.58E-06 | 0.00017545 | 3.845823 |
| March5 | 1452925_a_ -0.7250446 | | 10.1772411 | -8.5615103 | 2.38E-05 | 0.00038994 | 2.59503695 |
| Mapk9 | 1421877_at -0.7249887 | | 9.35983143 | -6.8440603 | 0.00012042 | 0.00122421 | 0.81758072 |
| Stat2 | 1421911_at -0.724741 | | 8.33080985 | -9.4045989 | 1.18E-05 | 0.00023927 | 3.36403568 |
| Me2 | 1426572_at -0.7241428 | | 8.2298033 | -9.1520997 | 1.44E-05 | 0.00027496 | 3.14005778 |
| Ube2o | 1433765_at -0.7240157 | | 9.0407565 | -3.7985814 | 0.00506444 | 0.02025241 | -3.2312164 |
| Cklf | 1451374_x_a-0.7236473 | | 7.23681028 | -9.0069247 | 1.63E-05 | 0.00029718 | 3.00888591 |
| Hip1 | 1434557_at -0.7236153 | | 9.26060729 | -5.4883835 | 0.00054431 | 0.00367352 | -0.8283293 |
| Chic2 | 1419659_s_a-0.7232682 | | 9.46379712 | -7.9841385 | 3.97E-05 | 0.00055251 | 2.03139609 |
| Sgpp1 | 1420822_s_a-0.7232591 | | 9.38821639 | -8.1824405 | 3.32E-05 | 0.00049033 | 2.22859211 |
| Grina | 1417423_at -0.7232101 | | 10.5102831 | -5.5036917 | 0.00053441 | 0.0036305 | -0.8083815 |
| Tbc1d14 | 1420196_s_a-0.7227069 | | 9.50811268 | -9.2144118 | 1.37E-05 | 0.00026503 | 3.19581788 |
| Panx1 | 1416379_at -0.7216215 | | 8.16956364 | -8.8431018 | 1.87E-05 | 0.00032794 | 2.8587034 |
| NA | 1420376_a_ -0.7212381 | | 10.839333 | -10.325651 | 5.79E-06 | 0.00014385 | 4.13907566 |
| Mapre2 | 1451989_a_ -0.7212347 | | 10.097878 | -5.1148923 | 0.00086013 | 0.00515757 | -1.325172 |
| Dynll1 | 1456125_a_ -0.7209861 | | 11.4085314 | -9.9715482 | 7.56E-06 | 0.00017545 | 3.84858665 |
| Prim1 | 1449061_a_ -0.7207878 | | 7.27557928 | -9.218535 | 1.37E-05 | 0.00026494 | 3.19949615 |
| Nfkb1 | 1427705_a_ -0.7207779 | | 10.6564657 | -7.751295 | 4.93E-05 | 0.00063865 | 1.79482434 |
| Il4ra | 1423996_a_ -0.7205674 | | 8.56982457 | -6.2040658 | 0.00023863 | 0.0020211 | 0.07020778 |
| Bax | 1416837_at -0.7204965 | | 9.36622159 | -7.3541869 | 7.21E-05 | 0.00083477 | 1.37828648 |
| Slirp | 1428554_a_ -0.7203689 | | 10.9474117 | -8.1586297 | 3.39E-05 | 0.00049572 | 2.2051178 |
| Lrp10 | 1416836_at -0.7197503 | | 10.913888 | -6.90908 | 0.00011264 | 0.00116187 | 0.89071707 |
| Wasf2 | 1454673_at -0.7197184 | | 11.0418527 | -7.3404022 | 7.31E-05 | 0.00084225 | 1.36352161 |
| Ddx21 | 1448271_a_ -0.7187961 | | 8.33176807 | -9.8252964 | 8.46E-06 | 0.00018883 | 3.72594011 |
| N4bp1 | 1418154_at -0.7187022 | | 9.52804649 | -9.0611507 | 1.56E-05 | 0.00028924 | 3.05808974 |
| Mtmr6 | 1425486_s_a-0.7185811 | | 9.98285894 | -9.7025436 | 9.30E-06 | 0.00020252 | 3.62175769 |
| Plcg2 | 1426926_at -0.718363 | | 9.56777494 | -9.2083708 | 1.38E-05 | 0.00026567 | 3.19042617 |
| Il3ra | 1419712_at -0.7178838 | | 8.22644069 | -5.3991766 | 0.0006061 | 0.003992 | -0.9452211 |
| Mcl1 | 1448503_at -0.7167357 | | 11.6030092 | -10.399141 | 5.49E-06 | 0.0001384 | 4.19824912 |
| Melk | 1416558_at -0.7166377 | | 6.93294356 | -8.0211931 | 3.84E-05 | 0.00053971 | 2.06853906 |
| NA | 1425471_x_a-0.7166178 | | 7.5846836 | -3.9921047 | 0.00384102 | 0.0163667 | -2.9365757 |
| Ssrp1 | 1426790_at -0.7165723 | | 9.36890101 | -6.9679012 | 0.00010607 | 0.00110959 | 0.9564528 |

| Clec5a | 1421366_at | -0.7162324 | 7.347762 | -6.3664168 | 0.00019971 | 0.00177076 | 0.26463861 |
| --- | --- | --- | --- | --- | --- | --- | --- |
| Plscr3 | 1449020_at | -0.7159627 | 9.21169669 | -6.697822 | 0.00014021 | 0.00137242 | 0.6512478 |
| Psma4 | 1460339_at | -0.7150521 | 11.1812082 | -7.9072153 | 4.27E-05 | 0.00058012 | 1.95385031 |
| Eno1 | 1419023_x_a-0.714643 | | 11.986251 | -3.5176789 | 0.00763604 | 0.02781088 | -3.6663502 |
| Sh3d19 | 1449084_s_a-0.7144059 | | 8.92548801 | -6.3912071 | 0.00019441 | 0.00174007 | 0.294032 |
| Ezr | 1450850_at -0.7139554 | | 9.34340292 | -6.8499035 | 0.0001197 | 0.00121794 | 0.82417387 |
| NA | 1456012_x_a-0.7138897 | | 12.0979867 | -8.9939328 | 1.65E-05 | 0.0002992 | 2.99706017 |
| Incenp | 1423092_at -0.7136224 | | 8.54924371 | -6.4299882 | 0.00018642 | 0.00169078 | 0.33985867 |
| Csf3r | 1418806_at -0.7133624 | | 8.50983797 | -6.2364793 | 0.00023024 | 0.00197209 | 0.1092959 |
| Pde4b | 1422473_at -0.7132525 | | 8.76272321 | -5.8246039 | 0.00036645 | 0.00277082 | -0.3976258 |
| G3bp2 | 1423758_at -0.7125202 | | 9.86273424 | -11.646666 | 2.30E-06 | 7.56E-05 | 5.14884459 |
| Etnk1 | 1433514_at -0.7117408 | | 9.7092078 | -9.4474385 | 1.14E-05 | 0.00023301 | 3.40152299 |
| Dynll1 | 1417339_a_ -0.7116671 | | 11.3156828 | -9.5521093 | 1.05E-05 | 0.0002205 | 3.49250288 |
| NA | 1448232_x_a-0.7115671 | | 9.31574114 | -4.72199 | 0.00142191 | 0.00761284 | -1.8691097 |
| Ogfrl1 | 1424412_at -0.7115436 | | 9.45742629 | -8.133933 | 3.47E-05 | 0.00050209 | 2.1807118 |
| NA | 1434396_a_ -0.7114008 | | 12.9079326 | -7.4143506 | 6.80E-05 | 0.00080029 | 1.4424842 |
| Psmd10 | 1436559_a_ -0.7107186 | | 8.4579853 | -7.0970987 | 9.31E-05 | 0.00101162 | 1.09942717 |
| Aars | 1451083_s_a-0.7105961 | | 10.301012 | -6.6730094 | 0.00014391 | 0.00139898 | 0.62277012 |
| NA | 1434503_s_a-0.7102322 | | 11.4502866 | -6.8658351 | 0.00011775 | 0.00120222 | 0.84212937 |
| NA | 1434872_x_a-0.7099853 | | 12.1916455 | -12.1463 | 1.66E-06 | 6.08E-05 | 5.50334678 |
| Mertk | 1422869_at -0.7098039 | | 8.42013035 | -4.4103092 | 0.0021528 | 0.01050424 | -2.316085 |
| Acp2 | 1424654_at -0.7097859 | | 8.58387421 | -5.9882964 | 0.00030386 | 0.00242856 | -0.1934771 |
| Tbca | 1437907_a_ -0.7096826 | | 10.2655197 | -7.0353115 | 9.91E-05 | 0.00105521 | 1.03129171 |
| Ywhab | 1420878_a_ -0.7091902 | | 9.39418695 | -5.0026216 | 0.00099074 | 0.00576703 | -1.4783671 |
| Eml3 | 1460366_at -0.7085384 | | 7.89949847 | -6.8792953 | 0.00011613 | 0.00119072 | 0.85727617 |
| Depdc1a | 1424292_at -0.7081659 | | 6.53743709 | -7.6664689 | 5.34E-05 | 0.00067533 | 1.70725446 |
| NA | 1424991_s_a-0.7080861 | | 7.68572703 | -7.4231328 | 6.75E-05 | 0.00079722 | 1.45182212 |
| Eif3e | 1460432_a_ -0.7076615 | | 11.8501928 | -7.3140907 | 7.50E-05 | 0.00085815 | 1.33528078 |
| Tbcd | 1455801_x_a-0.707636 | | 9.89387614 | -10.539639 | 4.95E-06 | 0.00012965 | 4.31034106 |
| Acp2 | 1436788_at -0.7075755 | | 8.77143536 | -4.0771485 | 0.00340733 | 0.01495113 | -2.8085471 |
| Tmem128 | 1448317_at -0.7072802 | | 8.90357021 | -10.480724 | 5.17E-06 | 0.00013297 | 4.26350224 |
| Pitpna | 1423283_at -0.7068663 | | 10.147653 | -6.0976686 | 0.00026864 | 0.00221488 | -0.0590556 |
| Avl9 | 1434659_at -0.7065486 | | 7.96273063 | -7.5911261 | 5.74E-05 | 0.00070855 | 1.62884159 |
| Ran | 1434578_x_a-0.7062827 | | 11.1985245 | -7.2635217 | 7.89E-05 | 0.00089274 | 1.28078841 |
| Slc30a7 | 1450697_at -0.7060691 | | 8.68463695 | -9.5933916 | 1.01E-05 | 0.00021564 | 3.52814821 |
| Gnptab | 1435335_a_ -0.7055504 | | 8.57076982 | -7.7908791 | 4.75E-05 | 0.00062152 | 1.83543305 |
| Tmbim1 | 1417162_at -0.7048995 | | 10.9256999 | -10.377048 | 5.58E-06 | 0.00014004 | 4.18049965 |
| Capn2 | 1416257_at -0.7043176 | | 10.1867121 | -8.4283531 | 2.67E-05 | 0.00042453 | 2.46785121 |
| Ggta1 | 1418483_a_ -0.7039984 | | 9.05641157 | -5.0586182 | 0.00092308 | 0.00547001 | -1.4017355 |
| Ube2i | 1422712_a_ -0.7039317 | | 10.5029673 | -8.0953643 | 3.59E-05 | 0.00051338 | 2.14247799 |
| Rarres2 | 1437902_s_a-0.7036198 | | 10.2215112 | -6.0936013 | 0.00026986 | 0.00222337 | -0.0640263 |
| Fmr1 | 1423369_at -0.7032449 | | 8.91908005 | -7.3643306 | 7.14E-05 | 0.00082823 | 1.38913818 |
| Eea1 | 1438045_at -0.7032327 | | 9.42121122 | -7.6600867 | 5.37E-05 | 0.00067791 | 1.70063545 |
| Zfp346 | 1417088_at -0.7025715 | | 9.31307109 | -8.7426406 | 2.03E-05 | 0.00034803 | 2.76544914 |
| Eprs | 1452158_at -0.7025359 | | 8.86463309 | -9.175428 | 1.42E-05 | 0.00027107 | 3.16097089 |
| Ankib1 | 1429193_at -0.70251 | | 8.73073994 | -7.7477477 | 4.95E-05 | 0.00064014 | 1.79117731 |
| Ifnar1 | 1449026_at -0.702212 | | 8.61558704 | -5.7611161 | 0.00039442 | 0.00291512 | -0.4777731 |
| Csf1 | 1460220_a_ -0.7021582 | | 10.3278371 | -10.475076 | 5.19E-06 | 0.00013313 | 4.25899941 |
| Tcrb-J | 1427667_s_a-0.7008604 | | 6.70408273 | -8.8701747 | 1.83E-05 | 0.00032237 | 2.88368256 |
| Uvrag | 1454706_at -0.7008596 | | 9.62067626 | -5.971034 | 0.00030987 | 0.00246272 | -0.2148373 |
| Nub1 | 1420535_a_ -0.7003306 | | 8.63759157 | -8.1790459 | 3.33E-05 | 0.00049151 | 2.22524884 |
| Lmna | 1421654_a_ -0.7001171 | | 9.8055089 | -5.432089 | 0.00058245 | 0.00387219 | -0.9019654 |
| Agfg1 | 1426923_at -0.6993613 | | 9.51385342 | -9.3457788 | 1.23E-05 | 0.00024711 | 3.31232415 |
| Kdm3a | 1426810_at -0.6989928 | | 8.99062963 | -9.6659488 | 9.57E-06 | 0.0002074 | 3.59047634 |
| Arhgef1 | 1421164_a_ -0.6986636 | | 9.87368002 | -3.2886188 | 0.01075492 | 0.036368 | -4.0266737 |
| Actb | AFFX-b-Actin -0.6983227 | | 13.0678848 | -5.7938176 | 0.00037973 | 0.00283797 | -0.4364225 |
| Ifngr1 | 1448167_at -0.6980672 | | 10.0275165 | -6.4756608 | 0.00017748 | 0.0016352 | 0.39358673 |
| Man2a1 | 1448647_at -0.6974855 | | 10.334425 | -6.0455381 | 0.00028483 | 0.00231643 | -0.1229289 |
| Cln3 | 1417551_at -0.6971097 | | 8.20307418 | -6.5991773 | 0.00015557 | 0.00148436 | 0.53758886 |
| Hvcn1 | 1424032_at -0.6969304 | | 8.05915652 | -5.0992373 | 0.00087715 | 0.00523892 | -1.3464264 |
| Myo5a | 1431320_a_ -0.6961253 | | 7.82576505 | -6.4221529 | 0.000188 | 0.0017002 | 0.33061508 |
| Tor1aip2 | 1418115_s_a-0.6960331 | | 10.725291 | -12.094688 | 1.71E-06 | 6.22E-05 | 5.46736849 |
| Akr1a1 | 1430123_a_ -0.6957306 | | 12.4571828 | -8.3499823 | 2.86E-05 | 0.00044246 | 2.39222385 |
| Slc30a4 | 1418843_at -0.6952716 | | 9.19951059 | -8.9816411 | 1.66E-05 | 0.00030156 | 2.98585841 |
| Ran | 1438977_x_a-0.6951138 | | 10.3430676 | -8.6596755 | 2.18E-05 | 0.00036605 | 2.68776024 |
| Rad51ap1 | 1417938_at -0.6950179 | | 7.18500894 | -9.362469 | 1.22E-05 | 0.00024489 | 3.32702564 |
| Snrpg | 1448358_s_a-0.6948228 | | 10.6580102 | -8.4814015 | 2.55E-05 | 0.00041074 | 2.51871702 |
| Nap1l1 | 1452778_x_a-0.6944748 | | 9.99530222 | -6.6628786 | 0.00014545 | 0.00140793 | 0.61112138 |
| Vamp3 | 1437708_x_a-0.694466 | | 10.6024501 | -9.5880124 | 1.02E-05 | 0.00021575 | 3.52351105 |
| Bcl10 | 1418971_x_a-0.6942627 | | 9.23558868 | -7.9513908 | 4.10E-05 | 0.00056419 | 1.99845602 |
| Cemip2 | 1451458_at -0.6941368 | | 8.49168669 | -7.7122019 | 5.12E-05 | 0.0006537 | 1.75455991 |
| Hspa5 | 1416064_a_ -0.6940296 | | 12.2906948 | -8.3130639 | 2.95E-05 | 0.00045315 | 2.35639717 |
| Tmbim6 | 1451586_at -0.6939071 | | 11.7813171 | -4.4613968 | 0.00200934 | 0.00995633 | -2.241895 |
| Arhgap9 | 1449619_s_a-0.6931305 | | 8.39132891 | -4.5765726 | 0.00172243 | 0.00882611 | -2.0759617 |
| Tent2 | 1419183_at -0.6912969 | | 9.22271878 | -8.0825454 | 3.63E-05 | 0.00051754 | 2.12973804 |
| Celf1 | 1427413_a_ -0.6911623 | | 9.66376236 | -5.975161 | 0.00030843 | 0.00245464 | -0.209727 |
| Mapre1 | 1422764_at -0.6908264 | | 10.2674673 | -7.1267324 | 9.04E-05 | 0.00098895 | 1.13195033 |
| Lcp2 | 1418642_at -0.6902599 | | 7.22408367 | -8.485323 | 2.54E-05 | 0.00040962 | 2.52246682 |
| S1pr4 | 1451024_at -0.6901414 | | 8.44359873 | -7.7811863 | 4.80E-05 | 0.00062481 | 1.82550428 |
| Cebpd | 1423233_at -0.6898983 | | 10.1441236 | -3.1119908 | 0.01406509 | 0.04480408 | -4.3070559 |
| Lmnb1 | 1423521_at -0.6895689 | | 9.39979179 | -8.1413256 | 3.44E-05 | 0.00050004 | 2.18802361 |
| Cmtr1 | 1423681_at -0.6890605 | | 9.85913288 | -8.6734671 | 2.16E-05 | 0.00036367 | 2.70071746 |
| Cpd | 1455009_at -0.6887432 | | 8.93496222 | -8.2818513 | 3.04E-05 | 0.00046153 | 2.32600668 |
| Loxl1 | 1451978_at -0.6886996 | | 10.5414042 | -5.7746346 | 0.00038827 | 0.00288149 | -0.4606616 |
| Tsr1 | 1433502_s_a-0.688691 | | 8.40453924 | -8.3446719 | 2.87E-05 | 0.00044424 | 2.38707839 |

| Zmynd8 | 1426614_at -0.6886678 | 9.74859939 | -7.4069856 | 6.85E-05 | 0.00080437 | 1.43464664 |
| --- | --- | --- | --- | --- | --- | --- |
| Uba2 | 1437278_a_ -0.6884236 | 9.93132644 | -8.8717432 | 1.82E-05 | 0.00032236 | 2.88512777 |
| Tex10 | 1439464_s_a-0.6883672 | 8.85249509 | -10.841398 | 3.99E-06 | 0.00011197 | 4.54660384 |
| Snrpa1 | 1417351_a_ -0.6883005 | 8.46443089 | -7.0127458 | 0.00010135 | 0.00107404 | 1.00629793 |
| Tspan4 | 1448276_at -0.6882877 | 10.2774075 | -10.884672 | 3.87E-06 | 0.0001096 | 4.57999493 |
| Ttc39b | 1452009_at -0.6881021 | 8.14832061 | -10.357557 | 5.66E-06 | 0.00014112 | 4.16481199 |
| Gmfb | 1448570_at -0.6880488 | 10.0619336 | -7.7410142 | 4.98E-05 | 0.00064205 | 1.78425089 |
| Pld3 | 1416013_at -0.6878302 | 9.9530741 | -4.604023 | 0.00166081 | 0.00858756 | -2.0366869 |
| Ddx39 | 1438168_x_a-0.6875986 | 9.26502322 | -6.0117991 | 0.00029589 | 0.00238326 | -0.1644586 |
| Leprotl1 | 1451018_at -0.6866697 | 9.51334513 | -6.3413568 | 0.00020523 | 0.00181053 | 0.23484633 |
| Arf4 | 1423053_at -0.686448 | 10.2046381 | -7.9829117 | 3.98E-05 | 0.00055279 | 2.03016399 |
| Rpl27 | 1453096_x_a-0.6863516 | 10.8728356 | -11.808318 | 2.06E-06 | 7.01E-05 | 5.26507562 |
| Trps1 | 1449530_at -0.6863417 | 8.10454051 | -5.646227 | 0.00045119 | 0.00320424 | -0.6242014 |
| Tubb3 | 1415978_at -0.6862902 | 7.86853173 | -6.0920768 | 0.00027032 | 0.00222475 | -0.0658898 |
| Spcs1 | 1448258_a_ -0.686164 | 11.1497701 | -7.0840702 | 9.43E-05 | 0.00102088 | 1.08509647 |
| Chfr | 1433550_at -0.6856854 | 9.33560021 | -8.7514759 | 2.02E-05 | 0.00034689 | 2.77368637 |
| Pgs1 | 1455229_x_a-0.6854991 | 9.03732549 | -7.0694999 | 9.57E-05 | 0.00103006 | 1.06904694 |
| Adcy7 | 1420970_at -0.685414 | 8.10310631 | -4.7026872 | 0.00145831 | 0.00775367 | -1.896396 |
| Syk | 1418261_at -0.6850851 | 9.47669547 | -4.1525558 | 0.00306662 | 0.01381136 | -2.6957978 |
| Bhlhe40 | 1418025_at -0.6844663 | 10.0379538 | -8.3748626 | 2.80E-05 | 0.00043853 | 2.41629572 |
| Scn10a | 1450266_at -0.6844395 | 7.40289648 | -8.1680491 | 3.36E-05 | 0.00049399 | 2.2144107 |
| Eif3i | 1448264_a_ -0.6842593 | 10.8489658 | -6.7846657 | 0.00012806 | 0.00127611 | 0.75033362 |
| Arf4 | 1423052_at -0.684231 | 11.2623056 | -7.0329987 | 9.93E-05 | 0.00105718 | 1.02873273 |
| Npm1 | 1432416_a_ -0.6840556 | 10.7046392 | -7.2442808 | 8.04E-05 | 0.00090287 | 1.25997993 |
| Tut7 | 1441986_at -0.6830622 | 7.84887731 | -8.9707659 | 1.68E-05 | 0.00030357 | 2.97593677 |
| Rilpl2 | 1423134_at -0.6829268 | 9.46513559 | -9.7688153 | 8.83E-06 | 0.00019515 | 3.67814591 |
| Smc1a | 1417830_at -0.6817173 | 9.6780913 | -6.6603803 | 0.00014583 | 0.00140993 | 0.60824684 |
| Cnot6 | 1426683_at -0.6811608 | 8.31580858 | -6.8200538 | 0.00012345 | 0.00124589 | 0.79045091 |
| B3gnt2 | 1450026_a_ -0.6811566 | 9.0835253 | -6.216894 | 0.00023527 | 0.00200177 | 0.08569386 |
| Adap2 | 1425639_at -0.6811078 | 8.31470428 | -5.4660982 | 0.00055907 | 0.00375306 | -0.857427 |
| Pkig | 1434820_s_a-0.6809404 | 11.5594987 | -7.146829 | 8.86E-05 | 0.00097539 | 1.1539496 |
| Cnn3 | 1438354_x_a-0.680872 | 8.26625725 | -6.2716301 | 0.0002215 | 0.00191607 | 0.15153223 |
| NA | 1424854_at -0.6804476 | 7.46088231 | -10.313891 | 5.85E-06 | 0.00014479 | 4.12957115 |
| Rgs16 | 1455265_a_ -0.6801309 | 7.45356145 | -10.418137 | 5.41E-06 | 0.00013727 | 4.21348422 |
| Calcrl | 1425814_a_ -0.6792201 | 8.53731943 | -6.4896502 | 0.00017483 | 0.00161881 | 0.40999135 |
| Ecm1 | 1448613_at -0.6791673 | 9.80541651 | -4.2914415 | 0.00253126 | 0.01190103 | -2.4900875 |
| Kif20a | 1449207_a_ -0.6789152 | 7.12717791 | -9.7615448 | 8.88E-06 | 0.00019588 | 3.67197606 |
| Klra7 | 1426171_x_a-0.6785764 | 7.14147295 | -5.4775567 | 0.00055143 | 0.00371341 | -0.842457 |
| Tmem123 | 1448623_at -0.6784362 | 10.4235471 | -5.8853463 | 0.00034171 | 0.00264169 | -0.3214529 |
| Kif22 | 1451128_s_a-0.6782331 | 7.08248823 | -7.0826261 | 9.44E-05 | 0.00102172 | 1.08350685 |
| Akr1a1 | 1451035_a_ -0.6777048 | 12.0067177 | -6.1396401 | 0.00025633 | 0.00213436 | -0.0078879 |
| Cp | 1417496_at -0.6777004 | 8.92661942 | -4.1821198 | 0.0029432 | 0.01336696 | -2.6517954 |
| Fkbp15 | 1455339_at -0.6776555 | 8.9476628 | -7.2870584 | 7.71E-05 | 0.00087632 | 1.30618653 |
| P2rx4 | 1452527_a_ -0.6773522 | 8.31617453 | -4.6835995 | 0.00149529 | 0.00790866 | -1.92343 |
| Il21r | 1450456_at -0.6771811 | 7.89816181 | -5.8034301 | 0.00037553 | 0.00281676 | -0.4242951 |
| Necap2 | 1418962_at -0.6770182 | 9.18695991 | -6.3535381 | 0.00020253 | 0.00179015 | 0.24933785 |
| Saraf | 1424039_at -0.6769691 | 13.0398105 | -10.671869 | 4.50E-06 | 0.0001212 | 4.41461469 |
| NA | 1455065_x_a-0.6767483 | 9.24798923 | -4.8558487 | 0.00119511 | 0.00665777 | -1.6813368 |
| Mif | 1416335_at -0.6761713 | 11.8863349 | -7.7400239 | 4.98E-05 | 0.00064223 | 1.78323177 |
| Sin3b | 1455039_a_ -0.6761004 | 10.6919166 | -7.6934016 | 5.21E-05 | 0.00066204 | 1.7351397 |
| Wdr1 | 1423054_at -0.6760728 | 9.9426114 | -5.7050257 | 0.00042109 | 0.00304383 | -0.5490365 |
| Ncl | 1415771_at -0.6760443 | 11.5466965 | -10.182654 | 6.44E-06 | 0.00015507 | 4.02284948 |
| Znrf2 | 1434016_at -0.6759357 | 9.36181154 | -6.6688024 | 0.00014454 | 0.00140278 | 0.61793432 |
| Fbxw11 | 1437468_x_a-0.6756142 | 11.1085628 | -11.430125 | 2.66E-06 | 8.47E-05 | 4.99077088 |
| Snrpb2 | 1426613_a_ -0.675397 | 9.03266813 | -7.8574627 | 4.47E-05 | 0.00059724 | 1.90337669 |
| Stat6 | 1426353_at -0.6753097 | 9.87368834 | -4.4172514 | 0.00213267 | 0.0104222 | -2.3059824 |
| Ptpn12 | 1450479_x_a-0.6751204 | 9.09417288 | -8.5294364 | 2.44E-05 | 0.00039758 | 2.56455079 |
| Atp6v0e | 1416328_a_ -0.6747454 | 10.5238644 | -7.5860911 | 5.77E-05 | 0.00071112 | 1.62358 |
| NA | 1425787_a_ -0.6746157 | 8.01305412 | -10.373122 | 5.60E-06 | 0.00014029 | 4.17734191 |
| Ran | 1433569_x_a-0.6742211 | 12.2132236 | -9.1508476 | 1.45E-05 | 0.00027496 | 3.13893403 |
| Pdcd1lg2 | 1450290_at -0.6741841 | 8.26806641 | -4.9799911 | 0.0010196 | 0.0058957 | -1.5094631 |
| Gnai2 | 1435652_a_ -0.6738549 | 12.2803768 | -7.8242903 | 4.61E-05 | 0.00060947 | 1.86958348 |
| Emb | 1415856_at -0.6737937 | 8.05958012 | -6.4362608 | 0.00018516 | 0.00168256 | 0.3472531 |
| NA | 1428475_at -0.6735472 | 8.60954762 | -6.5802028 | 0.00015873 | 0.00150378 | 0.51558995 |
| Ankrd10 | 1416065_a_ -0.6734803 | 8.22048612 | -8.5092153 | 2.49E-05 | 0.00040233 | 2.54528228 |
| Acsl4 | 1418911_s_a-0.6733872 | 8.54259677 | -6.6731593 | 0.00014388 | 0.00139898 | 0.62294235 |
| Vcan | 1421694_a_ -0.6733412 | 8.19883325 | -9.4493331 | 1.14E-05 | 0.00023301 | 3.40317749 |
| Srsf7 | 1424883_s_a-0.6729408 | 10.0115992 | -7.3351816 | 7.35E-05 | 0.00084532 | 1.35792427 |
| Caprin1 | 1416462_at -0.6727903 | 11.2118469 | -7.6541207 | 5.41E-05 | 0.00067943 | 1.69444407 |
| Jak3 | 1425750_a_ -0.6725641 | 8.95180276 | -6.0095239 | 0.00029665 | 0.00238855 | -0.1672645 |
| St6galnac4 | 1418074_at -0.6725381 | 8.17294642 | -7.5226795 | 6.13E-05 | 0.00074153 | 1.55708359 |
| Tppp3 | 1416713_at -0.6722839 | 9.55710068 | -3.0954343 | 0.01442585 | 0.0456963 | -4.3334228 |
| Eef1a1 | 1424635_at -0.6721432 | 13.9315546 | -7.4214216 | 6.76E-05 | 0.00079752 | 1.45000332 |
| Ppib | 1450911_at -0.6718164 | 11.938131 | -8.1676835 | 3.36E-05 | 0.00049399 | 2.21405015 |
| Cotl1 | 1416001_a_ -0.6709427 | 9.70875519 | -8.6717415 | 2.16E-05 | 0.00036389 | 2.69909719 |
| Dlgap4 | 1426465_at -0.6707759 | 9.46706398 | -6.5745346 | 0.00015969 | 0.00151097 | 0.5090096 |
| Atp6v1c1 | 1419545_a_ -0.6704689 | 9.2887328 | -6.4944083 | 0.00017394 | 0.00161354 | 0.41556529 |
| Flot2 | 1438164_x_a-0.6703123 | 11.7114213 | -7.1500308 | 8.83E-05 | 0.00097293 | 1.15745027 |
| Zfp36 | 1452519_a_ -0.6700917 | 10.0644066 | -5.1436855 | 0.00082974 | 0.00502073 | -1.2861708 |
| Eprs | 1426713_s_a-0.6700387 | 9.28315805 | -6.8035085 | 0.00012558 | 0.0012577 | 0.77171314 |
| Kpna2 | 1415860_at -0.6700173 | 10.3588493 | -9.3724205 | 1.21E-05 | 0.00024403 | 3.33578068 |
| Stxbp3 | 1435058_x_a-0.6697378 | 6.57714352 | -9.5366664 | 1.06E-05 | 0.00022237 | 3.47913429 |
| Tle3 | 1419655_at -0.6695471 | 8.53121562 | -5.744872 | 0.00040195 | 0.00294772 | -0.4983674 |
| Plod3 | 1415901_at -0.6693787 | 10.5536258 | -5.619494 | 0.00046565 | 0.0032721 | -0.6585319 |
| Ppp2r1b | 1428265_at -0.6691226 | 8.70277739 | -7.3882846 | 6.98E-05 | 0.00081409 | 1.41471918 |

| Gfi1 | 1417679_at | -0.6685818 | 6.82536418 | -7.3861504 | 6.99E-05 | 0.00081507 | 1.41244261 |
| --- | --- | --- | --- | --- | --- | --- | --- |
| Chmp1b | 1418817_at | -0.6684433 | 9.42991676 | -7.4677395 | 6.46E-05 | 0.00077318 | 1.4991221 |
| NA | 1456635_at | -0.6682506 | 8.67097965 | -5.5541545 | 0.00050317 | 0.00346177 | -0.7428544 |
| Ranbp1 | 1422547_at | -0.6681784 | 10.3003286 | -11.200252 | 3.11E-06 | 9.50E-05 | 4.81990049 |
| Arhgdia | 1451168_a_ | -0.6674877 | 12.0875201 | -5.0544105 | 0.00092798 | 0.0054919 | -1.4074783 |
| NA | 1433439_at | -0.6674324 | 9.66161829 | -7.2314952 | 8.14E-05 | 0.00091222 | 1.24612975 |
| Cd93 | 1419589_at | -0.6673256 | 10.6266298 | -4.4521237 | 0.00203459 | 0.01005331 | -2.2553349 |
| Pgam1 | 1426554_a_ | -0.6673161 | 10.8356177 | -4.9604018 | 0.00104532 | 0.00600313 | -1.536439 |
| Hnrnpu | 1450849_at | -0.6672575 | 10.2465047 | -6.8050732 | 0.00012538 | 0.00125655 | 0.77348666 |
| Ric8a | 1455809_x_a-0.6666425 | | 7.27544682 | -8.3385388 | 2.89E-05 | 0.00044635 | 2.38113256 |
| Psip1 | 1417166_at -0.6658699 | | 8.23282139 | -5.157973 | 0.0008151 | 0.00495036 | -1.2668616 |
| Fgr | 1419526_at -0.6658677 | | 8.5965187 | -5.9488075 | 0.00031781 | 0.00250557 | -0.2423983 |
| Cflar | 1425687_at -0.6656963 | | 7.18891078 | -10.05359 | 7.10E-06 | 0.00016821 | 3.91669446 |
| Grk5 | 1449514_at -0.6654143 | | 9.01739856 | -9.3613898 | 1.22E-05 | 0.00024489 | 3.32607573 |
| Vrk2 | 1451929_a_ -0.6653517 | | 7.3486054 | -6.6690298 | 0.00014451 | 0.00140278 | 0.61819569 |
| Hnrnpm | 1426698_a_ -0.6652031 | | 9.74058533 | -6.4441358 | 0.0001836 | 0.001674 | 0.35652949 |
| Sdf2l1 | 1418206_at -0.6651147 | | 9.32895341 | -6.5350494 | 0.00016654 | 0.00155701 | 0.4630608 |
| Il13ra1 | 1451775_s_a-0.6649383 | | 9.09336961 | -9.3877891 | 1.19E-05 | 0.00024147 | 3.3492858 |
| Rpl18 | 1436699_x_a-0.6648055 | | 11.6100103 | -10.425874 | 5.38E-06 | 0.00013693 | 4.21968168 |
| Atp2a3 | 1421129_a_ -0.6647086 | | 9.42167997 | -5.0285404 | 0.00095877 | 0.00563028 | -1.4428418 |
| Pbk | 1448627_s_a-0.664684 | | 7.46024181 | -5.5390718 | 0.00051229 | 0.00350772 | -0.7624027 |
| Plvap | 1418090_at -0.664438 | | 9.48474794 | -5.6977258 | 0.00042471 | 0.00306215 | -0.5583426 |
| Prmt2 | 1437234_x_a-0.6640862 | | 8.95078371 | -10.697491 | 4.42E-06 | 0.0001197 | 4.43468411 |
| Synpo | 1434089_at -0.6634636 | | 8.09839172 | -6.0929488 | 0.00027006 | 0.00222419 | -0.0648238 |
| Ankrd1 | 1420992_at -0.663417 | | 13.7702439 | -9.1968977 | 1.39E-05 | 0.00026749 | 3.18017789 |
| Rtcb | 1420129_s_a-0.6633438 | | 10.3254479 | -9.1452277 | 1.45E-05 | 0.00027577 | 3.13388861 |
| Pla2g15 | 1423704_at -0.662945 | | 8.84380678 | -3.7066932 | 0.00578567 | 0.02247818 | -3.3726382 |
| Endod1 | 1426541_a_ -0.6619488 | | 6.90269867 | -7.2354571 | 8.11E-05 | 0.00090943 | 1.25042348 |
| Ccnf | 1422513_at -0.6619432 | | 7.75859568 | -7.5721423 | 5.84E-05 | 0.00071784 | 1.60898942 |
| Mllt3 | 1431890_a_ -0.6618993 | | 8.06005037 | -9.0681279 | 1.55E-05 | 0.00028797 | 3.06440258 |
| Srd5a3 | 1439241_x_a-0.6618405 | | 7.77235256 | -9.8010366 | 8.62E-06 | 0.00019146 | 3.70544104 |
| NA | AFFX-18SRN -0.6617849 | | 9.17642024 | -1.7898326 | 0.11055573 | 0.20900762 | -6.3684928 |
| Resf1 | 1427334_s_a-0.661674 | | 8.72559121 | -6.1454232 | 0.00025468 | 0.00212533 | -0.0008558 |
| Cenpe | 1439040_at -0.6610683 | | 6.34905057 | -9.2102808 | 1.38E-05 | 0.00026548 | 3.19213117 |
| Naip5 | 1421525_a_ -0.6609137 | | 7.16582224 | -6.0667682 | 0.00027811 | 0.00227481 | -0.0968735 |
| Pip4k2a | 1419280_at -0.6604331 | | 7.44102625 | -4.4655329 | 0.00199818 | 0.00991864 | -2.2359043 |
| Nt5c3 | 1451050_at -0.6597745 | | 9.99896179 | -5.3724073 | 0.00062611 | 0.00409408 | -0.9805145 |
| Pgd | 1423706_a_ -0.6597478 | | 9.14270144 | -4.1392026 | 0.00312418 | 0.01401218 | -2.7157099 |
| Nip7 | 1448480_at -0.6595334 | | 8.57993204 | -8.6957069 | 2.12E-05 | 0.00035783 | 2.7215758 |
| Akirin1 | 1416911_a_ -0.6592643 | | 8.31379688 | -9.8302402 | 8.42E-06 | 0.00018834 | 3.73011214 |
| Ripor2 | 1460555_at -0.6589303 | | 8.14655464 | -6.6863972 | 0.0001419 | 0.00138559 | 0.63814468 |
| Rap2b | 1448885_at -0.6580483 | | 8.35860092 | -7.4947483 | 6.29E-05 | 0.00075809 | 1.52765687 |
| Il2rb | 1417546_at -0.6578954 | | 8.04693059 | -5.7643109 | 0.00039296 | 0.0029062 | -0.4737269 |
| Hltf | 1450889_at -0.6577487 | | 8.77173526 | -5.9187304 | 0.0003289 | 0.00257349 | -0.2797988 |
| Sec11c | 1460698_a_ -0.657427 | | 9.45593735 | -8.0191546 | 3.85E-05 | 0.0005402 | 2.06649917 |
| Adss | 1460726_at -0.6573546 | | 9.01420912 | -5.286672 | 0.00069522 | 0.00441213 | -1.094226 |
| NA | 1434280_at -0.6572001 | | 7.54292577 | -10.88323 | 3.87E-06 | 0.0001096 | 4.57888401 |
| Ap1s2 | 1452657_at -0.6571023 | | 9.84706557 | -6.8668888 | 0.00011762 | 0.00120167 | 0.84331593 |
| Rbpj | 1418114_at -0.656638 | | 7.90165591 | -6.8710243 | 0.00011713 | 0.00119764 | 0.84797137 |
| Dr1 | 1416018_at -0.6566001 | | 8.9019237 | -8.2256598 | 3.19E-05 | 0.00047885 | 2.27106032 |
| Ccz1 | 1433554_at -0.6565399 | | 10.0462885 | -7.6715068 | 5.32E-05 | 0.00067399 | 1.71247637 |
| Gpr137b-ps | 1439256_x_a-0.6560251 | | 8.02160334 | -5.3674531 | 0.00062989 | 0.00410934 | -0.9870573 |
| Ckap4 | 1426754_x_a-0.655921 | | 8.92138886 | -5.7540443 | 0.00039768 | 0.00292966 | -0.4867343 |
| Traf5 | 1448861_at -0.6559172 | | 8.26078528 | -6.5445696 | 0.00016486 | 0.00154474 | 0.47415713 |
| NA | 1439462_x_a-0.6557327 | | 9.38844313 | -9.577607 | 1.03E-05 | 0.00021691 | 3.51453468 |
| Snx1 | 1416260_a_ -0.655451 | | 9.0718541 | -7.4584527 | 6.52E-05 | 0.0007775 | 1.48929244 |
| Tmem165 | 1415741_at -0.6551465 | | 9.92072186 | -7.6910145 | 5.22E-05 | 0.00066316 | 1.73267129 |
| Tmem167 | 1450904_at -0.6547243 | | 9.0317204 | -7.5818883 | 5.79E-05 | 0.0007132 | 1.61918607 |
| Pbdc1 | 1417921_at -0.6544019 | | 8.38933767 | -8.8192728 | 1.91E-05 | 0.00033277 | 2.8366643 |
| Zfp281 | 1452045_at -0.6543281 | | 9.1681892 | -8.6529519 | 2.20E-05 | 0.00036789 | 2.6814372 |
| NA | 1423740_a_ -0.6541057 | | 8.40266416 | -7.3981891 | 6.91E-05 | 0.00080864 | 1.42527804 |
| Fam220a | 1451975_at -0.6540305 | | 10.1989179 | -5.2376593 | 0.00073843 | 0.00458842 | -1.159696 |
| Selenos | 1448704_s_a-0.6540106 | | 10.5495843 | -10.887648 | 3.86E-06 | 0.0001096 | 4.58228715 |
| Arap1 | 1428064_at -0.6538386 | | 9.49165856 | -4.112672 | 0.00324201 | 0.0144012 | -2.7553407 |
| Vav3 | 1417122_at -0.6538037 | | 7.40834743 | -4.5294367 | 0.0018341 | 0.00927907 | -2.1436472 |
| Rars | 1416312_at -0.6535295 | | 10.1244924 | -6.7880505 | 0.00012761 | 0.00127275 | 0.75417727 |
| Cd4 | 1419696_at -0.6534944 | | 8.69275035 | -7.6373621 | 5.49E-05 | 0.00068695 | 1.67703266 |
| Trem2 | 1421792_s_a-0.653189 | | 7.75890821 | -4.2294315 | 0.00275669 | 0.01270035 | -2.581617 |
| Tgif1 | 1422286_a_ -0.653016 | | 8.06916156 | -7.7016659 | 5.17E-05 | 0.00065911 | 1.74368109 |
| Psen1 | 1421853_at -0.6526573 | | 10.2805447 | -7.317574 | 7.48E-05 | 0.00085608 | 1.33902395 |
| Pold3 | 1426838_at -0.6526261 | | 8.81521994 | -8.1653143 | 3.37E-05 | 0.00049436 | 2.21171355 |
| Rnf138 | 1454064_a_ -0.6522867 | | 8.0666383 | -6.5964705 | 0.00015601 | 0.00148737 | 0.53445331 |
| Il17rc | 1419671_a_ -0.6518066 | | 9.35571812 | -7.6309519 | 5.53E-05 | 0.00068887 | 1.67036489 |
| Tmem219 | 1424497_at -0.651045 | | 8.49425942 | -9.0955781 | 1.51E-05 | 0.00028329 | 3.08919932 |
| Eif4ebp2 | 1436158_at -0.6507508 | | 8.93030892 | -5.4997602 | 0.00053694 | 0.00363892 | -0.8135015 |
| Rtp4 | 1449143_at -0.6507493 | | 7.34521124 | -6.1431232 | 0.00025534 | 0.00212974 | -0.003652 |
| Set | 1426854_a_ -0.6502096 | | 11.3925264 | -8.371657 | 2.80E-05 | 0.00043886 | 2.41319756 |
| Tor1aip2 | 1418116_at -0.6500019 | | 10.3108956 | -10.215621 | 6.29E-06 | 0.00015258 | 4.04977239 |
| Psmd4 | 1418874_a_ -0.6497213 | | 11.4244145 | -9.0678371 | 1.55E-05 | 0.00028797 | 3.06413955 |
| NA | 1426266_s_a-0.6494574 | | 8.40662948 | -5.7347145 | 0.00040674 | 0.00296748 | -0.5112632 |
| Ms4a8a | 1418797_at -0.649298 | | 7.71194185 | -7.4181406 | 6.78E-05 | 0.00079942 | 1.44651511 |
| Psmc2 | 1426611_at -0.6491478 | | 11.7132081 | -8.2759221 | 3.05E-05 | 0.00046351 | 2.32022307 |
| Tcerg1 | 1434434_s_a-0.6489482 | | 9.78459472 | -8.2837624 | 3.03E-05 | 0.00046105 | 2.32787006 |
| Pgs1 | 1454046_x_a-0.6488825 | | 9.24377881 | -4.9157115 | 0.00110669 | 0.00627142 | -1.5981845 |
| Ttc1 | 1448537_at -0.6488205 | | 10.0792654 | -7.0613365 | 9.65E-05 | 0.00103562 | 1.06004404 |

| Gadd45b | 1449773_s_a-0.6484781 | 8.47288921 | -5.3703958 | 0.00062764 | 0.0040982 | -0.9831706 |
| --- | --- | --- | --- | --- | --- | --- |
| Mis18a | 1428483_a_ -0.6483042 | 7.25135167 | -10.835802 | 4.01E-06 | 0.00011222 | 4.54227706 |
| Cenpk | 1418264_at -0.6481497 | 7.13835338 | -6.8978642 | 0.00011394 | 0.00117147 | 0.87813674 |
| Rtf2 | 1460362_at -0.6479558 | 9.01924598 | -7.6323583 | 5.52E-05 | 0.0006887 | 1.67182819 |
| NA | 1418188_a_ -0.6476427 | 11.5032611 | -2.3820597 | 0.04383209 | 0.10677533 | -5.4694973 |
| NA | 1417366_s_a-0.6474082 | 10.479221 | -6.7986062 | 0.00012622 | 0.00126222 | 0.76615492 |
| Celf2 | 1450069_a_ -0.6472158 | 10.3060479 | -9.2930533 | 1.29E-05 | 0.00025427 | 3.26573263 |
| Spn | 1422133_at -0.647207 | 8.0155684 | -6.2533111 | 0.00022601 | 0.00194246 | 0.12954041 |
| Cct4 | 1438560_x_a-0.6468203 | 10.4577832 | -8.1629964 | 3.38E-05 | 0.00049436 | 2.20942688 |
| NA | 1422301_at -0.646718 | 8.62608594 | -6.3190176 | 0.0002103 | 0.00184161 | 0.20822145 |
| Rlim | 1417250_at -0.6465579 | 8.7399729 | -6.8978074 | 0.00011395 | 0.00117147 | 0.87807301 |
| Trim25 | 1426415_a_ -0.646524 | 8.02287518 | -7.8474509 | 4.51E-05 | 0.00060068 | 1.89318942 |
| Snrpb2 | 1452422_a_ -0.6465133 | 9.36082888 | -6.2984582 | 0.00021508 | 0.00187409 | 0.18366164 |
| Nap1l1 | 1420479_a_ -0.6464388 | 8.9040746 | -5.2597437 | 0.00071861 | 0.00451168 | -1.1301543 |
| Gtpbp4 | 1423142_a_ -0.6463197 | 8.37463253 | -6.9431595 | 0.00010878 | 0.00112805 | 0.928852 |
| Lipa | 1423140_at -0.6462243 | 8.64288116 | -3.0973302 | 0.01438405 | 0.04558299 | -4.3304028 |
| Ebi3 | 1449222_at -0.6458856 | 7.93666661 | -5.0477154 | 0.00093585 | 0.00552049 | -1.4166213 |
| Riok3 | 1422650_a_ -0.6455664 | 10.1759639 | -6.6150693 | 0.00015297 | 0.001467 | 0.55598003 |
| Erp44 | 1423247_at -0.6455514 | 9.93500317 | -6.4231195 | 0.00018781 | 0.00169979 | 0.33175593 |
| Egr1 | 1417065_at -0.6454624 | 9.96644398 | -3.188709 | 0.0125124 | 0.04096183 | -4.1850522 |
| Fam32a | 1423217_a_ -0.6453946 | 9.25586131 | -8.1164513 | 3.52E-05 | 0.00050751 | 2.16339999 |
| Ube2v1 | 1415755_a_ -0.6445379 | 11.0670896 | -4.9006347 | 0.00112827 | 0.00636193 | -1.619079 |
| Tnfaip6 | 1418424_at -0.6444834 | 7.34072409 | -7.8415896 | 4.53E-05 | 0.00060325 | 1.88722053 |
| Cenpt | 1426846_at -0.6434064 | 8.49698377 | -8.4042419 | 2.73E-05 | 0.0004306 | 2.4446454 |
| Tmod3 | 1438556_a_ -0.643322 | 7.99996629 | -5.9816743 | 0.00030615 | 0.00244085 | -0.2016665 |
| NA | 1437133_x_a-0.643179 | 12.1599185 | -9.5112084 | 1.08E-05 | 0.00022588 | 3.45705487 |
| Dera | 1424047_at -0.6431129 | 8.27221723 | -7.4753824 | 6.41E-05 | 0.00076952 | 1.50720481 |
| Vps54 | 1418479_at -0.6430446 | 9.49774666 | -7.918656 | 4.22E-05 | 0.00057656 | 1.96542135 |
| Ssrp1 | 1426789_s_a-0.6429812 | 9.54329102 | -7.2219043 | 8.22E-05 | 0.000919 | 1.23572823 |
| Tspan14 | 1423924_s_a-0.6421534 | 8.67254919 | -4.5503248 | 0.00178368 | 0.00907232 | -2.1136143 |
| Etf1 | 1451208_at -0.6419344 | 9.33321121 | -6.8225059 | 0.00012314 | 0.00124424 | 0.79322522 |
| Slu7 | 1425488_at -0.6419085 | 8.38055656 | -6.4249253 | 0.00018744 | 0.00169783 | 0.33388667 |
| P2ry14 | 1424733_at -0.6410361 | 7.9372095 | -4.55704 | 0.00176779 | 0.00900765 | -2.1039721 |
| Hnrnpab | 1455855_x_a-0.6409628 | 11.9765919 | -10.501807 | 5.09E-06 | 0.0001322 | 4.2802907 |
| Faap20 | 1438154_x_a-0.6408963 | 7.91559795 | -8.0980859 | 3.58E-05 | 0.00051312 | 2.14518074 |
| Uba2 | 1448283_a_ -0.6408488 | 9.47377857 | -8.9431415 | 1.72E-05 | 0.0003082 | 2.95068891 |
| Fcho1 | 1436077_a_ -0.6408302 | 8.3636017 | -6.9612758 | 0.00010679 | 0.00111402 | 0.94906873 |
| Eif2s2 | 1417712_at -0.6407936 | 11.831752 | -7.7611039 | 4.89E-05 | 0.00063371 | 1.80490226 |
| Tmem243 | 1436337_at -0.6402431 | 8.64283659 | -9.7127409 | 9.23E-06 | 0.00020149 | 3.63045606 |
| Scfd1 | 1428335_a_ -0.6400742 | 9.1165863 | -7.3954061 | 6.93E-05 | 0.00080972 | 1.42231225 |
| Me2 | 1426573_at -0.6398666 | 6.77555222 | -6.8982735 | 0.00011389 | 0.00117147 | 0.87859605 |
| Mindy3 | 1418963_at -0.6398337 | 8.8500765 | -8.7719146 | 1.98E-05 | 0.00034273 | 2.79271503 |
| Stx3 | 1425536_at -0.6396735 | 8.80209585 | -5.3787585 | 0.0006213 | 0.00406496 | -0.9721318 |
| Agtrap | 1422965_at -0.6395735 | 8.96961484 | -8.0012181 | 3.91E-05 | 0.00054594 | 2.04853344 |
| Rps6ka3 | 1455206_at -0.6394917 | 9.8316883 | -7.4441079 | 6.61E-05 | 0.00078417 | 1.47409065 |
| Eftud2 | 1438835_a_ -0.6394656 | 10.0788765 | -6.6763992 | 0.00014339 | 0.0013958 | 0.62666504 |
| Tspan33 | 1455618_x_a-0.6390471 | 7.36637133 | -5.8306234 | 0.00036391 | 0.00275696 | -0.390055 |
| Entpd1 | 1450939_at -0.6390339 | 7.65786771 | -4.2705906 | 0.00260476 | 0.01217093 | -2.5208069 |
| Pgs1 | 1454045_a_ -0.6385431 | 9.07903109 | -5.2119953 | 0.00076223 | 0.00470739 | -1.1941121 |
| Eif4a1 | 1427058_at -0.6385302 | 12.0427654 | -9.8987885 | 7.99E-06 | 0.00018186 | 3.78776964 |
| Washc1 | 1424597_at -0.6384888 | 9.00471298 | -6.7312417 | 0.00013539 | 0.001331 | 0.68948604 |
| Gusb | 1430332_a_ -0.6382004 | 7.76666094 | -4.3171924 | 0.00244356 | 0.01159199 | -2.4522297 |
| Lgals1 | 1419573_a_ -0.6380833 | 12.8775761 | -10.939085 | 3.72E-06 | 0.00010725 | 4.62180995 |
| NA | 1452731_x_a-0.6375346 | 7.56318065 | -6.0896142 | 0.00027107 | 0.00222928 | -0.068901 |
| Pnrc1 | 1433668_at -0.6374218 | 11.2776052 | -4.1092852 | 0.0032574 | 0.01445538 | -2.7604062 |
| Prpf40a | 1450035_a_ -0.636818 | 8.11157903 | -4.3828407 | 0.00223448 | 0.01080799 | -2.3561233 |
| Btf3 | 1423839_a_ -0.6367694 | 10.8022339 | -6.7570406 | 0.00013179 | 0.00130471 | 0.71891265 |
| Tradd | 1452622_a_ -0.6358115 | 7.97328327 | -7.2775666 | 7.78E-05 | 0.00088286 | 1.29595157 |
| Spdl1 | 1424971_at -0.6355672 | 7.20666382 | -8.1541288 | 3.40E-05 | 0.00049652 | 2.20067433 |
| Bgn | 1437889_x_a-0.6353088 | 11.7292448 | -6.7837439 | 0.00012818 | 0.00127621 | 0.74928667 |
| Sptlc1 | 1436726_s_a-0.6346832 | 7.89795916 | -7.3528387 | 7.22E-05 | 0.00083531 | 1.3768434 |
| Akr1a1 | 1430124_x_a-0.633915 | 12.0349905 | -5.9256051 | 0.00032633 | 0.00255763 | -0.2712395 |
| Uba7 | 1437317_at -0.6337804 | 9.47364587 | -8.5796965 | 2.34E-05 | 0.00038561 | 2.61228101 |
| Mcm7 | 1416031_s_a-0.6337338 | 8.35909562 | -5.574211 | 0.00049131 | 0.00339978 | -0.7169084 |
| Psmd14 | 1421751_a_ -0.6337271 | 11.0648907 | -8.6645668 | 2.17E-05 | 0.00036525 | 2.69235758 |
| Rps20 | 1456373_x_a-0.6336898 | 12.8986016 | -5.9746801 | 0.00030859 | 0.00245512 | -0.2103223 |
| Gmfg | 1419193_a_ -0.6335795 | 7.72177714 | -6.379919 | 0.0001968 | 0.00175299 | 0.2806576 |
| Rexo4 | 1434113_a_ -0.6331177 | 8.06478662 | -7.7320404 | 5.02E-05 | 0.00064618 | 1.77501269 |
| Poldip3 | 1437837_x_a-0.6329681 | 10.41258 | -6.6525932 | 0.00014703 | 0.00141902 | 0.59928214 |
| Shroom3 | 1422629_s_a-0.632955 | 9.34361471 | -4.8871592 | 0.00114794 | 0.00645522 | -1.6377816 |
| Cers2 | 1423955_a_ -0.6325509 | 9.78819243 | -6.8314464 | 0.000122 | 0.00123693 | 0.80333436 |
| Klhl5 | 1426530_a_ -0.631359 | 8.96944381 | -5.0215459 | 0.00096729 | 0.00566393 | -1.4524193 |
| Pnpla7 | 1451361_a_ -0.6312956 | 8.73976712 | -7.7409263 | 4.98E-05 | 0.00064205 | 1.78416042 |
| Srsf1 | 1428099_a_ -0.6312594 | 10.3395795 | -6.7666952 | 0.00013048 | 0.00129501 | 0.72990424 |
| Rpn1 | 1456438_x_a-0.6311293 | 11.0997216 | -7.532087 | 6.07E-05 | 0.00073643 | 1.56697588 |
| Nfat5 | 1454627_a_ -0.6306078 | 13.0336795 | -8.5609083 | 2.38E-05 | 0.00038994 | 2.59446562 |
| Mgat2 | 1426350_at -0.6304497 | 8.15613038 | -9.4563797 | 1.13E-05 | 0.00023294 | 3.40932859 |
| Slamf1 | 1425570_at -0.6303526 | 8.1754189 | -8.7739433 | 1.98E-05 | 0.0003424 | 2.79460175 |
| Psmd4 | 1425859_a_ -0.6301244 | 10.1159245 | -8.2748727 | 3.06E-05 | 0.00046351 | 2.31919914 |
| Ap3s1 | 1422593_at -0.6300548 | 10.0104086 | -5.434957 | 0.00058044 | 0.00385996 | -0.8982033 |
| Hnrnpd | 1425142_a_ -0.6300403 | 10.1075872 | -9.332345 | 1.25E-05 | 0.00024827 | 3.30047468 |
| Pa2g4 | 1435372_a_ -0.6291452 | 10.424843 | -10.360243 | 5.65E-06 | 0.00014112 | 4.1669758 |
| 1110038F14 | 1416885_at -0.6291142 | 8.82415524 | -8.3302007 | 2.91E-05 | 0.00044874 | 2.3730433 |
| Ptpra | 1425340_a_ -0.6291063 | 9.28411156 | -5.2676947 | 0.00071161 | 0.00447643 | -1.1195354 |
| Rasal3 | 1456870_at -0.6288025 | 8.09003502 | -5.6695628 | 0.00043897 | 0.00313611 | -0.5943137 |

| Cct4 1433447_x_a-0.6287399 | 11.2084821 | -10.307588 | 5.87E-06 | 0.00014531 | 4.12447361 |
| --- | --- | --- | --- | --- | --- |
| Adam17 1421857_at -0.6286927 | 8.29211559 | -5.3495228 | 0.00064379 | 0.00417838 | -1.0107657 |
| Snhg1 1433674_a_ -0.6281888 | 8.6823362 | -6.5777317 | 0.00015915 | 0.00150695 | 0.51272172 |
| Cttnbp2nl 1418985_at -0.6279347 | 7.34809013 | -8.9470629 | 1.71E-05 | 0.00030811 | 2.95427695 |
| Ptger4 1421073_a_ -0.6274194 | 7.08239433 | -5.0396554 | 0.00094541 | 0.00556744 | -1.4276365 |
| Akr1a1 1435011_x_a-0.6272455 | 11.9798587 | -6.3631647 | 0.00020042 | 0.00177496 | 0.26077694 |
| NA 1448879_at -0.6269246 | 11.0714382 | -8.7150802 | 2.08E-05 | 0.0003539 | 2.73970964 |
| Gatad2a 1451197_s_a-0.6268159 | 9.21711154 | -5.0218139 | 0.00096696 | 0.00566348 | -1.4520523 |
| Bach1 1449311_at -0.6266479 | 8.11704175 | -6.0022559 | 0.0002991 | 0.00240231 | -0.1762327 |
| Tpst2 1416086_at -0.6261728 | 9.47263408 | -6.1629378 | 0.00024977 | 0.00209355 | 0.0204153 |
| Neat1 1428083_at -0.6261128 | 12.8413684 | -7.1982235 | 8.41E-05 | 0.00093624 | 1.21000166 |
| Fam76b 1426985_s_a-0.6259401 | 8.5242171 | -7.5854492 | 5.77E-05 | 0.00071117 | 1.62290904 |
| Pdgfb 1450414_at -0.6256127 | 9.83866993 | -7.485845 | 6.35E-05 | 0.00076381 | 1.51825932 |
| Aida 1460345_at -0.6255913 | 8.41116921 | -8.4670028 | 2.58E-05 | 0.00041505 | 2.50493662 |
| Eif2ak2 1422006_at -0.6254668 | 7.97443185 | -8.3633659 | 2.83E-05 | 0.00044026 | 2.40517979 |
| St8sia4 1419186_a_ -0.6254128 | 9.03041737 | -3.0605213 | 0.01521862 | 0.04770791 | -4.389061 |
| Derl2 1448438_at -0.6249981 | 9.83448245 | -4.8574626 | 0.00119263 | 0.00664855 | -1.6790883 |
| Dck 1428838_a_ -0.6247982 | 7.54926457 | -6.3746298 | 0.00019793 | 0.00175893 | 0.27438525 |
| Sft2d1 1460656_a_ -0.62477 | 9.4233605 | -8.3114587 | 2.96E-05 | 0.00045338 | 2.35483652 |
| Mast3 1435666_at -0.6246141 | 8.69388841 | -5.6296262 | 0.00046011 | 0.00324792 | -0.6455085 |
| Wdr81 1460234_at -0.6244663 | 9.38776255 | -7.1014173 | 9.27E-05 | 0.00100902 | 1.1041731 |
| Pisd-ps3 1434975_x_a-0.6244552 | 9.25881415 | -8.0457995 | 3.76E-05 | 0.00053179 | 2.09312867 |
| Rnf4 1423654_a_ -0.624455 | 8.7746196 | -7.1302322 | 9.00E-05 | 0.00098621 | 1.13578484 |
| Nek6 1423596_at -0.6242228 | 8.43374583 | -5.0882447 | 0.00088933 | 0.00529909 | -1.3613714 |
| Tlr7 1422010_at -0.6241674 | 7.54701299 | -5.9137447 | 0.00033078 | 0.0025801 | -0.2860101 |
| Selenow 1460561_x_a-0.6240691 | 12.7985841 | -5.8154025 | 0.00037036 | 0.00279318 | -0.409208 |
| Meox1 1417595_at -0.6237409 | 9.31768681 | -5.7224489 | 0.0004126 | 0.0029987 | -0.5268543 |
| Rpl3 1449323_a_ -0.6236961 | 12.7743455 | -10.086523 | 6.93E-06 | 0.00016516 | 3.94389589 |
| Med11 1417739_at -0.6236057 | 7.97198886 | -9.380691 | 1.20E-05 | 0.00024263 | 3.34305079 |
| St3gal1 1418946_at -0.6233985 | 8.11005549 | -8.1223253 | 3.50E-05 | 0.00050546 | 2.16922018 |
| Eif3c 1456083_x_a-0.6233677 | 10.7414019 | -7.3766848 | 7.06E-05 | 0.0008204 | 1.40233936 |
| Anxa3 1460330_at -0.623171 | 9.93872713 | -7.6551983 | 5.40E-05 | 0.00067917 | 1.69556265 |
| Hmmr 1427541_x_a-0.6230914 | 7.54238121 | -8.5237185 | 2.45E-05 | 0.0003987 | 2.5591061 |
| Fam91a1 1454705_at -0.622795 | 8.73035887 | -6.8532704 | 0.00011929 | 0.00121474 | 0.82797097 |
| Kras 1451979_at -0.6224968 | 9.90334215 | -7.9039131 | 4.28E-05 | 0.0005812 | 1.95050805 |
| Suv39h1 1427382_a_ -0.6224368 | 7.93203251 | -9.7983868 | 8.63E-06 | 0.00019148 | 3.70319932 |
| Rpl22l1 1417126_a_ -0.6215489 | 10.9566689 | -7.7609327 | 4.89E-05 | 0.00063371 | 1.80472643 |
| Alg5 1424160_at -0.6212252 | 8.8873448 | -6.2550456 | 0.00022558 | 0.00194023 | 0.13162454 |
| Washc4 1427319_at -0.6211187 | 8.42504711 | -7.9505308 | 4.10E-05 | 0.00056429 | 1.99758951 |
| Ttc13 1437709_x_a-0.6210648 | 8.75553646 | -6.6289494 | 0.00015074 | 0.00144808 | 0.57201757 |
| Xpo1 1418443_at -0.6209773 | 9.68694805 | -5.231719 | 0.00074387 | 0.00461537 | -1.1676538 |
| Il6ra 1452416_at -0.6208133 | 8.18933328 | -4.1320254 | 0.0031556 | 0.01411681 | -2.7264221 |
| D16Ertd472e 1451466_at -0.6207191 | 8.39124684 | -6.6372156 | 0.00014943 | 0.00143733 | 0.58155735 |
| Tyk2 1417306_at -0.6207101 | 8.69908189 | -7.7760669 | 4.82E-05 | 0.0006273 | 1.82025636 |
| Nars 1452866_at -0.6205709 | 10.9197741 | -10.825271 | 4.04E-06 | 0.00011292 | 4.53412878 |
| Aurkb 1451246_s_a-0.6204031 | 6.808268 | -5.1095187 | 0.00086593 | 0.00518689 | -1.3324636 |
| Snx6 1451602_at -0.6201205 | 7.96568987 | -8.618074 | 2.26E-05 | 0.00037632 | 2.6485716 |
| Ran 1439270_x_a-0.6200692 | 12.1600751 | -7.333525 | 7.36E-05 | 0.00084578 | 1.35614749 |
| Slc16a6 1417884_at -0.619917 | 8.14249669 | -4.6245078 | 0.00161638 | 0.00840416 | -2.007447 |
| Armc7 1424446_at -0.6190982 | 8.36339211 | -6.4613196 | 0.00018023 | 0.00165231 | 0.37674418 |
| Elmo2 1456098_a_ -0.6190721 | 9.03319731 | -5.5003861 | 0.00053653 | 0.00363728 | -0.8126862 |
| Psmb4 1438984_x_a-0.6190129 | 11.0547903 | -6.3168572 | 0.00021079 | 0.00184259 | 0.2056432 |
| Hnrnpf 1423382_a_ -0.6186687 | 10.171788 | -4.6313757 | 0.00160178 | 0.00833587 | -1.9976569 |
| Rbm14 1436979_x_a-0.6183618 | 8.8149959 | -7.3495095 | 7.25E-05 | 0.00083689 | 1.37327888 |
| Poldip3 1455788_x_a-0.6182452 | 9.43887087 | -8.040743 | 3.77E-05 | 0.00053281 | 2.08808053 |
| Mcl1 1416880_at -0.6181257 | 11.031587 | -5.0726174 | 0.00090696 | 0.00538434 | -1.382647 |
| Ube2h 1418631_at -0.6179796 | 9.93502749 | -3.690803 | 0.00592113 | 0.02286816 | -3.3971884 |
| Hnrnpk 1448176_a_ -0.6179465 | 11.5318267 | -6.9622479 | 0.00010668 | 0.00111394 | 0.95015249 |
| NA 1416336_s_a-0.6177778 | 8.83249489 | -8.9559107 | 1.70E-05 | 0.00030672 | 2.96236776 |
| NA 1422418_s_a-0.6177468 | 9.74304866 | -5.3291569 | 0.00065998 | 0.00425427 | -1.0377491 |
| NA 1423807_a_ -0.6177075 | 12.0097406 | -7.001935 | 0.00010246 | 0.00107991 | 0.99430302 |
| Gramd1a 1416708_a_ -0.6176105 | 8.97212825 | -5.5325523 | 0.00051629 | 0.00352852 | -0.7708622 |
| Col14a1 1427168_a_ -0.6174568 | 9.2081297 | -3.2238888 | 0.01186148 | 0.03929006 | -4.1292136 |
| Uap1l1 1436890_at -0.6172759 | 8.84796588 | -8.2594431 | 3.10E-05 | 0.00046864 | 2.3041313 |
| Nqo1 1423627_at -0.6166617 | 9.67304635 | -7.8053898 | 4.69E-05 | 0.00061671 | 1.85027893 |
| Ero1l 1419029_at -0.6166279 | 8.53602271 | -7.8038835 | 4.70E-05 | 0.00061686 | 1.84873881 |
| Sdad1 1452454_at -0.6166044 | 7.44567087 | -7.099199 | 9.29E-05 | 0.00101078 | 1.10173556 |
| Atm 1421205_at -0.6164417 | 8.05548462 | -8.0672475 | 3.68E-05 | 0.00052271 | 2.11451316 |
| NA 1425078_x_a-0.6163 | 6.83501241 | -5.284651 | 0.00069694 | 0.00441946 | -1.096919 |
| Atp11b 1451388_a_ -0.6159821 | 9.81499349 | -5.5060086 | 0.00053293 | 0.00362479 | -0.8053652 |
| Gmfb 1448571_a_ -0.6152579 | 7.62437677 | -10.418271 | 5.41E-06 | 0.00013727 | 4.21359134 |
| Cxcl13 1417851_at -0.6147011 | 8.27952928 | -1.1254971 | 0.2924093 | 0.41791175 | -7.2227239 |
| Bub1b 1416961_at -0.6145984 | 8.06207111 | -7.7809755 | 4.80E-05 | 0.00062481 | 1.82528834 |
| Adamts4 1455965_at -0.6144981 | 7.26063845 | -5.9870128 | 0.00030431 | 0.00243124 | -0.195064 |
| Bcl2l11 1435448_at -0.6140925 | 8.98973076 | -6.0954863 | 0.00026929 | 0.00221949 | -0.0617223 |
| Cd300a 1435903_at -0.6135735 | 7.8642325 | -5.2174333 | 0.00075712 | 0.0046822 | -1.186812 |
| Esd 1417825_at -0.613566 | 12.1556708 | -9.5881445 | 1.02E-05 | 0.00021575 | 3.52362498 |
| Prim2 1418036_at -0.613381 | 7.71931747 | -6.0598671 | 0.00028028 | 0.00228564 | -0.1053366 |
| Ccnd2 1434745_at -0.6126497 | 11.3441875 | -7.1181298 | 9.11E-05 | 0.00099655 | 1.12251923 |
| Specc1 1428794_at -0.6126471 | 8.09950705 | -4.9316244 | 0.0010844 | 0.00618062 | -1.5761663 |
| Creb3 1424740_at -0.6124697 | 8.5160684 | -7.0365419 | 9.89E-05 | 0.00105489 | 1.0326528 |
| NA 1454688_x_a-0.6123993 | 8.97211135 | -8.4529277 | 2.61E-05 | 0.00041871 | 2.49144724 |
| Ttc1 1416994_at -0.6120885 | 9.03271939 | -4.8759556 | 0.00116458 | 0.00653261 | -1.6533507 |
| Cct2 1433535_x_a-0.6120437 | 11.2828915 | -9.93453 | 7.78E-06 | 0.00017856 | 3.8176936 |
| Sh3bp1 1460222_at -0.6115111 | 8.19977053 | -3.8425562 | 0.0047538 | 0.01928705 | -3.1638745 |

| Pcolce | 1437165_a_ -0.6114106 | 11.1490441 | -8.373357 | 2.80E-05 | 0.00043881 | 2.4148407 |
| --- | --- | --- | --- | --- | --- | --- |
| Pxk | 1451253_at -0.6113657 | 8.73169827 | -6.0365944 | 0.00028772 | 0.00233571 | -0.1339232 |
| Supt20 | 1449738_s_a-0.6107995 | 8.94977877 | -6.2052311 | 0.00023832 | 0.00202 | 0.0716154 |
| Vrk1 | 1425006_a_ -0.6107699 | 7.71262873 | -6.4631471 | 0.00017988 | 0.00164973 | 0.37889184 |
| Pik3cg | 1422708_at -0.6102334 | 6.96206938 | -7.7452587 | 4.96E-05 | 0.00064046 | 1.78861754 |
| Poldip3 | 1437335_x_a-0.6102246 | 10.0028869 | -7.7666517 | 4.86E-05 | 0.00063141 | 1.8105978 |
| Serpinb1a | 1416318_at -0.6097118 | 7.65743359 | -9.267297 | 1.31E-05 | 0.00025761 | 3.24289042 |
| Myo9b | 1418031_at -0.6097063 | 9.29825819 | -4.6929813 | 0.00147699 | 0.00783193 | -1.9101361 |
| Epb41 | 1424092_at -0.6096719 | 10.1726088 | -2.5631795 | 0.03297058 | 0.08601844 | -5.1829305 |
| Nab2 | 1417930_at -0.6091259 | 8.75672531 | -5.7154876 | 0.00041597 | 0.00301643 | -0.5357121 |
| Bcr | 1452368_at -0.6090666 | 8.21297809 | -9.3691573 | 1.21E-05 | 0.0002441 | 3.33291074 |
| Lrrc59 | 1416235_at -0.6090003 | 8.94660644 | -6.7925698 | 0.00012701 | 0.00126791 | 0.75930697 |
| Actn4 | 1423449_a_ -0.6084833 | 11.2840321 | -7.0818277 | 9.45E-05 | 0.00102172 | 1.08262789 |
| Nadk | 1416248_at -0.6082025 | 9.95207414 | -3.9966296 | 0.00381651 | 0.0162898 | -2.9297409 |
| Nap1l1 | 1420478_at -0.6078422 | 10.7885424 | -5.1009376 | 0.00087529 | 0.00522914 | -1.3441161 |
| Mefv | 1460283_at -0.6077837 | 8.29420689 | -7.72585 | 5.05E-05 | 0.00064864 | 1.76863504 |
| Nme1 | 1424110_a_ -0.6077471 | 9.74387763 | -7.6579373 | 5.39E-05 | 0.00067854 | 1.69840528 |
| Dcaf13 | 1435442_at -0.6077246 | 9.7211489 | -8.1938789 | 3.29E-05 | 0.00048681 | 2.23984931 |
| Fnbp1 | 1426983_at -0.6075448 | 9.74526173 | -5.3008501 | 0.00068323 | 0.00436079 | -1.0753503 |
| Shkbp1 | 1424174_at -0.6067266 | 9.43721459 | -7.6697658 | 5.33E-05 | 0.0006746 | 1.71067202 |
| sep-06 | 1420876_a_ -0.6066883 | 6.96195932 | -6.5466582 | 0.00016449 | 0.00154295 | 0.47658997 |
| NA | 1452487_x_a-0.6066422 | 7.16580719 | -6.5050911 | 0.00017196 | 0.00159691 | 0.42806971 |
| NA | 1422115_a_ -0.6061199 | 7.22948864 | -6.3380424 | 0.00020597 | 0.00181497 | 0.23090007 |
| Ctsl | 1451310_a_ -0.6060821 | 11.9796495 | -4.7878695 | 0.00130494 | 0.00712958 | -1.776379 |
| Lpp | 1436714_at -0.6057753 | 9.85497267 | -4.9897219 | 0.00100708 | 0.0058352 | -1.4960833 |
| Tor1a | 1426515_a_ -0.6055577 | 7.77231618 | -6.4845292 | 0.00017579 | 0.00162409 | 0.40398908 |
| NA | 1426936_at -0.6054432 | 7.3636055 | -2.2597307 | 0.05313562 | 0.12297504 | -5.6609727 |
| Sharpin | 1424165_a_ -0.6053065 | 8.62033676 | -9.3154554 | 1.26E-05 | 0.00025102 | 3.28555615 |
| Pdlim5 | 1450786_x_a-0.6053046 | 8.30167537 | -3.9480736 | 0.0040885 | 0.01717609 | -3.003217 |
| Sertad1 | 1417406_at -0.6051356 | 8.73240229 | -9.0345574 | 1.59E-05 | 0.00029331 | 3.03399061 |
| Uba2 | 1433440_x_a-0.6050327 | 9.75840028 | -9.8417865 | 8.35E-06 | 0.00018774 | 3.7398486 |
| Pdk3 | 1426410_at -0.6050312 | 8.13971017 | -5.202029 | 0.00077169 | 0.00475549 | -1.2075023 |
| Frmd8 | 1432848_a_ -0.6040732 | 9.20737287 | -7.2634257 | 7.89E-05 | 0.00089274 | 1.28068469 |
| Nfkbiz | 1448728_a_ -0.6039349 | 8.19317389 | -7.0798338 | 9.47E-05 | 0.00102266 | 1.08043256 |
| Rb1 | 1417850_at -0.6038931 | 9.15567362 | -7.6277601 | 5.54E-05 | 0.00068982 | 1.66704324 |
| Krt18 | 1448169_at -0.6038873 | 8.13386718 | -3.0202534 | 0.01618966 | 0.04991599 | -4.4532896 |
| NA | 1428301_at -0.6038115 | 7.81671042 | -4.9107438 | 0.00111375 | 0.00630199 | -1.6050655 |
| Spag5 | 1433893_s_a-0.6037751 | 6.71264571 | -5.9141805 | 0.00033061 | 0.0025801 | -0.2854671 |
| Mob1a | 1424484_at -0.6034294 | 8.51743828 | -5.8566029 | 0.00035318 | 0.0026991 | -0.3574363 |
| Dnase1l1 | 1429173_at -0.6033289 | 8.46408928 | -5.2134554 | 0.00076085 | 0.00470274 | -1.1921516 |
| Sparc | 1416589_at -0.6031347 | 12.1874528 | -4.4298193 | 0.00209674 | 0.01028207 | -2.2877099 |
| Tab2 | 1423462_at -0.603037 | 10.4726336 | -10.297252 | 5.92E-06 | 0.00014595 | 4.11610786 |
| Atp13a2 | 1452746_at -0.6030239 | 9.9418289 | -7.6676498 | 5.34E-05 | 0.00067533 | 1.70847878 |
| Dnajc9 | 1426473_at -0.6025514 | 8.55333516 | -4.6777979 | 0.00150673 | 0.00795249 | -1.9316571 |
| Tnfaip1 | 1448863_a_ -0.6025427 | 9.9603028 | -6.9800872 | 0.00010476 | 0.00109845 | 0.97002076 |
| Sfpq | 1423796_at -0.6021924 | 7.89699487 | -7.4606552 | 6.51E-05 | 0.00077728 | 1.49162453 |
| B3galnt1 | 1418736_at -0.6021561 | 7.56472854 | -4.8651667 | 0.00118086 | 0.00660105 | -1.6683603 |
| Tars | 1460323_at -0.601711 | 9.20127452 | -8.5581616 | 2.38E-05 | 0.00039044 | 2.59185842 |
| Sec61a1 | 1448242_at -0.6016681 | 10.0143122 | -7.0753371 | 9.51E-05 | 0.00102646 | 1.07547966 |
| Bub3 | 1416815_s_a-0.6014369 | 10.6264566 | -8.0051335 | 3.90E-05 | 0.00054432 | 2.05245794 |
| Spcs2 | 1450907_at -0.6011383 | 10.1838529 | -7.8393284 | 4.54E-05 | 0.0006039 | 1.88491699 |
| Fam76b | 1426986_at -0.6010797 | 7.92414223 | -6.4592004 | 0.00018064 | 0.00165474 | 0.37425316 |
| Noc4l | 1423826_at -0.6007283 | 9.35494182 | -7.9098718 | 4.26E-05 | 0.00057986 | 1.95653822 |
| Ddx39 | 1423643_at -0.60048 | 9.49840664 | -7.18544 | 8.52E-05 | 0.00094451 | 1.19608753 |
| Ccng2 | 1416488_at -0.600412 | 8.68133981 | -4.8185612 | 0.00125405 | 0.00690461 | -1.7333877 |
| Tank | 1421640_a_ -0.6000728 | 7.94839869 | -5.9539375 | 0.00031596 | 0.00249532 | -0.2360312 |
| Snhg8 | 1435524_at -0.5997488 | 9.71296246 | -6.4354531 | 0.00018532 | 0.00168335 | 0.34630118 |
| Tbcb | 1448533_at -0.5992359 | 11.0992886 | -7.5789496 | 5.81E-05 | 0.00071482 | 1.61611253 |
| Magohb | 1450943_at -0.5991225 | 8.33651105 | -5.8528402 | 0.00035471 | 0.00270716 | -0.3621549 |
| Lox | 1416121_at -0.5991036 | 8.39841861 | -5.9566163 | 0.00031499 | 0.00249033 | -0.2327079 |
| Ap2s1 | 1433612_at -0.5983209 | 10.005238 | -5.7717772 | 0.00038956 | 0.00288863 | -0.4642764 |
| Psmd11 | 1437080_s_a-0.5981567 | 12.0365163 | -9.7988453 | 8.63E-06 | 0.00019148 | 3.70358728 |
| Eif3a | 1448425_at -0.5977574 | 11.0002949 | -5.1893185 | 0.00078395 | 0.00480506 | -1.2245998 |
| NA | 1435715_x_a-0.5976899 | 9.31205292 | -9.0327982 | 1.59E-05 | 0.00029347 | 3.03239426 |
| Nucb2 | 1418355_at -0.5975497 | 8.30555755 | -7.6165187 | 5.60E-05 | 0.00069384 | 1.65533587 |
| Lpar1 | 1448606_at -0.596907 | 8.88350433 | -5.9904985 | 0.00030311 | 0.00242336 | -0.190755 |
| Cebpg | 1451639_at -0.5967707 | 8.32982278 | -4.3596506 | 0.00230604 | 0.01107623 | -2.3900056 |
| Sumf1 | 1424604_s_a-0.5966144 | 8.50429913 | -5.8837596 | 0.00034233 | 0.0026429 | -0.3234364 |
| Ccnl1 | 1423622_a_ -0.5964382 | 9.62486047 | -7.1067308 | 9.22E-05 | 0.00100453 | 1.11000948 |
| Hps3 | 1450647_at -0.5964222 | 8.72831595 | -6.3156258 | 0.00021108 | 0.00184417 | 0.20417338 |
| Arntl | 1425099_a_ -0.596223 | 8.62097425 | -4.3706386 | 0.00227183 | 0.01094433 | -2.3739422 |
| Itm2c | 1415961_at -0.5959934 | 10.6392539 | -5.381408 | 0.0006193 | 0.00405541 | -0.9686365 |
| Usp1 | 1423674_at -0.5959868 | 8.07855612 | -7.0518348 | 9.74E-05 | 0.00104313 | 1.04955571 |
| Bzw1 | 1450845_a_ -0.5957861 | 9.77857703 | -8.571912 | 2.35E-05 | 0.00038765 | 2.6049035 |
| Uba2 | 1416280_at -0.5949396 | 9.93469047 | -5.5546858 | 0.00050285 | 0.00346064 | -0.7421664 |
| Acvrl1 | 1451604_a_ -0.5948708 | 8.7609415 | -4.7088124 | 0.00144665 | 0.00770989 | -1.8877319 |
| NA | 1439121_at -0.5944785 | 8.14522252 | -7.2141161 | 8.28E-05 | 0.00092385 | 1.22727408 |
| Aftph | 1426861_at -0.5943367 | 9.45802303 | -5.4350952 | 0.00058034 | 0.00385996 | -0.898022 |
| Cct2 | 1433534_a_ -0.594313 | 10.6012794 | -8.6676717 | 2.17E-05 | 0.00036462 | 2.69527469 |
| Ldlrap1 | 1424378_at -0.594127 | 8.34737553 | -7.698638 | 5.18E-05 | 0.00066025 | 1.74055249 |
| Arhgef3 | 1424250_a_ -0.5937931 | 7.92415888 | -5.9461867 | 0.00031876 | 0.00251219 | -0.2456525 |
| Syce2 | 1429270_a_ -0.5933181 | 8.10045658 | -7.4233545 | 6.74E-05 | 0.00079722 | 1.45205776 |
| Cttn | 1433908_a_ -0.5930486 | 10.0308049 | -8.7472351 | 2.03E-05 | 0.00034758 | 2.76973351 |
| Pglyrp1 | 1449184_at -0.5922454 | 7.62881802 | -4.0113868 | 0.00373774 | 0.01603509 | -2.9074679 |
| Plagl2 | 1417517_at -0.5919347 | 7.98755543 | -4.5170733 | 0.00186467 | 0.0093979 | -2.1614518 |

| Wdr26 | 1451188_at | -0.5918853 | 10.63927 | -7.976605 | 4.00E-05 | 0.00055532 | 2.0238278 |
| --- | --- | --- | --- | --- | --- | --- | --- |
| NA | 1424784_at | -0.5917847 | 8.63748372 | -6.4932812 | 0.00017415 | 0.00161483 | 0.41424519 |
| Poldip3 | 1452709_at | -0.5914682 | 9.12220039 | -4.8708617 | 0.00117224 | 0.00656742 | -1.6604352 |
| Xrn2 | 1422842_at | -0.5912132 | 8.54596008 | -4.326285 | 0.00241339 | 0.01147988 | -2.4388836 |
| Pole2 | 1427094_at | -0.59111 | 7.37378698 | -7.3978318 | 6.91E-05 | 0.00080864 | 1.42489729 |
| Usp39 | 1437007_x_a-0.5910573 | | 9.24531939 | -8.3281286 | 2.91E-05 | 0.0004489 | 2.37103198 |
| Rbm8a | 1418119_at -0.5910541 | | 10.2358565 | -7.1730072 | 8.63E-05 | 0.00095255 | 1.18253746 |
| Dna2 | 1452210_at -0.5909263 | | 7.77528929 | -7.0533091 | 9.73E-05 | 0.00104207 | 1.05118371 |
| Gemin6 | 1424300_at -0.590912 | | 8.56777146 | -7.372146 | 7.09E-05 | 0.00082319 | 1.39749135 |
| Nfatc1 | 1417621_at -0.590703 | | 9.00099057 | -4.6800657 | 0.00150225 | 0.00793464 | -1.9284407 |
| Slc31a2 | 1453721_a_ -0.5906838 | | 9.26982894 | -5.4521232 | 0.00056855 | 0.0038032 | -0.8757092 |
| Cln8 | 1459992_x_a-0.5905498 | | 8.88408106 | -6.6356804 | 0.00014968 | 0.00143905 | 0.5797863 |
| NA | 1456606_a_ -0.5905496 | | 6.70137033 | -7.8163107 | 4.64E-05 | 0.00061223 | 1.86143773 |
| Cct2 | 1416037_a_ -0.5905096 | | 10.8774511 | -8.0286265 | 3.82E-05 | 0.00053706 | 2.07597366 |
| Hnrnph2 | 1415963_at -0.5900298 | | 10.4683124 | -6.8074811 | 0.00012507 | 0.0012562 | 0.7762152 |
| Mbp | 1425264_s_a-0.5897466 | | 8.56750938 | -4.6847551 | 0.00149302 | 0.00790035 | -1.9217919 |
| NA | 1424783_a_ -0.58925 | | 8.92662624 | -4.3307632 | 0.00239867 | 0.01142679 | -2.4323145 |
| NA | 1426793_a_ -0.5891568 | | 11.0012079 | -5.8194825 | 0.00036862 | 0.00278336 | -0.4040709 |
| Pdcd10 | 1448528_at -0.5889793 | | 8.11056178 | -8.3885877 | 2.76E-05 | 0.00043475 | 2.42954986 |
| NA | 1448319_at -0.5884592 | | 12.2507995 | -7.9722108 | 4.02E-05 | 0.00055646 | 2.01941078 |
| Trip12 | 1423898_a_ -0.5881723 | | 10.7529472 | -7.7949504 | 4.73E-05 | 0.00062002 | 1.83960059 |
| Gdi2 | 1436016_x_a-0.5881341 | | 11.9761235 | -6.5048467 | 0.00017201 | 0.00159691 | 0.42778382 |
| Stx7 | 1418436_at -0.5879174 | | 10.6961802 | -6.5901246 | 0.00015707 | 0.0014949 | 0.5270987 |
| Tle4 | 1450853_at -0.5876723 | | 8.88847339 | -5.5455358 | 0.00050836 | 0.00348893 | -0.7540211 |
| Ampd2 | 1438941_x_a-0.5876253 | | 7.6096153 | -5.4171263 | 0.00059308 | 0.00392787 | -0.9216116 |
| Mtpn | 1420472_at -0.5876061 | | 8.85661377 | -6.78112 | 0.00012853 | 0.00127901 | 0.74630588 |
| Ttc39c | 1426223_at -0.5870314 | | 8.26854599 | -8.7516076 | 2.02E-05 | 0.00034689 | 2.77380906 |
| Clmp | 1448250_at -0.5868188 | | 8.31173336 | -6.5484162 | 0.00016419 | 0.0015407 | 0.47863735 |
| NA | 1426261_s_a-0.586637 | | 7.64597939 | -3.5238741 | 0.00756634 | 0.02761019 | -3.6566676 |
| Mmp13 | 1417256_at -0.586487 | | 6.74260658 | -6.5689151 | 0.00016064 | 0.00151749 | 0.50248196 |
| Psmd7 | 1451056_at -0.5864585 | | 9.41464109 | -6.4208187 | 0.00018828 | 0.00170106 | 0.3290404 |
| Cpt1a | 1434866_x_a-0.5860094 | | 7.58027169 | -5.3629456 | 0.00063336 | 0.00412718 | -0.9930131 |
| Eps15 | 1419251_at -0.5854954 | | 8.48605376 | -5.3381807 | 0.00065275 | 0.00422206 | -1.0257861 |
| Atp6v0c | 1435732_x_a-0.5846031 | | 12.4881096 | -8.5662177 | 2.37E-05 | 0.000389 | 2.59950331 |
| Slc31a2 | 1416654_at -0.5843096 | | 9.54131332 | -5.9968408 | 0.00030094 | 0.00241196 | -0.1829189 |
| Dclk1 | 1424271_at -0.5841938 | | 7.89432415 | -6.5679606 | 0.00016081 | 0.00151809 | 0.50137289 |
| Rps10 | 1434854_a_ -0.5839603 | | 12.3938511 | -8.2845686 | 3.03E-05 | 0.00046103 | 2.32865605 |
| Gtpbp2 | 1416690_at -0.5838556 | | 9.73017805 | -8.5392029 | 2.42E-05 | 0.00039564 | 2.57384382 |
| Sav1 | 1448204_at -0.5835882 | | 9.46504677 | -8.7602556 | 2.00E-05 | 0.00034509 | 2.78186488 |
| Lypla2 | 1417433_at -0.5834997 | | 10.7448505 | -6.347833 | 0.00020379 | 0.0017985 | 0.24255318 |
| Il17ra | 1420904_at -0.5834041 | | 8.00130072 | -6.834276 | 0.00012165 | 0.00123387 | 0.8065318 |
| Dnajb6 | 1448234_at -0.5832947 | | 9.8694697 | -7.6332319 | 5.51E-05 | 0.0006887 | 1.67273702 |
| Fuca1 | 1416109_at -0.5828187 | | 9.87772631 | -3.2484047 | 0.01142893 | 0.03818058 | -4.0903455 |
| Selp | 1420558_at -0.5827739 | | 8.43449231 | -6.6929833 | 0.00014092 | 0.00137821 | 0.64570023 |
| Fnbp4 | 1423191_at -0.5823959 | | 8.29231255 | -6.7134542 | 0.00013793 | 0.00135188 | 0.66915071 |
| Grk2 | 1451992_at -0.5822485 | | 9.32915408 | -4.3643251 | 0.00229142 | 0.01102233 | -2.38317 |
| Nap1l1 | 1437945_x_a-0.5821495 | | 9.92348603 | -5.6142129 | 0.00046857 | 0.00328343 | -0.6653254 |
| Cd28 | 1437025_at -0.5819634 | | 8.33679972 | -5.3332794 | 0.00065667 | 0.00424135 | -1.0322824 |
| NA | 1435725_x_a-0.5817725 | | 12.5355367 | -8.7497707 | 2.02E-05 | 0.00034713 | 2.77209717 |
| Plk4 | 1426580_at -0.5813076 | | 7.62065579 | -5.3074866 | 0.0006777 | 0.00433402 | -1.0665247 |
| NA | 1429775_a_ -0.5808138 | | 7.84434796 | -4.2244516 | 0.00277571 | 0.01276206 | -2.5889898 |
| Mcl1 | 1456243_x_a-0.5806597 | | 11.098883 | -8.0640448 | 3.69E-05 | 0.00052371 | 2.11132277 |
| Vps54 | 1449095_at -0.5802346 | | 9.19849007 | -6.0900182 | 0.00027095 | 0.00222908 | -0.0684069 |
| Selp | 1449906_at -0.5801855 | | 7.54009632 | -6.7941409 | 0.00012681 | 0.00126676 | 0.76108978 |
| Rspry1 | 1424135_at -0.5799879 | | 7.71864286 | -4.4516794 | 0.00203581 | 0.01005714 | -2.255979 |
| Glmp | 1416830_at -0.5799237 | | 9.84290982 | -4.7035222 | 0.00145671 | 0.00774887 | -1.8952145 |
| Casp7 | 1426062_a_ -0.579843 | | 8.35981342 | -5.4176572 | 0.0005927 | 0.0039265 | -0.920914 |
| Morf4l2 | 1439432_x_a-0.5794463 | | 10.8895685 | -9.136663 | 1.46E-05 | 0.00027702 | 3.12619436 |
| Nubp2 | 1416512_at -0.5794462 | | 9.08135219 | -6.9455534 | 0.00010851 | 0.00112581 | 0.9315256 |
| Sod3 | 1417633_at -0.5793546 | | 10.1475659 | -2.7232795 | 0.02565901 | 0.07143594 | -4.9277687 |
| Tm9sf3 | 1416509_at -0.5790881 | | 10.6584323 | -7.870943 | 4.41E-05 | 0.00059231 | 1.91707716 |
| Tradd | 1429117_at -0.5788381 | | 7.72311627 | -6.0611613 | 0.00027987 | 0.00228564 | -0.103749 |
| NA | 1436747_at -0.5787618 | | 10.5216799 | -5.9173346 | 0.00032942 | 0.00257479 | -0.2815373 |
| NA | 1423090_x_a-0.5785203 | | 11.2768955 | -5.2462208 | 0.00073068 | 0.00455971 | -1.1482353 |
| Rpl41 | 1422623_x_a-0.5784779 | | 13.730654 | -8.7850711 | 1.96E-05 | 0.00033994 | 2.80494435 |
| Stil | 1427707_a_ -0.5780004 | | 7.11001991 | -7.6192869 | 5.59E-05 | 0.00069384 | 1.65822011 |
| Sp1 | 1418180_at -0.5779787 | | 8.79762893 | -5.7901762 | 0.00038134 | 0.00284903 | -0.4410198 |
| Parp8 | 1451474_a_ -0.5778272 | | 7.61471588 | -5.1266104 | 0.00084761 | 0.00510007 | -1.3092854 |
| Jpt1 | 1416028_a_ -0.5777058 | | 10.4013182 | -7.9955975 | 3.93E-05 | 0.00054792 | 2.04289698 |
| Sec11a | 1418223_at -0.5770313 | | 10.6545822 | -6.3862365 | 0.00019546 | 0.00174616 | 0.28814476 |
| Rad51ap1 | 1448899_s_a-0.5770049 | | 7.25203741 | -5.5030078 | 0.00053485 | 0.00363199 | -0.809272 |
| Cnot6 | 1426682_at -0.576979 | | 9.78158176 | -5.9947282 | 0.00030166 | 0.00241522 | -0.1855285 |
| Cd2bp2 | 1448624_at -0.5767724 | | 8.58427739 | -5.1709399 | 0.00080205 | 0.00489471 | -1.249362 |
| Igbp1 | 1420000_s_a-0.5764856 | | 10.0384112 | -5.0232017 | 0.00096526 | 0.00565793 | -1.4501514 |
| Nubp1 | 1430778_a_ -0.5764089 | | 7.93204503 | -5.5697753 | 0.00049391 | 0.00341046 | -0.7226418 |
| Mfsd1 | 1437055_x_a-0.576306 | | 7.77985475 | -7.9927327 | 3.94E-05 | 0.00054885 | 2.04002291 |
| Cpxm1 | 1448901_at -0.5759996 | | 7.4547723 | -3.2773454 | 0.01093955 | 0.03686693 | -4.0445122 |
| Fbxo32 | 1417522_at -0.5754922 | | 9.54621144 | -4.651648 | 0.0015595 | 0.00816182 | -1.9687979 |
| Srpk1 | 1454042_a_ -0.5753143 | | 8.51393214 | -6.2756182 | 0.00022053 | 0.00191207 | 0.15631425 |
| N4bp2l1 | 1417707_at -0.5750973 | | 7.27862073 | -4.0811987 | 0.00338803 | 0.01488948 | -2.8024725 |
| Sh3kbp1 | 1432269_a_ -0.5749086 | | 7.7475545 | -7.2141991 | 8.28E-05 | 0.00092385 | 1.22736427 |
| Mgat2 | 1452037_at -0.57416 | | 9.41451275 | -4.6569193 | 0.0015487 | 0.00812298 | -1.9613034 |
| Morf4l2 | 1439413_x_a-0.5740274 | | 11.6376494 | -8.8273403 | 1.89E-05 | 0.00033156 | 2.84413141 |
| Crbn | 1423095_s_a-0.5738129 | | 10.0204979 | -6.4188229 | 0.00018868 | 0.00170221 | 0.32668425 |
| Cyp4v3 | 1417070_at -0.5737535 | | 7.22634099 | -4.756189 | 0.00135983 | 0.00736026 | -1.8208952 |

| Fkbp2 | 1450694_at -0.5736671 | 9.41626833 | -5.6625484 | 0.00044261 | 0.0031581 | -0.6032897 |
| --- | --- | --- | --- | --- | --- | --- |
| Fkbp1a | 1456196_x_a-0.57365 | 11.7228388 | -7.6665202 | 5.34E-05 | 0.00067533 | 1.70730763 |
| Il18 | 1417932_at -0.5735158 | 8.23429581 | -4.7160946 | 0.00143292 | 0.00765019 | -1.8774378 |
| Mogs | 1422489_at -0.5734 | 8.84139892 | -5.6145709 | 0.00046837 | 0.00328306 | -0.6648649 |
| Cetn3 | 1417239_at -0.5732484 | 9.20375592 | -6.1334887 | 0.00025809 | 0.00214432 | -0.0153728 |
| Tnfrsf18 | 1422303_a_ -0.5731268 | 7.84987714 | -6.7466936 | 0.00013322 | 0.00131615 | 0.70712031 |
| Lamp2 | 1428094_at -0.5729622 | 11.8758857 | -6.8588422 | 0.0001186 | 0.00120949 | 0.83425176 |
| Casp8ap2 | 1449217_at -0.5729109 | 8.4948557 | -6.1385501 | 0.00025664 | 0.00213538 | -0.0092139 |
| Kdm1a | 1426762_s_a-0.5728533 | 9.52524117 | -7.2274903 | 8.17E-05 | 0.00091483 | 1.24178757 |
| Hnrnpab | 1415914_at -0.5727724 | 11.8119364 | -7.6947046 | 5.20E-05 | 0.0006616 | 1.73648688 |
| Rpn1 | 1438943_x_a-0.5723995 | 11.1383922 | -6.5266326 | 0.00016805 | 0.00156655 | 0.45324129 |
| Casp2 | 1448165_at -0.5723169 | 8.78953423 | -4.5517691 | 0.00178025 | 0.00906503 | -2.11154 |
| Tut7 | 1433910_at -0.5722496 | 9.93876617 | -7.8793922 | 4.38E-05 | 0.00058936 | 1.92565493 |
| Hif1a | 1448183_a_ -0.5720346 | 8.11449303 | -7.1882042 | 8.50E-05 | 0.0009433 | 1.19909781 |
| Os9 | 1426974_at -0.5719068 | 10.0632767 | -4.622059 | 0.00162163 | 0.0084237 | -2.0109393 |
| NA | 1420875_at -0.5718364 | 9.19657927 | -6.1621077 | 0.00025 | 0.00209393 | 0.019408 |
| Srsf1 | 1453722_s_a-0.5716588 | 10.6275058 | -7.6194011 | 5.59E-05 | 0.00069384 | 1.65833903 |
| Api5 | 1437593_x_a-0.5715295 | 10.6797108 | -7.0018599 | 0.00010247 | 0.00107991 | 0.99421958 |
| Mcl1 | 1437527_x_a-0.5710078 | 11.8951188 | -7.8474914 | 4.51E-05 | 0.00060068 | 1.89323057 |
| Rest | 1425565_at -0.5707037 | 8.86115895 | -6.4127524 | 0.00018992 | 0.00171007 | 0.31951494 |
| Mbd2 | 1417165_at -0.5706947 | 10.3935547 | -8.2080303 | 3.24E-05 | 0.00048233 | 2.25375897 |
| Seh1l | 1424202_at -0.5703968 | 8.0091813 | -7.4941565 | 6.30E-05 | 0.00075812 | 1.52703254 |
| Cmtr1 | 1451082_at -0.5703311 | 9.48357827 | -5.0471929 | 0.00093646 | 0.00552049 | -1.417335 |
| Gnb2 | 1450623_at -0.5702781 | 10.852412 | -4.7124336 | 0.00143981 | 0.00767964 | -1.882612 |
| Hnrnpr | 1427129_a_ -0.5700552 | 9.99112552 | -6.4698844 | 0.00017858 | 0.00164048 | 0.38680597 |
| Dnajc2 | 1417657_s_a-0.5693001 | 9.21994084 | -5.2960966 | 0.00068723 | 0.00437887 | -1.0816756 |
| Sec61a1 | 1416191_at -0.5690701 | 8.95127212 | -4.5147532 | 0.00187047 | 0.00941523 | -2.1647954 |
| Arap2 | 1452291_at -0.5687288 | 8.81460468 | -5.8314742 | 0.00036355 | 0.00275518 | -0.3889853 |
| Fam107b | 1416893_at -0.5687118 | 8.33027291 | -6.0651266 | 0.00027862 | 0.00227655 | -0.0988862 |
| Nsf | 1422456_at -0.5683671 | 9.18512582 | -5.5835296 | 0.00048591 | 0.00337149 | -0.7048722 |
| Set | 1421819_a_ -0.5683668 | 10.3119362 | -5.8072197 | 0.00037388 | 0.00281099 | -0.4195176 |
| Slc25a24 | 1427483_at -0.5683359 | 7.44858905 | -4.9124294 | 0.00111135 | 0.00628997 | -1.6027303 |
| Sema3f | 1425840_a_ -0.5682778 | 8.23672638 | -5.6246796 | 0.00046281 | 0.00326019 | -0.6518649 |
| Capn1 | 1417229_at -0.5679665 | 8.53619274 | -3.1603628 | 0.01306409 | 0.04232817 | -4.2300953 |
| Pom121 | 1427886_at -0.5678784 | 10.3267263 | -8.211236 | 3.23E-05 | 0.00048158 | 2.25690725 |
| Nap1l1 | 1420476_a_ -0.5678309 | 9.21166833 | -5.8035936 | 0.00037546 | 0.00281676 | -0.424089 |
| Vamp8 | 1420624_a_ -0.5677955 | 10.9923025 | -5.1248353 | 0.0008495 | 0.00510598 | -1.3116906 |
| Tmed3 | 1416108_a_ -0.5677335 | 9.86708608 | -4.1411666 | 0.00311564 | 0.01399238 | -2.7127797 |
| Zc3h7a | 1419898_s_a-0.5676746 | 7.73439253 | -8.1692239 | 3.36E-05 | 0.00049394 | 2.21556905 |
| Ddx21 | 1448270_at -0.56728 | 8.60513692 | -7.7997865 | 4.71E-05 | 0.00061815 | 1.84454876 |
| Srsf3 | 1416150_a_ -0.5669177 | 10.7041602 | -5.9541199 | 0.00031589 | 0.00249532 | -0.2358049 |
| Haus6 | 1455488_at -0.5667717 | 7.2495355 | -5.9231139 | 0.00032726 | 0.00256314 | -0.2743404 |
| Dap | 1451112_s_a-0.5665621 | 10.3661831 | -5.2881889 | 0.00069392 | 0.00440669 | -1.0922053 |
| Sf3a3 | 1423811_at -0.5665614 | 8.79793305 | -6.3541949 | 0.00020238 | 0.00179015 | 0.25011869 |
| Cebpg | 1425262_at -0.5664414 | 9.36956494 | -5.2386971 | 0.00073749 | 0.00458735 | -1.1583061 |
| Col4a2 | 1424051_at -0.5661416 | 11.2904004 | -7.3908986 | 6.96E-05 | 0.00081247 | 1.41750688 |
| Rps11 | 1424000_a_ -0.5661285 | 12.9124766 | -8.1779625 | 3.33E-05 | 0.00049167 | 2.22418161 |
| Ltb4r1 | 1420407_at -0.5658875 | 7.52867632 | -8.4474762 | 2.62E-05 | 0.00042012 | 2.48621774 |
| Col6a3 | 1424131_at -0.5658563 | 10.0649705 | -7.8698988 | 4.42E-05 | 0.00059246 | 1.91601656 |
| Rab11a | 1449256_a_ -0.5656062 | 10.8034875 | -6.1038274 | 0.00026679 | 0.0022013 | -0.0515329 |
| Bola2 | 1434543_a_ -0.5654098 | 9.54751887 | -6.8651327 | 0.00011784 | 0.00120222 | 0.84133844 |
| Rpl7 | 1426162_a_ -0.5653719 | 12.3487998 | -7.6402954 | 5.48E-05 | 0.00068542 | 1.68008232 |
| Ccnd2 | 1430127_a_ -0.5652635 | 10.6485986 | -5.7745625 | 0.00038831 | 0.00288149 | -0.4607528 |
| Skil | 1452214_at -0.565236 | 10.1964624 | -5.6235927 | 0.0004634 | 0.00326235 | -0.653262 |
| Pak2 | 1454887_at -0.5651122 | 9.10386953 | -5.3242661 | 0.00066394 | 0.00427203 | -1.0442377 |
| Atad2b | 1428982_at -0.5651031 | 8.44458531 | -6.3307982 | 0.00020761 | 0.00182511 | 0.22227011 |
| Atp8a1 | 1423597_at -0.5646948 | 9.62604779 | -5.3496098 | 0.00064372 | 0.00417838 | -1.0106504 |
| Rmi1 | 1430805_s_a-0.5644322 | 7.68288951 | -9.3692871 | 1.21E-05 | 0.0002441 | 3.33302488 |
| Tnfrsf12a | 1418572_x_a-0.5644248 | 10.9944121 | -6.2986724 | 0.00021503 | 0.00187409 | 0.18391788 |
| Trmt112 | 1425299_s_a-0.5641317 | 10.7397123 | -7.8835476 | 4.36E-05 | 0.0005878 | 1.92987098 |
| Blnk | 1451780_at -0.5640201 | 8.2441755 | -4.5234543 | 0.00184882 | 0.0093367 | -2.1522598 |
| Ap3b1 | 1423235_at -0.5637336 | 9.96494872 | -4.5965891 | 0.00167726 | 0.00864736 | -2.0473125 |
| Cd2bp2 | 1417224_a_ -0.5635336 | 7.73694476 | -7.2471538 | 8.02E-05 | 0.00090254 | 1.26308964 |
| Gna15 | 1421302_a_ -0.5635178 | 7.63114786 | -4.8597453 | 0.00118913 | 0.00663584 | -1.6759088 |
| Cggbp1 | 1454641_at -0.5634956 | 10.5293489 | -5.8657207 | 0.00034949 | 0.00267636 | -0.3460098 |
| Gdi2 | 1456581_x_a-0.5634705 | 11.7945299 | -6.82945 | 0.00012226 | 0.00123771 | 0.80107779 |
| Slc7a8 | 1417929_at -0.5634425 | 8.20326156 | -4.7266375 | 0.0014133 | 0.00757565 | -1.8625479 |
| Tiam1 | 1422993_s_a-0.5632822 | 8.94638112 | -5.492281 | 0.00054177 | 0.00366213 | -0.8232474 |
| Aup1 | 1415742_at -0.5631893 | 9.03634352 | -4.5294208 | 0.00183414 | 0.00927907 | -2.14367 |
| Wdr43 | 1428389_s_a-0.5631718 | 8.69647422 | -9.325349 | 1.25E-05 | 0.00024924 | 3.29429792 |
| Ppil2 | 1452920_a_ -0.563159 | 9.23839156 | -5.5753257 | 0.00049066 | 0.00339735 | -0.7154679 |
| Nfkb2 | 1425902_a_ -0.563122 | 8.95728781 | -5.3568748 | 0.00063805 | 0.00415301 | -1.001039 |
| Oas2 | 1425065_at -0.5628446 | 9.25537674 | -4.2168168 | 0.00280514 | 0.01286357 | -2.6002997 |
| Ckb | 1455106_a_ -0.5628355 | 10.5346577 | -2.9668307 | 0.01757843 | 0.05331646 | -4.5385768 |
| Slc7a7 | 1417392_a_ -0.5628081 | 8.29542105 | -4.0670549 | 0.00345594 | 0.01512253 | -2.8236945 |
| Eif4ebp1 | 1417562_at -0.5627227 | 10.976959 | -6.0063835 | 0.0002977 | 0.00239533 | -0.1711387 |
| Nub1 | 1452777_a_ -0.5620204 | 8.72216804 | -7.1796145 | 8.57E-05 | 0.00094796 | 1.18974076 |
| Rtcb | 1423880_at -0.5618353 | 8.97948972 | -5.6391308 | 0.00045498 | 0.00322509 | -0.6333048 |
| Dpm2 | 1415675_at -0.5617637 | 8.9099177 | -6.1423427 | 0.00025556 | 0.00213028 | -0.0046011 |
| Tmtc4 | 1432360_a_ -0.5613597 | 7.53392363 | -6.7609447 | 0.00013126 | 0.00130112 | 0.72335876 |
| Pdcd4 | 1418840_at -0.5613549 | 9.09383538 | -3.1880069 | 0.01252577 | 0.04099967 | -4.1861674 |
| Frzb | 1416658_at -0.5609601 | 8.0408043 | -5.8560406 | 0.00035341 | 0.00269993 | -0.3581413 |
| NA | 1422327_s_a-0.5609419 | 7.51374326 | -5.0003275 | 0.00099362 | 0.00577789 | -1.481516 |
| Cmtm6 | 1451114_at -0.5608475 | 8.85953209 | -3.8087162 | 0.00499098 | 0.02001506 | -3.2156764 |
| Fam32a | 1423216_a_ -0.5606002 | 8.69208234 | -6.399816 | 0.0001926 | 0.00172801 | 0.30422135 |

| Tbca | 1456205_x_a-0.560509 | 11.0202018 | -6.6119869 | 0.00015347 | 0.00146868 | 0.55241527 |
| --- | --- | --- | --- | --- | --- | --- |
| NA | 1448157_s_a-0.560421 | 13.208606 | -8.7815919 | 1.97E-05 | 0.00034045 | 2.80171179 |
| Slu7 | 1451727_at -0.5603198 | 8.53588662 | -4.3136061 | 0.00245558 | 0.01163682 | -2.4574968 |
| Cysltr1 | 1418944_at -0.5601765 | 7.83171519 | -5.2309217 | 0.0007446 | 0.00461738 | -1.1687223 |
| Tipin | 1426612_at -0.5599404 | 8.42811969 | -8.2512233 | 3.12E-05 | 0.00047052 | 2.29609487 |
| Tmed5 | 1424573_at -0.5599331 | 9.47073605 | -6.7416679 | 0.00013392 | 0.00131945 | 0.70138801 |
| NA | 1433432_x_a-0.5596836 | 13.0920608 | -8.3599569 | 2.83E-05 | 0.00044044 | 2.40188132 |
| Ubfd1 | 1448968_at -0.5596233 | 8.97573992 | -6.2449209 | 0.0002281 | 0.00195694 | 0.11945357 |
| NA | 1416790_a_ -0.5592695 | 9.86463291 | -8.6283487 | 2.24E-05 | 0.0003741 | 2.65826491 |
| Cald1 | 1424769_s_a-0.5592049 | 10.35646 | -7.3118795 | 7.52E-05 | 0.00085915 | 1.33290402 |
| Srsf1 | 1434972_x_a-0.5591758 | 10.9648092 | -5.5198484 | 0.00052419 | 0.00357493 | -0.7873633 |
| F11r | 1424595_at -0.5591078 | 9.96829738 | -5.9091819 | 0.00033251 | 0.00259264 | -0.2916975 |
| Tor3a | 1450454_at -0.5590788 | 7.62322449 | -8.2531688 | 3.12E-05 | 0.00047002 | 2.29799759 |
| Gng10 | 1450649_at -0.5590379 | 9.42381999 | -5.5910427 | 0.0004816 | 0.00334788 | -0.6951768 |
| NA | 1437589_x_a-0.5589859 | 8.6159888 | -7.2401441 | 8.07E-05 | 0.00090612 | 1.25550076 |
| NA | 1454620_x_a-0.5589473 | 12.8620524 | -9.1631526 | 1.43E-05 | 0.0002734 | 3.14997204 |
| Slc44a2 | 1438559_x_a-0.5589011 | 9.32789589 | -4.7420886 | 0.00138506 | 0.00745601 | -1.8407543 |
| Osbpl9 | 1450964_a_ -0.558717 | 10.621253 | -5.2950844 | 0.00068808 | 0.00438026 | -1.083023 |
| Man1a | 1417110_at -0.558481 | 9.11599249 | -5.3855874 | 0.00061617 | 0.00403826 | -0.963125 |
| Pros1 | 1426246_at -0.558449 | 8.87142959 | -4.8337783 | 0.00122963 | 0.00681272 | -1.7121217 |
| NA | 1453467_s_a-0.5579016 | 12.7741242 | -6.4740153 | 0.00017779 | 0.0016352 | 0.39165547 |
| Impa1 | 1436848_x_a-0.5578651 | 8.53009415 | -6.1707556 | 0.00024761 | 0.00207928 | 0.02989695 |
| NA | 1426657_s_a-0.557852 | 7.04207493 | -6.786281 | 0.00012785 | 0.00127453 | 0.75216808 |
| Fasl | 1418803_a_ -0.5576898 | 7.4176891 | -5.8799011 | 0.00034385 | 0.00265192 | -0.3282612 |
| NA | 1453752_at -0.5574505 | 8.67061693 | -8.2288528 | 3.18E-05 | 0.00047812 | 2.27419075 |
| Hspb3 | 1449872_at -0.5572629 | 9.45060115 | -6.4328726 | 0.00018584 | 0.00168669 | 0.34325953 |
| Pgd | 1438627_x_a-0.5571335 | 9.17745464 | -3.09133 | 0.01451677 | 0.04590098 | -4.3399608 |
| Smc1a | 1417831_at -0.5569195 | 8.18120826 | -6.0060771 | 0.00029781 | 0.00239533 | -0.1715168 |
| Dab2 | 1420498_a_ -0.5566455 | 10.1154855 | -4.1140134 | 0.00323594 | 0.01437986 | -2.7533347 |
| Cpsf2 | 1420937_at -0.5564302 | 8.55257499 | -5.1235323 | 0.00085088 | 0.00511295 | -1.3134566 |
| Olfml3 | 1448475_at -0.5559807 | 9.47088475 | -3.6393839 | 0.00638308 | 0.02415032 | -3.4768141 |
| Nabp1 | 1452203_at -0.555405 | 9.70244932 | -9.5045632 | 1.09E-05 | 0.00022658 | 3.45128317 |
| Nup50 | 1450722_at -0.555242 | 8.44730983 | -5.3091603 | 0.00067632 | 0.00432759 | -1.0642999 |
| Tceal9 | 1451230_a_ -0.5551955 | 9.98102061 | -8.1839682 | 3.31E-05 | 0.00048998 | 2.23009635 |
| Eif4a1 | 1430980_a_ -0.5550497 | 11.4646279 | -6.4686394 | 0.00017882 | 0.00164068 | 0.38534386 |
| Gpr146 | 1423632_at -0.5549685 | 8.59607856 | -8.015633 | 3.86E-05 | 0.00054078 | 2.06297444 |
| Ncl | 1415773_at -0.5547324 | 10.2269311 | -5.0768399 | 0.00090216 | 0.00536256 | -1.3768949 |
| Bcl2l11 | 1456005_a_ -0.5540451 | 8.97077768 | -5.6385141 | 0.00045531 | 0.00322643 | -0.6340963 |
| Fpr1 | 1450808_at -0.5537758 | 8.07033293 | -6.3485692 | 0.00020362 | 0.00179776 | 0.24342895 |
| Tacc3 | 1455834_x_a-0.5537113 | 7.80224438 | -6.4184913 | 0.00018875 | 0.00170221 | 0.32629284 |
| BC037034 | 1454820_at -0.5534663 | 8.10690155 | -6.5399368 | 0.00016568 | 0.0015502 | 0.46875876 |
| Maff | 1418936_at -0.5531267 | 8.44839131 | -5.3896334 | 0.00061315 | 0.00402559 | -0.9577916 |
| Arhgef2 | 1421043_s_a-0.5530938 | 8.98552531 | -4.7087142 | 0.00144684 | 0.00770989 | -1.8878707 |
| Hmmr | 1425815_a_ -0.5526616 | 7.85485178 | -6.7799143 | 0.00012869 | 0.00127905 | 0.74493582 |
| Bud31 | 1433611_s_a-0.5526346 | 10.4859275 | -8.9395476 | 1.72E-05 | 0.00030888 | 2.94739936 |
| Atp6v0d1 | 1415671_at -0.5525271 | 10.660739 | -5.7377209 | 0.00040531 | 0.00296164 | -0.5074449 |
| NA | 1436046_x_a-0.5524771 | 12.5707248 | -8.6314677 | 2.24E-05 | 0.00037365 | 2.66120558 |
| Clcf1 | 1437270_a_ -0.552474 | 6.6471109 | -6.9381816 | 0.00010933 | 0.00113225 | 0.92329024 |
| Ewsr1 | 1417238_at -0.552429 | 9.43725255 | -4.2534772 | 0.0026668 | 0.01237095 | -2.5460635 |
| Matr3 | 1438368_a_ -0.5524267 | 10.7807379 | -5.4651758 | 0.00055969 | 0.00375611 | -0.8586327 |
| Ttc39b | 1440192_at -0.5523778 | 7.21454261 | -7.0313127 | 9.95E-05 | 0.00105849 | 1.02686698 |
| Slu7 | 1425487_at -0.5520797 | 7.42731572 | -5.3529397 | 0.00064112 | 0.00416698 | -1.0062442 |
| Arf6 | 1418823_at -0.5520195 | 9.85593858 | -5.220894 | 0.00075388 | 0.00466475 | -1.1821683 |
| Il33 | 1416200_at -0.5519516 | 8.18179111 | -7.8023371 | 4.70E-05 | 0.00061739 | 1.84715751 |
| Nsmaf | 1416412_at -0.5517941 | 7.74076343 | -4.4080035 | 0.00215953 | 0.01052625 | -2.3194418 |
| Armcx2 | 1456739_x_a-0.5516134 | 8.78113891 | -5.267774 | 0.00071154 | 0.00447643 | -1.1194295 |
| Utp11 | 1429485_a_ -0.5516047 | 9.04617558 | -8.2950069 | 3.00E-05 | 0.00045831 | 2.33882707 |
| Pprc1 | 1426381_at -0.5515513 | 8.56606812 | -7.7179408 | 5.09E-05 | 0.0006518 | 1.76048077 |
| Eed | 1448653_at -0.5514015 | 9.68250319 | -6.2741415 | 0.00022089 | 0.00191269 | 0.15454383 |
| Arsa | 1460346_at -0.5513145 | 9.35061542 | -4.499709 | 0.00190854 | 0.00955744 | -2.1864942 |
| NA | 1419858_at -0.5512871 | 7.60316096 | -8.4535006 | 2.61E-05 | 0.00041871 | 2.49199663 |
| Cmpk1 | 1423073_at -0.551161 | 10.5149722 | -4.4607137 | 0.00201118 | 0.00996153 | -2.2428848 |
| Lrg1 | 1417290_at -0.5511255 | 11.270876 | -4.940894 | 0.00107164 | 0.00612328 | -1.5633568 |
| Ppp1r21 | 1428655_at -0.5508991 | 8.9133572 | -6.5214536 | 0.00016898 | 0.001573 | 0.44719481 |
| Meox2 | 1424234_s_a-0.550834 | 8.82709203 | -6.4460348 | 0.00018322 | 0.00167296 | 0.35876522 |
| Nol12 | 1423453_at -0.5508117 | 9.36195016 | -7.2115737 | 8.30E-05 | 0.00092573 | 1.22451285 |
| Rpl13a | 1455001_x_a-0.5507916 | 10.7052582 | -9.1292525 | 1.47E-05 | 0.00027833 | 3.11953194 |
| Trim26 | 1424929_a_ -0.550325 | 8.81583815 | -5.2130876 | 0.0007612 | 0.0047036 | -1.1926456 |
| Plagl2 | 1417519_at -0.5502932 | 7.99530139 | -4.4809294 | 0.00195725 | 0.00974971 | -2.2136248 |
| Bzw1 | 1423040_at -0.5500793 | 9.78104788 | -5.0208117 | 0.00096819 | 0.00566607 | -1.4534251 |
| Rap1gds1 | 1425266_a_ -0.5492708 | 9.75845971 | -4.937354 | 0.0010765 | 0.00614637 | -1.5682472 |
| NA | 1453729_a_ -0.5491021 | 13.3441832 | -8.3050477 | 2.97E-05 | 0.00045516 | 2.34860091 |
| NA | 1422414_a_ -0.5489936 | 10.2205518 | -4.8164926 | 0.00125741 | 0.00691654 | -1.7362811 |
| Rell1 | 1452359_at -0.5488368 | 8.29544086 | -6.56776 | 0.00016084 | 0.00151809 | 0.50113969 |
| Pvr | 1451160_s_a-0.5485428 | 9.48158955 | -5.1958003 | 0.00077767 | 0.00478149 | -1.215878 |
| Erbin | 1428011_a_ -0.5483792 | 9.31034155 | -6.7501956 | 0.00013274 | 0.00131233 | 0.71111297 |
| Apbb1ip | 1425269_at -0.5481357 | 8.16580052 | -4.0119692 | 0.00373467 | 0.01603094 | -2.9065894 |
| Sf3b6 | 1417055_at -0.5481225 | 9.75079334 | -6.3838904 | 0.00019595 | 0.00174871 | 0.28536486 |
| Dph5 | 1452839_at -0.5480841 | 8.50962797 | -8.146789 | 3.43E-05 | 0.00049854 | 2.19342392 |
| NA | 1439444_x_a-0.5480263 | 8.94513514 | -6.9661419 | 0.00010626 | 0.00111055 | 0.95449251 |
| Commd4 | 1417006_at -0.5478867 | 9.34630833 | -5.6251011 | 0.00046258 | 0.00325958 | -0.6513231 |
| Man2b2 | 1416584_at -0.5477229 | 8.0276638 | -4.2674035 | 0.0026162 | 0.01220426 | -2.5255075 |
| Suz12 | 1420021_s_a-0.5461545 | 9.27990003 | -5.9738925 | 0.00030887 | 0.00245646 | -0.2112975 |
| Il13ra1 | 1427165_at -0.5461299 | 9.37144667 | -6.5217705 | 0.00016892 | 0.001573 | 0.44756487 |
| Hars | 1438510_a_ -0.5460253 | 8.94400633 | -7.3340416 | 7.36E-05 | 0.00084578 | 1.35670164 |

| Itsn2 1435023_at -0.5455926 | 8.76529654 | -5.7202726 | 0.00041365 | 0.00300525 | -0.5296229 |
| --- | --- | --- | --- | --- | --- |
| Nap1l1 1429227_x_a-0.5454384 | 10.2774687 | -5.2583881 | 0.00071981 | 0.00451672 | -1.1319658 |
| H2-DMb2 1419744_at -0.5454169 | 8.33230034 | -3.307929 | 0.01044625 | 0.0354988 | -3.9961388 |
| Wrn 1425074_at -0.5451352 | 7.97385554 | -5.2476295 | 0.00072941 | 0.0045543 | -1.1463506 |
| Utp6 1424500_at -0.545123 | 8.98966648 | -6.4205805 | 0.00018832 | 0.00170106 | 0.32875915 |
| Hnrnpu 1423050_s_a-0.5450553 | 10.7088814 | -4.6746834 | 0.00151291 | 0.00797515 | -1.9360756 |
| Runx1 1422865_at -0.5449124 | 7.15200519 | -4.7604653 | 0.00135228 | 0.00733316 | -1.814878 |
| Gpm6b 1425942_a_ -0.5447676 | 8.37236009 | -5.6174518 | 0.00046678 | 0.00327594 | -0.6611585 |
| Tmed9 1439448_x_a-0.544566 | 9.15117007 | -5.6008906 | 0.00047601 | 0.00332226 | -0.6824802 |
| Tbc1d10b 1424236_at -0.5443615 | 9.81078202 | -4.2906624 | 0.00253397 | 0.01190881 | -2.4912342 |
| Sgpp1 1420821_at -0.5443499 | 7.50427953 | -5.9032399 | 0.00033478 | 0.00260406 | -0.2991081 |
| Zwint 1429787_x_a-0.5442588 | 8.65473831 | -7.660393 | 5.37E-05 | 0.00067791 | 1.70095313 |
| NA 1438655_a_ -0.5442562 | 12.5396831 | -8.4426949 | 2.63E-05 | 0.00042159 | 2.48162875 |
| Thumpd3 1417684_at -0.5436279 | 8.97350147 | -6.2335398 | 0.00023098 | 0.00197403 | 0.10575669 |
| Brca1 1424629_at -0.5434496 | 7.01260384 | -5.8142789 | 0.00037084 | 0.0027955 | -0.4106231 |
| Lpar1 1417143_at -0.5434155 | 8.45966633 | -6.5774355 | 0.0001592 | 0.00150695 | 0.5123778 |
| Gadd45g 1453851_a_ -0.543385 | 8.89262827 | -5.288983 | 0.00069325 | 0.00440487 | -1.0911474 |
| Ankrd13a 1460428_at -0.5433372 | 9.94610077 | -4.2555434 | 0.00265923 | 0.01234897 | -2.543012 |
| Prdm1 1420425_at -0.5432345 | 7.9827329 | -6.2448479 | 0.00022812 | 0.00195694 | 0.11936583 |
| Eif5 1454663_at -0.5432313 | 9.69604912 | -5.6068093 | 0.00047269 | 0.00330415 | -0.6748558 |
| Col5a1 1416740_at -0.5428383 | 9.14938307 | -5.9145097 | 0.00033049 | 0.0025801 | -0.2850568 |
| Slc35b3 1448937_at -0.5428359 | 8.28775691 | -6.3794523 | 0.0001969 | 0.00175299 | 0.28010423 |
| Rap1a 1424139_at -0.5427089 | 11.5465085 | -7.7691474 | 4.85E-05 | 0.00063065 | 1.81315891 |
| Mapre1 1450740_a_ -0.5426811 | 11.1020326 | -5.1227363 | 0.00085173 | 0.00511669 | -1.3145354 |
| Olr1 1419534_at -0.5420248 | 6.76712906 | -6.8086026 | 0.00012492 | 0.00125571 | 0.77748575 |
| Rnf26 1436824_x_a-0.5419847 | 8.53662454 | -5.7552736 | 0.00039711 | 0.00292737 | -0.485176 |
| Spon2 1417860_a_ -0.5419811 | 8.0960798 | -4.124611 | 0.00318841 | 0.01421874 | -2.7374951 |
| Eif2s1 1452662_a_ -0.5416566 | 10.4726535 | -7.8557729 | 4.47E-05 | 0.0005978 | 1.90165803 |
| Fam13b 1434620_s_a-0.5416193 | 8.98958161 | -4.8277708 | 0.00123921 | 0.00685127 | -1.7205134 |
| Rfc3 1423700_at -0.541398 | 8.02057456 | -7.0910661 | 9.36E-05 | 0.00101615 | 1.09279409 |
| Ugcg 1421269_at -0.5413074 | 7.59037321 | -8.1318962 | 3.47E-05 | 0.00050239 | 2.17869632 |
| Prkx 1451299_at -0.5412958 | 9.39956154 | -5.2117241 | 0.00076248 | 0.00470769 | -1.1944764 |
| Ywhab 1420880_a_ -0.5412142 | 12.3595349 | -7.3859976 | 6.99E-05 | 0.00081507 | 1.4122795 |
| Snap29 1423356_at -0.5409317 | 8.68473855 | -5.6521279 | 0.00044807 | 0.00318804 | -0.6166367 |
| Dnase1l3 1421056_at -0.5405403 | 7.13219211 | -3.9027695 | 0.00436106 | 0.01804385 | -3.0720323 |
| Sfpq 1438458_a_ -0.5404646 | 8.66374355 | -4.7345485 | 0.00139877 | 0.00752062 | -1.8513853 |
| Gtpbp4 1450873_at -0.5402666 | 9.94528122 | -6.9746202 | 0.00010535 | 0.00110355 | 0.96393592 |
| Timm10b 1448887_x_a-0.5402302 | 9.70334851 | -7.2309636 | 8.14E-05 | 0.00091222 | 1.24555342 |
| Prpf38b 1452869_at -0.5399179 | 8.85627254 | -3.8126121 | 0.00496304 | 0.01993133 | -3.209706 |
| Eif5 1454664_a_ -0.5397411 | 10.6916113 | -4.6596863 | 0.00154307 | 0.00810092 | -1.9573708 |
| Trim47 1426784_at -0.5390108 | 9.46089861 | -4.3194468 | 0.00243604 | 0.01156356 | -2.4489196 |
| Hmgcr 1427229_at -0.5386147 | 7.50857107 | -4.5463136 | 0.00179324 | 0.0091128 | -2.1193769 |
| Endod1 1426543_x_a-0.5385923 | 7.42490937 | -7.8268326 | 4.60E-05 | 0.00060838 | 1.87217732 |
| Clock 1418659_at -0.5383411 | 8.77019884 | -4.6357327 | 0.00159259 | 0.00830543 | -1.9914496 |
| Lats2 1439441_x_a-0.5383277 | 8.54087193 | -6.1068607 | 0.00026589 | 0.0021975 | -0.0478298 |
| Hnrnpr 1437526_x_a-0.5381517 | 10.2574321 | -7.3487154 | 7.25E-05 | 0.00083711 | 1.37242847 |
| Zbtb1 1424750_at -0.5380504 | 8.54405477 | -6.8803631 | 0.00011601 | 0.00118995 | 0.85847683 |
| Fam104a 1449258_at -0.537966 | 9.32584692 | -7.3039092 | 7.58E-05 | 0.00086459 | 1.32433224 |
| NA 1435791_x_a-0.5377181 | 13.1375969 | -8.3637805 | 2.82E-05 | 0.00044026 | 2.40558086 |
| Acsl4 1451828_a_ -0.5376561 | 7.35011527 | -5.4414482 | 0.00057591 | 0.00384111 | -0.8896925 |
| NA 1423206_s_a-0.5375909 | 10.8783504 | -8.8081459 | 1.92E-05 | 0.00033466 | 2.82635611 |
| NA 1423531_a_ -0.537221 | 9.1064601 | -7.4831815 | 6.37E-05 | 0.00076537 | 1.51544625 |
| Anxa7 1416137_at -0.5371682 | 9.34651029 | -6.5114397 | 0.0001708 | 0.00158698 | 0.43549417 |
| Dnpep 1438557_x_a-0.5370396 | 11.5142564 | -6.5813741 | 0.00015853 | 0.00150317 | 0.51694925 |
| Batf3 1453076_at -0.5367614 | 8.13320437 | -4.7451801 | 0.00137949 | 0.00743482 | -1.8363978 |
| Cd34 1416072_at -0.5367251 | 10.5094857 | -3.0649519 | 0.01511554 | 0.04745042 | -4.3819976 |
| Dlgap5 1455730_at -0.5366814 | 7.5901442 | -5.4824716 | 0.00054818 | 0.00369417 | -0.8360417 |
| 1700123O20 1416917_at -0.5365609 | 9.18955949 | -6.1769187 | 0.00024592 | 0.00206893 | 0.03736611 |
| Chpf2 1452170_at -0.536221 | 8.58477276 | -7.8991345 | 4.30E-05 | 0.00058145 | 1.94566947 |
| Slc41a3 1425439_a_ -0.5360352 | 10.3612208 | -3.9213312 | 0.00424711 | 0.01769173 | -3.0438077 |
| Cbx4 1419583_at -0.5354486 | 9.32203292 | -4.5511464 | 0.00178172 | 0.00907052 | -2.1124343 |
| Pou3f1 1460038_at -0.5354252 | 7.29550684 | -8.7932804 | 1.95E-05 | 0.00033812 | 2.81256738 |
| Ccdc86 1452414_s_a-0.5353438 | 8.19127804 | -5.3472212 | 0.0006456 | 0.00418532 | -1.0138121 |
| Ythdf2 1460357_at -0.5352972 | 9.69936688 | -5.7375082 | 0.00040541 | 0.00296164 | -0.5077151 |
| Tor1aip1 1426084_a_ -0.5350978 | 9.00303324 | -4.3274348 | 0.0024096 | 0.01146681 | -2.4371967 |
| Rhoh 1429319_at -0.5350571 | 7.6259611 | -5.2447916 | 0.00073197 | 0.00456524 | -1.1501478 |
| Mtmr2 1425460_at -0.5348448 | 8.356042 | -6.5983989 | 0.00015569 | 0.00148496 | 0.53668729 |
| Resf1 1452397_at -0.534489 | 7.1998901 | -5.1258285 | 0.00084844 | 0.0051037 | -1.3103448 |
| Slc35a5 1419971_s_a-0.5342582 | 7.87749326 | -4.5007503 | 0.00190588 | 0.00954833 | -2.1849912 |
| Nub1 1452776_a_ -0.5342319 | 9.74707688 | -6.3830107 | 0.00019614 | 0.00174871 | 0.28432228 |
| Prm1 1439379_x_a-0.5340651 | 6.29688596 | -5.2691499 | 0.00071034 | 0.0044749 | -1.1175928 |
| Fas 1460251_at -0.5335933 | 9.04532267 | -7.8056799 | 4.69E-05 | 0.00061671 | 1.85057545 |
| Ap3b1 1450915_at -0.5334792 | 8.37915253 | -6.8162975 | 0.00012393 | 0.00124896 | 0.78619981 |
| 2310031A07 1430419_at -0.5332264 | 7.19285966 | -5.5413655 | 0.00051089 | 0.00350112 | -0.7594279 |
| Thoc1 1424641_a_ -0.5332259 | 7.86518189 | -5.7664979 | 0.00039196 | 0.00290297 | -0.4709579 |
| Tbcd 1452194_at -0.5330389 | 8.69855748 | -6.9047573 | 0.00011314 | 0.00116473 | 0.88587022 |
| Pycr1 1424556_at -0.5329261 | 7.76688339 | -7.0504452 | 9.76E-05 | 0.00104354 | 1.04802086 |
| Nadk 1416249_at -0.5328195 | 9.04475622 | -3.5333499 | 0.00746102 | 0.0273103 | -3.6418644 |
| Eif2s2 1448820_a_ -0.5327852 | 10.0971212 | -5.8271639 | 0.00036537 | 0.00276615 | -0.3944055 |
| Akip1 1450959_at -0.5325199 | 9.04245571 | -6.8051655 | 0.00012537 | 0.00125655 | 0.77359119 |
| Tor2a 1438547_x_a-0.5325073 | 7.19673429 | -7.8759016 | 4.39E-05 | 0.00059092 | 1.92211211 |
| Snx18 1416359_at -0.5317877 | 9.59574999 | -4.2080381 | 0.0028394 | 0.01298509 | -2.6133136 |
| Dctn5 1415748_a_ -0.5317473 | 9.84803884 | -7.2077861 | 8.33E-05 | 0.0009283 | 1.22039791 |
| Sipa1 1416206_at -0.5315476 | 9.10072078 | -3.6749822 | 0.00605935 | 0.02325947 | -3.4216581 |
| Etv6 1423401_at -0.531332 | 7.88310025 | -6.1474223 | 0.00025412 | 0.00212138 | 0.00157414 |

| Snw1 | 1429002_at -0.5312291 | 10.5144282 | -5.537575 | 0.00051321 | 0.00351168 | -0.7643445 |
| --- | --- | --- | --- | --- | --- | --- |
| Cnpy2 | 1437783_x_a-0.5311147 | 9.76551322 | -6.9967926 | 0.000103 | 0.00108346 | 0.98859259 |
| Rp2 | 1419586_at -0.5310796 | 6.79529809 | -4.0844201 | 0.00337277 | 0.01484253 | -2.7976426 |
| Rlim | 1439403_x_a-0.5310659 | 7.86196009 | -7.0665536 | 9.60E-05 | 0.00103264 | 1.06579854 |
| Stx2 | 1418164_at -0.5310326 | 9.00381814 | -6.4706467 | 0.00017843 | 0.0016398 | 0.387701 |
| 2510039O18 | 1438646_x_a-0.5305659 | 10.3319602 | -5.8120326 | 0.00037181 | 0.00279905 | -0.4134526 |
| Rhno1 | 1452313_at -0.5302734 | 8.85549765 | -6.5531663 | 0.00016336 | 0.00153482 | 0.48416736 |
| Capza2 | 1423058_at -0.5300701 | 11.2035514 | -7.5926899 | 5.73E-05 | 0.00070819 | 1.63047526 |
| Eftud2 | 1416557_a_ -0.5299864 | 8.99173845 | -5.9782469 | 0.00030735 | 0.00244778 | -0.2059074 |
| Atox1 | 1460639_a_ -0.5299306 | 11.0967683 | -4.8623455 | 0.00118515 | 0.00661691 | -1.6722878 |
| Myadm | 1439389_s_a-0.5295654 | 12.0966608 | -8.3623224 | 2.83E-05 | 0.00044036 | 2.40417022 |
| Tax1bp1 | 1448399_at -0.5293213 | 10.3525075 | -8.1269859 | 3.49E-05 | 0.00050366 | 2.17383579 |
| Olfm1 | 1425784_a_ -0.5289699 | 8.96900596 | -2.8120478 | 0.0223432 | 0.06416399 | -4.7859386 |
| Csnk1d | 1437690_x_a-0.5288379 | 10.5087401 | -7.5441623 | 6.00E-05 | 0.00073147 | 1.57965959 |
| Aurka | 1424511_at -0.5287333 | 7.71573702 | -5.8739314 | 0.00034621 | 0.00266111 | -0.3357298 |
| Scamp2 | 1448404_at -0.5286198 | 9.10510227 | -5.7944794 | 0.00037944 | 0.00283673 | -0.4355872 |
| Virma | 1427906_at -0.5285355 | 8.50282536 | -5.7102443 | 0.00041853 | 0.00303207 | -0.5423881 |
| Morf4l2 | 1456743_x_a-0.5284626 | 11.6963693 | -8.3531012 | 2.85E-05 | 0.00044215 | 2.39524456 |
| Ccr2 | 1421188_at -0.5283964 | 8.34453195 | -4.7579411 | 0.00135673 | 0.00734883 | -1.8184296 |
| Slc6a6 | 1421346_a_ -0.5281693 | 9.88954013 | -4.5077206 | 0.00188816 | 0.00947693 | -2.1749349 |
| Syk | 1418262_at -0.528161 | 8.23755833 | -4.4153573 | 0.00213814 | 0.01044219 | -2.3087382 |
| Tmem33 | 1425568_a_ -0.5281577 | 9.80203932 | -4.4834464 | 0.00195064 | 0.00972321 | -2.2099858 |
| Stab1 | 1450199_a_ -0.5280953 | 9.34318865 | -3.7752126 | 0.00523828 | 0.02075371 | -3.2670931 |
| Adam19 | 1418403_at -0.5279365 | 7.6214546 | -3.5741944 | 0.00702452 | 0.02598409 | -3.5781553 |
| Slc8b1 | 1417811_at -0.5279357 | 8.75570697 | -4.0137229 | 0.00372543 | 0.01600947 | -2.9039446 |
| Klf10 | 1416029_at -0.5278155 | 8.32288203 | -2.9092548 | 0.01921438 | 0.05705724 | -4.6305643 |
| Arrdc4 | 1424759_at -0.5276992 | 7.87719293 | -5.994712 | 0.00030166 | 0.00241522 | -0.1855486 |
| Syngr2 | 1417081_a_ -0.5275853 | 11.2609275 | -6.1403154 | 0.00025614 | 0.00213353 | -0.0070666 |
| Erh | 1430536_a_ -0.5273739 | 9.58735171 | -8.1580525 | 3.39E-05 | 0.00049572 | 2.20454813 |
| Ttc13 | 1438631_x_a-0.5271886 | 8.47473367 | -6.653901 | 0.00014683 | 0.00141767 | 0.6007883 |
| Dok3 | 1418096_at -0.5268145 | 8.51451674 | -5.997913 | 0.00030057 | 0.00241023 | -0.1815947 |
| Pdlim7 | 1428319_at -0.5266981 | 8.51695156 | -3.8565222 | 0.00465946 | 0.01899988 | -3.1425342 |
| Enoph1 | 1437327_x_a-0.5266762 | 8.36103064 | -8.1728341 | 3.35E-05 | 0.0004933 | 2.21912811 |
| Psma6 | 1416506_at -0.5266573 | 11.1837199 | -7.8903093 | 4.33E-05 | 0.00058517 | 1.93672745 |
| Vars | 1448472_at -0.5264761 | 9.65815187 | -4.9796557 | 0.00102003 | 0.00589671 | -1.5099245 |
| Psma6 | 1437144_x_a-0.5264291 | 11.9331085 | -7.60062 | 5.69E-05 | 0.00070363 | 1.6387554 |
| Smc6 | 1422910_s_a-0.5264257 | 9.12830546 | -3.4264637 | 0.00874495 | 0.03089738 | -3.8093163 |
| Bud31 | 1439466_s_a-0.5263975 | 10.2933924 | -5.3296386 | 0.0006596 | 0.00425415 | -1.0371102 |
| Blvra | 1428580_at -0.5263918 | 9.36750014 | -5.8686809 | 0.00034831 | 0.00267358 | -0.3423026 |
| Celf2 | 1451154_a_ -0.5263496 | 10.4444509 | -5.6321785 | 0.00045873 | 0.00324152 | -0.6422302 |
| Slc15a4 | 1448385_at -0.5262457 | 8.55009455 | -3.6338579 | 0.00643496 | 0.02428631 | -3.4853878 |
| Wnk1 | 1436746_at -0.5262157 | 10.6204416 | -3.2552766 | 0.01131069 | 0.03786359 | -4.0794575 |
| Ehmt2 | 1460692_at -0.526212 | 9.56403248 | -3.1029373 | 0.01426118 | 0.0453071 | -4.3214724 |
| Tmem168 | 1423822_a_ -0.5261058 | 9.13224308 | -5.331302 | 0.00065826 | 0.00424797 | -1.0349042 |
| Tnpo1 | 1433585_at -0.5260347 | 10.0366497 | -5.6670557 | 0.00044027 | 0.00314438 | -0.5975211 |
| 9530068E07 | 1427108_at -0.5259431 | 10.8328976 | -4.6722833 | 0.0015177 | 0.00799362 | -1.9394815 |
| Ptrhd1 | 1455207_at -0.5259234 | 8.488215 | -6.5887946 | 0.00015729 | 0.00149601 | 0.52555675 |
| Ugcg | 1421268_at -0.5258239 | 8.55331451 | -4.3607106 | 0.00230272 | 0.01106729 | -2.3884553 |
| Mfap3 | 1424721_at -0.5255435 | 9.48241908 | -5.3676548 | 0.00062974 | 0.00410934 | -0.9867909 |
| Chmp1b | 1418816_at -0.5253224 | 8.93389592 | -6.6606872 | 0.00014578 | 0.00140993 | 0.60860004 |
| Ebna1bp2 | 1428315_at -0.5250465 | 8.93281318 | -6.2458381 | 0.00022787 | 0.00195629 | 0.12055671 |
| NA | 1430019_a_ -0.5248072 | 10.4092346 | -5.1276738 | 0.00084649 | 0.00509736 | -1.3078446 |
| Ywhab | 1455815_a_ -0.5247424 | 12.1834573 | -5.7603725 | 0.00039476 | 0.00291669 | -0.4787151 |
| Fbxo4 | 1427121_at -0.5245452 | 8.41019282 | -4.8940835 | 0.00113779 | 0.00640539 | -1.6281682 |
| Tm9sf3 | 1460186_at -0.5245103 | 10.3773288 | -5.036294 | 0.00094943 | 0.00558662 | -1.4322329 |
| Sntb2 | 1420372_at -0.5244486 | 8.2803008 | -7.1404915 | 8.91E-05 | 0.00097968 | 1.1470171 |
| Fam167b | 1455587_at -0.5244231 | 7.53713422 | -8.1341824 | 3.47E-05 | 0.00050209 | 2.18095856 |
| NA | 1433685_a_ -0.5240834 | 9.20826556 | -3.9866929 | 0.00387055 | 0.01644621 | -2.9447536 |
| Rcc2 | 1452226_at -0.5238388 | 7.96430125 | -7.246629 | 8.02E-05 | 0.00090256 | 1.2625216 |
| Pnrc2 | 1416187_s_a-0.5237681 | 9.58673195 | -6.6708693 | 0.00014423 | 0.00140093 | 0.62031044 |
| Galnt7 | 1426908_at -0.5232622 | 8.3313131 | -4.920268 | 0.00110026 | 0.00624277 | -1.5918761 |
| Clec1b | 1421182_at -0.5230102 | 7.29407566 | -4.5360385 | 0.001818 | 0.00921179 | -2.1341485 |
| Ncaph | 1423920_at -0.5230047 | 7.54257216 | -5.8672598 | 0.00034888 | 0.00267524 | -0.3440822 |
| Gjc1 | 1449094_at -0.5229997 | 8.70453213 | -7.1872816 | 8.51E-05 | 0.0009437 | 1.19809315 |
| Snhg6 | 1436506_a_ -0.522856 | 9.83543323 | -5.7771548 | 0.00038714 | 0.00287818 | -0.4574742 |
| Rbm14 | 1456566_x_a-0.5228029 | 8.25186583 | -8.099344 | 3.58E-05 | 0.00051312 | 2.1464298 |
| Nup160 | 1418530_at -0.5226642 | 8.04362882 | -6.3951285 | 0.00019358 | 0.00173487 | 0.29867441 |
| Stk4 | 1421107_at -0.5226208 | 7.18721421 | -5.8779755 | 0.00034461 | 0.00265598 | -0.3306697 |
| Ctnnbl1 | 1448582_at -0.5225166 | 8.68356816 | -5.7025981 | 0.00042229 | 0.00305152 | -0.5521304 |
| Ccm2 | 1434649_at -0.5224605 | 8.48423612 | -4.545204 | 0.0017959 | 0.00912221 | -2.1209713 |
| Mafb | 1451715_at -0.5221697 | 7.45385805 | -3.7071959 | 0.00578144 | 0.02246634 | -3.371862 |
| Eif2s2 | 1456617_a_ -0.5220837 | 11.291997 | -6.2032057 | 0.00023886 | 0.00202211 | 0.06916882 |
| H2afj | 1424771_at -0.5219311 | 9.59886906 | -5.9292057 | 0.00032499 | 0.00255106 | -0.2667592 |
| Atxn7l3 | 1435019_at -0.5219127 | 9.49702032 | -4.3290469 | 0.0024043 | 0.0114464 | -2.4348319 |
| Anp32e | 1420592_a_ -0.5213803 | 9.33617541 | -7.3328608 | 7.37E-05 | 0.00084578 | 1.35543506 |
| Zfp281 | 1426377_at -0.5213768 | 8.33945997 | -7.4225214 | 6.75E-05 | 0.00079727 | 1.4511723 |
| Dnpep | 1439452_x_a-0.521281 | 11.4834709 | -6.5834734 | 0.00015818 | 0.00150108 | 0.51938502 |
| Tcea1 | 1419258_at -0.5211937 | 10.5437379 | -7.4495368 | 6.58E-05 | 0.00078196 | 1.47984648 |
| Ube2d3 | 1455479_a_ -0.5211868 | 12.1859691 | -7.041095 | 9.85E-05 | 0.00105153 | 1.03768809 |
| Git2 | 1423391_at -0.5211552 | 9.74827255 | -5.3944954 | 0.00060955 | 0.00400665 | -0.9513857 |
| Glod4 | 1428421_a_ -0.5210769 | 10.1834408 | -7.3854173 | 7.00E-05 | 0.00081511 | 1.41166047 |
| Matr3 | 1434888_a_ -0.5210386 | 10.3401038 | -5.4772199 | 0.00055165 | 0.00371341 | -0.8428968 |
| Zfp277 | 1433953_at -0.5208891 | 8.92649705 | -6.9921535 | 0.00010348 | 0.00108707 | 0.98343844 |
| NA | 1455319_x_a-0.5208689 | 12.0708406 | -6.2068214 | 0.0002379 | 0.00201739 | 0.07353614 |
| NA | 1436944_x_a-0.520719 | 8.99390266 | -4.4955064 | 0.00191932 | 0.00959662 | -2.1925613 |

| D16Ertd472e | 1424724_a_ | -0.5206746 | 7.48708869 | -5.1735451 | 0.00079946 | 0.00488381 | -1.2458491 |
| --- | --- | --- | --- | --- | --- | --- | --- |
| Ubap2l | 1454643_at | -0.520537 | 9.37050961 | -5.7458331 | 0.0004015 | 0.00294614 | -0.497148 |
| Med10 | 1448295_at | -0.5202025 | 10.2735149 | -3.6153948 | 0.00661158 | 0.02477158 | -3.5140568 |
| Usp12 | 1434483_at | -0.5200031 | 8.57732863 | -7.6288332 | 5.54E-05 | 0.0006895 | 1.66816009 |
| Fbxo33 | 1426871_at | -0.5199565 | 8.43760096 | -5.5083654 | 0.00053143 | 0.00361783 | -0.8022978 |
| Csf1 | 1425155_x_a-0.5196554 | | 8.00016348 | -5.6440279 | 0.00045236 | 0.00321054 | -0.6270218 |
| Snw1 | 1429003_at -0.5196375 | | 8.73434612 | -4.9325982 | 0.00108305 | 0.00617449 | -1.5748199 |
| Rp2 | 1419587_s_a-0.5194102 | | 7.07839297 | -3.5583599 | 0.00719044 | 0.02651138 | -3.6028347 |
| B4galt1 | 1418014_a_ -0.5192307 | | 11.2028932 | -4.8003276 | 0.00128402 | 0.007039 | -1.7589122 |
| Odc1 | 1437711_x_a-0.5189706 | | 10.7493143 | -4.5173275 | 0.00186404 | 0.0093979 | -2.1610854 |
| NA | 1460680_a_ -0.5188245 | | 13.0650311 | -8.0637601 | 3.70E-05 | 0.00052371 | 2.1110392 |
| Fem1b | 1418324_at -0.5187704 | | 9.57315084 | -7.6785875 | 5.28E-05 | 0.00066987 | 1.71981105 |
| Fech | 1418698_a_ -0.5186741 | | 10.3029921 | -1.7440138 | 0.11859824 | 0.21950864 | -6.4340885 |
| Rps23 | 1460175_at -0.5185684 | | 13.3728743 | -7.0614897 | 9.65E-05 | 0.00103562 | 1.06021316 |
| Mknk1 | 1417630_at -0.5185268 | | 8.85572757 | -5.2263542 | 0.00074881 | 0.00463717 | -1.174845 |
| Mcm3 | 1426653_at -0.5184934 | | 8.14456084 | -6.0247757 | 0.00029158 | 0.00235527 | -0.1484678 |
| Tox4 | 1416574_at -0.518401 | | 9.39584573 | -5.7697289 | 0.00039049 | 0.00289457 | -0.4668683 |
| Vps37c | 1437615_s_a-0.5183589 | | 9.08286511 | -6.9278047 | 0.0001105 | 0.00114276 | 0.91168679 |
| Ppih | 1424136_a_ -0.5182558 | | 7.69457076 | -5.7680088 | 0.00039127 | 0.00289941 | -0.4690453 |
| Spred1 | 1423162_s_a-0.5181836 | | 7.6095489 | -6.620149 | 0.00015215 | 0.00145976 | 0.561852 |
| Elk4 | 1427162_a_ -0.5178995 | | 10.0848094 | -6.9773163 | 0.00010506 | 0.00110104 | 0.96693713 |
| NA | 1456319_at -0.517837 | | 7.81890348 | -3.1019738 | 0.01428222 | 0.04533893 | -4.3230069 |
| Crbn | 1423094_at -0.5176878 | | 8.59130632 | -4.3429176 | 0.00235922 | 0.01127356 | -2.4144993 |
| Psen1 | 1450399_at -0.5175561 | | 10.0693191 | -6.3387322 | 0.00020582 | 0.00181431 | 0.23172159 |
| Pold1 | 1456055_x_a-0.5174688 | | 8.07754142 | -8.9181122 | 1.75E-05 | 0.00031322 | 2.92775612 |
| Gstp3 | 1424953_at -0.5174596 | | 7.81869978 | -7.8119368 | 4.66E-05 | 0.00061438 | 1.85696999 |
| Clint1 | 1452152_at -0.5172452 | | 10.5216708 | -6.2628411 | 0.00022365 | 0.00193098 | 0.14098653 |
| Il18rap | 1421291_at -0.5171924 | | 7.91276209 | -8.7700399 | 1.99E-05 | 0.00034276 | 2.79097119 |
| Leprot | 1424438_a_ -0.5170073 | | 9.6526363 | -3.8237897 | 0.00488381 | 0.01969374 | -3.1925857 |
| Notch1 | 1418634_at -0.5166429 | | 9.75901618 | -4.4233654 | 0.00211511 | 0.01036093 | -2.2970905 |
| Trnt1 | 1425562_s_a-0.5165031 | | 8.55737674 | -5.4087962 | 0.00059908 | 0.00395553 | -0.9325627 |
| Plcl2 | 1426450_at -0.5164996 | | 9.08698407 | -5.2349963 | 0.00074087 | 0.0046005 | -1.1632628 |
| Smarcd2 | 1448401_at -0.5161347 | | 9.34640732 | -3.6312275 | 0.00645982 | 0.02435467 | -3.4894701 |
| Ewsr1 | 1436884_x_a-0.515955 | | 9.84763364 | -4.0803948 | 0.00339185 | 0.01489947 | -2.8036781 |
| Cdk6 | 1460291_at -0.5158779 | | 7.81726481 | -6.6767942 | 0.00014333 | 0.0013958 | 0.62711884 |
| Cdc7 | 1426002_a_ -0.5157769 | | 7.13810636 | -7.0358242 | 9.90E-05 | 0.00105516 | 1.03185894 |
| NA | 1419426_s_a-0.5157739 | | 9.41568972 | -6.6019075 | 0.00015512 | 0.00148132 | 0.54075067 |
| Atf5 | 1425927_a_ -0.5157288 | | 9.97672087 | -4.1066653 | 0.00326935 | 0.01448859 | -2.7643259 |
| Ywhaq | 1436846_x_a-0.515546 | | 9.05775429 | -7.7947005 | 4.74E-05 | 0.00062002 | 1.83934481 |
| Padi2 | 1418252_at -0.5154637 | | 8.10773848 | -4.3407581 | 0.00236617 | 0.01130046 | -2.4176631 |
| Sec24c | 1452147_at -0.5154483 | | 10.5043046 | -6.1934254 | 0.00024146 | 0.0020382 | 0.05734683 |
| Dnajc2 | 1448794_s_a-0.5153288 | | 8.80659025 | -5.5890622 | 0.00048273 | 0.00335394 | -0.6977319 |
| Ubxn4 | 1426485_at -0.515309 | | 10.178927 | -5.7805667 | 0.00038561 | 0.00287245 | -0.4531607 |
| Ccl11 | 1417789_at -0.5152112 | | 7.37008931 | -5.4149762 | 0.00059462 | 0.00393465 | -0.9244374 |
| Secisbp2l | 1450642_at -0.5151935 | | 9.43435444 | -4.8335798 | 0.00122994 | 0.00681272 | -1.712399 |
| Plscr1 | 1429527_a_ -0.5151597 | | 8.12246483 | -5.4456247 | 0.00057302 | 0.00382632 | -0.8842198 |
| Ipo4 | 1436420_a_ -0.5149456 | | 10.0217961 | -6.0738322 | 0.00027591 | 0.00225764 | -0.0882171 |
| Yipf1 | 1424196_at -0.5149235 | | 9.63784716 | -5.6817184 | 0.00043275 | 0.00310823 | -0.5787747 |
| Dnajc1 | 1420500_at -0.5148813 | | 9.04340879 | -5.2844645 | 0.0006971 | 0.00441946 | -1.0971675 |
| Tspan33 | 1451608_a_ -0.5148178 | | 7.6249182 | -6.202572 | 0.00023902 | 0.00202211 | 0.06840311 |
| Ckap4 | 1452181_at -0.5147638 | | 8.78511355 | -5.0245209 | 0.00096366 | 0.00564996 | -1.4483449 |
| Atox1 | 1415760_s_a-0.5146117 | | 10.7903516 | -4.6793906 | 0.00150358 | 0.00793955 | -1.929398 |
| Ctdsp2 | 1451075_s_a-0.5145549 | | 10.9979413 | -6.182532 | 0.00024439 | 0.00205684 | 0.04416479 |
| Rpl13a | 1455485_x_a-0.5144575 | | 13.2864053 | -7.7196102 | 5.08E-05 | 0.00065172 | 1.76220246 |
| Mthfr | 1434087_at -0.5141321 | | 8.82622598 | -7.4091724 | 6.84E-05 | 0.0008035 | 1.43697437 |
| Psenen | 1415679_at -0.5140807 | | 11.5709887 | -6.8176722 | 0.00012375 | 0.00124798 | 0.78775572 |
| Hnrnpab | 1453849_s_a-0.5135781 | | 10.4550524 | -6.8654827 | 0.00011779 | 0.00120222 | 0.8417325 |
| Aprt | 1423801_a_ -0.5134763 | | 10.2764209 | -7.3365715 | 7.34E-05 | 0.00084498 | 1.35941474 |
| Txndc9 | 1436951_x_a-0.5134425 | | 10.7275015 | -7.8163301 | 4.64E-05 | 0.00061223 | 1.86145755 |
| Mtf2 | 1418516_at -0.5134197 | | 8.59249781 | -7.8205705 | 4.62E-05 | 0.00061123 | 1.86578705 |
| Rps6ka4 | 1448498_at -0.5131786 | | 9.57520705 | -4.7082508 | 0.00144771 | 0.00771276 | -1.8885259 |
| Dhx15 | 1416144_a_ -0.5131116 | | 10.8537775 | -5.9389913 | 0.00032138 | 0.00252673 | -0.2545913 |
| Eif3c | 1415859_at -0.5129009 | | 10.1180712 | -4.4761789 | 0.00196978 | 0.00980138 | -2.2204954 |
| Ssrp1 | 1426788_a_ -0.5127803 | | 10.1060121 | -6.0766556 | 0.00027504 | 0.00225131 | -0.084759 |
| Haus4 | 1451103_at -0.5127212 | | 7.47817007 | -6.0772474 | 0.00027486 | 0.00225063 | -0.0840344 |
| NA | 1430020_x_a-0.5125848 | | 9.39276182 | -6.1742717 | 0.00024664 | 0.00207348 | 0.03415881 |
| Coro1c | 1417752_at -0.5121019 | | 7.69791524 | -3.8221518 | 0.00489533 | 0.01971956 | -3.1950935 |
| Brix1 | 1451144_at -0.512097 | | 9.14074608 | -8.3286892 | 2.91E-05 | 0.0004489 | 2.37157621 |
| Atg3 | 1448993_at -0.5115635 | | 10.3373784 | -4.1043593 | 0.00327991 | 0.01452679 | -2.7677766 |
| Mkl1 | 1434900_at -0.5115393 | | 8.34286536 | -3.0904098 | 0.01453723 | 0.04595289 | -4.3414268 |
| Kctd17 | 1435525_at -0.511338 | | 9.06862266 | -3.7003122 | 0.00583967 | 0.022619 | -3.3824936 |
| Vta1 | 1415722_a_ -0.5113008 | | 10.1764301 | -6.2357533 | 0.00023042 | 0.00197293 | 0.10842193 |
| Trp53bp1 | 1426956_a_ -0.5112875 | | 8.88819847 | -6.322686 | 0.00020945 | 0.00183613 | 0.21259804 |
| Rps18 | 1455572_x_a-0.5111352 | | 12.8525122 | -7.9083374 | 4.26E-05 | 0.00057999 | 1.95498574 |
| Uck2 | 1426909_at -0.5109431 | | 9.53743325 | -5.6767235 | 0.0004353 | 0.00311573 | -0.5851575 |
| Pola2 | 1448369_at -0.5106358 | | 8.81692869 | -5.7982518 | 0.00037778 | 0.00282996 | -0.4308267 |
| Zfp622 | 1438000_x_a-0.5103578 | | 10.8020053 | -8.082316 | 3.63E-05 | 0.00051754 | 2.12950982 |
| Ap2b1 | 1452292_at -0.5100721 | | 9.80563407 | -3.8207937 | 0.00490491 | 0.0197467 | -3.1971731 |
| Rell1 | 1427243_at -0.5099939 | | 8.50517881 | -7.0021846 | 0.00010244 | 0.00107991 | 0.9945801 |
| Stk38 | 1416252_at -0.5099236 | | 9.70561058 | -3.8880469 | 0.00445377 | 0.01834382 | -3.0944486 |
| Mbnl1 | 1416904_at -0.5096143 | | 12.1814397 | -6.172626 | 0.00024709 | 0.00207573 | 0.0321643 |
| Nab1 | 1448781_at -0.509554 | | 9.07196361 | -5.4305329 | 0.00058355 | 0.00387651 | -0.9040071 |
| Sdc3 | 1436482_a_ -0.50925 | | 7.84307192 | -4.746072 | 0.00137788 | 0.0074297 | -1.8351412 |
| Plekha2 | 1417289_at -0.509011 | | 8.7734171 | -5.1372635 | 0.00083641 | 0.00505412 | -1.2948594 |
| Hk1 | 1437974_a_ -0.5089945 | | 11.479407 | -4.2581012 | 0.00264989 | 0.01232341 | -2.5392353 |

| Vps35l | 1426694_at | -0.5088903 | 9.05134295 | -5.3315335 | 0.00065807 | 0.00424797 | -1.0345973 |
| --- | --- | --- | --- | --- | --- | --- | --- |
| Myo7a | 1421385_a_ | -0.5088692 | 8.44477143 | -4.7798042 | 0.00131868 | 0.00718933 | -1.7876986 |
| Eef1b2 | 1448252_a_ | -0.5087084 | 11.7543383 | -7.7638486 | 4.87E-05 | 0.0006327 | 1.8077204 |
| NA | 1448697_s_a-0.5086211 | | 11.6002391 | -7.6078946 | 5.65E-05 | 0.00069915 | 1.64634531 |
| Eid1 | 1448406_at -0.5085711 | | 9.34867847 | -3.6722563 | 0.00608352 | 0.02333192 | -3.425877 |
| Gnl3 | 1433656_a_ -0.5085359 | | 9.2023929 | -6.7603498 | 0.00013134 | 0.00130136 | 0.72268142 |
| Carhsp1 | 1415975_at -0.5081962 | | 9.48965034 | -4.5155847 | 0.00186839 | 0.00941037 | -2.1635969 |
| Eif3f | 1452285_a_ -0.5080677 | | 12.424193 | -7.0973593 | 9.31E-05 | 0.00101162 | 1.09971355 |
| Gch1 | 1420499_at -0.5079828 | | 9.07673716 | -6.1242306 | 0.00026077 | 0.00216263 | -0.0266471 |
| Ap1g2 | 1419113_at -0.5077572 | | 8.27819164 | -7.2563859 | 7.94E-05 | 0.00089612 | 1.27307606 |
| Cdca4 | 1423683_at -0.5076969 | | 8.97309399 | -5.1873545 | 0.00078586 | 0.00481405 | -1.2272438 |
| Abcc3 | 1428988_at -0.5076845 | | 8.18032127 | -7.0650151 | 9.61E-05 | 0.00103326 | 1.06410187 |
| Rnpc3 | 1424589_s_a-0.5075308 | | 7.15235859 | -4.8450682 | 0.00121184 | 0.00673606 | -1.6963654 |
| Adamts1 | 1450716_at -0.5074581 | | 9.06285195 | -5.1956598 | 0.00077781 | 0.00478149 | -1.2160671 |
| Napg | 1424069_at -0.5071656 | | 9.34876149 | -5.6731318 | 0.00043714 | 0.00312496 | -0.5897491 |
| Celf2 | 1423895_a_ -0.5069401 | | 10.4757027 | -6.4776561 | 0.0001771 | 0.00163345 | 0.39592805 |
| Mtmr6 | 1425485_at -0.5068715 | | 8.99319425 | -4.2079703 | 0.00283967 | 0.01298509 | -2.6134141 |
| Eif3c | 1415858_at -0.5067205 | | 11.3419772 | -5.6204504 | 0.00046512 | 0.0032719 | -0.657302 |
| Sin3b | 1434637_x_a-0.5067117 | | 10.3757155 | -6.4439658 | 0.00018363 | 0.001674 | 0.3563293 |
| Spin1 | 1436809_a_ -0.5065533 | | 10.2576145 | -6.6603141 | 0.00014584 | 0.00140993 | 0.60817072 |
| Rabif | 1419927_s_a-0.5061831 | | 8.85057988 | -8.101711 | 3.57E-05 | 0.00051238 | 2.14877956 |
| Gas5 | 1436222_at -0.5060379 | | 7.62106759 | -7.7182703 | 5.09E-05 | 0.0006518 | 1.76082055 |
| Xpo6 | 1422759_a_ -0.5059586 | | 8.6544718 | -5.5926249 | 0.00048069 | 0.00334467 | -0.693136 |
| NA | 1455348_x_a-0.5059438 | | 13.0507405 | -7.8850136 | 4.35E-05 | 0.00058735 | 1.93135793 |
| Fbl | 1416684_at -0.5059285 | | 9.50862478 | -7.1161046 | 9.13E-05 | 0.00099766 | 1.12029784 |
| D1Ertd622e | 1417471_s_a-0.5058597 | | 7.26535163 | -7.6178298 | 5.59E-05 | 0.00069384 | 1.65670197 |
| Fryl | 1427199_at -0.5054762 | | 9.0122717 | -4.5276366 | 0.00183852 | 0.00929293 | -2.1462381 |
| Pafah1b3 | 1416410_at -0.5052678 | | 7.58813764 | -4.7173281 | 0.00143061 | 0.00764498 | -1.875695 |
| Dnajc13 | 1434038_at -0.5052076 | | 8.89206367 | -4.6527702 | 0.00155719 | 0.00815432 | -1.9672021 |
| Il1b | 1449399_a_ -0.5050568 | | 7.95117307 | -7.1987529 | 8.41E-05 | 0.00093621 | 1.21057751 |
| Eif3a | 1416661_at -0.5048754 | | 7.68587738 | -4.2111714 | 0.00282712 | 0.01294337 | -2.6086675 |
| Ss18 | 1419360_a_ -0.5046398 | | 10.3422831 | -6.015366 | 0.0002947 | 0.00237452 | -0.1600609 |
| Rfc3 | 1432538_a_ -0.5044868 | | 7.20974657 | -5.7473486 | 0.00040079 | 0.00294303 | -0.4952252 |
| Pold4 | 1427885_at -0.5042927 | | 8.77702864 | -3.8479836 | 0.0047169 | 0.01919412 | -3.1555786 |
| Gmfb | 1417069_a_ -0.5039966 | | 7.52658739 | -4.6959234 | 0.0014713 | 0.00780541 | -1.9059697 |
| Sumo1 | 1438289_a_ -0.5035328 | | 10.8240622 | -5.1667024 | 0.00080629 | 0.00491133 | -1.2550781 |
| Tnfsf9 | 1422924_at -0.5034861 | | 7.71699018 | -7.7061983 | 5.15E-05 | 0.00065667 | 1.74836242 |
| Prkd2 | 1437509_x_a-0.5033707 | | 7.45860715 | -7.2099488 | 8.32E-05 | 0.00092676 | 1.22274771 |
| Tbca | 1417652_a_ -0.5032177 | | 11.3171168 | -5.8110529 | 0.00037223 | 0.00280129 | -0.4146869 |
| NA | 1435873_a_ -0.5032031 | | 13.6659878 | -7.6460099 | 5.45E-05 | 0.00068246 | 1.68602107 |
| Med15 | 1448435_at -0.5030599 | | 9.04554554 | -3.1955297 | 0.01238334 | 0.04063898 | -4.1742204 |
| Leprot | 1451350_a_ -0.5026766 | | 8.59194387 | -5.2688763 | 0.00071058 | 0.0044749 | -1.117958 |
| Gatad2a | 1423992_at -0.5026699 | | 10.0982928 | -3.8401668 | 0.00477015 | 0.01933107 | -3.1675277 |
| Ssr1 | 1417764_at -0.5024022 | | 9.15633439 | -5.1774346 | 0.0007956 | 0.00486714 | -1.2406061 |
| Trir | 1428201_at -0.502369 | | 10.1982625 | -5.7456092 | 0.0004016 | 0.00294614 | -0.497432 |
| Gmpr2 | 1416356_at -0.5020426 | | 8.61127241 | -4.7498281 | 0.00137115 | 0.00740396 | -1.8298505 |
| Diaph2 | 1427564_at -0.5018421 | | 8.09958943 | -4.9744428 | 0.00102681 | 0.00592685 | -1.517098 |
| Gdi2 | 1420815_at -0.5015058 | | 12.1841716 | -3.9621846 | 0.00400738 | 0.01691987 | -2.9818339 |
| Slu7 | 1447146_s_a-0.5014137 | | 9.9248082 | -5.2526054 | 0.00072495 | 0.0045352 | -1.1396955 |
| Eif3d | 1416100_at -0.500835 | | 10.9377603 | -4.618083 | 0.00163018 | 0.00845599 | -2.0166113 |
| Trp53 | 1426538_a_ -0.5007308 | | 8.21668644 | -4.8661893 | 0.0011793 | 0.00659562 | -1.6669369 |
| Rbck1 | 1415792_at -0.5006449 | | 9.9081931 | -4.2346109 | 0.00273705 | 0.01263042 | -2.5739523 |
| NA | 1437546_at -0.5006189 | | 8.80461543 | -5.0111261 | 0.00098012 | 0.0057111 | -1.4667 |
| Atp6v1h | 1415826_at -0.5005005 | | 8.91206404 | -4.3414882 | 0.00236382 | 0.01129159 | -2.4165933 |
| Tnfrsf12a | 1418571_at -0.500416 | | 11.0216814 | -6.8224151 | 0.00012315 | 0.00124424 | 0.79312256 |
| Cd96 | 1419226_at -0.5002905 | | 8.06746234 | -5.1068265 | 0.00086885 | 0.00520166 | -1.3361184 |
| Uap1 | 1437490_x_a-0.5000241 | | 8.32151124 | -5.3401872 | 0.00065116 | 0.00421294 | -1.0231275 |
| Igfbp4 | 1421992_a_ 0.50001436 | | 9.49060521 | 2.82422389 | 0.02192407 | 0.06325155 | -4.7664765 |
| Ybx2 | 1420762_a_ 0.50015773 | | 8.79776883 | 5.69245853 | 0.00042734 | 0.0030772 | -0.565062 |
| Pex19 | 1448332_at 0.50057862 | | 9.49311456 | 4.67481763 | 0.00151265 | 0.00797515 | -1.9358851 |
| Klf9 | 1428289_at 0.50086145 | | 11.8599457 | 5.15580151 | 0.0008173 | 0.00495971 | -1.2697944 |
| Cdh13 | 1423551_at 0.5011094 | | 10.985102 | 4.71458453 | 0.00143576 | 0.00766165 | -1.8795718 |
| Aldh6a1 | 1448104_at 0.50152932 | | 10.9413492 | 5.56328931 | 0.00049773 | 0.00343268 | -0.7310303 |
| Perp | 1416271_at 0.50156245 | | 10.9687614 | 5.9637256 | 0.00031246 | 0.00247834 | -0.2238925 |
| Foxo3 | 1434831_a_ 0.50184255 | | 10.1729942 | 6.52243249 | 0.0001688 | 0.00157295 | 0.44833795 |
| Pfn2 | 1418210_at 0.50203701 | | 10.0923035 | 4.90685307 | 0.00111931 | 0.00632403 | -1.6104572 |
| Rbpms | 1425652_s_a0.50222071 | | 9.41025789 | 5.38590899 | 0.00061593 | 0.00403826 | -0.9627009 |
| Tgfbr3 | 1425620_at 0.50251848 | | 8.00991618 | 8.29328284 | 3.01E-05 | 0.00045839 | 2.33714786 |
| Ndrg4 | 1426615_s_a0.50289176 | | 12.7154013 | 8.21025621 | 3.24E-05 | 0.00048169 | 2.25594514 |
| Mybpc3 | 1418551_at 0.50323947 | | 13.7878758 | 3.90616749 | 0.00433996 | 0.01798277 | -3.0668623 |
| Rab40b | 1436566_at 0.50330379 | | 7.42511263 | 6.25948596 | 0.00022448 | 0.00193444 | 0.13695809 |
| Slc25a29 | 1423980_at 0.50332962 | | 8.2573503 | 8.71347813 | 2.08E-05 | 0.00035402 | 2.73821137 |
| Immp2l | 1419495_at 0.50449367 | | 9.13517756 | 6.57347215 | 0.00015987 | 0.00151205 | 0.50777579 |
| Wee1 | 1416774_at 0.50461651 | | 7.71220561 | 5.5833073 | 0.00048604 | 0.00337149 | -0.7051592 |
| Hhatl | 1424553_at 0.50471251 | | 10.7845308 | 4.93432607 | 0.00108066 | 0.00616397 | -1.5724316 |
| Scara5 | 1451204_at 0.50487099 | | 9.26198208 | 2.04973517 | 0.07387161 | 0.15713385 | -5.9838072 |
| NA | 1418328_at 0.505092 | | 11.9309166 | 4.19860811 | 0.0028767 | 0.01312272 | -2.6273043 |
| Slc25a23 | 1419045_at 0.50538521 | | 8.50199527 | 3.37420053 | 0.00945594 | 0.03291231 | -3.8915524 |
| Asb8 | 1424225_at 0.50601952 | | 9.86381988 | 4.65941451 | 0.00154362 | 0.00810195 | -1.957757 |
| Opa1 | 1418768_at 0.50616728 | | 8.71885035 | 5.46474534 | 0.00055998 | 0.00375694 | -0.8591956 |
| Hopx | 1428662_a_ 0.50675376 | | 13.1749454 | 7.63502974 | 5.50E-05 | 0.00068809 | 1.67460707 |
| Slmap | 1421215_a_ 0.50677603 | | 8.2110822 | 4.95475347 | 0.00105287 | 0.00603731 | -1.5442272 |
| Mrpl45 | 1423492_at 0.50707409 | | 10.2473962 | 5.47559572 | 0.00055273 | 0.00371817 | -0.8450177 |
| Tfpi | 1452432_at 0.5071186 | | 9.36697621 | 5.10196252 | 0.00087416 | 0.00522518 | -1.3427239 |
| Rab4a | 1418341_at 0.50736852 | | 8.26660779 | 4.15419951 | 0.00305961 | 0.01378802 | -2.6933483 |

| Ank3 | 1451628_a_ | 0.5074171 | 9.38183404 | 6.19963215 | 0.0002398 | 0.00202725 | 0.06485065 |
| --- | --- | --- | --- | --- | --- | --- | --- |
| Hspa8 | 1431182_at | 0.50782132 | 8.37942498 | 2.76514928 | 0.02403622 | 0.0679752 | -4.8608875 |
| Sdha | 1426688_at | 0.50800835 | 13.1008759 | 7.54569811 | 5.99E-05 | 0.00073079 | 1.58127164 |
| Tecr | 1429681_a_ | 0.50814542 | 12.9524514 | 7.46430977 | 6.48E-05 | 0.00077533 | 1.49549296 |
| Sesn1 | 1454699_at | 0.50833606 | 10.1411659 | 4.51675496 | 0.00186546 | 0.00939982 | -2.1619105 |
| Pex11a | 1419365_at | 0.50866098 | 8.62286553 | 5.69004287 | 0.00042855 | 0.00308299 | -0.5681449 |
| Suox | 1451339_at | 0.50929221 | 8.93165345 | 5.96244623 | 0.00031291 | 0.00247992 | -0.2254784 |
| Lrrc20 | 1438422_at | 0.50936319 | 8.64467477 | 4.69236412 | 0.00147818 | 0.00783644 | -1.9110103 |
| Letm1 | 1420826_at | 0.50954166 | 9.42445293 | 5.63574421 | 0.0004568 | 0.00323295 | -0.6376517 |
| Abhd10 | 1427959_at | 0.51000324 | 8.84439841 | 7.10633362 | 9.22E-05 | 0.00100453 | 1.1095733 |
| Me1 | 1416632_at | 0.51000921 | 10.3456426 | 4.78579671 | 0.00130846 | 0.00714363 | -1.7792873 |
| Mrpl28 | 1437622_x_a0.51002565 | | 11.1756128 | 8.08557849 | 3.62E-05 | 0.00051688 | 2.13275387 |
| Adrb3 | 1455918_at 0.51007471 | | 8.0707113 | 3.36110637 | 0.00964347 | 0.03340104 | -3.9121904 |
| Fitm1 | 1451488_at 0.51014092 | | 10.8938472 | 8.13324989 | 3.47E-05 | 0.00050209 | 2.18003593 |
| Nipsnap2 | 1419484_a_ 0.51019213 | | 12.3179138 | 8.35685611 | 2.84E-05 | 0.00044128 | 2.39888011 |
| Ghr | 1417962_s_a0.51086312 | | 10.8791736 | 7.35248145 | 7.23E-05 | 0.00083531 | 1.37646093 |
| Kif1c | 1424746_at 0.51093464 | | 11.1055034 | 7.79177823 | 4.75E-05 | 0.00062136 | 1.83635359 |
| Wfdc2 | 1424351_at 0.51124969 | | 7.62710047 | 2.57772413 | 0.03222649 | 0.08468085 | -5.1598034 |
| Atp1b1 | 1423890_x_a0.51129928 | | 12.9626381 | 6.10348463 | 0.00026689 | 0.0022013 | -0.0519515 |
| Tesc | 1418744_s_a0.51152379 | | 11.5663026 | 2.96227564 | 0.01770243 | 0.05359148 | -4.5458521 |
| Cul3 | 1450756_s_a0.51163444 | | 9.04935744 | 7.88726893 | 4.34E-05 | 0.00058647 | 1.93364504 |
| Mmab | 1435097_at 0.51184193 | | 8.17849075 | 5.31763426 | 0.00066934 | 0.00429386 | -1.0530416 |
| Narf | 1451678_at 0.51191428 | | 10.4251854 | 4.90417616 | 0.00112316 | 0.00634257 | -1.6141681 |
| Ank3 | 1425202_a_ 0.51211615 | | 8.89459699 | 4.79796638 | 0.00128796 | 0.00704866 | -1.7622211 |
| Hadh | 1455972_x_a0.51247188 | | 13.6361216 | 6.6783152 | 0.00014311 | 0.00139479 | 0.62886591 |
| Ndufaf1 | 1423711_at 0.51322851 | | 10.1086819 | 7.41546898 | 6.80E-05 | 0.00079991 | 1.44367384 |
| Igfbp4 | 1423757_x_a0.5132368 | | 10.1986703 | 3.25746756 | 0.01127325 | 0.037755 | -4.0759868 |
| Bckdhb | 1427153_at 0.51386611 | | 10.3060766 | 6.0546634 | 0.00028192 | 0.00229605 | -0.1117223 |
| Btbd1 | 1455286_at 0.51395325 | | 11.7070578 | 7.81775222 | 4.63E-05 | 0.00061212 | 1.86290979 |
| Tuba4a | 1417373_a_ 0.51409335 | | 13.8924536 | 5.05386549 | 0.00092862 | 0.00549281 | -1.4082224 |
| Ryr2 | 1450123_at 0.51417864 | | 12.4181536 | 5.87226119 | 0.00034688 | 0.00266532 | -0.3378202 |
| Coq3 | 1436351_at 0.51435056 | | 9.9928595 | 6.80334232 | 0.0001256 | 0.0012577 | 0.7715248 |
| E2f6 | 1448835_at 0.51450024 | | 10.4624427 | 5.89900329 | 0.0003364 | 0.00261158 | -0.3043948 |
| Nnt | 1416105_at 0.51451699 | | 12.6694693 | 8.16434212 | 3.37E-05 | 0.00049436 | 2.21075452 |
| Fhl1 | 1417872_at 0.51467723 | | 11.3954939 | 6.90760391 | 0.00011281 | 0.00116187 | 0.88906219 |
| Acadvl | 1424184_at 0.51492672 | | 13.1161226 | 7.44275105 | 6.62E-05 | 0.00078465 | 1.47265153 |
| Atp5d | 1423716_s_a0.51514681 | | 13.1434668 | 8.1701025 | 3.36E-05 | 0.00049387 | 2.21643535 |
| Emc9 | 1418474_at 0.51570251 | | 9.56362573 | 7.08229928 | 9.45E-05 | 0.00102172 | 1.08314711 |
| Slc9a3r2 | 1428954_at 0.51597254 | | 8.97589991 | 4.45873111 | 0.00201656 | 0.00997945 | -2.2457573 |
| Ndufb2 | 1416834_x_a0.5162013 | | 12.471737 | 7.19099133 | 8.47E-05 | 0.00094161 | 1.20213212 |
| Eno3 | 1417951_at 0.51656196 | | 13.5766934 | 8.41897366 | 2.69E-05 | 0.00042686 | 2.45883046 |
| Ptges2 | 1417591_at 0.51669927 | | 9.73758189 | 7.03992152 | 9.86E-05 | 0.00105228 | 1.03639055 |
| Efcab2 | 1449884_at 0.51689943 | | 8.43503926 | 4.49130189 | 0.00193018 | 0.00964028 | -2.1986338 |
| Metap1d | 1452464_a_ 0.5169624 | | 9.17785563 | 6.89008043 | 0.00011485 | 0.00117866 | 0.86939728 |
| Mul1 | 1434118_at 0.51710701 | | 8.91619433 | 2.97961794 | 0.01723512 | 0.05248489 | -4.5181558 |
| Fbln5 | 1416164_at 0.51755003 | | 8.64699472 | 2.71330336 | 0.02606198 | 0.07230941 | -4.943698 |
| Tufm | 1433952_at 0.51772621 | | 10.5062251 | 7.34620814 | 7.27E-05 | 0.00083874 | 1.36974294 |
| Pcolce2 | 1451527_at 0.51786252 | | 9.33054435 | 4.02507056 | 0.00366625 | 0.0158206 | -2.8868393 |
| Adamtsl4 | 1451932_a_ 0.51808625 | | 9.7618715 | 3.75885798 | 0.00536371 | 0.02114362 | -3.2922379 |
| Idh1 | 1419821_s_a0.51843699 | | 9.43787232 | 3.90295438 | 0.00435991 | 0.01804385 | -3.071751 |
| Adhfe1 | 1424392_at 0.51893934 | | 9.9135683 | 4.80338113 | 0.00127895 | 0.00701458 | -1.7546345 |
| Hibadh | 1435967_s_a0.51898254 | | 12.0431478 | 5.84259775 | 0.00035892 | 0.00272829 | -0.3750092 |
| Dnaja3 | 1420629_a_ 0.51923368 | | 9.45090631 | 5.77828397 | 0.00038663 | 0.00287818 | -0.4560465 |
| Hadha | 1452173_at 0.51932325 | | 13.1143 | 7.13387549 | 8.97E-05 | 0.00098475 | 1.13977499 |
| Thbd | 1448529_at 0.51945971 | | 9.82822023 | 3.71143965 | 0.00574586 | 0.02234718 | -3.3653104 |
| Ttll1 | 1436833_x_a0.51956346 | | 11.0127261 | 6.97006319 | 0.00010584 | 0.00110766 | 0.95886123 |
| Gatad1 | 1452085_at 0.52063515 | | 9.70234291 | 7.38421866 | 7.01E-05 | 0.00081565 | 1.41038149 |
| Lgals4 | 1451336_at 0.52064729 | | 9.11009643 | 3.7616064 | 0.00534241 | 0.02108163 | -3.2880102 |
| Iscu | 1452579_at 0.52130643 | | 11.7302843 | 7.24532066 | 8.03E-05 | 0.0009026 | 1.26110553 |
| Hdlbp | 1419869_s_a0.52168157 | | 11.2716492 | 5.68542533 | 0.00043087 | 0.00309678 | -0.57404 |
| Impad1 | 1437290_at 0.52237377 | | 9.37041645 | 5.50101861 | 0.00053613 | 0.00363665 | -0.8118624 |
| Cpe | 1415949_at 0.5226355 | | 11.7841169 | 4.19624369 | 0.00288614 | 0.01315255 | -2.6308141 |
| Cox8b | 1449218_at 0.52289476 | | 13.6786372 | 8.892451 | 1.79E-05 | 0.00031806 | 2.90418811 |
| Tmem141 | 1435259_s_a0.52311986 | | 9.37816734 | 8.32674601 | 2.92E-05 | 0.0004489 | 2.36968973 |
| Pdss1 | 1431893_a_ 0.52332902 | | 8.6007627 | 4.51327021 | 0.00187418 | 0.0094291 | -2.1669329 |
| Galm | 1452583_s_a0.52355159 | | 8.47916874 | 4.11076267 | 0.00325068 | 0.0144312 | -2.7581963 |
| Tbrg4 | 1448796_s_a0.5241685 | | 8.82335635 | 4.91463329 | 0.00110822 | 0.00627839 | -1.5996777 |
| Spr | 1432372_a_ 0.52503715 | | 9.88514094 | 5.40576941 | 0.00060128 | 0.00396716 | -0.9365442 |
| Retsat | 1424715_at 0.52522547 | | 9.47143888 | 6.56638693 | 0.00016108 | 0.00151904 | 0.49954399 |
| Tac1 | 1416783_at 0.52536366 | | 8.03924758 | 5.84980275 | 0.00035595 | 0.00271299 | -0.3659655 |
| Fmod | 1437718_x_a0.52655606 | | 7.57874933 | 5.6596393 | 0.00044412 | 0.00316395 | -0.6070143 |
| Mmachc | 1418915_at 0.52662531 | | 10.031314 | 5.74603204 | 0.00040141 | 0.00294614 | -0.4968955 |
| Klf9 | 1422264_s_a0.52688617 | | 8.25081482 | 4.2661138 | 0.00262084 | 0.01221838 | -2.5274101 |
| Gid4 | 1438006_at 0.52766678 | | 9.23454218 | 6.87594584 | 0.00011653 | 0.00119321 | 0.85350902 |
| Slc25a35 | 1451489_at 0.52829463 | | 8.08767072 | 3.69101868 | 0.00591927 | 0.02286816 | -3.396855 |
| Prkaca | 1450519_a_ 0.52832608 | | 11.2253893 | 3.01896748 | 0.01622172 | 0.04996888 | -4.4553416 |
| NA | 1435716_x_a0.52861047 | | 11.0429702 | 5.87903018 | 0.00034419 | 0.00265367 | -0.3293504 |
| Mmd | 1423488_at 0.52892648 | | 9.48377278 | 2.72965353 | 0.02540489 | 0.07088501 | -4.9175896 |
| Ehhadh | 1448382_at 0.53099551 | | 7.52345927 | 4.89003005 | 0.00114372 | 0.00643626 | -1.633795 |
| Sorbs1 | 1417358_s_a0.53113711 | | 9.50354645 | 3.89791503 | 0.0043914 | 0.01813961 | -3.0794207 |
| Asb10 | 1421466_at 0.5312009 | | 10.1022323 | 5.75124024 | 0.00039898 | 0.00293698 | -0.4902894 |
| Dlat | 1426264_at 0.53203518 | | 11.2504342 | 7.61646906 | 5.60E-05 | 0.00069384 | 1.65528416 |
| Dnajb5 | 1421961_a_ 0.53306373 | | 8.44392358 | 6.23456472 | 0.00023072 | 0.00197403 | 0.10699086 |
| Coq5 | 1417264_at 0.53321154 | | 10.6966357 | 7.56184828 | 5.90E-05 | 0.00072229 | 1.59820851 |
| Dusp7 | 1427416_x_a0.53368376 | | 9.1076181 | 4.39429006 | 0.00220003 | 0.01067324 | -2.3394221 |

| Tmem70 | 1424541_at 0.53418493 | 10.538942 | 7.82899779 | 4.59E-05 | 0.00060786 | 1.87438595 |
| --- | --- | --- | --- | --- | --- | --- |
| Dnajc28 | 1420542_at 0.53448944 | 8.23283457 | 6.53323906 | 0.00016687 | 0.00155874 | 0.46094954 |
| Igfbp4 | 1437406_x_a0.5346082 | 10.9446661 | 3.12105618 | 0.01387156 | 0.04429295 | -4.292624 |
| Sccpdh | 1426510_at 0.53468839 | 8.88087846 | 5.80034425 | 0.00037687 | 0.00282497 | -0.428187 |
| Gsta3 | 1423437_at 0.53511044 | 7.10890273 | 7.53594059 | 6.05E-05 | 0.0007345 | 1.57102531 |
| Tmem143 | 1451304_at 0.53559542 | 9.39674709 | 5.83260207 | 0.00036308 | 0.00275252 | -0.3875675 |
| Acss2 | 1422479_at 0.53715624 | 9.60276932 | 3.71964668 | 0.00567769 | 0.02214281 | -3.3526455 |
| Pts | 1450660_at 0.53798357 | 9.57138216 | 7.45815765 | 6.52E-05 | 0.0007775 | 1.48897994 |
| Speg | 1425968_s_a0.53815329 | 11.8879967 | 6.43876639 | 0.00018466 | 0.00167869 | 0.35020536 |
| Ccdc85a | 1427157_at 0.53816215 | 7.00450809 | 4.60806923 | 0.00165193 | 0.00855336 | -2.0309066 |
| Rtn4r | 1419732_at 0.53834411 | 9.30324931 | 7.77268448 | 4.83E-05 | 0.00062893 | 1.81678758 |
| Igfbp5 | 1452114_s_a0.53843514 | 10.9942539 | 2.41696474 | 0.04148958 | 0.10237124 | -5.4145152 |
| Slc24a3 | 1424308_at 0.53891518 | 8.09687066 | 2.82240759 | 0.02198607 | 0.06333977 | -4.7693798 |
| Ndufs1 | 1425143_a_ 0.53903168 | 12.4444128 | 7.54911563 | 5.97E-05 | 0.00072918 | 1.58485796 |
| Pfkfb1 | 1427213_at 0.53905646 | 8.11289475 | 6.44403417 | 0.00018362 | 0.001674 | 0.35640978 |
| Gsn | 1456569_x_a0.53906396 | 6.57207739 | 4.39715853 | 0.00219149 | 0.01064353 | -2.3352406 |
| Adk | 1438292_x_a0.53920005 | 12.1627157 | 8.31494366 | 2.95E-05 | 0.00045301 | 2.35822447 |
| Ethe1 | 1417203_at 0.53952781 | 9.79302414 | 5.52603224 | 0.00052033 | 0.00355075 | -0.7793283 |
| Bpifb1 | 1423719_at 0.54028641 | 6.77305405 | 2.19699884 | 0.05864263 | 0.13249042 | -5.7582896 |
| Gpm6a | 1426442_at 0.54061094 | 7.92562582 | 2.19473627 | 0.05885145 | 0.13281674 | -5.7617869 |
| Cmbl | 1451322_at 0.54125799 | 9.44883117 | 6.03896261 | 0.00028695 | 0.0023308 | -0.131011 |
| Npr3 | 1448024_at 0.54183021 | 7.01695257 | 4.03698181 | 0.00360522 | 0.01562608 | -2.8689021 |
| Dkk3 | 1448669_at 0.54216673 | 8.7390583 | 1.12796564 | 0.29142424 | 0.41695037 | -7.2200265 |
| Cmc2 | 1427921_s_a0.5422346 | 10.2057003 | 8.04419846 | 3.76E-05 | 0.00053179 | 2.09153055 |
| Hspa1a | 1452388_at 0.54318723 | 8.59028597 | 1.90029089 | 0.09322811 | 0.18522087 | -6.2074248 |
| Acad11 | 1454647_at 0.54321904 | 10.4261611 | 4.83639162 | 0.00122548 | 0.00680025 | -1.708473 |
| Mrpl9 | 1430976_a_ 0.54329051 | 9.1723763 | 6.12648709 | 0.00026012 | 0.00215851 | -0.0238981 |
| Mccc2 | 1428021_at 0.54410543 | 8.1325797 | 7.05426726 | 9.72E-05 | 0.00104156 | 1.05224173 |
| NA | 1417505_s_a0.54431577 | 10.366897 | 5.1304253 | 0.00084358 | 0.00508256 | -1.3041176 |
| Rps6ka2 | 1417542_at 0.54467467 | 9.72742876 | 3.78938801 | 0.00513208 | 0.02047599 | -3.2453231 |
| Fcrls | 1448891_at 0.54495876 | 8.11391934 | 7.62169548 | 5.57E-05 | 0.00069305 | 1.6607289 |
| Tmem35a | 1416710_at 0.54514338 | 8.09591694 | 8.09149572 | 3.60E-05 | 0.00051486 | 2.1386349 |
| Tob1 | 1423176_at 0.54544227 | 10.5402116 | 3.96019384 | 0.00401872 | 0.01694567 | -2.9848492 |
| Fblim1 | 1418569_at 0.54660705 | 10.689814 | 4.44230265 | 0.00206171 | 0.0101507 | -2.2695817 |
| Copg2os2 | 1427320_at 0.54679396 | 7.13046009 | 5.76132762 | 0.00039432 | 0.00291512 | -0.4775051 |
| Narf | 1425344_at 0.54790094 | 8.9944352 | 4.14106918 | 0.00311606 | 0.01399238 | -2.7129251 |
| Fmod | 1438966_x_a0.54829789 | 6.57234814 | 3.98775394 | 0.00386474 | 0.01642768 | -2.94315 |
| Dhrs4 | 1419382_a_ 0.54846522 | 9.2970578 | 5.67917194 | 0.00043405 | 0.00310875 | -0.5820283 |
| Asph | 1425275_at 0.54953846 | 10.5220948 | 6.35530861 | 0.00020214 | 0.00178879 | 0.25144263 |
| Sdha | 1426689_s_a0.55018634 | 12.7617322 | 8.40331754 | 2.73E-05 | 0.00043065 | 2.44375468 |
| Gata6 | 1425463_at 0.55035593 | 8.85500764 | 5.63161608 | 0.00045903 | 0.00324266 | -0.6429526 |
| Casq1 | 1422598_at 0.55111606 | 8.50909072 | 7.28241756 | 7.74E-05 | 0.00087953 | 1.30118359 |
| Poldip2 | 1450727_a_ 0.55139625 | 10.2321882 | 6.08917105 | 0.0002712 | 0.00222939 | -0.0694429 |
| Doc2g | 1450707_at 0.55179138 | 10.9410599 | 3.73193676 | 0.00557723 | 0.02182225 | -3.3336936 |
| Acot13 | 1417316_at 0.55279726 | 12.426834 | 5.91695061 | 0.00032957 | 0.00257503 | -0.2820157 |
| Slc25a20 | 1423109_s_a0.55282999 | 10.9327261 | 5.46221972 | 0.00056169 | 0.00376615 | -0.862498 |
| Bcat2 | 1425764_a_ 0.5530648 | 9.36089342 | 4.08889539 | 0.00335168 | 0.01477266 | -2.7909348 |
| Slc25a26 | 1428929_s_a0.55309197 | 9.47098725 | 6.18422975 | 0.00024393 | 0.00205503 | 0.04622021 |
| Plcb4 | 1425339_at 0.55358536 | 8.86356968 | 5.08765769 | 0.00088999 | 0.00530161 | -1.3621699 |
| Kcnj3 | 1421468_at 0.55364386 | 8.65364193 | 5.91372456 | 0.00033078 | 0.0025801 | -0.2860352 |
| Tspan12 | 1454604_s_a0.55407731 | 7.99815337 | 5.1026515 | 0.00087341 | 0.00522342 | -1.341788 |
| Gucy1a1 | 1420534_at 0.5542261 | 7.41895858 | 3.06710349 | 0.01506575 | 0.04731376 | -4.3785678 |
| Echs1 | 1452341_at 0.55444478 | 11.9992025 | 7.02397809 | 0.0001002 | 0.00106438 | 1.01874619 |
| Rab6b | 1434914_at 0.55766607 | 8.63533237 | 4.03177506 | 0.00363177 | 0.01570812 | -2.8767408 |
| Mgst3 | 1448300_at 0.55848056 | 12.5863808 | 9.43653667 | 1.15E-05 | 0.00023442 | 3.39199713 |
| Slc25a13 | 1449481_at 0.55906307 | 10.7804585 | 8.16254357 | 3.38E-05 | 0.00049436 | 2.20898015 |
| Sord | 1438183_x_a0.5593233 | 9.68043697 | 7.45715133 | 6.53E-05 | 0.00077785 | 1.4879142 |
| Pink1 | 1451148_at 0.55935389 | 11.8827371 | 7.93100686 | 4.17E-05 | 0.00057175 | 1.97789801 |
| Idh1 | 1422433_s_a0.55952911 | 10.9107425 | 5.46342794 | 0.00056087 | 0.00376179 | -0.8609181 |
| Acss2 | 1422478_a_ 0.55980742 | 8.8273972 | 4.77959162 | 0.00131905 | 0.00718933 | -1.7879971 |
| Tmem218 | 1417174_at 0.55994698 | 8.25813274 | 8.03744258 | 3.78E-05 | 0.00053375 | 2.08478417 |
| Retsat | 1424716_at 0.5605271 | 8.48072653 | 8.09614664 | 3.59E-05 | 0.00051338 | 2.14325494 |
| Kcnj11 | 1450515_at 0.56083422 | 8.42677461 | 4.81838937 | 0.00125433 | 0.00690461 | -1.733628 |
| Ccng1 | 1450017_at 0.56085765 | 12.3052602 | 5.80531868 | 0.00037471 | 0.0028162 | -0.421914 |
| Plin4 | 1418595_at 0.56105034 | 11.3113681 | 4.43922799 | 0.00207028 | 0.01018309 | -2.2740447 |
| Limch1 | 1435106_at 0.56121859 | 10.733465 | 6.67848715 | 0.00014308 | 0.00139479 | 0.6290634 |
| Afg1l | 1427073_at 0.56127825 | 9.73502395 | 6.15256191 | 0.00025267 | 0.00211239 | 0.00781884 |
| Dlat | 1426265_x_a0.5631183 | 11.2591062 | 7.78800887 | 4.77E-05 | 0.00062282 | 1.83249398 |
| Palld | 1427228_at 0.56324326 | 11.0946616 | 5.38756816 | 0.00061469 | 0.00403219 | -0.9605137 |
| Prdx3 | 1416292_at 0.56432979 | 11.9833085 | 7.9019571 | 4.29E-05 | 0.0005812 | 1.94852775 |
| Dcakd | 1460205_at 0.56603793 | 8.86096921 | 5.24300839 | 0.00073358 | 0.00457277 | -1.1525343 |
| Aqp4 | 1434449_at 0.56757176 | 7.15374462 | 6.83670502 | 0.00012134 | 0.00123188 | 0.80927582 |
| Echdc3 | 1418862_at 0.56760879 | 9.35442823 | 8.7031473 | 2.10E-05 | 0.00035653 | 2.72854418 |
| Smtn | 1452469_a_ 0.56821592 | 9.68905737 | 4.80550141 | 0.00127544 | 0.00699872 | -1.7516648 |
| Ednra | 1451691_at 0.56845266 | 8.14189106 | 5.41891542 | 0.00059179 | 0.00392282 | -0.9192609 |
| Myh11 | 1448962_at 0.56871324 | 7.89171471 | 2.32433198 | 0.04800054 | 0.11417136 | -5.5601099 |
| Khdrbs3 | 1453317_a_ 0.56903257 | 8.96633094 | 4.96875578 | 0.00103427 | 0.00595774 | -1.5249283 |
| Mmd | 1423489_at 0.5694513 | 10.0031642 | 3.1295284 | 0.01369321 | 0.04386544 | -4.2791401 |
| Ak3 | 1423718_at 0.57041663 | 10.1397321 | 4.0510725 | 0.00353443 | 0.01539384 | -2.8477059 |
| Pde2a | 1452202_at 0.57091612 | 8.60093304 | 5.86351088 | 0.00035038 | 0.00268226 | -0.3487782 |
| Gna12 | 1421026_at 0.57299846 | 8.73408953 | 3.44010726 | 0.0085688 | 0.03039805 | -3.7878856 |
| Cmc2 | 1427922_at 0.57359939 | 9.04195958 | 8.46032147 | 2.59E-05 | 0.0004166 | 2.4985356 |
| Pank1 | 1418715_at 0.57413565 | 9.80005427 | 5.62674109 | 0.00046168 | 0.00325428 | -0.6492155 |
| Fbln1 | 1422540_at 0.57427844 | 9.506254 | 2.59337406 | 0.03144491 | 0.08319799 | -5.1349043 |
| Acat1 | 1424182_at 0.57519177 | 11.2316734 | 7.43457412 | 6.67E-05 | 0.00079006 | 1.46397489 |

| Isca2 | 1424154_a_ 0.5755139 | 10.6887596 | 6.10364111 | 0.00026685 | 0.0022013 | -0.0517604 |
| --- | --- | --- | --- | --- | --- | --- |
| Acaa2 | 1428146_s_a0.57561387 | 12.9569498 | 6.08075158 | 0.00027378 | 0.00224504 | -0.0797442 |
| Lpin1 | 1418288_at 0.57613117 | 11.2690619 | 3.7321894 | 0.00557518 | 0.02182144 | -3.3333042 |
| Angptl2 | 1450085_at 0.57630669 | 8.28073471 | 5.30278511 | 0.00068162 | 0.00435536 | -1.0727764 |
| Sspn | 1417644_at 0.57721911 | 11.8590973 | 8.52976677 | 2.44E-05 | 0.00039758 | 2.56486532 |
| Spock2 | 1435026_at 0.57794248 | 8.20807464 | 5.01943172 | 0.00096988 | 0.00566637 | -1.4553156 |
| Coq5 | 1417265_s_a0.57816072 | 9.9730296 | 7.09362321 | 9.34E-05 | 0.00101451 | 1.0956062 |
| Foxo3 | 1434832_at 0.5783504 | 9.98356646 | 4.91762491 | 0.00110398 | 0.00625765 | -1.5955351 |
| Tef | 1450184_s_a0.57888158 | 8.28960942 | 8.33671515 | 2.89E-05 | 0.00044677 | 2.37936386 |
| Smr2 | 1426180_a_ 0.57906603 | 10.8057859 | 8.14379963 | 3.44E-05 | 0.00049956 | 2.19046946 |
| Cidea | 1417956_at 0.57950723 | 10.3107294 | 1.43249508 | 0.18919812 | 0.306658 | -6.8568743 |
| Fmod | 1437324_x_a0.58046398 | 7.48979385 | 9.07443826 | 1.54E-05 | 0.00028726 | 3.07010858 |
| Dlst | 1423710_at 0.58113781 | 12.1330921 | 6.05226834 | 0.00028268 | 0.00229977 | -0.1146626 |
| Gpsm1 | 1423690_s_a0.5825472 | 10.4802035 | 5.77843645 | 0.00038656 | 0.00287818 | -0.4558537 |
| Eif1ax | 1449576_at 0.58364371 | 9.03390866 | 7.74577645 | 4.96E-05 | 0.00064046 | 1.78915005 |
| Heyl | 1419302_at 0.58518332 | 8.53034205 | 7.04167061 | 9.84E-05 | 0.00105141 | 1.03832447 |
| Gas1 | 1416855_at 0.58532486 | 10.2895799 | 3.53563273 | 0.00743588 | 0.02723927 | -3.6382995 |
| Uqcr11 | 1448292_at 0.58592562 | 13.1161121 | 8.30097461 | 2.99E-05 | 0.0004565 | 2.34463729 |
| Prss23 | 1437671_x_a0.58675998 | 7.88775681 | 2.73800038 | 0.02507601 | 0.07020045 | -4.9042585 |
| Nov | 1426852_x_a0.58691626 | 8.12768657 | 3.27515595 | 0.01097579 | 0.03694418 | -4.0479777 |
| Rtn4ip1 | 1417668_at 0.58703939 | 9.18290822 | 6.56593654 | 0.00016115 | 0.00151914 | 0.49902049 |
| NA | 1424505_at 0.5873279 | 9.30968261 | 5.04532985 | 0.00093867 | 0.00553204 | -1.4198805 |
| Tmem163 | 1428077_at 0.58765112 | 8.38892059 | 2.49423976 | 0.03674163 | 0.09311602 | -5.2923483 |
| Cd59a | 1429830_a_ 0.58819935 | 10.229904 | 6.85651241 | 0.00011889 | 0.00121185 | 0.83162599 |
| Ank1 | 1419421_at 0.58919807 | 10.9671623 | 9.83079507 | 8.42E-06 | 0.00018834 | 3.73058023 |
| NA | 1422230_s_a0.58936372 | 7.3975421 | 2.24219278 | 0.05462149 | 0.12550498 | -5.6882455 |
| Mgll | 1450391_a_ 0.58982637 | 9.29743798 | 3.59660428 | 0.00679662 | 0.02533244 | -3.5432695 |
| Ccng1 | 1450016_at 0.58986258 | 11.8939356 | 6.6020252 | 0.0001551 | 0.00148132 | 0.54088692 |
| Serpinh1 | 1456733_x_a0.58987587 | 11.0291843 | 1.74502706 | 0.11841461 | 0.21933286 | -6.4326461 |
| Prg4 | 1449824_at 0.59001146 | 8.79951682 | 6.44826408 | 0.00018278 | 0.00166962 | 0.36138926 |
| Supv3l1 | 1460557_at 0.59102675 | 9.64118288 | 5.99826751 | 0.00030045 | 0.00241023 | -0.1811569 |
| Prss23 | 1431057_a_ 0.591251 | 7.95498495 | 2.85745143 | 0.02082104 | 0.06090363 | -4.7133634 |
| Cmya5 | 1427312_at 0.59137727 | 12.2895574 | 6.41163705 | 0.00019015 | 0.0017101 | 0.31819717 |
| Map2k6 | 1426850_a_ 0.59139545 | 8.04026642 | 10.0877259 | 6.92E-06 | 0.00016516 | 3.94488818 |
| Serpine2 | 1416666_at 0.59213105 | 9.55133125 | 4.05028764 | 0.00353833 | 0.0154068 | -2.8488858 |
| NA | 1436917_s_a0.59247597 | 10.2548939 | 3.76361712 | 0.00532689 | 0.0210379 | -3.2849177 |
| Dnajc28 | 1420543_at 0.59285607 | 9.57234648 | 9.26340442 | 1.32E-05 | 0.00025798 | 3.23943348 |
| Pex19 | 1416425_at 0.59291774 | 10.7171962 | 7.47549732 | 6.41E-05 | 0.00076952 | 1.50732632 |
| Ivd | 1449001_at 0.59317871 | 10.896968 | 7.90678224 | 4.27E-05 | 0.00058012 | 1.95341203 |
| Lum | 1423607_at 0.59381327 | 10.8725053 | 2.57450498 | 0.0323897 | 0.08500142 | -5.1649233 |
| Rmdn1 | 1438034_at 0.59453233 | 8.39055883 | 3.7296148 | 0.00559606 | 0.02188086 | -3.3372729 |
| Gbe1 | 1420654_a_ 0.59598891 | 8.73039465 | 3.65371421 | 0.0062506 | 0.02378258 | -3.4545948 |
| Abcc9 | 1420408_a_ 0.59631438 | 10.5775617 | 6.56730634 | 0.00016092 | 0.00151819 | 0.50061253 |
| Acaa2 | 1428145_at 0.59637101 | 12.9388841 | 7.99524668 | 3.93E-05 | 0.00054792 | 2.04254509 |
| Aldh4a1 | 1452375_at 0.59666282 | 9.77491997 | 6.80634986 | 0.00012521 | 0.00125655 | 0.77493337 |
| Tfrc | AFFX-TransR 0.59873172 | 7.59595909 | 4.47388617 | 0.00197586 | 0.00982948 | -2.2238126 |
| Tpm2 | 1419739_at 0.59927881 | 8.29446453 | 4.88391827 | 0.00115273 | 0.00647893 | -1.6422835 |
| Plcb4 | 1425338_at 0.59930931 | 7.67391313 | 4.81704428 | 0.00125652 | 0.00691328 | -1.7355094 |
| Efemp1 | 1427183_at 0.59978274 | 8.37974696 | 2.7757002 | 0.02364417 | 0.0671363 | -4.8440285 |
| Mmp15 | 1422597_at 0.6011169 | 8.61527095 | 3.62525142 | 0.00651666 | 0.02451309 | -3.4987474 |
| Col15a1 | 1448755_at 0.60142538 | 9.38198713 | 3.50786215 | 0.0077479 | 0.02812346 | -3.6817006 |
| Idh3a | 1432016_a_ 0.60144728 | 13.0114172 | 9.02805132 | 1.60E-05 | 0.00029436 | 3.02808559 |
| Gsta4 | 1416368_at 0.60148019 | 10.619367 | 6.37056801 | 0.00019881 | 0.00176417 | 0.26956606 |
| Egln1 | 1451110_at 0.60193253 | 10.8264839 | 3.08657529 | 0.01462284 | 0.04617847 | -4.347536 |
| Cpeb3 | 1456048_at 0.60199486 | 8.86446407 | 5.29839268 | 0.0006853 | 0.00437271 | -1.07862 |
| Gpsm1 | 1423689_a_ 0.6028067 | 10.0008534 | 4.37295487 | 0.00226469 | 0.01091224 | -2.3705582 |
| Timp2 | 1454677_at 0.60300186 | 10.6444475 | 2.42214232 | 0.04115298 | 0.10173905 | -5.4063482 |
| Cyp2d22 | 1419040_at 0.60306102 | 8.27959657 | 4.53638025 | 0.00181717 | 0.00920964 | -2.133657 |
| Slc40a1 | 1417061_at 0.60332408 | 8.94808139 | 5.49110284 | 0.00054254 | 0.00366592 | -0.8247834 |
| Gpt | 1426502_s_a0.60413902 | 9.75892386 | 6.39367169 | 0.00019389 | 0.0017368 | 0.29695002 |
| Ms4a1 | 1423226_at 0.60525389 | 7.767291 | 1.77119231 | 0.11376397 | 0.21326045 | -6.3952701 |
| Acss1 | 1416617_at 0.60596484 | 11.6134428 | 4.97095683 | 0.00103138 | 0.00594712 | -1.5218972 |
| Fmod | 1415939_at 0.60600765 | 8.091737 | 7.966697 | 4.04E-05 | 0.00055787 | 2.0138655 |
| Asb15 | 1460281_at 0.60606658 | 7.73453527 | 4.90364128 | 0.00112393 | 0.00634534 | -1.6149097 |
| Sorcs2 | 1419358_at 0.60624348 | 8.2751354 | 9.87953054 | 8.11E-06 | 0.00018383 | 3.77160686 |
| Abcd3 | 1416679_at 0.60664912 | 10.8726825 | 7.08823698 | 9.39E-05 | 0.00101758 | 1.08968186 |
| Miga2 | 1433493_at 0.60706551 | 9.87912598 | 4.92786399 | 0.00108962 | 0.0062026 | -1.5813662 |
| Klf9 | 1436763_a_ 0.60707027 | 9.62771172 | 7.08966498 | 9.38E-05 | 0.00101661 | 1.09125285 |
| Nudt8 | 1450111_a_ 0.60729304 | 11.011085 | 5.29784518 | 0.00068576 | 0.00437401 | -1.0793485 |
| Cul3 | 1422795_at 0.60857889 | 10.1332479 | 7.45953334 | 6.51E-05 | 0.00077728 | 1.49043669 |
| Lims2 | 1424408_at 0.6095642 | 10.0054713 | 4.90949414 | 0.00111553 | 0.00630893 | -1.6067971 |
| Ech1 | 1448491_at 0.60973048 | 13.8146894 | 8.94469269 | 1.71E-05 | 0.00030811 | 2.95210839 |
| Ogdh | 1451274_at 0.61121488 | 12.7959028 | 4.92695445 | 0.00109089 | 0.0062067 | -1.5826242 |
| Mgll | 1426785_s_a0.61196953 | 10.9198483 | 4.42086678 | 0.00212227 | 0.01039151 | -2.3007237 |
| D10Jhu81e | 1449000_at 0.61233503 | 12.4178175 | 10.050203 | 7.12E-06 | 0.00016847 | 3.91389257 |
| Sirt3 | 1417892_a_ 0.61246114 | 9.97035465 | 8.62988403 | 2.24E-05 | 0.00037388 | 2.65971255 |
| Gata6 | 1425464_at 0.61359246 | 10.2480757 | 6.71936816 | 0.00013708 | 0.00134471 | 0.67591598 |
| Hacd1 | 1456315_a_ 0.6143544 | 11.1812556 | 7.93729076 | 4.15E-05 | 0.00056914 | 1.98424003 |
| Ptgr2 | 1429474_at 0.61461341 | 9.26611239 | 6.37838482 | 0.00019713 | 0.00175376 | 0.27883859 |
| Ivns1abp | 1420961_a_ 0.61670709 | 9.13260842 | 3.74409145 | 0.00547973 | 0.02152614 | -3.3149668 |
| Myzap | 1455180_at 0.61801006 | 12.2851205 | 7.80463259 | 4.69E-05 | 0.00061679 | 1.84950475 |
| Fhod3 | 1435551_at 0.61994409 | 11.3897315 | 8.44124049 | 2.64E-05 | 0.00042183 | 2.48023245 |
| Me1 | 1430307_a_ 0.62024048 | 10.6264688 | 4.31961176 | 0.00243549 | 0.01156337 | -2.4486774 |
| Tesc | 1418743_a_ 0.62103579 | 9.42331746 | 3.78656957 | 0.00515301 | 0.02051981 | -3.2496497 |
| Ptger3 | 1450344_a_ 0.62198904 | 6.88936081 | 3.19226969 | 0.01244485 | 0.04079365 | -4.1793973 |

| Poldip2 | 1422732_at | 0.62207385 | 9.92734406 | 4.35544651 | 0.00231928 | 0.01112097 | -2.396156 |
| --- | --- | --- | --- | --- | --- | --- | --- |
| Dmpk | 1434944_at | 0.62299622 | 11.7821619 | 2.8838326 | 0.01998618 | 0.05894026 | -4.6711952 |
| Coro6 | 1426122_a_ | 0.62304702 | 10.060476 | 4.06781163 | 0.00345227 | 0.01511324 | -2.8225584 |
| Dsp | 1427610_at | 0.62389973 | 9.62005993 | 3.13809235 | 0.01351536 | 0.0433774 | -4.2655136 |
| Klhl21 | 1428081_at | 0.62473736 | 9.24565398 | 7.42113381 | 6.76E-05 | 0.00079752 | 1.44969741 |
| Dnajb5 | 1421962_at | 0.62487835 | 9.67216112 | 4.06421058 | 0.00346977 | 0.01517229 | -2.8279653 |
| Fgf13 | 1418498_at | 0.62594985 | 8.37556918 | 4.29254155 | 0.00252745 | 0.01189788 | -2.4884683 |
| Dnajb5 | 1450436_s_a0.62660131 | | 10.6600729 | 4.66619426 | 0.0015299 | 0.00804857 | -1.9481259 |
| Sord | 1426584_a_ 0.62714006 | | 11.1323928 | 8.49456638 | 2.52E-05 | 0.00040662 | 2.53129981 |
| Fem1a | 1423583_at 0.62980499 | | 10.6182975 | 5.81379262 | 0.00037105 | 0.00279614 | -0.4112355 |
| Ptp4a3 | 1418181_at 0.62996757 | | 11.9064288 | 4.09362353 | 0.00332956 | 0.01468943 | -2.7838509 |
| Hibch | 1451512_s_a0.63048979 | | 10.3179405 | 7.70795265 | 5.14E-05 | 0.00065595 | 1.7501738 |
| Pon1 | 1418190_at 0.63143367 | | 7.44747876 | 5.61999995 | 0.00046537 | 0.0032719 | -0.6578812 |
| Dkk3 | 1417312_at 0.63177879 | | 8.67875765 | 1.12192514 | 0.29383943 | 0.41949911 | -7.2266196 |
| Smyd1 | 1450203_at 0.6325108 | | 10.1898746 | 5.1252331 | 0.00084907 | 0.00510487 | -1.3111516 |
| Hccs | 1420890_at 0.63334919 | | 10.7563364 | 7.27797261 | 7.77E-05 | 0.00088286 | 1.29638953 |
| Pfkm | 1416780_at 0.63373447 | | 13.0094746 | 7.27110378 | 7.83E-05 | 0.00088717 | 1.28897689 |
| Casq2 | 1422529_s_a0.63374734 | | 13.3174389 | 9.69534649 | 9.35E-06 | 0.00020327 | 3.61561375 |
| Ank3 | 1452124_at 0.63392607 | | 9.54915027 | 8.59794269 | 2.30E-05 | 0.0003809 | 2.6295517 |
| NA | 1438317_a_ 0.63440932 | | 10.4999062 | 8.24789202 | 3.13E-05 | 0.00047161 | 2.2928361 |
| Sparcl1 | 1416114_at 0.63452969 | | 10.6541007 | 2.69663534 | 0.02674979 | 0.07381159 | -4.970306 |
| Map1lc3a | 1451290_at 0.63503319 | | 12.4647171 | 8.03397671 | 3.80E-05 | 0.00053477 | 2.08132142 |
| Egln1 | 1423785_at 0.63531968 | | 12.3162224 | 7.90044125 | 4.29E-05 | 0.0005812 | 1.94699283 |
| Mlf1 | 1418589_a_ 0.63645562 | | 10.8231774 | 9.28107121 | 1.30E-05 | 0.00025586 | 3.25511295 |
| Jph2 | 1421453_at 0.63739053 | | 9.27847041 | 5.17152794 | 0.00080146 | 0.00489245 | -1.248569 |
| Pygm | 1448602_at 0.63778955 | | 12.7240326 | 4.74942392 | 0.00137187 | 0.0074061 | -1.8304197 |
| Fastk | 1460635_at 0.63817292 | | 10.7719632 | 6.22817783 | 0.00023236 | 0.00198337 | 0.09929792 |
| Ttc7b | 1433460_at 0.6388518 | | 9.5627233 | 4.82322056 | 0.00124652 | 0.00687661 | -1.7268727 |
| Cpt2 | 1416772_at 0.63905012 | | 12.0835803 | 7.66309542 | 5.36E-05 | 0.00067711 | 1.70375632 |
| Slc25a11 | 1426586_at 0.63997885 | | 12.1322429 | 8.74131315 | 2.04E-05 | 0.00034806 | 2.76421093 |
| Nudt4 | 1418505_at 0.6404899 | | 11.8328397 | 4.04177155 | 0.00358099 | 0.01555373 | -2.8616942 |
| Lamb3 | 1417812_a_ 0.64254165 | | 8.0963465 | 7.52493367 | 6.11E-05 | 0.00074032 | 1.55945476 |
| Uqcc1 | 1416436_a_ 0.64287398 | | 10.3492796 | 7.94279499 | 4.13E-05 | 0.00056695 | 1.98979191 |
| Igfbp4 | 1437405_a_ 0.643489 | | 11.7753094 | 2.5229954 | 0.03511826 | 0.09000715 | -5.2467522 |
| Lyve1 | 1453128_at 0.64364442 | | 8.64468868 | 10.4751217 | 5.19E-06 | 0.00013313 | 4.25903592 |
| Gpt2 | 1455007_s_a0.64455031 | | 8.13834121 | 9.65984111 | 9.62E-06 | 0.00020799 | 3.58524545 |
| Myl1 | 1452651_a_ 0.64455786 | | 10.8183864 | 2.30679702 | 0.04934342 | 0.11652717 | -5.5875471 |
| Csdc2 | 1451147_x_a0.64469427 | | 8.33733465 | 4.35379209 | 0.00232451 | 0.011139 | -2.3985769 |
| Hrc | 1419109_at 0.64495995 | | 12.977916 | 9.06748248 | 1.55E-05 | 0.00028797 | 3.06381879 |
| Eif4e | 1450909_at 0.64511002 | | 8.44974633 | 9.53443813 | 1.06E-05 | 0.00022237 | 3.47720377 |
| Nnat | 1423506_a_ 0.64894224 | | 7.12061671 | 5.58541566 | 0.00048482 | 0.00336719 | -0.7024376 |
| Ivd | 1418238_at 0.65051978 | | 11.3037739 | 8.43594877 | 2.65E-05 | 0.00042254 | 2.47515045 |
| Gstm1 | 1425627_x_a0.65183029 | | 9.94957199 | 9.01497994 | 1.62E-05 | 0.00029585 | 3.01621095 |
| Gsta3 | 1423436_at 0.65183456 | | 7.2980375 | 7.4725842 | 6.43E-05 | 0.00077119 | 1.50424634 |
| Elovl6 | 1417403_at 0.65254965 | | 7.67536803 | 1.86627889 | 0.09826838 | 0.19228353 | -6.2574374 |
| Slc35e1 | 1434103_at 0.65268008 | | 10.025966 | 5.82454507 | 0.00036647 | 0.00277082 | -0.3976998 |
| D2hgdh | 1437243_at 0.65326151 | | 9.35479521 | 8.64677456 | 2.21E-05 | 0.00036958 | 2.67562424 |
| Zfyve21 | 1424669_at 0.65465081 | | 8.51952888 | 5.94120332 | 0.00032057 | 0.00252211 | -0.2518426 |
| Phkg1 | 1422315_x_a0.65477612 | | 7.50963576 | 7.1860961 | 8.52E-05 | 0.00094436 | 1.19680214 |
| Bdh1 | 1452257_at 0.65507401 | | 10.7075724 | 3.18730373 | 0.01253917 | 0.04102578 | -4.1872842 |
| Dusp1 | 1448830_at 0.65510315 | | 10.9329739 | 5.56574925 | 0.00049628 | 0.00342474 | -0.7278481 |
| Plagl1 | 1426208_x_a0.65511054 | | 9.43212712 | 2.04736716 | 0.0741456 | 0.15744845 | -5.9873952 |
| Grb14 | 1417673_at 0.65832769 | | 11.4244193 | 9.14338679 | 1.45E-05 | 0.00027587 | 3.13223531 |
| Tagln | 1423505_at 0.66014422 | | 8.81756097 | 2.47682677 | 0.03776134 | 0.09503158 | -5.3199257 |
| Pkdcc | 1460411_s_a0.66019711 | | 9.41193432 | 6.95179567 | 0.00010782 | 0.00111969 | 0.93849425 |
| Mlxipl | 1419185_a_ 0.66032822 | | 9.2013931 | 5.64008071 | 0.00045447 | 0.00322284 | -0.6320858 |
| Penk | 1427038_at 0.66074928 | | 7.92500367 | 6.42115251 | 0.00018821 | 0.00170106 | 0.32943439 |
| Cd36 | 1450884_at 0.66084052 | | 11.1081684 | 7.45961314 | 6.51E-05 | 0.00077728 | 1.49052119 |
| Tmem246 | 1423679_at 0.66088251 | | 9.98287124 | 8.67587095 | 2.15E-05 | 0.00036342 | 2.70297412 |
| Elovl6 | 1417404_at 0.66104767 | | 8.28105639 | 2.17750771 | 0.06046576 | 0.13547626 | -5.7883869 |
| Cyp1a1 | 1422217_a_ 0.66167949 | | 8.20874403 | 8.43578751 | 2.65E-05 | 0.00042254 | 2.47499554 |
| Atp2a2 | 1427251_at 0.66174305 | | 10.5443991 | 4.91388296 | 0.00110928 | 0.00628141 | -1.6007169 |
| Tmod1 | 1422754_at 0.66405421 | | 12.2235373 | 8.3990489 | 2.74E-05 | 0.00043197 | 2.43964029 |
| Mid1ip1 | 1416840_at 0.6641198 | | 11.0340065 | 6.39506006 | 0.0001936 | 0.00173487 | 0.29859345 |
| Rpl3l | 1449398_at 0.66425808 | | 11.1495599 | 7.767922 | 4.86E-05 | 0.00063102 | 1.81190143 |
| Zfp612 | 1427104_at 0.66576225 | | 8.1281185 | 7.31041288 | 7.53E-05 | 0.00085952 | 1.33132724 |
| Coq3 | 1437364_at 0.66639108 | | 8.52536571 | 7.72606657 | 5.05E-05 | 0.00064864 | 1.7688582 |
| Mrpl28 | 1416284_at 0.66747641 | | 10.9510285 | 7.69543693 | 5.20E-05 | 0.00066151 | 1.73724393 |
| Oxld1 | 1460254_at 0.6681775 | | 9.21193896 | 9.9488103 | 7.69E-06 | 0.00017716 | 3.82962306 |
| Cry2 | 1426383_at 0.66836283 | | 8.09965175 | 9.46760338 | 1.12E-05 | 0.00023108 | 3.41911783 |
| Csdc2 | 1423845_at 0.66857215 | | 8.3161079 | 5.25054268 | 0.0007268 | 0.00454423 | -1.1424539 |
| P3h4 | 1455288_at 0.66899545 | | 9.23685052 | 7.86094226 | 4.45E-05 | 0.000596 | 1.90691487 |
| Mterf2 | 1455700_at 0.6701021 | | 9.08157289 | 9.9321515 | 7.79E-06 | 0.0001787 | 3.8157052 |
| Ppif | 1416940_at 0.67107155 | | 11.5578162 | 6.37170602 | 0.00019856 | 0.00176326 | 0.27091648 |
| Kcnn2 | 1448927_at 0.67151027 | | 7.70233761 | 11.3574873 | 2.79E-06 | 8.75E-05 | 4.93712288 |
| Coq10a | 1433628_at 0.67173804 | | 11.1664214 | 7.40157102 | 6.89E-05 | 0.00080695 | 1.42888091 |
| Hccs | 1420889_at 0.67339526 | | 9.47069052 | 6.54947402 | 0.000164 | 0.0015396 | 0.47986906 |
| Adk | 1416319_at 0.67340772 | | 10.3439245 | 8.88170434 | 1.81E-05 | 0.00032017 | 2.89430108 |
| Bves | 1419003_at 0.67495547 | | 10.1836333 | 7.8712139 | 4.41E-05 | 0.00059231 | 1.91735233 |
| NA | 1435630_s_a0.67505134 | | 9.07433614 | 4.83345894 | 0.00123013 | 0.00681272 | -1.7125678 |
| Bckdha | 1416647_at 0.67516728 | | 11.4148351 | 7.91724498 | 4.23E-05 | 0.00057662 | 1.96399492 |
| Mut | 1416838_at 0.67711706 | | 10.9229598 | 7.6292557 | 5.53E-05 | 0.0006895 | 1.6685998 |
| Pitpnc1 | 1428878_a_ 0.67767999 | | 9.6808104 | 8.26873313 | 3.07E-05 | 0.00046509 | 2.31320624 |
| Fah | 1417220_at 0.67970221 | | 9.20887033 | 8.30816467 | 2.97E-05 | 0.0004542 | 2.35163311 |
| Mrpl12 | 1452048_at 0.68121207 | | 10.9149668 | 10.0469349 | 7.14E-06 | 0.00016871 | 3.91118815 |

| Grsf1 | 1433457_s_a0.68293371 | 11.1153227 | 5.32741994 | 0.00066139 | 0.00426209 | -1.0400531 |
| --- | --- | --- | --- | --- | --- | --- |
| Armh4 | 1450770_at 0.68299737 | 7.73138244 | 7.26324875 | 7.89E-05 | 0.00089274 | 1.28049352 |
| Hibadh | 1423780_at 0.68314618 | 11.6198389 | 8.47806121 | 2.55E-05 | 0.00041164 | 2.51552186 |
| Rgs5 | 1417466_at 0.68383647 | 12.5636547 | 4.82584475 | 0.0012423 | 0.00686668 | -1.7232048 |
| Prodh | 1417629_at 0.68506155 | 8.84672616 | 7.90085153 | 4.29E-05 | 0.0005812 | 1.9474083 |
| Gnao1 | 1421152_a_ 0.6853525 | 8.77430354 | 3.77212241 | 0.00526174 | 0.0208321 | -3.2718418 |
| Acads | 1460216_at 0.68641299 | 11.0190984 | 8.12778681 | 3.49E-05 | 0.00050361 | 2.17462876 |
| Alas1 | 1424126_at 0.68642414 | 11.4865418 | 8.8947436 | 1.79E-05 | 0.00031793 | 2.90629602 |
| Hmgcs2 | 1423858_a_ 0.68761456 | 7.23492585 | 6.92406419 | 0.00011092 | 0.00114661 | 0.9075011 |
| Hadh | 1460184_at 0.68934752 | 12.7190188 | 8.63546811 | 2.23E-05 | 0.00037291 | 2.66497587 |
| Hlf | 1434735_at 0.68946766 | 8.44909667 | 10.2593302 | 6.09E-06 | 0.00014914 | 4.08535031 |
| Gpc1 | 1417389_at 0.69201571 | 11.4635552 | 4.16760355 | 0.00300312 | 0.01357971 | -2.6733867 |
| NA | 1421063_s_a0.69823733 | 9.03015127 | 5.25598899 | 0.00072194 | 0.00452382 | -1.135172 |
| Ndrg4 | 1436188_a_ 0.69843092 | 12.1365086 | 9.14272923 | 1.46E-05 | 0.00027587 | 3.13164469 |
| Amy1 | 1417765_a_ 0.69910337 | 7.629532 | 3.42509787 | 0.0087628 | 0.03094115 | -3.8114626 |
| Ephx2 | 1448499_a_ 0.6997451 | 11.6585601 | 9.05133287 | 1.57E-05 | 0.00029065 | 3.04919968 |
| Herpud1 | 1435626_a_ 0.70011084 | 11.1663995 | 7.53877668 | 6.03E-05 | 0.00073368 | 1.57400452 |
| Apoe | 1432466_a_ 0.70088627 | 10.6798247 | 1.55781861 | 0.15717726 | 0.26844954 | -6.6921141 |
| F13a1 | 1448929_at 0.70121351 | 10.051692 | 4.5504881 | 0.00178329 | 0.00907232 | -2.1133798 |
| Dbt | 1449118_at 0.70140813 | 9.5332223 | 8.46983239 | 2.57E-05 | 0.00041432 | 2.50764618 |
| Cmtm8 | 1427964_at 0.70319407 | 8.77020499 | 11.0218016 | 3.51E-06 | 0.00010336 | 4.68501349 |
| Oxct1 | 1428140_at 0.70413401 | 12.7966727 | 9.66439789 | 9.58E-06 | 0.00020745 | 3.58914837 |
| Sh3bgr | 1422644_at 0.70468387 | 11.5560289 | 10.7307828 | 4.32E-06 | 0.00011799 | 4.46069703 |
| Trdn | 1451801_at 0.7077916 | 11.2664387 | 9.79244722 | 8.67E-06 | 0.00019199 | 3.69817255 |
| Rwdd4a | 1424243_at 0.70933552 | 9.4311523 | 10.3053889 | 5.88E-06 | 0.00014539 | 4.12269435 |
| Uqcc1 | 1448337_at 0.71086243 | 10.7040375 | 9.74135979 | 9.02E-06 | 0.00019797 | 3.65482566 |
| L3hypdh | 1424692_at 0.71160706 | 7.84561905 | 8.44799612 | 2.62E-05 | 0.00042012 | 2.48671656 |
| Dele1 | 1452685_at 0.71311111 | 10.1760782 | 11.3277507 | 2.85E-06 | 8.88E-05 | 4.91506884 |
| NA | 1448827_s_a0.71383771 | 10.7467753 | 2.64951361 | 0.0287965 | 0.07823187 | -5.0454766 |
| Tuba4a | 1417374_at 0.71456377 | 12.9238742 | 7.05972707 | 9.66E-05 | 0.00103682 | 1.05826827 |
| Plin5 | 1424937_at 0.7146291 | 10.4724853 | 8.2703081 | 3.07E-05 | 0.00046474 | 2.31474392 |
| Cavin2 | 1416778_at 0.71506727 | 11.8393544 | 5.4149871 | 0.00059461 | 0.00393465 | -0.924423 |
| Epas1 | 1449888_at 0.71716746 | 8.90449164 | 5.76611054 | 0.00039214 | 0.00290297 | -0.4714483 |
| Gpm6a | 1456741_s_a0.71742048 | 8.26713146 | 2.14941923 | 0.06319154 | 0.14002109 | -5.8316348 |
| Rgcc | 1438511_a_ 0.71890259 | 9.81241297 | 5.23969855 | 0.00073658 | 0.00458735 | -1.1569652 |
| Adhfe1 | 1424393_s_a0.71896586 | 10.1142219 | 6.67249828 | 0.00014398 | 0.00139913 | 0.62218271 |
| Coq9 | 1428134_at 0.71935418 | 12.7982535 | 9.20019462 | 1.39E-05 | 0.00026722 | 3.18312395 |
| Aes | 1420619_a_ 0.71942496 | 11.3255761 | 3.65659774 | 0.0062243 | 0.02371213 | -3.4501265 |
| Lynx1 | 1417283_at 0.71956233 | 10.6614117 | 8.58694266 | 2.32E-05 | 0.00038367 | 2.6191434 |
| Reep1 | 1433509_s_a0.71991794 | 9.01409033 | 11.9685734 | 1.86E-06 | 6.53E-05 | 5.37884138 |
| Aoc3 | 1449396_at 0.72001232 | 8.68452991 | 2.82301946 | 0.02196517 | 0.06329561 | -4.7684017 |
| Ptgds | 1423859_a_ 0.72024201 | 12.9235077 | 7.3500786 | 7.24E-05 | 0.00083685 | 1.37388826 |
| Gstz1 | 1427552_a_ 0.72142167 | 8.71163932 | 6.22239182 | 0.00023384 | 0.00199321 | 0.09232419 |
| Rnf207 | 1436277_at 0.72196842 | 9.37285332 | 7.83045746 | 4.58E-05 | 0.00060739 | 1.87587459 |
| NA | 1418258_s_a0.72225113 | 12.3225537 | 6.42001826 | 0.00018844 | 0.00170142 | 0.3280955 |
| Zfyve21 | 1424670_s_a0.72351305 | 10.5528305 | 9.31225634 | 1.27E-05 | 0.00025145 | 3.28272783 |
| Rtn4ip1 | 1448800_at 0.72513162 | 8.69789307 | 10.9184046 | 3.78E-06 | 0.00010852 | 4.60594013 |
| Gas1 | 1448494_at 0.72544158 | 9.17232414 | 4.3085742 | 0.00247254 | 0.01168305 | -2.4648898 |
| Lgi1 | 1423183_at 0.725464 | 7.11181762 | 10.9078125 | 3.81E-06 | 0.00010867 | 4.59780124 |
| Ndufs4 | 1418117_at 0.72579445 | 8.21047452 | 8.79050479 | 1.95E-05 | 0.00033866 | 2.80999064 |
| Acad11 | 1433545_s_a0.72666615 | 10.7628898 | 8.78424608 | 1.96E-05 | 0.00033994 | 2.80417792 |
| Ptgr2 | 1453156_s_a0.72667544 | 9.68068014 | 7.41739169 | 6.78E-05 | 0.00079959 | 1.44571871 |
| Ppara | 1449051_at 0.72783331 | 9.2302149 | 4.78645502 | 0.00130734 | 0.00713924 | -1.7783636 |
| Cpeb3 | 1455372_at 0.72784597 | 10.4138861 | 9.89379054 | 8.02E-06 | 0.00018219 | 3.78357764 |
| Mcee | 1438477_a_ 0.72833151 | 10.3573306 | 9.25651041 | 1.33E-05 | 0.00025906 | 3.23330803 |
| Ppp1r3c | 1425631_at 0.72909342 | 9.61784036 | 3.82649585 | 0.00486483 | 0.01964808 | -3.188443 |
| Deptor | 1453571_at 0.72919151 | 8.82060532 | 6.397749 | 0.00019303 | 0.0017312 | 0.30177571 |
| Aldh1a7 | 1418601_at 0.73035042 | 7.63431167 | 3.82583417 | 0.00486946 | 0.0196563 | -3.1894558 |
| Nr1d2 | 1416958_at 0.73035683 | 10.7130413 | 7.00560398 | 0.00010208 | 0.00107882 | 0.99837537 |
| Rhot2 | 1426822_at 0.73110113 | 10.0101567 | 9.48514695 | 1.10E-05 | 0.00022887 | 3.43439913 |
| Gnai1 | 1454959_s_a0.73259408 | 8.07440768 | 4.85920532 | 0.00118996 | 0.00663882 | -1.6766608 |
| Mut | 1448486_at 0.73339705 | 10.5178963 | 9.39215884 | 1.19E-05 | 0.00024105 | 3.35312225 |
| Frmd5 | 1436243_at 0.73467742 | 10.971475 | 10.3360786 | 5.75E-06 | 0.00014306 | 4.14749464 |
| Aldh2 | 1448143_at 0.73478476 | 11.0449385 | 5.23230153 | 0.00074333 | 0.00461331 | -1.1668732 |
| Igfbp4 | 1423756_s_a0.73525041 | 10.7986464 | 2.7993207 | 0.02279014 | 0.06514521 | -4.8062804 |
| Csdc2 | 1437841_x_a0.73778258 | 9.78650364 | 8.17517916 | 3.34E-05 | 0.00049258 | 2.22143922 |
| Apbb1 | 1423893_x_a0.74135502 | 10.6855394 | 7.83916612 | 4.54E-05 | 0.0006039 | 1.88475162 |
| Phkg1 | 1425164_a_ 0.74213787 | 7.57966097 | 8.1573557 | 3.40E-05 | 0.00049572 | 2.20386026 |
| Cavin2 | 1416779_at 0.74238466 | 10.2079122 | 4.6824532 | 0.00149754 | 0.00791847 | -1.9250552 |
| Fblim1 | 1449141_at 0.7451513 | 11.3381783 | 5.80073673 | 0.0003767 | 0.00282462 | -0.4276919 |
| Fabp4 | 1424155_at 0.74569534 | 9.13201775 | 4.70190897 | 0.00145979 | 0.0077553 | -1.8974973 |
| Igfbp3 | 1423062_at 0.74621604 | 7.87623621 | 5.05244328 | 0.00093029 | 0.00549836 | -1.4101641 |
| S100a1 | 1417421_at 0.74996838 | 11.8429394 | 7.54298058 | 6.01E-05 | 0.00073151 | 1.578419 |
| Herpud1 | 1448185_at 0.74999018 | 11.1570642 | 5.59874969 | 0.00047722 | 0.00332946 | -0.6852393 |
| Pdlim3 | 1449178_at 0.75765298 | 8.80525568 | 3.82420788 | 0.00488087 | 0.01968834 | -3.1919455 |
| Pkd2l2 | 1449469_at 0.75817007 | 7.83236438 | 8.25634144 | 3.11E-05 | 0.00046931 | 2.30109959 |
| Gck | 1425303_at 0.75851658 | 8.79672352 | 4.75830147 | 0.00135609 | 0.00734713 | -1.8179225 |
| Igf2 | 1448152_at 0.76140689 | 9.17075055 | 5.41401274 | 0.00059531 | 0.00393809 | -0.9257037 |
| Pdk2 | 1448825_at 0.76217146 | 11.3175242 | 4.47329824 | 0.00197742 | 0.00983509 | -2.2246634 |
| Noct | 1425837_a_ 0.76274862 | 10.0552004 | 5.87751308 | 0.00034479 | 0.00265649 | -0.3312482 |
| Chd6 | 1427384_at 0.7638982 | 9.48924715 | 7.64958052 | 5.43E-05 | 0.00068129 | 1.68972999 |
| Rgcc | 1418003_at 0.7655825 | 8.66064367 | 5.8152871 | 0.00037041 | 0.00279318 | -0.4093533 |
| Sdhaf4 | 1418327_at 0.76656063 | 11.0531316 | 10.8600217 | 3.94E-06 | 0.000111 | 4.56098927 |
| Endog | 1421097_at 0.76816268 | 10.7278302 | 10.0707963 | 7.01E-06 | 0.00016673 | 3.93091619 |
| NA | 1425546_a_ 0.76904765 | 8.89910823 | 3.94045678 | 0.00413302 | 0.01732143 | -3.0147693 |

| Dhrs4 | 1451559_a_ | 0.76967523 | 10.2667988 | 7.56992971 | 5.86E-05 | 0.00071828 | 1.60667314 |
| --- | --- | --- | --- | --- | --- | --- | --- |
| Acaa2 | 1455061_a_ | 0.77014851 | 12.1867434 | 6.11086234 | 0.0002647 | 0.00219038 | -0.0429462 |
| Tpm1 | 1456623_at | 0.77077929 | 8.63989072 | 3.21498854 | 0.01202273 | 0.03970825 | -4.1433335 |
| Fmo2 | 1422904_at | 0.77256658 | 8.82988774 | 8.19662963 | 3.28E-05 | 0.00048602 | 2.24255457 |
| Auh | 1420776_a_ | 0.77382866 | 10.6871553 | 8.22341671 | 3.20E-05 | 0.00047918 | 2.26886069 |
| Pdha1 | 1449137_at | 0.77471876 | 12.0659031 | 7.94202977 | 4.13E-05 | 0.000567 | 1.98902024 |
| 6720475J19 | 1423071_x_a0.77505514 | | 12.3317277 | 11.7854469 | 2.10E-06 | 7.09E-05 | 5.24872164 |
| Mme | 1455961_at 0.77565978 | | 7.15693378 | 4.79963265 | 0.00128518 | 0.00704194 | -1.759886 |
| Ckm | 1417614_at 0.77591897 | | 13.7222246 | 10.84069 | 3.99E-06 | 0.00011197 | 4.54605662 |
| Pnkd | 1418746_at 0.77596653 | | 9.13275108 | 6.2844846 | 0.0002184 | 0.00189936 | 0.16693834 |
| NA | 1417729_at 0.77627532 | | 9.90822131 | 3.20404493 | 0.01222416 | 0.04019799 | -4.1607015 |
| Hsdl2 | 1426856_at 0.77973917 | | 10.9105642 | 10.6037219 | 4.73E-06 | 0.0001258 | 4.36102205 |
| Fbln1 | 1451119_a_ 0.78132739 | | 10.1063188 | 3.6797395 | 0.00601743 | 0.02315339 | -3.4142974 |
| Hamp | 1419197_x_a0.7858484 | | 8.27151496 | 2.53168063 | 0.03464236 | 0.08922079 | -5.2329679 |
| Dlat | 1452005_at 0.78688962 | | 10.7165687 | 12.5543883 | 1.28E-06 | 5.03E-05 | 5.78283019 |
| Apbb1 | 1423892_at 0.79276144 | | 10.0521686 | 6.01974935 | 0.00029324 | 0.00236531 | -0.154659 |
| Retn | 1449182_at 0.79752128 | | 8.62018015 | 3.78794332 | 0.0051428 | 0.02049735 | -3.2475407 |
| Kcnk3 | 1426058_a_ 0.79817085 | | 9.67715704 | 4.56538899 | 0.00174824 | 0.00892813 | -2.0919928 |
| 6720475J19 | 1423072_at 0.79886472 | | 11.6719896 | 6.74191444 | 0.00013389 | 0.00131945 | 0.70166924 |
| Gpx3 | 1449106_at 0.79911416 | | 12.1458445 | 2.11843176 | 0.06633865 | 0.14527787 | -5.8791657 |
| Pxmp2 | 1417841_at 0.79941974 | | 10.9599394 | 10.6125329 | 4.70E-06 | 0.00012558 | 4.36796877 |
| Etfbkmt | 1427202_at 0.79988484 | | 9.0948313 | 9.83588137 | 8.39E-06 | 0.00018822 | 3.73487032 |
| Homer2 | 1424367_a_ 0.80085313 | | 10.0406997 | 6.43909305 | 0.0001846 | 0.00167869 | 0.3505902 |
| Smim10l1 | 1453208_at 0.80088583 | | 10.2859651 | 6.51229316 | 0.00017064 | 0.00158674 | 0.43649188 |
| Htra3 | 1419292_at 0.80629863 | | 10.6028311 | 3.46845712 | 0.00821464 | 0.02948872 | -3.7434062 |
| Ndufa10 | 1418068_at 0.80734357 | | 10.0584344 | 8.42484406 | 2.68E-05 | 0.00042524 | 2.46447735 |
| Mrps35 | 1452111_at 0.80828981 | | 10.4612686 | 8.63164331 | 2.24E-05 | 0.00037365 | 2.66137108 |
| P2ry1 | 1421456_at 0.81047404 | | 8.31042828 | 11.849477 | 2.01E-06 | 6.91E-05 | 5.29443211 |
| Deptor | 1451348_at 0.81344205 | | 8.05089008 | 4.13939705 | 0.00312333 | 0.01401115 | -2.7154197 |
| Pkia | 1420858_at 0.81469231 | | 10.0022272 | 7.58640009 | 5.76E-05 | 0.00071112 | 1.62390298 |
| Crat | 1417008_at 0.81533695 | | 11.030842 | 9.53440727 | 1.06E-05 | 0.00022237 | 3.47717703 |
| Rbm38 | 1421265_a_ 0.81648996 | | 10.7286448 | 5.71343244 | 0.00041697 | 0.00302272 | -0.5383284 |
| Hsdl2 | 1426857_a_ 0.81771099 | | 10.9478698 | 10.1917545 | 6.40E-06 | 0.00015435 | 4.03028916 |
| Bdh1 | 1426959_at 0.81908914 | | 8.87328668 | 3.4519073 | 0.00841946 | 0.02999021 | -3.7693634 |
| Sult1a1 | 1427345_a_ 0.82374096 | | 9.46050348 | 7.37276763 | 7.08E-05 | 0.00082312 | 1.39815545 |
| Tfrc | 1422966_a_ 0.83077522 | | 8.9466321 | 6.66462365 | 0.00014518 | 0.00140655 | 0.61312882 |
| Acsl1 | 1450643_s_a0.83203976 | | 11.734554 | 8.50065567 | 2.50E-05 | 0.00040476 | 2.53711445 |
| Fgf12 | 1451693_a_ 0.84183475 | | 8.34124896 | 3.52931706 | 0.00750565 | 0.02743727 | -3.6481635 |
| Cpeb3 | 1437765_at 0.84212015 | | 9.70529926 | 7.12538996 | 9.05E-05 | 0.0009898 | 1.13047919 |
| Ano10 | 1426672_at 0.84541384 | | 9.79233214 | 9.5676499 | 1.03E-05 | 0.00021831 | 3.50593708 |
| Fkbp4 | 1416362_a_ 0.84601913 | | 10.7290917 | 5.70051179 | 0.00042332 | 0.00305607 | -0.5547901 |
| Mgl2 | 1438467_at 0.84668782 | | 7.95935205 | 10.9328372 | 3.74E-06 | 0.00010758 | 4.61701846 |
| Pam | 1418908_at 0.85066649 | | 10.1517748 | 4.26295599 | 0.00263225 | 0.01226148 | -2.5320694 |
| Ak1 | 1422184_a_ 0.85563373 | | 12.1667788 | 8.56961417 | 2.36E-05 | 0.00038814 | 2.60272471 |
| Tmem45b | 1424357_at 0.86096394 | | 7.38987918 | 2.51794544 | 0.035398 | 0.09052984 | -5.2547644 |
| Gpd1 | 1416204_at 0.86239327 | | 8.879437 | 2.60086173 | 0.0310778 | 0.08246753 | -5.1229863 |
| Slc25a42 | 1424790_at 0.86332593 | | 9.36373743 | 6.08859926 | 0.00027138 | 0.00222939 | -0.0701422 |
| Kcnk3 | 1425342_a_ 0.87095221 | | 8.88612781 | 4.59344876 | 0.00168426 | 0.00867557 | -2.0518035 |
| Reg3g | 1448872_at 0.8715889 | | 7.49189613 | 1.7195158 | 0.12311924 | 0.22539742 | -6.4688433 |
| Reep5 | 1426376_at 0.87241156 | | 10.7038814 | 11.0254486 | 3.51E-06 | 0.00010332 | 4.68779013 |
| Decr1 | 1449443_at 0.87346676 | | 10.7171691 | 9.69140048 | 9.38E-06 | 0.0002037 | 3.61224345 |
| Fmo1 | 1417429_at 0.87787484 | | 9.99660674 | 4.60882807 | 0.00165027 | 0.00854707 | -2.0298229 |
| Ppargc1b | 1449945_at 0.87850926 | | 10.5908436 | 9.04811341 | 1.57E-05 | 0.00029095 | 3.04628268 |
| Rab3a | 1422589_at 0.89422028 | | 9.45636678 | 13.4931816 | 7.30E-07 | 3.35E-05 | 6.39408912 |
| Pdlim7 | 1417959_at 0.8960544 | | 9.86958491 | 11.3681351 | 2.77E-06 | 8.72E-05 | 4.94500681 |
| Dgat2 | 1422678_at 0.8966578 | | 12.514531 | 8.14720658 | 3.43E-05 | 0.00049854 | 2.19383655 |
| Tmem38a | 1424177_at 0.89983342 | | 10.7058112 | 6.82911094 | 0.0001223 | 0.00123771 | 0.80069451 |
| Ighm | 1427329_a_ 0.90024981 | | 8.56026237 | 1.0289807 | 0.33303856 | 0.45870128 | -7.3247204 |
| Idh3a | 1422501_s_a0.90125649 | | 10.9654498 | 10.7892655 | 4.14E-06 | 0.00011443 | 4.50621599 |
| Vegfb | 1451803_a_ 0.90338749 | | 11.4307636 | 6.54401628 | 0.00016496 | 0.00154474 | 0.47351248 |
| Itgb1bp2 | 1423238_at 0.90509706 | | 11.5550329 | 14.0302683 | 5.37E-07 | 2.68E-05 | 6.72546295 |
| Pik3r1 | 1425515_at 0.91158695 | | 9.23913973 | 10.0014244 | 7.39E-06 | 0.00017283 | 3.87344585 |
| Tuba8 | 1419518_at 0.91178078 | | 11.3769831 | 5.62934513 | 0.00046026 | 0.00324792 | -0.6458697 |
| Acsl1 | 1422526_at 0.9164383 | | 10.9961085 | 6.99442927 | 0.00010325 | 0.00108506 | 0.98596718 |
| Dgat2 | 1422677_at 0.91693651 | | 11.0791556 | 8.37037316 | 2.81E-05 | 0.00043905 | 2.41195644 |
| Gstm1 | 1448330_at 0.91892257 | | 11.0144025 | 9.98124464 | 7.50E-06 | 0.00017479 | 3.85666202 |
| Hp | 1448881_at 0.92254345 | | 9.59385088 | 2.37998363 | 0.04397553 | 0.10701467 | -5.4727632 |
| Kcnk3 | 1425341_at 0.92305374 | | 10.8919824 | 5.41837578 | 0.00059218 | 0.00392424 | -0.9199699 |
| Car4 | 1448949_at 0.92662189 | | 8.47098924 | 9.26845909 | 1.31E-05 | 0.00025759 | 3.24392217 |
| Ces1d | 1435371_x_a0.93239148 | | 8.27969085 | 6.00415362 | 0.00029846 | 0.00239885 | -0.1738903 |
| NA | 1428333_at 0.93365748 | | 6.93610118 | 4.83830572 | 0.00122246 | 0.00678845 | -1.7058011 |
| Gpd1 | 1448249_at 0.93380718 | | 9.87923169 | 1.91139438 | 0.09163672 | 0.18301264 | -6.191022 |
| Idh3a | 1422500_at 0.93716896 | | 9.89390503 | 13.226622 | 8.53E-07 | 3.70E-05 | 6.22481317 |
| Gstm7 | 1419072_at 0.93992487 | | 9.40715935 | 13.9052507 | 5.76E-07 | 2.83E-05 | 6.64945439 |
| Gstk1 | 1452823_at 0.94043473 | | 10.7484916 | 10.7922026 | 4.13E-06 | 0.00011443 | 4.50849614 |
| Sod2 | 1417194_at 0.94281415 | | 8.82187268 | 9.36707091 | 1.21E-05 | 0.00024421 | 3.33107528 |
| Per3 | 1421087_at 0.94415696 | | 8.09685089 | 11.8170739 | 2.05E-06 | 7.01E-05 | 5.27132881 |
| Adig | 1424729_at 0.95642055 | | 7.66661224 | 3.57137379 | 0.00705378 | 0.0260753 | -3.5825497 |
| Gstm1 | 1416416_x_a0.96567718 | | 11.6394075 | 10.4798305 | 5.17E-06 | 0.00013297 | 4.26279002 |
| Ctsf | 1451019_at 0.97059844 | | 9.34891743 | 8.74645738 | 2.03E-05 | 0.00034758 | 2.7690084 |
| Trp53inp2 | 1452646_at 0.97783874 | | 12.4771963 | 8.06792366 | 3.68E-05 | 0.00052271 | 2.11518657 |
| Retnla | 1449015_at 0.98230612 | | 8.47833667 | 5.34790873 | 0.00064506 | 0.00418301 | -1.0129021 |
| Rgs5 | 1420942_s_a0.98788808 | | 10.7481133 | 5.78769157 | 0.00038244 | 0.00285537 | -0.4441578 |
| Amd1 | 1448484_at 0.98819704 | | 11.2612863 | 11.2163708 | 3.07E-06 | 9.42E-05 | 4.83198669 |
| Rgs5 | 1420941_at 0.98955099 | | 10.4178642 | 7.82817145 | 4.59E-05 | 0.00060798 | 1.8735431 |

| Wnk2 | 1453355_at 0.99720084 | 9.08401912 | 12.4172022 | 1.40E-06 | 5.36E-05 | 5.68985227 |
| --- | --- | --- | --- | --- | --- | --- |
| Tmem38a | 1424178_at 0.99985518 | 10.3989582 | 8.39519147 | 2.75E-05 | 0.00043283 | 2.43592078 |
| Ighm | 1427351_s_a1.00069091 | 9.47513184 | 1.07840793 | 0.3117135 | 0.43794797 | -7.2733435 |
| Npr3 | 1435184_at 1.00308553 | 8.44208087 | 4.11952989 | 0.0032111 | 0.01430273 | -2.7450877 |
| Igfbp6 | 1417933_at 1.00900141 | 9.51056343 | 4.97531235 | 0.00102568 | 0.00592274 | -1.5159011 |
| Cdkn1c | 1417649_at 1.00908757 | 8.99385793 | 7.24508184 | 8.03E-05 | 0.0009026 | 1.26084702 |
| NA | AFFX-MURIN 1.00928953 | 12.5652566 | 7.87081793 | 4.41E-05 | 0.00059231 | 1.91695016 |
| Pcp4l1 | 1452913_at 1.01228957 | 10.3550952 | 8.80317028 | 1.93E-05 | 0.00033581 | 2.82174303 |
| Per2 | 1417602_at 1.01345324 | 7.639036 | 11.9680333 | 1.86E-06 | 6.53E-05 | 5.37846035 |
| Synpo2 | 1450828_at 1.01475231 | 9.06228246 | 8.19910177 | 3.27E-05 | 0.00048526 | 2.24498523 |
| Kcnip2 | 1425870_a_ 1.01710205 | 8.68837675 | 7.00623216 | 0.00010202 | 0.00107882 | 0.99907245 |
| Hamp | 1419196_at 1.0241461 | 8.17171584 | 2.65732693 | 0.02844638 | 0.07746619 | -5.0330185 |
| Ptgds | 1423860_at 1.03474229 | 11.6715079 | 8.21217808 | 3.23E-05 | 0.00048149 | 2.25783228 |
| Fmod | 1456084_x_a1.03908024 | 7.03616848 | 7.65584896 | 5.40E-05 | 0.00067913 | 1.69623802 |
| Fmo2 | 1422905_s_a1.04449909 | 8.59236478 | 6.20226281 | 0.00023911 | 0.00202211 | 0.06802958 |
| Gpcpd1 | 1429144_at 1.04532723 | 11.5258819 | 12.4767539 | 1.35E-06 | 5.24E-05 | 5.73033301 |
| Car4 | 1418094_s_a1.0483459 | 8.72820013 | 8.72905111 | 2.06E-05 | 0.00035066 | 2.75276596 |
| Gsn | 1437171_x_a1.04979989 | 12.0760261 | 6.14092787 | 0.00025596 | 0.00213286 | -0.0063217 |
| Upk3b | 1434237_at 1.05647076 | 9.04862449 | 3.86474601 | 0.00460484 | 0.01884828 | -3.1299789 |
| Gsn | 1436991_x_a1.05729292 | 11.7660588 | 6.60678847 | 0.00015432 | 0.0014756 | 0.54640084 |
| Ryr2 | 1421126_at 1.06048293 | 9.44396295 | 4.89397045 | 0.00113795 | 0.00640539 | -1.6283251 |
| Amd1 | 1416835_s_a1.06074096 | 10.1778268 | 9.49184104 | 1.10E-05 | 0.00022812 | 3.44022358 |
| Gsn | 1456312_x_a1.06434956 | 12.4568528 | 6.4004568 | 0.00019247 | 0.00172749 | 0.30497936 |
| Lyve1 | 1429379_at 1.06640301 | 9.58086628 | 10.4938561 | 5.12E-06 | 0.00013236 | 4.27396307 |
| Slc2a4 | 1415958_at 1.07145702 | 11.8329601 | 8.37996827 | 2.78E-05 | 0.00043716 | 2.42122826 |
| Car14 | 1450725_s_a1.07550451 | 10.9782653 | 10.2078004 | 6.32E-06 | 0.00015308 | 4.04339281 |
| Hlf | 1434736_at 1.08064276 | 8.38647529 | 8.67330493 | 2.16E-05 | 0.00036367 | 2.70056519 |
| G0s2 | 1448700_at 1.08891166 | 10.1807868 | 5.72303155 | 0.00041232 | 0.00299762 | -0.5261133 |
| Scgb3a2 | 1425218_a_ 1.0905875 | 8.677095 | 1.96321794 | 0.08454673 | 0.17273234 | -6.1139954 |
| Cbr2 | 1418509_at 1.09287074 | 9.36318374 | 3.51355932 | 0.00768277 | 0.02794071 | -3.6727909 |
| Usp2 | 1417169_at 1.09690314 | 10.8376158 | 12.0605569 | 1.75E-06 | 6.29E-05 | 5.44349588 |
| Ank1 | 1425677_a_ 1.13158862 | 11.476741 | 7.061515 | 9.65E-05 | 0.00103562 | 1.06024104 |
| Scgb3a1 | 1419699_at 1.13308487 | 8.47347507 | 2.01175656 | 0.07838666 | 0.16368427 | -6.0411889 |
| Ces1d | 1435370_a_ 1.13645188 | 9.86840211 | 6.03878001 | 0.00028701 | 0.0023308 | -0.1312355 |
| Msrb2 | 1424433_at 1.13907926 | 9.81269677 | 12.7109493 | 1.16E-06 | 4.70E-05 | 5.88776212 |
| Abca8a | 1427371_at 1.13911636 | 10.4209715 | 7.17605075 | 8.60E-05 | 0.00095013 | 1.18585616 |
| Pck1 | 1423439_at 1.14117783 | 6.904407 | 3.25920101 | 0.01124373 | 0.03766725 | -4.073241 |
| Smoc2 | 1415935_at 1.15145379 | 9.5912578 | 8.011952 | 3.87E-05 | 0.00054194 | 2.05928869 |
| NA | 1448194_a_ 1.15292634 | 8.83662051 | 4.63053256 | 0.00160356 | 0.00834324 | -1.9988585 |
| Timp4 | 1450974_at 1.15353408 | 8.54315176 | 11.0634873 | 3.41E-06 | 0.00010153 | 4.71670109 |
| Nrep | 1436736_x_a1.1625039 | 8.91365107 | 7.22636045 | 8.18E-05 | 0.0009154 | 1.24056225 |
| Thrsp | 1422973_a_ 1.17561222 | 9.08935288 | 2.68715311 | 0.02714934 | 0.07469608 | -4.985439 |
| Mgp | 1448416_at 1.18430971 | 12.1537795 | 5.95399798 | 0.00031593 | 0.00249532 | -0.2359562 |
| Slc2a4 | 1415959_at 1.18438419 | 11.0975328 | 8.71473692 | 2.08E-05 | 0.0003539 | 2.73938864 |
| Scd1 | 1415965_at 1.19121168 | 7.6532404 | 2.53477427 | 0.03447443 | 0.0889296 | -5.2280566 |
| Upk3b | 1454881_s_a1.19230378 | 9.30381807 | 3.8408662 | 0.00476536 | 0.0193151 | -3.1664583 |
| Cidec | 1452260_at 1.19819687 | 7.24563878 | 3.70379812 | 0.00581011 | 0.02253912 | -3.3771091 |
| Myot | 1418155_at 1.2080048 | 9.66114741 | 7.4953099 | 6.29E-05 | 0.00075808 | 1.52824941 |
| Nrep | 1450839_at 1.22091296 | 8.87086584 | 10.3975631 | 5.50E-06 | 0.0001384 | 4.19698273 |
| Usp2 | 1417168_a_ 1.22559568 | 11.1821793 | 10.9640601 | 3.66E-06 | 0.00010619 | 4.64093937 |
| Efnb3 | 1423085_at 1.23005898 | 9.35068795 | 16.8248379 | 1.28E-07 | 9.96E-06 | 8.26772745 |
| D7Wsu130e | 1447462_at 1.23874194 | 7.63582552 | 9.44690372 | 1.14E-05 | 0.00023301 | 3.40105594 |
| Mtfp1 | 1424223_at 1.25393099 | 10.9239706 | 18.3193781 | 6.48E-08 | 6.45E-06 | 8.98769145 |
| Stard10 | 1448956_at 1.25687814 | 10.4171868 | 3.94326826 | 0.00411653 | 0.01727145 | -3.0105043 |
| Ces1d | 1449081_at 1.26263767 | 10.1991562 | 7.09224703 | 9.35E-05 | 0.00101543 | 1.09409285 |
| Tef | 1424175_at 1.26898762 | 10.9001854 | 17.2923534 | 1.03E-07 | 8.82E-06 | 8.49991309 |
| Ltbp4 | 1436665_a_ 1.29130802 | 9.92212567 | 6.73205165 | 0.00013527 | 0.00133091 | 0.69041111 |
| Gsn | 1415812_at 1.30140173 | 12.6738232 | 5.61200702 | 0.00046979 | 0.00329099 | -0.6681642 |
| Inmt | 1418697_at 1.34810618 | 8.40627053 | 6.53038031 | 0.00016738 | 0.00156222 | 0.4576147 |
| Cyp2f2 | 1448792_a_ 1.35829071 | 7.3863626 | 2.22815568 | 0.05584035 | 0.1276592 | -5.7100382 |
| Adh1 | 1416225_at 1.37022796 | 8.88497383 | 7.29922933 | 7.61E-05 | 0.00086701 | 1.31929591 |
| Myl9 | 1452670_at 1.39280667 | 9.41156927 | 4.66900074 | 0.00152426 | 0.00802262 | -1.944141 |
| NA | 1442026_at 1.40576214 | 9.20831697 | 6.5345865 | 0.00016663 | 0.00155714 | 0.46252103 |
| Nppa | 1456062_at 1.41037528 | 13.4890458 | 2.44306085 | 0.03982076 | 0.09915858 | -5.3733233 |
| Dbp | 1438211_s_a1.44233464 | 9.07095509 | 6.95499994 | 0.00010747 | 0.00111773 | 0.9420696 |
| Tfrc | 1452661_at 1.4549965 | 10.9319839 | 6.95734118 | 0.00010722 | 0.00111773 | 0.94468122 |
| Acta1 | 1427735_a_ 1.51812896 | 12.9600932 | 10.0375151 | 7.19E-06 | 0.0001695 | 3.90338863 |
| Thrsp | 1424737_at 1.62481166 | 10.4733058 | 3.45939199 | 0.00832617 | 0.02977007 | -3.7576212 |
| Zbtb16 | 1419874_x_a1.64096114 | 9.29352939 | 9.56308949 | 1.04E-05 | 0.0002188 | 3.50199675 |
| Cd163 | 1419144_at 1.64387719 | 7.36415404 | 17.1871789 | 1.08E-07 | 9.08E-06 | 8.44825404 |
| Scgb1a1 | 1452543_a_ 1.64402028 | 7.9598935 | 1.69345126 | 0.12810475 | 0.23175704 | -6.5055673 |
| Dbp | 1418174_at 1.66526487 | 9.14205557 | 13.1195113 | 9.09E-07 | 3.89E-05 | 6.15585974 |
| NA | 1442025_a_ 1.79254323 | 9.65190488 | 10.4987967 | 5.10E-06 | 0.00013234 | 4.27789562 |
| Bpifa1 | 1420347_at 1.83534394 | 7.8037026 | 1.64010659 | 0.1388946 | 0.24598178 | -6.579878 |
| Timp4 | 1423405_at 1.85651065 | 8.44505126 | 15.1609509 | 2.92E-07 | 1.75E-05 | 7.38388166 |
| Aqp1 | 1416203_at 1.8895681 | 11.5319137 | 13.4379127 | 7.54E-07 | 3.43E-05 | 6.35925998 |
| Fasn | 1423828_at 1.90009073 | 9.85258671 | 3.34670153 | 0.00985429 | 0.03401186 | -3.9349096 |
| Acta2 | 1416454_s_a2.04153057 | 11.903868 | 13.8936618 | 5.80E-07 | 2.83E-05 | 6.64237457 |
| Cdo1 | 1448842_at 2.04795235 | 8.47327735 | 4.54638146 | 0.00179308 | 0.0091128 | -2.1192794 |
| Cyp2e1 | 1415994_at 2.18491318 | 9.13874989 | 6.11779826 | 0.00026265 | 0.00217584 | -0.0344867 |
| Ucp1 | 1418197_at 2.19039665 | 8.52019893 | 2.57414561 | 0.03240797 | 0.08503935 | -5.1654948 |
| Car3 | 1460256_at 2.53400187 | 9.27192428 | 5.65322127 | 0.00044749 | 0.00318493 | -0.6152355 |
| Cfd | 1417867_at 2.64229611 | 9.71826775 | 5.22817034 | 0.00074714 | 0.00463057 | -1.1724101 |
| Adipoq | 1422651_at 3.15316692 | 8.96143461 | 5.86726318 | 0.00034888 | 0.00267524 | -0.3440779 |
| Scd1 | 1415964_at 3.18419729 | 10.1200799 | 5.51560849 | 0.00052685 | 0.00359093 | -0.7928755 |

| Sln | 1420884_at | 3.39074779 | 10.3996108 | 3.38749904 | 0.00926943 | 0.03238234 | -3.8706061 |
| --- | --- | --- | --- | --- | --- | --- | --- |
| Myl4 | 1422580_at | 3.55429222 | 11.1950595 | 2.93118185 | 0.01857349 | 0.05559797 | -4.5955255 |
| Myl7 | 1449071_at | 3.70611847 | 11.1165693 | 2.96598495 | 0.01760138 | 0.05335676 | -4.5399276 |
