# Supplementary figures and images for: Circulating miR-146a as a possible candidate biomarker in the indeterminate phase of Chagas disease
Source: Biol Res. 2021 Jul 21;54:21. doi: 10.1186/s40659-021-00345-3 (PMC8293491; doi:10.1186/s40659-021-00345-3)

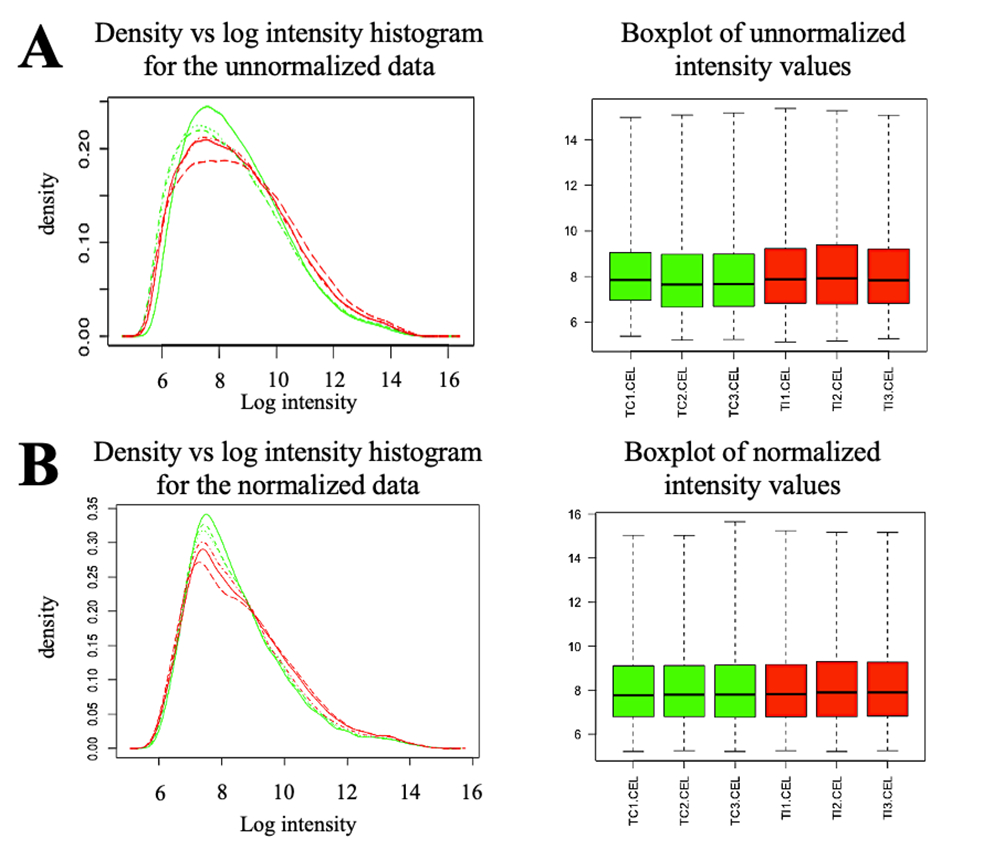

Supplement: Supplementary file 3 — Additional file 3: Figure S1. Intensity histogram and box plot of gene expression in different samples before and after normalization. (A) Gene expression in histogram and box plot of heart tissue samples from control (TC) and T. cruzi infected (TI) mice before normalization. (B) Gene expression in histogram and box plot of heart tissue samples from control mice and T. cruzi-infected mice after normalization. [file 40659_2021_345_MOESM3_ESM.tif]

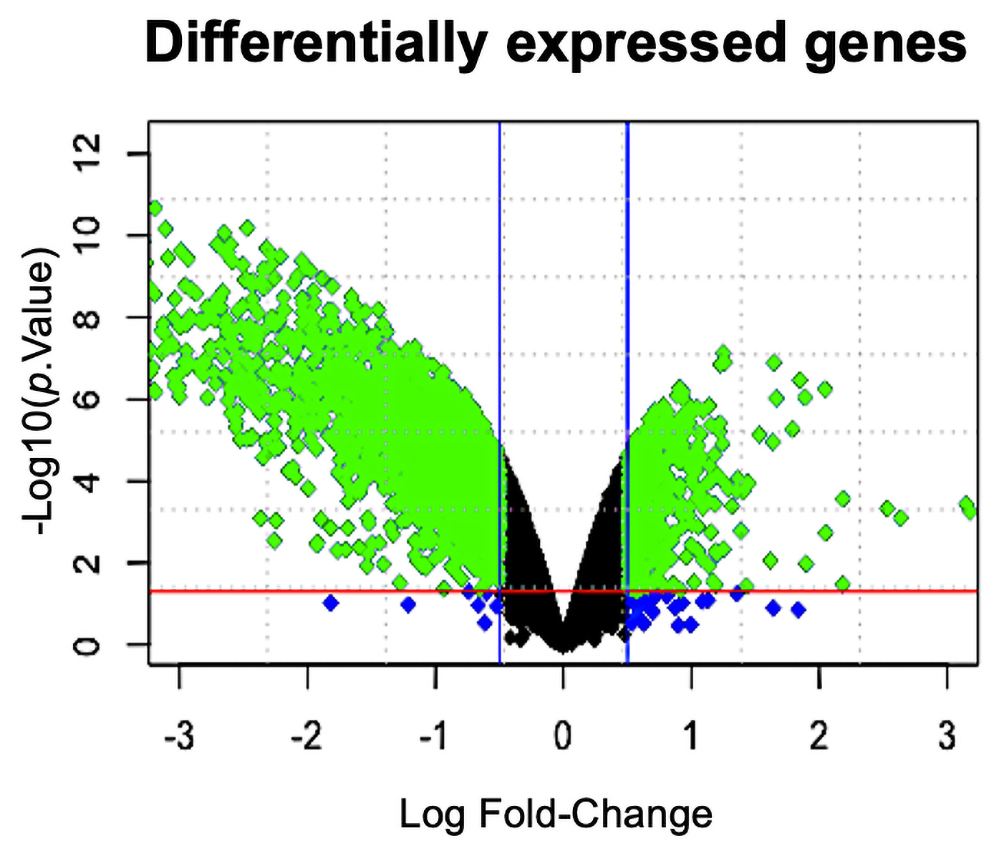

Supplement: Supplementary file 4 — Additional file 4: Figure S2. Volcano plot of the differentially expressed genes that were evaluated by microarray analysis in cardiac tissue from healthy mice and T. cruzi-infected mice. The y-axis indicates the—log10 of the P—values and the x-axis is the CF (measured as the log2 transformed ratio of expression between both experimental groups). [file 40659_2021_345_MOESM4_ESM.tif]
