# Supplementary material for: Circulating miR-146a as a possible candidate biomarker in the indeterminate phase of Chagas disease
Source: Biol Res. 2021 Jul 21;54:21. doi: 10.1186/s40659-021-00345-3 (PMC8293491; doi:10.1186/s40659-021-00345-3)
Supplement: Supplementary file 5 — Additional file 5: Table S3. Oligonucleotide Design for RT-qPCR. [file 40659_2021_345_MOESM5_ESM.docx]

Table S3. Oligonucleotide Design for RT-qPCR.

| **Gene** | **Oligonucleotide forward** | **Oligonucleotide reverse** |
| --- | --- | --- |
| *Gsk3b* | AAG GCA CAT CCT TGG ACA AA | GGT GTG TAC TCC AGC AGA CG |
| *Smad5* | TGA GCT CAC CAA GAT GTG TAC C | GTA CTG GTG ACG TCC TGT CG |
| *Nfatc1* | TCC AAA GTC ATT TTC GTG GA | CTT TGC TTC CAT CTC CCA GA |
| *Creb1* | GGA GCT TGT ACC ACC GGT AA | GCA TCT CCA CTC TGC TGG TT |
| *Igf1r* | GAG AAT TTC CTT CAC AAT TCC ATC | CAC TTG CAT GAC GTC TCT CC |
| *Pdpk1* | TGA TGA AGA CTG CTA TGG CAA C | CGT AGA CAG GGA GTG GGA AG |
| *Map3k1* | CTA GCA CAT CCA CAT CTA GTT CAG A | CAG CAA GCA GAT GGG ACA C |
| *Iqgap1* | GCC AGG AGA GAC CTT GAC TG | GCC CTC TGA TGT TCA GCT TC |
| *Tiam1* | AAG ACT GGT CCC TGA CTT GG | CCC CCA GAG AGA AGA GCA C |
| *Nfat5* | TCC TCC AGC TGT AGT TGC TG | GGT GGT AAA GGA GCT GCA AG |
| *Bcl2* | AGT ACC TGA ACC GGC ATC TG | GGG GCC ATA TAG TTC CAC AAA |

***GSK3B***; glycogen synthase kinase 3 beta, ***Smad5***; mothers against decapentaplegic homolog 5, ***Nfatc1***; nuclear factor of activated T-cells, cytoplasmic 1, ***Creb1***; cyclic AMP-responsive element-binding protein 1, ***Igf1r***; insulin like growth factor 1 receptor, ***Pdpk1***; 3-phosphoinositide dependent protein kinase 1, ***Map3k1***; mitogen-activated protein kinase kinase kinase 1, ***Iqgap1***; Ras GTPase-activating-like protein 1, ***Tiam1***; T-lymphoma invasion and metastasis-inducing protein 1, ***Nfat5***; nuclear factor of activated T-cells 5, ***Bcl2***; apoptosis regulator Bcl2.
